# RNA-Dependent Cysteine Biosynthesis in Bacteria and Archaea

**DOI:** 10.1128/mBio.00561-17

**Published:** 2017-05-09

**Authors:** Takahito Mukai, Ana Crnković, Takuya Umehara, Natalia N. Ivanova, Nikos C. Kyrpides, Dieter Söll

**Affiliations:** a Department of Molecular Biophysics and Biochemistry, Yale University, New Haven, Connecticut, USA; b Department of Biological Science and Technology, Tokyo University of Science, Katsushika-ku, Tokyo, Japan; c Department of Energy Joint Genome Institute (DOE JGI), Walnut Creek, California, USA; d Department of Chemistry, Yale University, New Haven, Connecticut, USA; University of Washington

**Keywords:** biochemistry, bioinformatics, cysteine biosynthesis, genetic code, translation

## Abstract

The diversity of the genetic code systems used by microbes on earth is yet to be elucidated. It is known that certain methanogenic archaea employ an alternative system for cysteine (Cys) biosynthesis and encoding; tRNA^Cys^ is first acylated with phosphoserine (Sep) by *O*-phosphoseryl-tRNA synthetase (SepRS) and then converted to Cys-tRNA^Cys^ by Sep-tRNA:Cys-tRNA synthase (SepCysS). In this study, we searched all genomic and metagenomic protein sequence data in the Integrated Microbial Genomes (IMG) system and at the NCBI to reveal new clades of SepRS and SepCysS proteins belonging to diverse archaea in the four major groups (DPANN, *Euryarchaeota*, TACK, and Asgard) and two groups of bacteria (“*Candidatus* Parcubacteria” and *Chloroflexi*). Bacterial SepRS and SepCysS charged bacterial tRNA^Cys^ species with cysteine *in vitro*. Homologs of SepCysE, a scaffold protein facilitating SepRS⋅SepCysS complex assembly in Euryarchaeota class I methanogens, are found in a few groups of TACK and Asgard archaea, whereas the C-terminally truncated homologs exist fused or genetically coupled with diverse SepCysS species. Investigation of the selenocysteine (Sec)- and pyrrolysine (Pyl)-utilizing traits in SepRS-utilizing archaea and bacteria revealed that the archaea carrying full-length SepCysE employ Sec and that SepRS is often found in Pyl-utilizing archaea and *Chloroflexi* bacteria. We discuss possible contributions of the SepRS-SepCysS system for sulfur assimilation, methanogenesis, and other metabolic processes requiring large amounts of iron-sulfur enzymes or Pyl-containing enzymes.

## IMPORTANCE

Comprehensive analyses of all genomic and metagenomic protein sequence data in public databases revealed the distribution and evolution of an alternative cysteine-encoding system in diverse archaea and bacteria. The finding that the SepRS-SepCysS-SepCysE- and the selenocysteine-encoding systems are shared by the *Euryarchaeota* class I methanogens, the *Crenarchaeota* AK8/W8A-19 group, and an Asgard archaeon suggests that ancient archaea may have used both systems. In contrast, bacteria may have obtained the SepRS-SepCysS system from archaea. The SepRS-SepCysS system sometimes coexists with a pyrrolysine-encoding system in both archaea and bacteria. Our results provide additional bioinformatic evidence for the contribution of the SepRS-SepCysS system for sulfur assimilation and diverse metabolisms which require vast amounts of iron-sulfur enzymes and proteins. Among these biological activities, methanogenesis, methylamine metabolism, and organohalide respiration may have local and global effects on earth. Taken together, uncultured bacteria and archaea provide an expanded record of the evolution of the genetic code.

## INTRODUCTION

Two minor genetic code systems were discovered in methanogenic archaea a decade ago (1–3). In most organisms, Cys biosynthesis and Cys-tRNA^Cys^ formation are carried out separately by a cysteine synthase and cysteinyl-tRNA synthetase (CysRS), respectively. However, methanogens employ a tRNA^Cys^-dependent Cys biosynthesis pathway (3). In these archaea, Cys-tRNA^Cys^ is formed in a two-step process; first, *O*-phosphoserine (Sep) is acylated to tRNA^Cys^ by SepRS and then Sep-tRNA^Cys^ is converted by Sep-tRNA:Cys-tRNA synthase (SepCysS) to Cys-tRNA^Cys^ (3–6). An additional component, SepCysE, stabilizes the SepRS⋅SepCysS⋅tRNA^Cys^ ternary complex, but it is known to be present in class I methanogens only (4, 7). The class I methanogens are also exceptional among methanogens in that they encode selenocysteine (Sec), the 21st genetically encoded amino acid used in some archaea and many bacteria and eukaryotes (8, 9). The coupled biosynthesis and coding of Cys are considered as the original mechanism of Cys-tRNA^Cys^ formation in the last common ancestor of archaea (3, 4) because archaeal CysRS genes appear to have multiple bacterial origins (10, 11) and bacterial CysRS is a highly evolved Cys-specific enzyme using a zinc atom to ensure specificity (12, 13). However, our knowledge is confined to well-studied lineages of cultured archaea, and it remains unclear whether the SepRS-SepCysS pathway is present outside the major *Euryarchaeota* clade, which includes class I, II, and III methanogens, methanotrophic archaea 1 (ANME-1), and *Archaeoglobi* (4, 14–16).

Pyrrolysine (Pyl), the 22nd genetically encoded amino acid, is charged to tRNA^Pyl^ by pyrrolysyl-tRNA synthetase (PylRS) (17, 18), which is specific for this unusual amino acid. PylRS is present in diverse bacteria and a few archaeal groups (19). PylRS is encoded by a single *pylS* gene in the *Methanosarcinaceae*, by the *pylSn* and *pylSc* gene, encoding the N- or C-terminal part, respectively, of PylRS in some anaerobic bacteria and “*Candidatus* Methanomethylicus sp. V1,” or by *pylSc* only in *Methanomassiliicoccales* (1, 19–21). The evolutionary pathways of the three types of PylRS remain unclear (19). Pyrrolysine biosynthesis genes (*pylBCD*), a tRNA^Pyl^ gene (*pylT*), and Pyl-utilizing methylamine methyltransferase genes (*mtxBC*) usually form a single gene cluster with the PylRS gene, which may have facilitated the horizontal gene transfer (HGT) of a Pyl-encoding system (22). The Pyl-utilizing methylamine:corrinoid methyltransferases (MtxB) transfer a methyl group from methylamines to their corrinoid protein partners (MtxC). The methyl group is then transferred to coenzyme M (CoM) in methanogens and possibly to CoM or tetrahydrofolate (THF) in bacteria (8, 23). Finally, the methyl group is released as methane by methyl-CoM reductase in methanogens and probably fuels anaerobic respiration in bacteria (8, 23).

In the last few years, analyses of genomic and metagenomic sequences have identified large numbers of novel bacterial and archaeal lineages. Some of these archaea are methanogens (21, 24–27). Most importantly, single-cell genomics and the composite-genome approach have dissected microbial dark matter (MDM) (28), the candidate phylum radiation (CPR) (29, 30), and the Asgard archaeal superphylum (31) by detecting and classifying uncultivated microbes (28, 29, 31–34). Progress in DNA sequence and *de novo* assembly technologies have led to the generation of larger genomic and metagenomic contigs encoding proteins. Phylogenetic studies of organisms based on protein sequences challenge traditional phylogenies based solely on rRNA sequences (29).

In this study, we assumed that the SepRS-SepCysS-SepCysE system might exist in diverse organisms whose genomic sequences were not available several years ago. We addressed (i) the distribution of the genes for SepRS, SepCysS, and SepCysE homologs outside the major *Euryarchaeota* groups and (ii) any relationships between RNA-dependent Cys biosynthesis and Sec- or Pyl-utilizing traits. In addition to investigating the genomic data, we investigated metagenomic protein sequence data, whose usage has been limited due to the low reliability of the data and the difficulty of inferring firm phylogenetic results. To overcome these problems, we performed a comprehensive survey of all metagenomic data sets in the IMG system (35) and at the NCBI rather than using an individual data set.

## RESULTS

### Identification of homologs of SepRS, SepCysS, and SepCysE.

In a preliminary search, we found SepRS genes in *Hadesarchaea* and MSBL1 (36, 37), “*Candidatus* Bathyarchaeota” (24, 38), and a few more groups of *Euryarchaeota* (26, 27) in the NCBI database. Furthermore, a SepRS-SepCysS operon was found in a metagenomic bin of a CPR bacterium, “*Candidatus* Parcubacteria” bacterium DG_74_2 (39). Although this “*Ca*. Parcubacteria” DG_74_2 bin is apparently composed of a few different genomes, including those of two “*Ca*. Parcubacteria” species, the SepRS-SepCysS operon is flanked by a typical “*Ca*. Parcubacteria” gene encoding a signal transduction histidine kinase. Thus, it is suggested that SepRS is not limited to the archaeal domain of life. Because the “*Ca*. Parcubacteria” DG_74_2 SepRS sequence (GenBank accession no. KPJ56532) differs from the methanogen SepRS sequences (about 40% similarity), we used it as query for the first round of genomic and metagenomic BLASTp searches. SepRS sequences that showed more than 40% similarity with the query were collected and grouped by similarity using Clustal X (40). Representative sequences of each group were subsequently used as queries for another run of metagenomic BLASTp to identify close relatives.

Corresponding/paired SepCysS genes are readily available in genomic sequences and metagenomic contig sequences, in which they exist in the vicinity of the SepRS gene. However, to identify the SepCysS genes paired with SepRSs obtained from different metagenomic contigs, the metagenomic contigs were binned based on GC contents and read depths. For precise phylogenetic inference, (i) some raw sequence data were used to connect neighboring contigs (41), (ii) binning of a single-cell genome and metagenomic contigs was performed in cases where both the cell and the DNA samples derived from the same sampling point, and (iii) rRNA and protein sequences were identified whenever possible. Binning was facilitated by an observation that similar organisms have similar SepRS and SepCysS genes and thrive in similar environments. In our analysis, we were able to pair most of the representative SepRS genes with one or two SepCysS genes.

### (i) Occurrence of SepRS.

**FIG 1:**
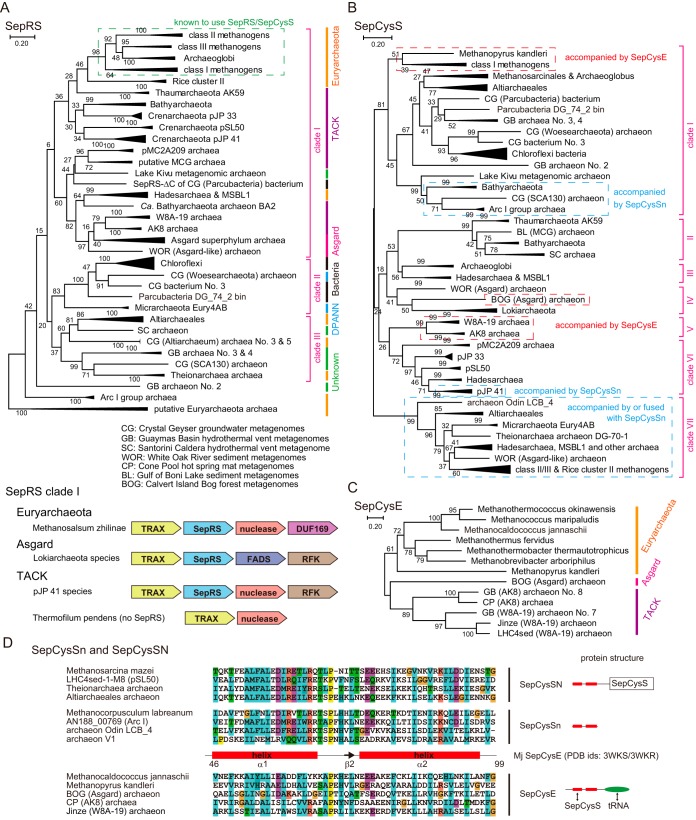
Distribution of SepRS, SepCysS, and SepCysE homologs in the prokaryotic domains of life. The bootstrap values (percentages) are shown for the unrooted maximum likelihood trees made with 100 replicates using MEGA 7. (A) Distribution of SepRS in archaea and bacteria. The archaeal species are (i) Rice cluster II or “*Ca.* Methanoflorentaceae” archaea (26); (ii) *Thaumarchaeota* AK59 archaea similar to clone AK59 locus tag AY555832 (all accession numbers are from GenBank unless otherwise specified) and clone 24Earc79 locus tag JN605031 (48, 82); (iii) “*Ca.* Bathyarchaeota” archaea (24, 38); (iv) three subgroups of uncultured *Crenarchaeota* (83) each similar to pSL50 gene U63342, pJP 33 gene L25300, and pJP 41 gene L25301, probably including “*Ca.* Verstraetearchaeota” archaea (21); (v) pMC2A209 archaea (49) similar to clone IAN1-71 locus tag AB175574 and clone ARC_OTU_72 locus tag KP091046 (47, 84); (vi) *Hadesarchaea* and MSBL1 archaea (36, 37); (vii) clone AK8/W8A-19 archaea similar to clone W8A-19 locus tag KM221272 (46) and clone AK8 locus tag AY555814 (48) or clone ARC_OTU_92 locus tag KP091068 (47); (viii) archaeon Odin LCB_4, “*Ca.* Lokiarchaeota” archaea, and a few unknown species in the Asgard superphylum (9, 31, 33); (ix) a Crystal Geyser groundwater (“*Ca.* Woesearchaeota”) archaeon most similar to archaeon GW2011_AR9 (85); (x) “*Ca.* Micrarchaeota” (Eury4AB group) archaea most similar to clone C1AA1CA10 locus tag GU127467 (86, 87); (xi) “*Ca.* Altiarchaeales” archaea (88–91); (xii) Crystal Geyser groundwater archaea (91), one of which is similar to clone SCA130 locus tag EU735580 (92); (xiii) locus tag Z7ME43 or *Theionarchaea* archaea (93); (xiv) Arc 1 group or “*Ca.* Methanofastidiosa” archaea (27); and (xv) unknown groups of archaea. The SepRS-harboring bacterial species are the “*Ca*. Parcubacteria” DG_74_2 bin bacterium (39), a putative deltaproteobacterium (CG bacterium no. 3), and *Chloroflexi* (probably *Dehalococcoides*) bacteria (34). SepRS sequences were classified into three clades and a few orphans. A few representative genetic loci of clade I SepRS genes are shown below the tree. As indicated, modern *Crenarchaeota*, including *Thermofilum pendens*, lack SepRS. TRAX belongs to translin superfamily proteins. FADS and RFK denote FAD synthase and riboflavin kinase, respectively. (B) Distribution of SepCysS in the prokaryotic domains of life. SepCysS sequences were classified into seven clades. (C) Distribution of SepCysE in three groups of selenocysteine-encoding archaea. (D) Multiple-alignment analysis of SepCysE homologs based on the crystal structure of *M. jannaschii* SepCysE (PDB accession no. 3wkr). The SepCysSn peptide is either encoded as a split gene preceding the SepCysS gene or N-terminally fused with SepCysS (SepCysSN).

Our analysis shows that SepRS is widespread among uncultured archaea and bacteria (Fig. 1A and see Fig. S1 in the supplemental material). SepRS is present in four clades of archaea (*Euryarchaeota*, DPANN, TACK, and Asgard) and in *Chloroflexi* and a few other bacterial species (Fig. 1A; Fig. S1). As an exception, a truncated SepRS gene that lacks the C-terminal anticodon binding domain (SepRS-ΔC) exists in an uncultured Crystal Geyser groundwater (“*Ca*. Parcubacteria”) bacterium (Fig. 1A). SepRS is common in some lineages of archaea (*Euryarchaeota* methanogens and *Archaeoglobi*, *Hadesarchaea*/MSBL1, “*Candidatus* Altiarchaeales,” *Crenarchaeota* pJP 33/pSL50/pJP 41, and “*Ca.* Bathyarchaeota”), while it is sparsely distributed or appears to be absent in others. Within the same SepRS subgroup, SepRS phylogeny tends to show lineage specificity (Fig. 1A) (see reference 4 for the case of methanogens), indicating coevolution with the host organism for various time periods.

10.1128/mBio.00561-17.3 FIG S1  Full trees for SepRS and SepCysS. Download FIG S1, PDF file, 0.1 MB. Copyright © 2017 Mukai et al. 2017 Mukai et al. This content is distributed under the terms of the Creative Commons Attribution 4.0 International license.

SepRS genes form three major clades (Fig. 1A). SepRS clade I is the largest and probably arose from a common ancestral operon containing the gene encoding an archaeal translin-associated protein X (TRAX) homolog (42) (Fig. 1A; Fig. S2). Assuming that the recently published (29) phylogenetic tree of life is mostly true, SepRS clade I represents relatively modern lineages of *Euryarchaeota* and the TACK and Asgard superphyla, whereas SepRS clades II and III represent more-ancient lineages of archaea (DPANN, “*Ca.* Altiarchaeales,” and Z7ME43/*Theionarchaea*) as well as bacteria. However, the evolutionary relationship of the three SepRS clades and the archaeal lineages remains unclear, notably because of an HGT event of SepRS in “*Ca.* Bathyarchaeota” and rapid evolution of SepRS in putative *Euryarchaeota* archaea (Fig. 1A). A late-branching “*Ca.* Bathyarchaeota” archaeon, BA2 (38), has a *Hadesarchaea*-type SepRS, which is different from other “*Ca.* Bathyarchaeota” SepRSs (Fig. 1A). The latter represent the most-diverged SepRS (or SepRS-like) genes, which were not paired with SepCysS genes by our contig binning. They may belong to the rapidly evolving groups of *Euryarchaeota* (Fig. 1A) (27, 43), which explains the extent of divergence.

10.1128/mBio.00561-17.4 FIG S2  Genetic loci of SepRS and SepCysS genes and bacterial tRNA^Cys^ genes. Bacterial tRNA^Cys^ species are shown with residue 37 marked with a red circle. Download FIG S2, PDF file, 0.5 MB. Copyright © 2017 Mukai et al. 2017 Mukai et al. This content is distributed under the terms of the Creative Commons Attribution 4.0 International license.

### (ii) Occurrence of SepCysS.

Classification of the collected SepCysS sequences revealed that, in addition to the three known SepCysS clades (here, clades I, III, and VII) (10), four other clades exist (here, clades II, IV, V, and VI) (Fig. 1B; Fig. S1). Residues critical for the Sep-to-Cys conversion are conserved in all SepCysS species, with only two exceptions. While residues involved in pyridoxal-phosphate (PLP) binding are conserved in all collected SepCysSs, the Cys residues involved in persulfide formation (6) are missing in these two cases, thereby suggesting that these SepCysS proteins might employ a different mechanism for sulfur transfer (see below).

SepCysS phylogeny shows a very low correlation with the SepRS phylogeny. There are two plausible explanations for it. (i) Some archaea have two copies of SepCysS genes (of the same clade or different clades) that are shared within the same subgroup of archaea (Fig. 1B; Fig. S1 and S2) (10, 44). Likewise, it is possible that a second SepCysS gene copy was excluded from our analysis due to incomplete genome sequencing and contig binning. Importantly, in our genome and metagenome analyses, no additional copies of SepRS genes were identified, nor were any SepCysS genes found in the complete genomes lacking SepRS. (ii) Because SepCysS shows less tRNA specificity than SepRS (45), SepCysS genes may be more prone to HGT than SepRS genes. The occurrence of a SepCysS gene duplication in some *Euryarchaeota* methanogens (Fig. S1) implies that an additional gene copy may enhance RNA-dependent cysteine biosynthesis under certain conditions.

### (iii) Occurrence of SepCysE homologs.

SepCysE genes are present in a few selenocysteine-encoding archaea other than class I methanogens (Fig. 1C; Fig. S3). SepCysE is present either in an operon with SepCysS in AK8/W8A-19 group archaea (46–49) or separately in a BOG (Asgard) archaeon (Fig. 1B and C; Fig. S2 and S3). In the archaeal domain, the Sec utilization trait was found within the *Euryarchaeota* class I methanogens (8) and two Asgard superphylum members (31) (“*Candidatus* Lokiarchaeota” [9] and *Thorarchaeota* [50]). The AK8/W8A-19 group archaea and the BOG (Asgard) archaeon share four selenoproteins (SPS, HdrA, VhuD, and VhuU) with *Methanopyrus*, *Methanococcus*, and “*Ca.* Lokiarchaeota” (9), whereas the AK8/W8A-19 SPS proteins are split in two fragments (Fig. S3). Our findings lend support to the hypotheses that the archaeal Sec-encoding system and the SepRS-SepCysS-SepCysE system emerged prior to the divergence of class I methanogens and “*Ca.* Lokiarchaeota” (9) and prior to the divergence of class I, II, and III methanogens (4), respectively. However, the possibility of HGT events after the division of these archaeal groups cannot be excluded.

10.1128/mBio.00561-17.5 FIG S3  Cooccurrence of SepRS-SepCysS-SepCysE and Sec-encoding systems in the clone AK8 W8A-19 group archaea and the BOG (Asgard) archaeon. See Table S1 for other members. Download FIG S3, PDF file, 0.4 MB. Copyright © 2017 Mukai et al. 2017 Mukai et al. This content is distributed under the terms of the Creative Commons Attribution 4.0 International license.

10.1128/mBio.00561-17.9 TABLE S1  Binning and naming of genomic and metagenomic contigs. Download TABLE S1, DOCX file, 0.03 MB. Copyright © 2017 Mukai et al. 2017 Mukai et al. This content is distributed under the terms of the Creative Commons Attribution 4.0 International license.

Some archaeal genomes contain homologs of the N-terminal helix-turn-helix domain of SepCysE (Fig. 1B and D). This homolog is present as an additional domain fused to SepCysS (some of the clade VII SepCysSs) or encoded as a split gene in front of clade SepCysS genes (clade VII SepCysSs and a few clade I and VI SepCysS genes) (Fig. 1B and D; Fig. S1). This SepCysE homolog was named “SepCysSn” when encoded by a separate gene or “SepCysSN” when fused to SepCysS (Fig. 1D).

### The genetic loci of SepRS and SepCysS.

The genetic loci and genes accompanying SepRS and SepCysS genes support the protein sequence-based phylogenies (Fig. S2). (i) Bacteria (and an archaeon) share the SepRS-SepCysS operon. In two cases, bacterial tRNA^Cys^ is found in an operon with either SepRS-ΔC or the second copy of SepCysS (Fig. S2). (ii) “*Ca*. Bathyarchaeota” archaeon BA2 has a compact operon encoding tRNA^Cys^, SepCysSn, SepCysS, and SepRS. This operon is widespread among marine sediment archaea, possibly because it is so amenable to HGT. (iii) Clade VII SepCysS genes are often associated with a tRNA^Cys^ gene, a few sulfur metabolism genes, and a small gene which was annotated to encode tRNA-Thr-editing domain (ED). As shown in Fig. S4, tRNA-Thr-ED is a homolog of the editing domain of archaeal threonyl-tRNA synthetase (ThrRS-R) (51) and the editing domain of archaeal transediting ThrRS-ED protein (52) (see Text S1 in the supplemental material). The tRNA-Thr-ED proteins of *Euryarchaeota* methanogens form a clade distinct from those of MSBL1/*Hadesarchaea* (Fig. S4B and C). The SepCysS proteins associated with these tRNA-Thr-ED proteins are distributed in the same manner (methanogens’ clade and MSBL1/*Hadesarchaea* clade) in our clade VII SepCysS phylogeny (Fig. 1B).

10.1128/mBio.00561-17.1 TEXT S1  Supplemental text about tRNA-Thr-ED proteins. Download TEXT S1, DOCX file, 0.01 MB. Copyright © 2017 Mukai et al. 2017 Mukai et al. This content is distributed under the terms of the Creative Commons Attribution 4.0 International license.

10.1128/mBio.00561-17.6 FIG S4  Identification and analysis of homologs of the editing domain of archaeal ThrRS. (A) Crystal structure of the editing domain of ThrRS from *M. jannaschii* with l-Ser3AA (a substrate analog) (PDB accession no. 4RRF). (B) Phylogeny of tRNA-Thr-ED homologs and the editing domains of archaeal ThrRS (ThrRS-R) and ThrRS-ED. The bootstrap values (percentages) are shown for the unrooted maximum likelihood tree made with 100 replicates using MEGA 7. The tRNA-Thr-ED proteins associating with clade VII SepCysS (shown in the red box) are very similar to the archaeal ThrRS (ThrRS-R) and ThrRS-ED proteins. d-Tyrosyl-tRNA^Tyr^ deacylase (DTD), used as an outgroup, was previously known as the closest homolog of the editing domain of ThrRS-R/ThrRS-ED. The full tree is shown below the compressed tree. (C) The SepCysS-associating tRNA-Thr-ED proteins from class II/III methanogens and rice cluster II methanogens have an additional C-terminal cysteine-rich motif, while *Hadesarchaea* MSBL1 proteins have no extension. Download FIG S4, PDF file, 0.3 MB. Copyright © 2017 Mukai et al. 2017 Mukai et al. This content is distributed under the terms of the Creative Commons Attribution 4.0 International license.

### Idiosyncrasies of bacterial SepRS and SepCysS.

**FIG 2:**
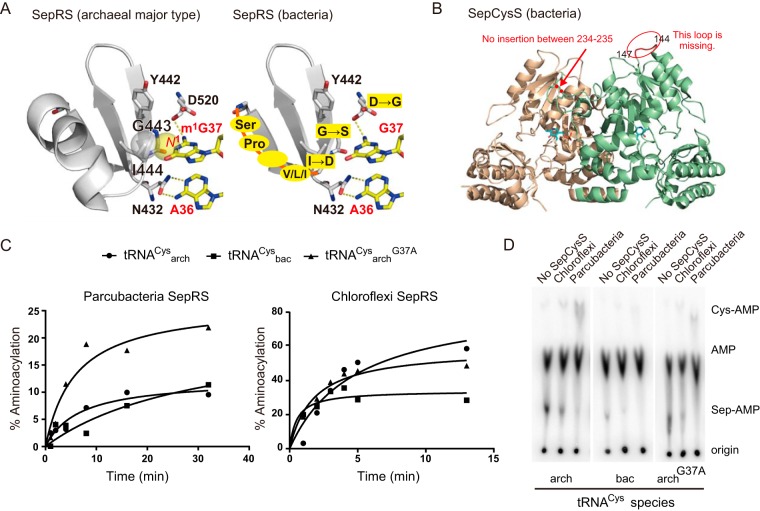
Bacterial SepRS and SepCysS and “*Ca*. Parcubacteria” tRNA^Cys^ species. (A) Modeling of the N37-recognizing motif of SepRS. The crystal structure of *A. fulgidus* SepRS⋅tRNA^Cys^ (PDB accession no. 2du3) was used for the modeling. Although G37 in the crystal structure is unmodified, *N*^1^-methylation may create a van der Waals interaction between the methyl group and the side chain of Ile444, as indicated with spheres. In bacterial SepRS species, Ile444 is replaced by hydrophilic Asp in most cases, and the following helix is totally missing. (B) Modeling of the dimer structure of SepCysS. The crystal structure of *A. fulgidus* SepCysS1 (PDB accession no. 2e7j) was used for the modeling. In bacterial SepCysS species, a loop (amino acids 144 to 147) is missing, and there is no insertion between amino acids 234 and 235. (C) Bacterial SepRS activity *in vitro*. Time course for plateau aminoacylation obtained by monitoring the accumulation of phosphoseryl-[^32^P]tRNA^Cys^ using thin-layer chromatograms in Fig. S5A. tRNA^Cys^ substrates from the “*Ca*. Parcubacteria” DG_74_2 bin are indicated (G37 containing tRNA^Cys^, [tRNA^Cys^_arch_], as a circle, its variant [tRNA^Cys^_arch_^G37A^] as a triangle, and “*Ca*. Parcubacteria”-type tRNA^Cys^ as rectangles [tRNA^Cys^_bac_]). (D) Thin-layer chromatograms showing SepCysS-dependent *O*-phosphoseryl- to cysteinyl-[^32^P]tRNA^Cys^ conversion. Reaction mixtures contained either “*Ca*. Parcubacteria” or *Chloroflexi* SepCysS (marked above the chromatogram). As a control, reaction mixtures containing only SepRS were also inspected (denoted “No SepCysS”). tRNA substrates are indicated below the chromatograms. Reaction products (*O*-phosphoseryl- and cysteinyl-[^32^P]tRNA^Cys^) were monitored as *O*-phosphoseryl- and cysteinyl-adenylates (Sep-AMP and Cys-AMP, respectively) after P1 nuclease digestion.

Bacterial SepRSs are highly distant from the well-studied methanogen SepRS gene (Fig. 1A). Bacterial SepCysSs, on the other hand, have a close evolutionary relationship with methanogens’ SepCysSs (Fig. 1B). Therefore, bacterial SepRS and SepCysS genes may have different archaeal origins and formed an operon after the branching of class I and class II and III methanogens. It is apparent from the multiple alignments of SepRS sequences that bacterial SepRS lacks a small motif involved in archaeal tRNA^Cys^ recognition (53, 54) (Fig. 2A). As this motif binds methylated guanosine 37, an identity determinant in methanogen SepRS systems (53–55), it appears that the *N*^1^-methyl modification of G37 does not contribute to bacterial SepRS⋅tRNA^Cys^ recognition. This is consistent with the fact that bacteria lack methyltransferase Trm5, which catalyzes m^1^G37 formation in archaeal tRNA^Cys^ species (54, 56–58). Our structural models of bacterial SepRSs based on the *Archaeoglobus fulgidus* SepRS⋅tRNA^Cys^ (PDB accession no. 2du3) crystal structure (59) show that a hydrophilic residue (mostly Asp) replaces the hydrophobic Ile444 within the enzyme’s anticodon binding domain. In *A. fulgidus* and methanogen SepRSs, Ile444 might be involved in m^1^G37 recognition (Fig. 2A). In addition, the vicinal helix of Ile444 is replaced with a short loop in bacterial SepRSs (Fig. 2A).

Bacterial SepCysS has the same structure as the archaeal clade I SepCysSs (e.g., *A. fulgidus* SepCysS1 [PDB accession no. 2e7j] [44]). Bacterial SepCysS lacks a fragment corresponding to a loop of *A. fulgidus* SepCysS1 (residues 144 to 147) (Fig. 2B). Only the CG (“*Ca*. Parcubacteria”) bacterium SepCysS retains this loop. Like the other clade I SepCysS genes (except *Methanopyrus kandleri*), bacterial SepCysS has an 8-amino-acid deletion between residues 234 and 235 (Fig. 2B). Because clade II-to-VII SepCysS species (with two exceptions) and *M. kandleri* SepCysS have an 8-amino-acid insertion here, the bacterial SepRS-SepCysS operons form a new lineage of SepCysS.

### (i) Bacterial SepRSs aminoacylate bacterial type (A37) tRNA^Cys^ species.

We established the function of two bacterial SepRS proteins (from “*Ca*. Parcubacteria” and *Chloroflexi*) with two tRNA^Cys^ species (A37 or G37) found in the “*Ca*. Parcubacteria” DG_74_2 bin (Fig. S2). The tRNA^Cys^ with A37 is similar to other “*Ca*. Parcubacteria” tRNAs^Cys^ and is designated tRNA^Cys^_bac_, whereas the tRNA^Cys^ with G37, which is similar to some archaeal tRNAs^Cys^ species, is designated tRNA^Cys^_arch_. We also examined the artificial G37A variant of tRNA^Cys^_arch_. Recombinant “*Ca*. Parcubacteria” SepRS aminoacylated both tRNA^Cys^_bac_ and tRNA^Cys^_arch_, albeit at a low level (10%) (Fig. 2C; Fig. S5A). The aminoacylation plateau level of the variant tRNA^Cys^_arch_^G37A^ was about twice that of tRNA^Cys^_arch_, suggesting that G37 is not a determinant for “*Ca*. Parcubacteria” SepRS (55). Recombinant *Chloroflexi* SepRS acylated tRNA^Cys^_arch_ and tRNA^Cys^_arch_^G37A^ to 50 to 60%, while tRNA^Cys^_bac_ was charged less well (to 35%) (Fig. 2C; Fig. S5A). Interestingly, an archaeal SepRS from *Methanococcus maripaludis* was also able to aminoacylate all three tRNA^Cys^ types (Fig. S5B), indicating a more relaxed tRNA specificity than expected from previous studies (3, 53–55, 59).

10.1128/mBio.00561-17.7 FIG S5  *In vitro* and *in vivo* assays of bacterial SepRS, SepCysS, and tRNA^Cys^. (A) Bacterial SepRS activity *in vitro*. This is the original data for Fig. 2C. Thin-layer chromatograms showing phosphoseryl-[^32^P]tRNA^Cys^ accumulation during a reaction time course in the case of “*Ca*. Parcubacteria” (left) and *Chloroflexi* SepRS (right). tRNA substrates are indicated below and time points are indicated above the chromatograms. A reaction mixture without the enzyme was loaded prior (c) and after (d) nuclease P1 digestion. (B) Bacterial tRNA^Cys^ variants are substrates for *Methanococcus maripaludis* SepRS. Thin-layer chromatograms showing phosphoseryl-[^32^P]tRNA^Cys^ (Sep-AMP) accumulation after 20 min (marked as “No SepCysS”). The produced phosphoseryl-[^32^P]tRNA^Cys^ is a substrate for *Chloroflexi* SepCysS (denoted as “*Chloroflexi*” above the chromatogram). Only phosphoseryl-(Sep-AMP) and cysteinyl-[^32^P]adenylate (Cys-AMP) are monitored after nuclease P1 digestion of phosphoseryl- and cysteinyl-[^32^P]tRNA^Cys^, respectively. tRNA substrates are indicated below and SepCysS presence is indicated above the chromatograms. (C) Bacterial SepCysS activity in *E. coli*. *E. coli* JS1 (Δ*selA*) was transformed with pACYC-MjPSTK-EcselC, pBAD-CDF-fdhF, and either of the SepCysS-expressing plasmids and an empty vector. In JS1 cells, conversion of Sep-tRNA^Sec^ to Cys-tRNA^Sec^ by SepCysS leads to the expression of full-length formate dehydrogenase H (FDH_H_), which converts benzyl viologen into a purple dye. Only the edges of the cell spots on agar plates were stained, because Cys-containing FDH_H_ is far less active than the wild-type-Sec-containing FDH_H_. Supplementation of the growth medium with sodium selenite (final concentration, 1 μM) enhanced the FDH_H_ activity of the cells expressing the “*Ca*. Parcubacteria” SepCysS, possibly due to the formation of Sec-tRNA^Sec^. Download FIG S5, PDF file, 1.1 MB. Copyright © 2017 Mukai et al. 2017 Mukai et al. This content is distributed under the terms of the Creative Commons Attribution 4.0 International license.

### (ii) Bacterial SepCysSs catalyze the tRNA-dependent Sep-to-Cys conversion.

Although the exact mechanism of the SepCysS-catalyzed reaction has not yet been fully elucidated, the Sep-to-Cys conversion most likely proceeds through a PLP-dependent generation of a dehydroalanyl-tRNA^Cys^ intermediate, which is subsequently attacked by a persulfide group to form Cys-tRNA^Cys^ (60, 61). Both “*Ca*. Parcubacteria” and *Chloroflexi* SepCysS possess residues involved in PLP binding (59), while only *Chloroflexi* SepCysS harbors conserved Cys residues implicated in persulfide (60, 61) and Fe−S cluster formation (6). “*Ca*. Parcubacteria” SepCysS is one of the two exceptional SepCysSs that lacks 2 out of 3 conserved cysteines (see above).

Both bacterial SepCysS proteins were expressed in *Escherichia coli* and purified anaerobically. Consistently with the presence of an Fe−S cluster, samples containing purified *Chloroflexi* SepCysS displayed a brown color, while “*Ca*. Parcubacteria” SepCysS samples were colorless, in agreement with the lack of cysteines needed for an Fe−S cluster formation (6). To demonstrate SepCysS activity, reaction mixtures containing 5 µM *Chloroflexi* SepRS and either 40 µM *Chloroflexi* or 20 µM “*Ca*. Parcubacteria” SepCysS were performed. Like SepRS, both SepCysSs were shown to be functional in the cases of all three tRNA^Cys^ variants (Fig. 2D). While conversion was complete in the case of “*Ca*. Parcubacteria” SepCysS (95 to 99% of the total Sep-tRNA^Cys^ intermediate), *Chloroflexi* SepCysS converted ~30 to 60% of Sep-tRNA^Cys^ to Cys-tRNA^Cys^ (Fig. 2D). We also found using a previously reported method (45) that “*Ca*. Parcubacteria” and *Chloroflexi* SepCysS proteins are active in *E. coli* (Fig. S5C).

### Cooccurrence of PylRS and SepRS in some archaea and bacteria.

**FIG 3:**
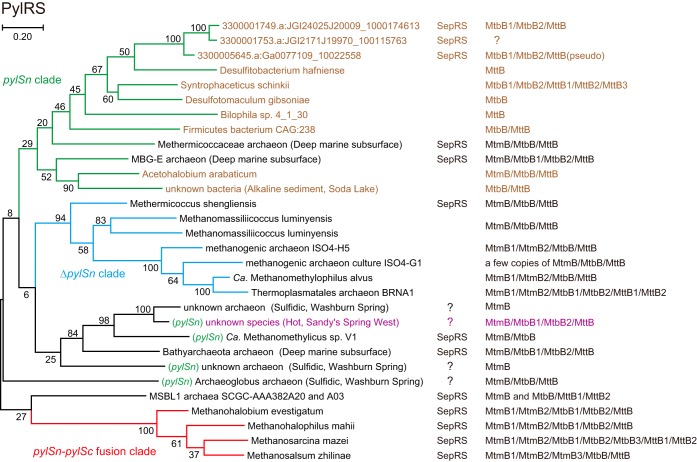
Distribution of PylRS in the prokaryotic domains of life. The bootstrap values (percentages) are shown for the unrooted maximum likelihood tree made with 100 replicates using MEGA 7. Bacterial origin is indicated with brown letters, while archaeal origin is indicated with black letters. Purple letters indicate a pending phylogenetic inference. Metagenomic origins are described in black parentheses, whereas “(*pylSn*)” indicates the existence of a *pylSn* gene. The occurrence of SepRS and Pyl-containing methyltransferases are shown next to the tree. For some of the archaeal bins in the hot spring metagenomes, the presence and absence of SepRS is pending, which is designated with “?.”

Diverse archaea and a group of *Chloroflexi* bacteria possess both SepRS and Pyl-encoding systems. Methane-producing *Methermicoccus shengliensis* strains AmaM and ZC-1 (62, 63) contain a *Methanomassiliicoccales*-type PylSc and tRNA^Pyl^ (Fig. 3). *M. shengliensis* uses trimethylamine for methanogenesis (62, 63), consistent with the presence of Pyl-containing mono-, di-, and trimethylamine methyltransferases (MtmB, MtbB, MttB) (Fig. 3) (23, 63). Two closely related MSBL1 archaea, SCGC-AAA382A20 and SCGC-AAA382A03 (37), have an incomplete or defective Pyl-encoding system and MtmB/MtbB/MttB genes. Very similar and complete Pyl-encoding operons of another halophilic archaeon have been reported (BioSample accession no. SAMN05770050). The V1 strain of “*Candidatus* Verstraetearchaeota,” or “*Ca*. Methanomethylicus” sp. strain V1, a newly proposed methanogen within the Terrestrial Miscellaneous Crenarchaeota group (TMCG), was reported to have PylSc, PylSn, and MtmB/MtbB (21), although tRNA^Pyl^ was not identified. We found the V1 to V5 strains of “*Ca.* Verstraetearchaeota” (21) to have SepRS of the pJP 41 SepRS group (Fig. 1A). Surprisingly, the PylRS species of the MSBL1 and “*Ca.* Verstraetearchaeota” archaea are not grouped within the three known clades of PylRS (Fig. 3).

In analyzing the metagenomic data sets, we focused on particular metagenomes because a whole Pyl-encoding gene cluster is rarely contained in a single metagenomic contig, which hampers the phylogenetic inference of PylRS genes. We chose data sets from deep marine and hot spring environments because of the abundance of archaeal species in these niches, the presence of SepRS genes, and high-quality data that are provided by Microbial Dark Matter, phase II.

Invaluable information was obtained from the metagenome of a deep-oceanic, basalt-hosted subsurface ecosystem from Juan de Fuca Ridge flank, Pacific Ocean (CORK borehole 1362A_J2.573). Three dominant archaeal species, *Methermicoccaceae*, marine benthic group E (MBG-E), and “*Ca.* Bathyarchaeota” (64), possess both SepRS and PylRS genes as well as the genes for Pyl-utilizing MtmB/MtbB/MttB enzymes (Fig. 3). The PylRS species of the *Methermicoccaceae* and MBG-E archaea divide the bacterial PylRS clade into two (*Acetohalobium* and others) (Fig. 3), indicating the occurrence of horizontal gene transfer of a Pyl-encoding system between bacteria and archaea (65). The “*Ca.* Bathyarchaeota” PylRS (PylSc) forms a new PylRS clade (Fig. 3) together with the V1 PylSc and some PylSc species found in the Deep Marine Sediments White Oak River (WOR) estuary metagenomes (data not shown). Pyl-encoding systems are also present in the hot spring metagenomes, although their metagenomic bins are less reliable due to the complex composition of the prokaryotic communities (Fig. 3). In the sulfidic Washburn Spring metagenome, one *Archaeoglobus*-type and several *Crenarchaeota*-type SepRS genes were also found. Thus, it is tempting to assume that a few subgroups of *Archaeoglobus* and TMCG possess both SepRS and PylRS.

The metagenome data revealed many *Chloroflexi*-type *pylSc* and *pylSn* genes (Fig. 3; Fig. S6A). Interestingly, a lineage of *Dehalococcoides* was found to have both SepRS and PylRS (Fig. 3). In one case, a SepRS-SepCysS operon and a *pylSn* gene exist on the same metagenomic contig (Fig. S6B).

10.1128/mBio.00561-17.8 FIG S6  Cooccurrence of SepRS and PylRS in bacteria. (A) Lineage of *Dehalococcoides* carrying SepRS, SepCysS, and PylRS; (B) binning of metagenomic contigs of the three *Dehalococcoides* strains with SepRS, SepCysS, and PylRS. Download FIG S6, PDF file, 0.01 MB. Copyright © 2017 Mukai et al. 2017 Mukai et al. This content is distributed under the terms of the Creative Commons Attribution 4.0 International license.

## DISCUSSION

In this study, we searched all the genomic and metagenomic protein sequence data in the public databases for the RNA-dependent cysteine biosynthesis pathway. Previous studies used only genome sequences and a particular metagenome sequence datum to search for a particular aminoacyl-tRNA synthesis system, in part due to the low reliability and accessibility of metagenomic sequence data. We encountered a similar problem with the “*Ca*. Parcubacteria” DG_74_2 bin, which is apparently composed of a few different genomes, including two “*Ca*. Parcubacteria” species. Our contig binning was greatly facilitated by the fact that minor genetic code systems rely on multiple components, which are, in turn, frequently dispersed on different metagenomic contigs. This approach eventually led us to the detection of rRNA and protein genes useful for phylogenetic inference. This work and other recent studies (66–68) will lead future studies of gene evolution in uncultured microbes.

Our phylogenetic analyses demonstrate that (i) the well-investigated class I/II/III methanogen and *Archaeoglobi* SepRSs constitute only a terminal branch of one of the clades, (ii) the TRAX-SepRS genes, SepCysE, the Sec-encoding system, and the four selenoproteins are shared by *Euryarchaeota* and TACK/Asgard, (iii) a few groups of proteins accompany SepCysS genes within the genetic loci, (iv) new PylRS types occur in nature and represent a missing link between the three known clades of PylRS, and (v) modern archaea may have fused the adapter peptides PylSn and SepCysSn to PylSc and SepCysS, respectively. In addition, our biochemical analyses confirm that bacterial SepRS and SepCysS species from uncultured “*Ca*. Parcubacteria” and *Chloroflexi* bacteria possess canonical activity. It is still not clear whether the SepRS system was present in the last common archaeal ancestor (3, 4), because the SepRS system was rarely found in the DPANN group, which was predicted (29) to have diverged first among archaea. It is also unclear why the SepRS system is absent or sparsely distributed in many branches of archaea. Was it gradually replaced by CysRS in each branch or horizontally transferred from another branch?

There may be diverse mechanisms for RNA-dependent cysteine biosynthesis in nature. The composite genome of a bacterium, ADurb.Bin236 (BioSample accession no. SAMN05004151), encodes a noncanonical SepRS homolog (GenBank accession no. OQA87054.1) and a SepCysSn-SepCysS operon (GenBank accession no. OQA83877.1 and OQA83876.1). Their protein sequences are highly diverged and may have archaeal origins. Surprisingly, this SepRS homolog has an additional N-terminal domain corresponding to the serine-editing domain of archaeal ThrRS (ThrRS-R). This is consistent with the genetic coupling of some clade VII SepCysS and tRNA-Thr-ED genes in *Euryarchaeota*. Although further study and validation are required, one may hypothesize that some SepRS species might possess a serine-editing activity in *cis* or in *trans*, because dephosphorylation of Sep-tRNA^Cys^ produces Ser-tRNA^Cys^, which may translate cysteine codons as serine.

The presence of the SepRS and SepCysS system may correlate with a high demand for iron-sulfur proteins, one of which is SepCysS (6), in obligate anaerobes for methanogenesis and for other metabolisms (14, 69, 70). For example, organohalide respiration in *Dehalococcoides* relies on iron-sulfur proteins (71). The coexisting tendency of PylRS and SepRS in archaea and bacteria may be partially explained by the facts that methylamine metabolism by Pyl-utilizing enzymes requires an iron-sulfur protein, RamA (8, 72), and that methylornithine synthase (PylB) is an iron-sulfur enzyme (73). Apart from assisting iron-sulfur proteins, the SepRS system may be useful for extreme thermophiles, because free phosphoserine is stable even at an extremely high temperature (74).

It has been shown that genes characteristic of methanogen-type sulfur assimilation and mobilization exist in some deltaproteobacteria and *Chloroflexi* (75). These genes encode proteins involved in methanogen-idiosyncratic homocysteine synthesis and facilitate growth when sulfide is provided as the sole sulfur source (5, 61, 75). As predicted, these genes cooccur with SepCysS and are present in SepRS-carrying *Chloroflexi Dehalococcoidia* bacterium CG2_30_46_9, *Dehalococcoidia* bacterium CG2_30_46_19, and *Chloroflexi* bacterium RBG_13_51_36 (see Fig. S1 in the supplemental material). Because of the vast abundance of *Chloroflexi* in deep sediments, their metabolic traits have a direct impact on sulfur cycling within the marine subsurface.

## MATERIALS AND METHODS

### Bioinformatics.

A BLAST search was performed by using three public Web servers, JGI IMG/MER (35), NCBI BLAST, and NCBI SRA BLAST. Some of the SepCysSn and tRNA sequences were manually identified. The SepRS sequences of “*Ca.* Verstraetearchaeota” were obtained by using tBLASTn from accession no. PRJNA321438. Multiple-alignment analyses of protein sequences were performed using Clustal X 2.1 (40), followed by manual curation based on the reported structure-based alignment analyses of SepRS (59), SepCysS (44), SepCysE (4), the ThrRS editing domain (76), and PylRS (77) using SeaView (78). The phylogeny reconstruction analyses of the alignment files were performed by using MEGA 7 (79) with the default settings (maximum likelihood, Jones-Taylor-Thornton [JTT] model, uniform rates, use all gaps/missing sites). Protein structure models were made with PyMol 1.7.6.0 (Schrödinger, LLC). The sequence and alignment data used in this study are provided in the supplemental material (see Data Set S1).

10.1128/mBio.00561-17.10 DATA SET S1  Sequence and alignment data. Download DATA SET S1, RTF file, 0.4 MB. Copyright © 2017 Mukai et al. 2017 Mukai et al. This content is distributed under the terms of the Creative Commons Attribution 4.0 International license.

Binning of metagenomic contigs was performed based on GC contents and read depths. Some of the WOR metagenomic contigs lack the read depth information. For the binning of metagenomic contigs of the AK8/W8A-19 group archaea, contaminating “*Ca.* Bathyarchaeota” contigs were removed. For the binning of metagenomic contigs of the BOG (Asgard) archaeon, each contig was confirmed to harbor Asgard-like protein genes, and contaminating *Methanocella* contigs were removed. The automatic annotation pipelines of the NCBI and JGI databases and our manual annotation/curation identified or predicted the host archaea and bacteria of these metagenomic contigs (Table S1). Our 16S rRNA phylogeny revealed that unclassified “LHC4-2-B” archaea JGI MDM2 LHC4sed-1-M8 and N8 belong to the pSL50 group and that unclassified “LHC4-2-B” archaeon JGI MDM2 LHC4sed-1-M18 belongs to the pJP 33 group. It was revealed that W8A-19 archaea, which were annotated to belong to the *Korarchaeota* (46), have ribosomal protein operons very similar to those of AK8 archaea (Fig. S3).

### *In vitro* and *in vivo* assays of bacterial SepRS and SepCysS.

Assays were performed using traditional methods (45, 80, 81). Detailed materials and methods are provided in Text S2 in the supplemental material.

10.1128/mBio.00561-17.2 TEXT S2  Supplemental materials and methods. Download TEXT S2, DOCX file, 0.2 MB. Copyright © 2017 Mukai et al. 2017 Mukai et al. This content is distributed under the terms of the Creative Commons Attribution 4.0 International license.
