# Supplementary figures and images for: RNA-Dependent Cysteine Biosynthesis in Bacteria and Archaea
Source: mBio. 2017 May 9;8(3):e00561-17. doi: 10.1128/mBio.00561-17 (PMC5424206; doi:10.1128/mBio.00561-17)

# SepRS

0.20

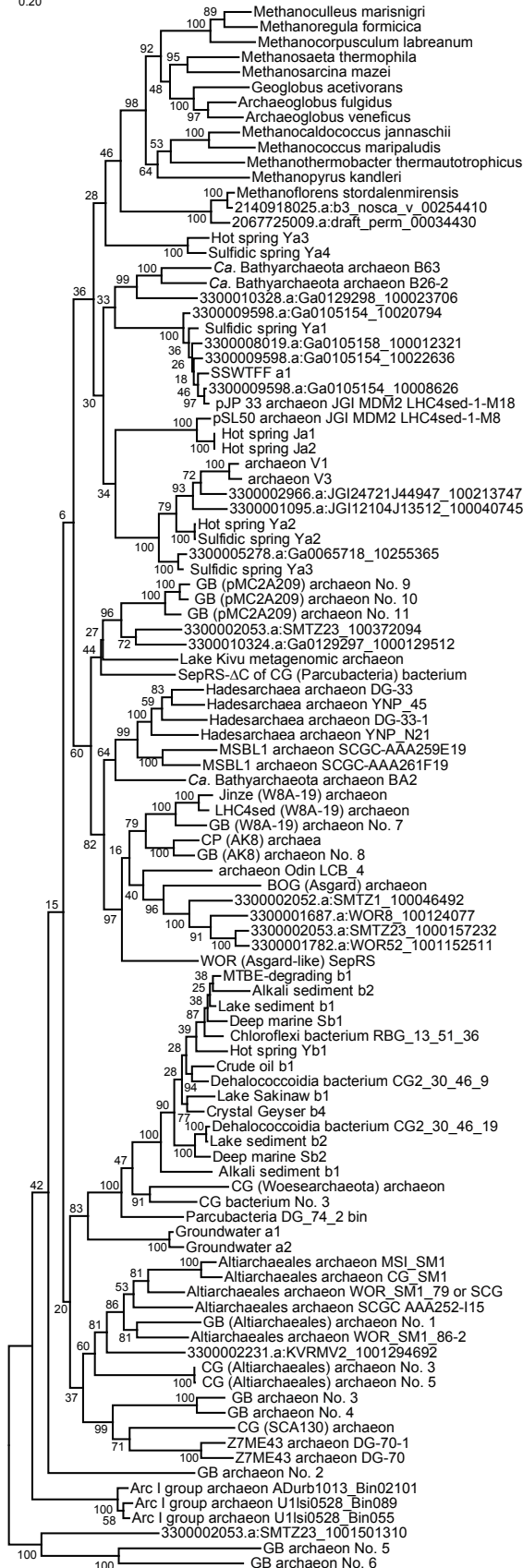

# SepCysS

0.20

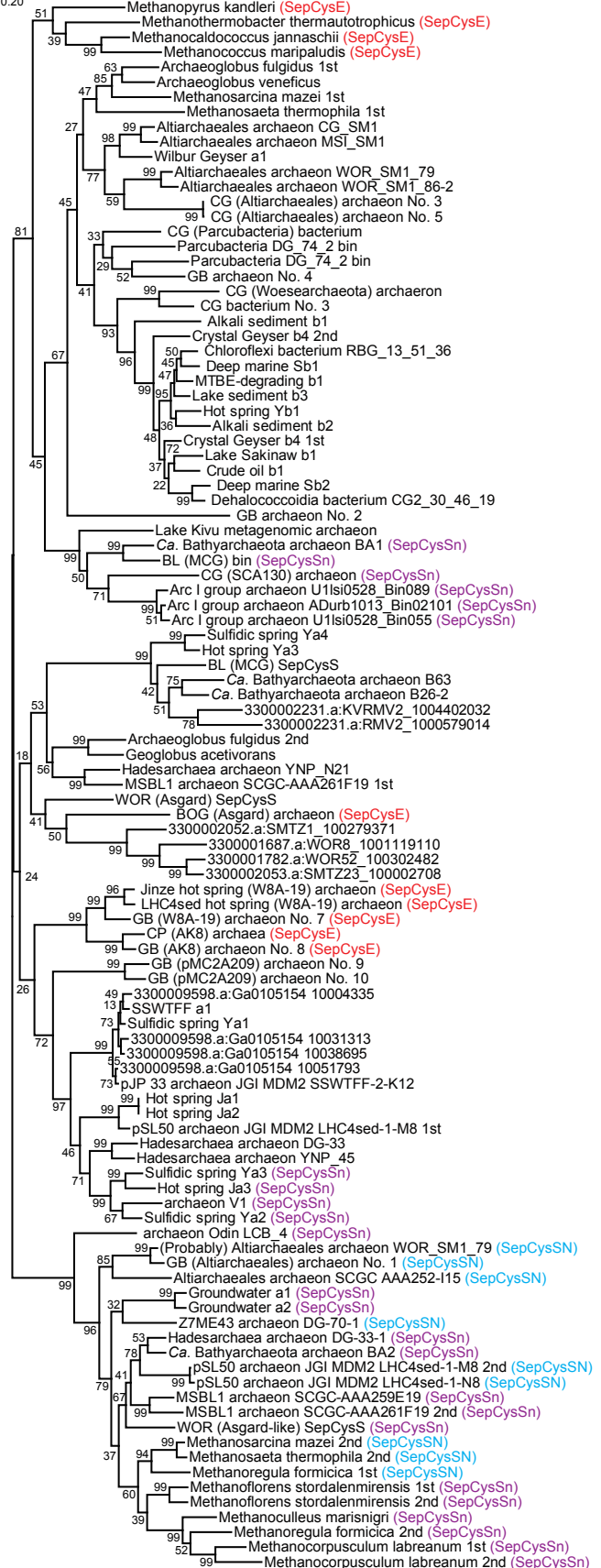

Supplement: FIG S1 [file mbo002173292sf1.pdf]

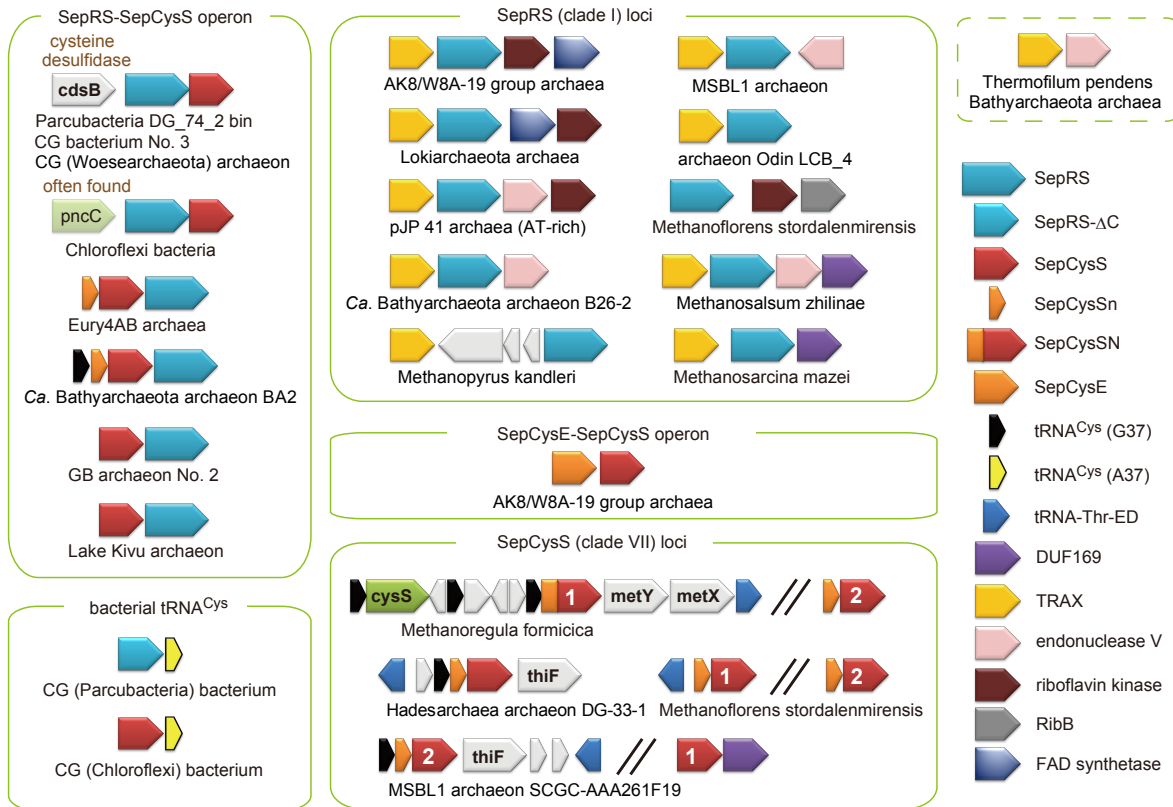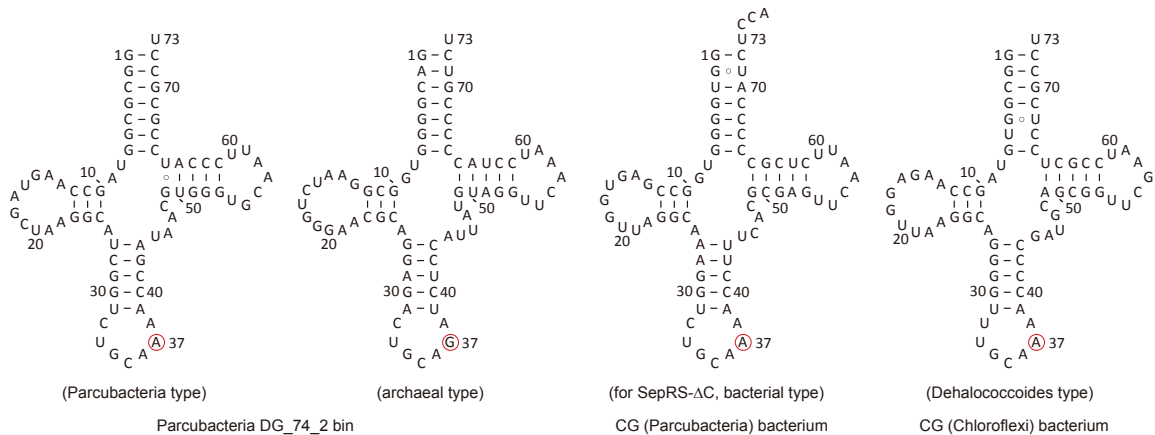

Supplement: FIG S2 [file mbo002173292sf2.pdf]

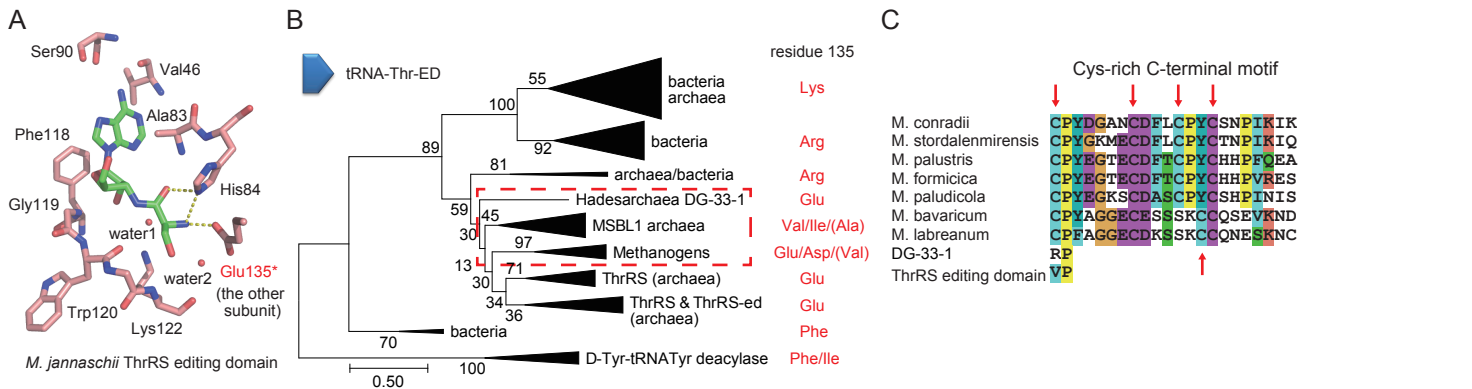

tRNA-Thr-ED, DTD, & the editing domains of ThrRS-R & ThrRS-ed

0.50

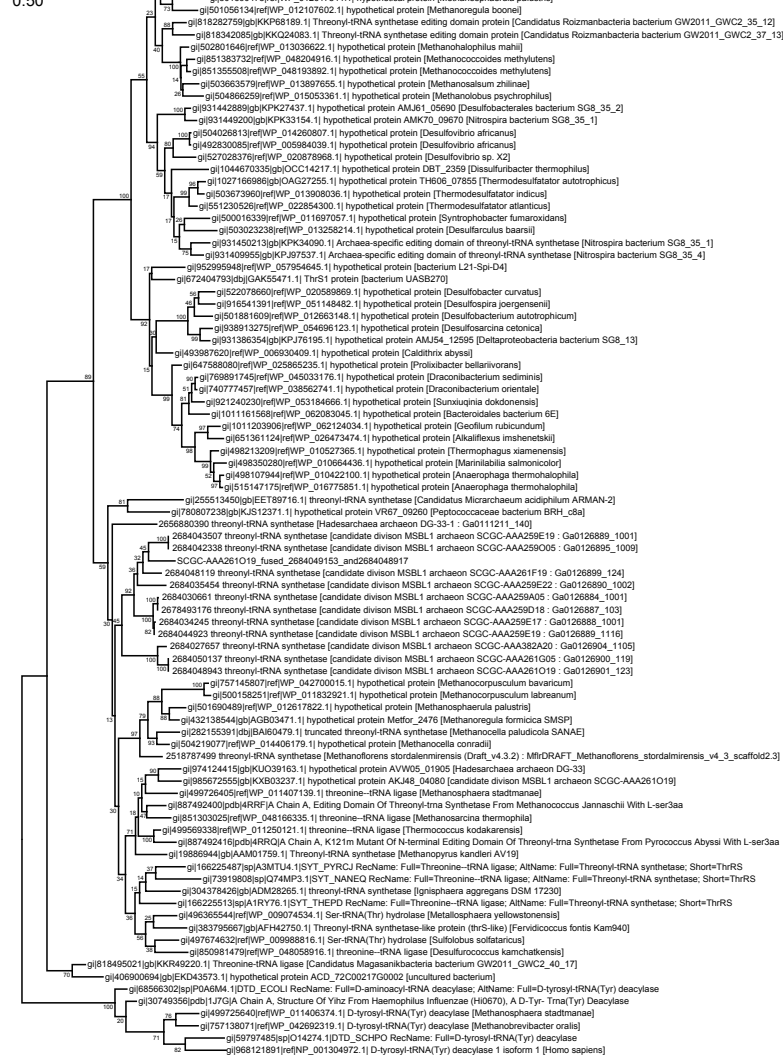

Supplement: FIG S4 [file mbo002173292sf4.pdf]

A

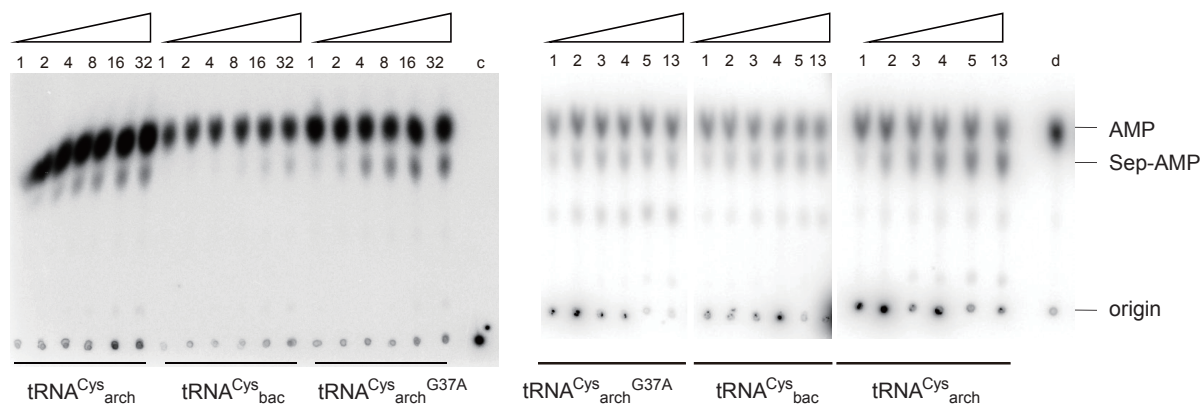

B

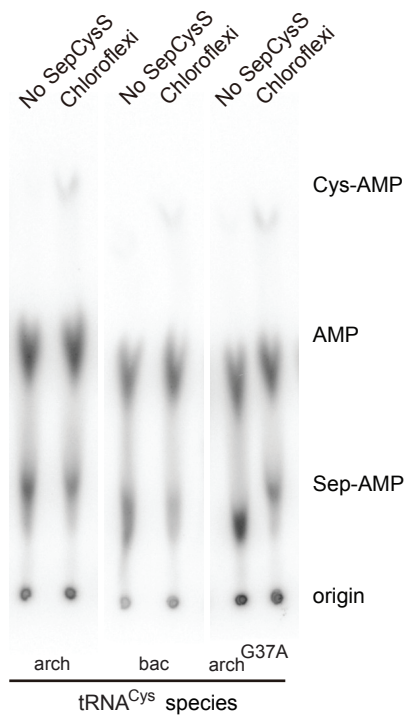

C

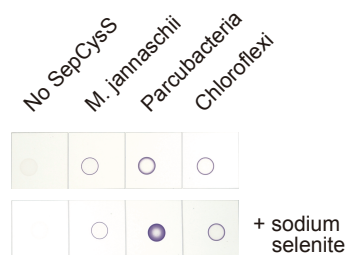

Supplement: FIG S5 [file mbo002173292sf5.pdf]
