# Supplementary material for: RNA-Dependent Cysteine Biosynthesis in Bacteria and Archaea
Source: mBio. 2017 May 9;8(3):e00561-17. doi: 10.1128/mBio.00561-17 (PMC5424206; doi:10.1128/mBio.00561-17)

Archaea having SepRS-SepCysS-SepCysE and SelBCD-PSTK-SepSecS

Cone Pool mat layer E metaG (CP (AK8) archaea)

3300010308.a:Ga0136652_1000925 (13206bp gc=0.44 depth=97)

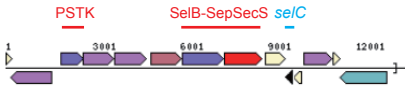

3300010308.a:Ga0136652_1000397 (21116bp gc=0.44 depth=99)

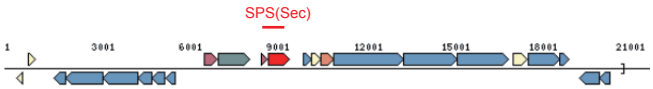

3300010308.a:Ga0136652_1000089 (50853bp gc=0.4 depth=108)

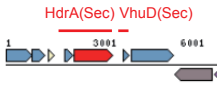

3300010308.a:Ga0136652_1000328 (23679bp gc=0.42 depth=97)

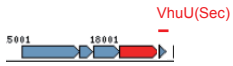

3300010308.a:Ga0136652_1000125 (42716bp gc=0.43 depth=93)

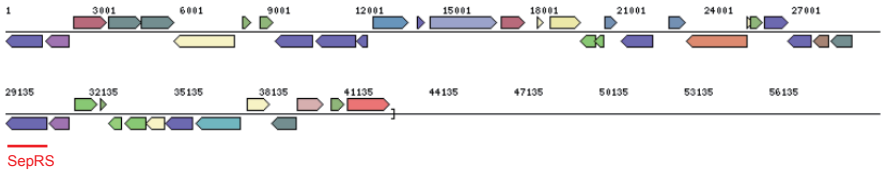

3300010308.a:Ga0136652_1005653 (3749bp gc=0.41 depth=92)

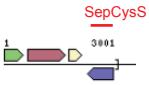

3300010308.a:Ga0136652_1003451 (5437bp gc=0.4 depth=76)

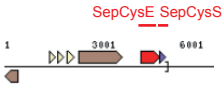

3300010308.a:Ga0136652_1000286 (25883bp gc=0.41 depth=94)

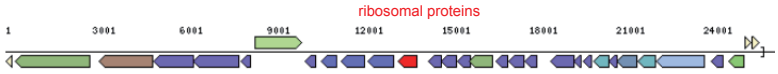

3300010308.a:Ga0136652_1000698 (15661bp gc=0.48 depth=112)

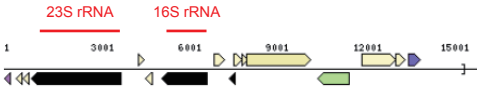

3300010308.a:Ga0136652_1000996 (12706bp gc=0.42 depth=106)

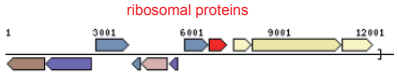

Cone Pool mat layer H metaG

SepSecS: 3300010284.a:Ga0129301_1039518 (1065bp gc=0.46 depth=9)
PSTK: 3300010284.a:Ga0129301_1045679 (962bp gc=0.42 depth=11)
SelD: 3300010284.a:Ga0129301_1037255 (1111bp gc=0.47 depth=17)
SepRS: 3300010284.a:Ga0129301_1050581 (897bp gc=0.43 depth=9)
SepRS: 3300010284.a:Ga0129301_1017419 (1906bp gc=0.4 depth=13)
SepCysS: 3300010284.a:Ga0129301_1037654 (1102bp gc=0.43 depth=10)
16S rRNA: 3300010284.a:Ga0129301_1015111 (2114bp gc=0.54 depth=22)

Cone Pool mat layer C metaG

SelB & SepSecS: 3300010289.a:Ga0129299_1020826 (3367bp gc=0.47 depth=16)
PSTK: 3300010289.a:Ga0129299_1010064 (5504bp gc=0.44 depth=15)
SelD: 3300010289.a:Ga0129299_1045395 (1838bp gc=0.44 depth=13)
SepRS: 3300010289.a:Ga0129299_1052619 (1629bp gc=0.42 depth=13)
SepCysS & SepCysE: 3300010289.a:Ga0129299_1008071 (6310bp gc=0.41 depth=16)
16S rRNA: 3300010289.a:Ga0129299_1006100 (7467bp gc=0.52 depth=26)

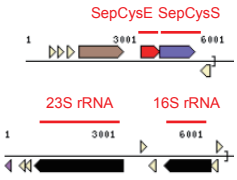

LHC4sed (W8A-19) archaeon

3300008019.a:Ga0105158_1000434 (18213bp gc=0.51 depth=10)

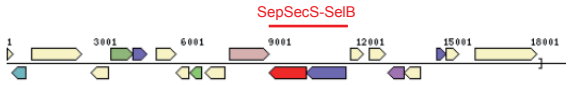

3300008019.a:Ga0105158_1000225 (25547bp gc=0.51 depth=9)

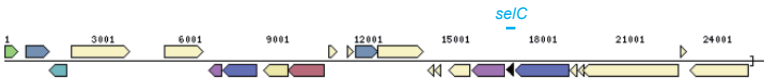

3300008019.a:Ga0105158_1000077 (44819bp gc=0.49 depth=10)

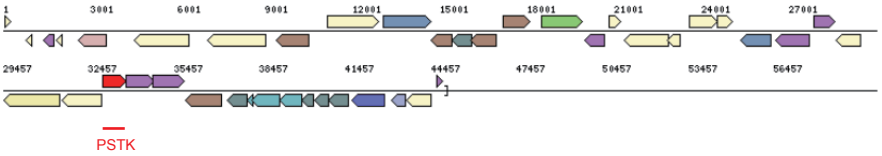

3300008019.a:Ga0105158_1000017 (71907bp gc=0.47 depth=10)

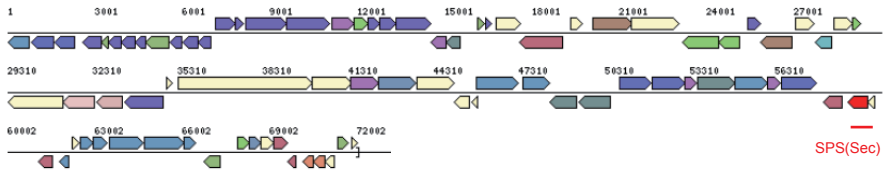

3300008019.a:Ga0105158_1000003 (103783bp gc=0.44 depth=10)

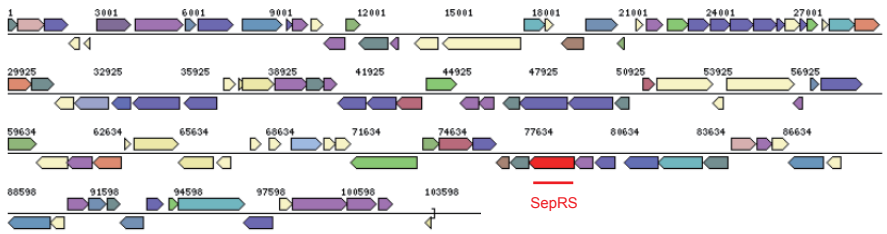

3300008019.a:Ga0105158_1000011 (82209bp gc=0.46 depth=10)

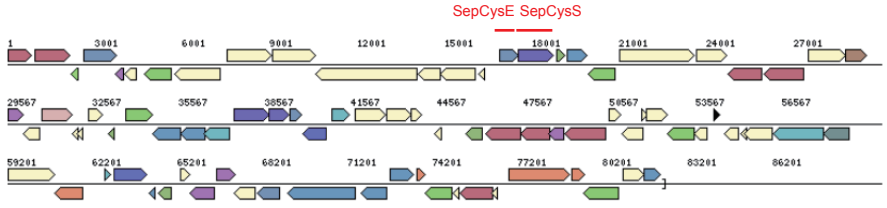

3300008019.a:Ga0105158_1000055 (51471bp gc=0.49 depth=10)

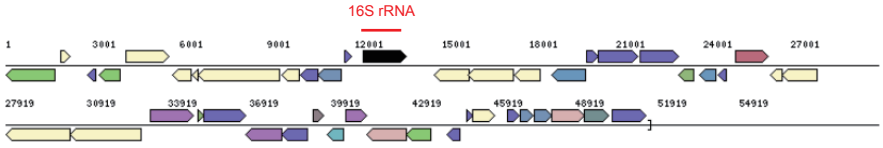

BOG (Asgard) archaeon

3300003218.a:JGI26339J46600_10000823 (10266bp gc=0.47 depth=21)

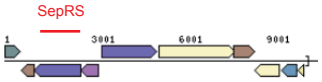

3300003218.a:JGI26339J46600_10134722 (583bp gc=0.54 depth=14)

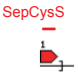

3300003218.a:JGI26339J46600_10001817 (6631bp gc=0.45 depth=24)

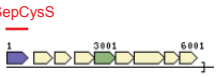

3300003218.a:JGI26339J46600_10002441 (5642bp gc=0.47 depth=11)

3300003218.a:JGI26339J46600_10022902 (1805bp gc=0.49 depth=22)

3300003218.a:JGI26339J46600_10000844 (10066bp gc=0.48 depth=16)

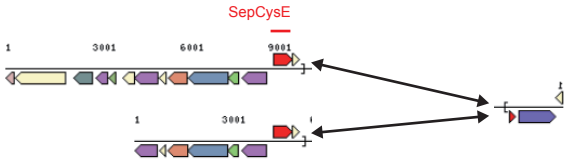

3300003218.a:JGI26339J46600_10002384 (5718bp gc=0.49 depth=21)

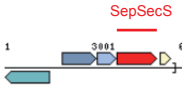

3300003218.a:JGI26339J46600_10001324 (7927bp gc=0.48 depth=21)

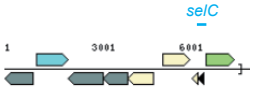

3300003218.a:JGI26339J46600_10004459 (4136bp gc=0.47 depth=20)

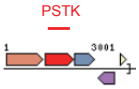

3300003218.a:JGI26339J46600_10012099 (2520bp gc=0.47 depth=15)

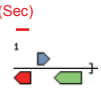

3300003218.a:JGI26339J46600_10003870 (4447bp gc=0.52 depth=18)

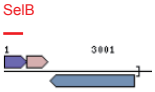

3300003218.a:JGI26339J46600_10003240 (4890bp gc=0.48 depth=22)

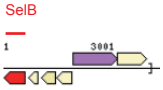

3300003218.a:JGI26339J46600_10000997 (9218bp gc=0.45 depth=20)

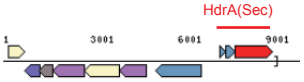

3300003218.a:JGI26339J46600_10001173 (8485bp gc=0.44 depth=21)

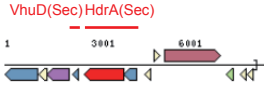

3300003218.a:JGI26339J46600_10001041 (8978bp gc=0.5 depth=20)

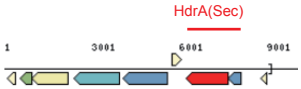

3300003218.a:JGI26339J46600_10002996 (5080bp gc=0.46 depth=23)

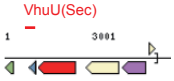

Supplement: FIG S3 [file mbo002173292sf3.pdf]
