# Supplementary material for: RNA-Dependent Cysteine Biosynthesis in Bacteria and Archaea
Source: mBio. 2017 May 9;8(3):e00561-17. doi: 10.1128/mBio.00561-17 (PMC5424206; doi:10.1128/mBio.00561-17)
Supplement: TABLE S1 [file mbo002173292st1.docx]

**Table S1.** Binning and naming of genomic and metagenomic contigs.

| name of bin | 16S rRNA or conserved proteins | most similar to | contigs |
| --- | --- | --- | --- |
| CG (Woesearchaeota) archaeon |  |  | 3300004105.a:Ga0065182_1000623 |
|  |  |  | 3300004106.a:Ga0065180_10158585 |
|  |  |  | 3300004105.a:Ga0065182_1013619 |
|  |  |  | 3300004106.a:Ga0065180_10058363 |
|  | Ga0065182_10001472 (L11) | KHO49120 | 3300004105.a:Ga0065182_1000147 |
|  |  |  | 3300004106.a:Ga0065180_10237606 |
|  | Ga0065182_10063122 (eIF2α) | KHO50122 | 3300004105.a:Ga0065182_1006312 |
|  |  |  | 3300004108.a:Ga0065181_1454869 |
|  | Ga0065182_100017621 (ValRS) | KHO50090 | 3300004105.a:Ga0065182_1000176 |
|  |  |  | 3300004108.a:Ga0065181_1184189 |
|  | Ga0051980_104128371 (CysRS) | KHO50087 | 3300003830.a:Ga0051980_10412837 |
|  |  |  | 3300004105.a:Ga0065182_1003172 |
| Altiarchaeales archaeon CG_SM1 |  |  | 3300003830.a:Ga0051980_10036067 |
| (CG2_30_SUB100_Altiarchaeum_32_3053) |  |  | 3300003830.a:Ga0051980_10011024 |
|  | Ga0051980_100844191 | KU868082 | 3300003830.a:Ga0051980_10084419 |
| CG (Altiarchaeales) archaeon No. 3 | Ga0065182_10320161 | 2658568218 | 3300004105.a:Ga0065182_1032016 |
|  |  |  | 3300004105.a:Ga0065182_1028247 |
|  |  |  | 3300004105.a:Ga0065182_10180171 |
|  |  |  | 3300003830.a:Ga0051980_10044286 |
|  | Ga0065182_12279731 | AUK59_06545 | 3300004105.a:Ga0065182_1227973 |
|  |  |  | gnl\|SRA\|SRR1534387.232758667.1  gnl\|SRA\|SRR1534387.232758667.2 |
|  | Ga0065182_10180012 | OIQ04580 | 3300004105.a:Ga0065182_1018001 |
| CG (SCA130) archaeon | Ga0066637_100137931 | EU735580 | 3300005235.a:Ga0066637_10013793 |
|  |  |  | 3300005236.a:Ga0066636_10019643 |
|  |  |  | 3300005235.a:Ga0066637_10000520 |
|  |  |  | 3300005235.a:Ga0066637_10000072 |
| CG (Altiarchaeales) archaeon No. 5 | Ga0100401_10102701 | AUK59_06545 | 3300007965.a:Ga0100401_10102707 |
| Wilbur Geyser a1 |  |  | 3300003902.a:JGI26788J51865_103265 |
|  |  |  | 3300003902.a:JGI26788J51865_105659 |
| Groundwater a1 |  |  | 3300005326.a:Ga0074195_1000198 |
|  |  |  | 3300005326.a:Ga0074195_1000053 |
|  | Ga0074195_10108343 | GU127467 | 3300005326.a:Ga0074195_1010834 |
|  |  |  | 3300005326.a:Ga0074195_1000056 |
|  |  |  | 3300005326.a:Ga0074195_1000110 |
|  |  |  | 3300005326.a:Ga0074195_1306236 |
|  |  |  | 3300005326.a:Ga0074195_1000604 |
|  |  |  | 3300005326.a:Ga0074195_1037187 |
|  |  |  | 3300005326.a:Ga0074195_1000103 |
|  |  |  | 3300005326.a:Ga0074195_1005032 |
| Groundwater a2 |  |  | 3300005326.a:Ga0074195_1000033 |
|  |  |  | 3300005326.a:Ga0074195_1000027 |
|  |  |  | 3300005326.a:Ga0074195_1000168 |
|  |  |  | 3300005326.a:Ga0074195_1000072 |
|  | Ga0074195_10108343 | GU127467 | 3300005326.a:Ga0074195_1010834 |
| BL (MCG) SepCysS |  |  | 3300010324.a:Ga0129297_10005987 |
| BL (MCG) bin |  |  | 3300010328.a:Ga0129298_10002370 |
|  |  |  | 3300010324.a:Ga0129297_10001997 |
| BOG (Asgard) archaeon |  |  | 3300003218.a:JGI26339J46600_10000823 |
|  |  |  | 3300003218.a:JGI26339J46600_10134722 |
|  |  |  | 3300003218.a:JGI26339J46600_10001817 |
|  |  |  | 3300003218.a:JGI26339J46600_10000844 |
|  |  |  | 3300003218.a:JGI26339J46600_10002441 |
|  |  |  | 3300003218.a:JGI26339J46600_10002384 |
|  |  |  | 3300003218.a:JGI26339J46600_10003240 |
|  |  |  | 3300003218.a:JGI26339J46600_10003870 |
|  |  |  | 3300003218.a:JGI26339J46600_10004459 |
|  |  |  | 3300003218.a:JGI26339J46600_10001324 |
|  |  |  | 3300003218.a:JGI26339J46600_10012099 |
|  |  |  | 3300003218.a:JGI26339J46600_10000997 |
|  |  |  | 3300003218.a:JGI26339J46600_10001173 |
|  |  |  | 3300003218.a:JGI26339J46600_10001041 |
|  |  |  | 3300003218.a:JGI26339J46600_10002996 |
| (probably) | JGI26339J46600_100283311 | EU731647 | 3300003218.a:JGI26339J46600_10028331 |
| WOR (Asgard-like) SepRS | SMTZ23_100471309 | OLS17870 | 3300002053.a:SMTZ23_10047130 |
|  |  |  | 3300001753.a:JGI2171J19970_10118396 |
|  |  |  | 3300001753.a:JGI2171J19970_10151886 |
|  |  |  | 3300001751.a:JGI2172J19969_10101847 |
|  |  |  | 3300001751.a:JGI2172J19969_10035237 |
| WOR (Asgard) SepCysS |  |  | 3300002053.a:SMTZ23_10000431 |
| WOR (Asgard-like) SepCysS |  |  | 3300002053.a:SMTZ23_10067740 |
|  |  |  | 3300001753.a:JGI2171J19970_10488438 |
|  | SMTZ1_100055877 | OLS19033 | 3300002052.a:SMTZ1_10005587 |
| GB (Altiarchaeales) archaeon No. 1 |  |  | 3300010332.a:Ga0116200_10008766 |
|  |  |  | 3300010332.a:Ga0116200_10005001 |
| GB archaeon No. 2 |  |  | 3300010332.a:Ga0116200_10002063 |
| GB archaeon No. 3 |  |  | 3300010330.a:Ga0136651_10015067 |
|  | Ga0136651_100013727 | AJS13171 | 3300010330.a:Ga0136651_10001372 |
| GB archaeon No. 4 |  |  | 3300010330.a:Ga0136651_10088126 |
|  |  |  | 3300010330.a:Ga0136651_10021977 |
| GB archaeon No. 5 | Ga0116200_100010773 | KYK30570 | 3300010332.a:Ga0116200_10001077 |
| GB archaeon No. 6 |  |  | 3300010330.a:Ga0136651_10043221 |
| GB (W8A-19) archaeon No. 7 |  |  | 3300010332.a:Ga0116200_10024747 |
|  |  |  | 3300010332.a:Ga0116200_10109079 |
|  | Ga0116200_103175671 | Ga0105158_100005513 | 3300010332.a:Ga0116200_10317567 |
|  |  |  | 3300010332.a:Ga0116200_10066030 |
|  |  |  | 3300010332.a:Ga0116200_10053834 |
| GB (AK8) archaeon No. 8 |  |  | 3300010332.a:Ga0116200_10533927 |
|  |  |  | 3300010332.a:Ga0116200_10264246 |
|  |  |  | 3300010332.a:Ga0116200_10284406 |
|  |  |  | 3300010332.a:Ga0116200_10090925 |
|  |  |  | 3300010332.a:Ga0116200_10568540 |
|  | Ga0116200_100970881 | KP091068 | 3300010332.a:Ga0116200_10097088 |
| GB (pMC2A209) archaeon No. 9 |  |  | 3300010332.a:Ga0116200_10040516 |
|  |  |  | 3300010332.a:Ga0116200_10002059 |
|  | Ga0116200_100160501 | AB175574 | 3300010332.a:Ga0116200_10016050 |
| GB (pMC2A209) archaeon No. 10 |  |  | 3300010332.a:Ga0116200_10067475 |
|  |  |  | 3300010332.a:Ga0116200_10014528 |
|  |  |  | 3300010332.a:Ga0116200_10040982 |
|  | Ga0116200_101386802 | KP091046 | 3300010332.a:Ga0116200_10138680 |
|  | Ga0116200_104926031 | KP091046 | 3300010332.a:Ga0116200_10492603 |
| GB (pMC2A209) archaeon No. 11 |  |  | 3300010332.a:Ga0116200_10051283 |
| Altiarchaeales archaeon SCGC AAA252-I15 |  |  | AQSC01000000 |
|  | Ga0077109_10009638 | KU868094 | 3300005645.a:Ga0077109_1000963 |
|  |  |  | 3300005645.a:Ga0077109_1001640 |
| Altiarchaeales archaeon MSI_SM1 | CCXY01000234 | KU868082 | CCXY01000234 |
|  |  |  | CCXY01000203 |
|  |  |  | CCXY01000036 |
| Altiarchaeales archaeon WOR_SM1_SCG | JGI24422J19971_100105003 | MCBE01000267 | 3300001854.a:JGI24422J19971_10010500 |
|  |  |  | MCBE01000153 |
| Altiarchaeales archaeon WOR_SM1_79 |  |  | MCBD01000257 |
|  |  |  | 3300001854.a:JGI24422J19971_10001723 |
|  |  |  | MCBD01000004 and MCBD01000132 |
| (Probably) |  |  | 3300001854.a:JGI24422J19971_10000095 |
| WOR_SM1_79 or WOR_SM1_SCG |  |  | 3300001854.a:JGI24422J19971_10016442 |
| Altiarchaeales archaeon WOR_SM1_86-2 | JGI24422J19971_100171005 | MCBC01000108 | 3300001854.a:JGI24422J19971_10017100 |
|  |  |  | 3300001854.a:JGI24422J19971_10014574 |
|  |  |  | MCBC01000019 |
| Z7ME43 archaea DG-70 and DG-70-1 |  |  | LSSB01000028 |
|  |  |  | LSSC01000183 |
|  | JGI2171J19970_100029886 | JQ245675 | 3300001753.a:JGI2171J19970_10002988 |
|  |  |  | 3300001854.a:JGI24422J19971_10019800 |
|  |  |  | 3300001753.a:JGI2171J19970_10000079 |
|  |  |  | 3300001753.a:JGI2171J19970_10002673 |
| SSWTFF a1 |  |  | 3300009596.a:Ga0105156_1000007 |
|  |  |  | 3300009596.a:Ga0105156_1000703 |
| Wastewater b1 |  |  | 3300001095.a:JGI12104J13512_10004074 |
| Hot spring Na1 |  |  | 3300005278.a:Ga0065718_1025536 |
| Hot spring Ya1 |  |  | 3300002966.a:JGI24721J44947_10021374 |
| Hot spring Ya2 |  |  | 3300002966.a:JGI24721J44947_10007340 |
|  |  |  | 3300002966.a:JGI24721J44947_10006107 |
|  | JGI24721J44947_100933081 | KX213896 | 3300002966.a:JGI24721J44947_10093308 |
| Hot spring Ya3 |  |  | 3300002966.a:JGI24721J44947_10053492 |
|  |  |  | 3300002966.a:JGI24721J44947_10012638 |
|  |  |  | 3300002966.a:JGI24721J44947_10007631 |
| Sulfidic spring Ya1 |  |  | 3300005860.a:Ga0080004_1158381 |
|  |  |  | 3300005860.a:Ga0080004_1089617 |
|  | Ga0080004_113893371 | CNBRG16SA | 3300005860.a:Ga0080004_1138933 |
| Sulfidic spring Ya2 |  |  | 3300005860.a:Ga0080004_1206622 |
|  |  |  | 3300005860.a:Ga0080004_1131213 |
|  | Ga0080004_11765496 | KP784727 | 3300005860.a:Ga0080004_1176549 |
| Sulfidic spring Ya3 |  |  | 3300005860.a:Ga0080004_1159908 |
|  |  |  | 3300005860.a:Ga0080004_1097279 |
|  | Ga0080004_107280538 | DQ243735 | 3300005860.a:Ga0080004_1072805 |
| Sulfidic spring Ya4 | Ga0080004_112641834 | FJ936656 | 3300005860.a:Ga0080004_1126418 |
|  |  |  | 3300005860.a:Ga0080004_1228288 |
| Hot spring Ja1 |  |  | 3300007999.a:Ga0105163_1006643 |
|  |  |  | 3300007999.a:Ga0105163_1011070 |
|  | Ga0105163_10039835 | FN553679 | 3300007999.a:Ga0105163_1003983 |
| Hot spring Ja2 |  |  | 3300008000.a:Ga0105162_1002065 |
|  |  |  | 3300008000.a:Ga0105162_1000880 |
|  | Ga0105162_100098210 | EU924234 | 3300008000.a:Ga0105162_1000982 |
| Hot spring Ja3 |  |  | 3300008000.a:Ga0105162_1017647 |
| Jinze (W8A-19) archaeon |  |  | 3300008000.a:Ga0105162_1001467 |
|  |  |  | 3300008000.a:Ga0105162_1000958 |
|  | Ga0105162_10319741 | Ga0105158_100005513 | 3300008000.a:Ga0105162_1031974 |
|  |  |  | 3300008000.a:Ga0105162_1031974 |
|  |  |  | 3300008000.a:Ga0105162_1005487 |
|  |  |  | 3300008000.a:Ga0105162_1010765 |
|  |  |  | 3300008000.a:Ga0105162_1048702 |
|  |  |  | 3300008000.a:Ga0105162_1019307 |
|  |  |  | 3300008000.a:Ga0105162_1004597 |
| pSL50 archaea JGI MDM2 LHC4sed-1-M8/N8 |  |  | 2643221461 |
|  |  |  | 2643221462 |
|  |  |  | 2643221450 |
|  |  |  | 3300008019.a:Ga0105158_1019547 |
|  |  |  | 3300008019.a:Ga0105158_1048210 |
|  |  |  | 3300008019.a:Ga0105158_1008947 |
|  |  |  | 3300008019.a:Ga0105158_1012818 |
|  | Ga0105158_10004456 | EU924234 | 3300008019.a:Ga0105158_1000445 |
| LHC4sed (W8A-19) archaeon |  |  | 3300008019.a:Ga0105158_1000011 |
|  |  |  | 3300008019.a:Ga0105158_1000003 |
|  | Ga0105158_100005513 | KM221272 | 3300008019.a:Ga0105158_1000055 |
|  |  |  | 3300008019.a:Ga0105158_1000434 |
|  |  |  | 3300008019.a:Ga0105158_1000225 |
|  |  |  | 3300008019.a:Ga0105158_1000017 |
|  |  |  | 3300008019.a:Ga0105158_1000077 |
|  |  |  | 3300008019.a:Ga0105158_1000576 |
|  |  |  | 3300008019.a:Ga0105158_1000238 |
| CP (AK8) archaea |  |  | 3300010308.a:Ga0136652_1000925 |
|  |  |  | 3300010308.a:Ga0136652_1000397 |
|  |  |  | 3300010308.a:Ga0136652_1000125 |
|  |  |  | 3300010308.a:Ga0136652_1005653 |
|  |  |  | 3300010308.a:Ga0136652_1003451 |
|  |  |  | 3300010308.a:Ga0136652_1000127 |
|  |  |  | 3300010308.a:Ga0136652_1000286 |
|  |  |  | 3300010308.a:Ga0136652_1000996 |
|  |  |  | 3300010308.a:Ga0136652_1000089 |
|  |  |  | 3300010308.a:Ga0136652_1000328 |
|  | Ga0136652_10006987 | AY555814 | 3300010308.a:Ga0136652_1000698 |
| Dehalococcoidia bacterium CG2_30_46_9 |  |  | 3300005235.a:Ga0066637_10056611  MNYE01000003.1 |
| Dehalococcoidia bacterium CG2_30_46_19 |  |  | 3300005237.a:Ga0066644_10004197  MNYD01000137.1 |
| CG bacterium No. 3 |  |  | 3300005236.a:Ga0066636_10011280 |
| Crystal Geyser b4 |  |  | 3300005236.a:Ga0066636_10001963 |
|  |  |  | 3300005236.a:Ga0066636_10003412 |
| CG (Parcubacteria) bacterium |  |  | 3300005235.a:Ga0066637_10039315 |
|  |  |  | 3300004107.a:Ga0065179_1171907 |
|  | Ga0066637_100217121 (IleRS) | KKT87699 | 3300005235.a:Ga0066637_10021712 |
|  |  |  | 3300005235.a:Ga0066637_10119152 |
| Alkali sediment b1 |  |  | 3300000362.a:SL_1KL_011_SEDDRAFT_10011494 |
| Alkali sediment b2 |  |  | 3300000362.a:SL_1KL_011_SEDDRAFT_10000546 |
| Lake sediment b1 |  |  | 3300009039.a:Ga0105152_10009400 |
| Lake sediment b2 |  |  | 3300009039.a:Ga0105152_10009423 |
| Lake sediment b3 |  |  | 3300009504.a:Ga0114946_10007232 |
|  |  |  | 3300009504.a:Ga0114946_10018055 |
| Lake Sakinaw b1 |  |  | 3300005645.a:Ga0077109_1000075 |
| Crude oil b1 |  |  | 3300001749.a:JGI24025J20009_10000084 |
| Hot spring Yb1 |  |  | 3300002966.a:JGI24721J44947_10000937 |
| MTBE-degrading b1 |  |  | 3300005254.a:Ga0068714_10004623 |
| Deep marine Sb1 |  |  | 3300001854.a:JGI24422J19971_10006019 |
| Deep marine Sb2 |  |  | 3300001854.a:JGI24422J19971_10001652 |
| MBG-E archaeon |  |  | 3300002481.a:JGI24020J35080_1000057 |
|  | JGI24020J35080_1000023169 | AB329774 | 3300002481.a:JGI24020J35080_1000023 |
|  |  |  | 3300002481.a:JGI24020J35080_1000005 |
|  |  |  | 3300002481.a:JGI24020J35080_1000115 |
|  |  |  | 3300002481.a:JGI24020J35080_1000016 |
|  |  |  | 3300002481.a:JGI24020J35080_1000105 |
| Methermicoccaceae archaeon |  |  | 3300002481.a:JGI24020J35080_1000146 |
|  |  |  | 3300002481.a:JGI24020J35080_1000986 |
|  |  |  | 3300002481.a:JGI24020J35080_1000033 |
|  |  |  | 3300002481.a:JGI24020J35080_1000261 |
|  |  |  | 3300002481.a:JGI24020J35080_1000039 |
|  | JGI24020J35080_100005273 | KT152866 | 3300002481.a:JGI24020J35080_1000052 |
| Bathyarchaeota archaeon |  |  | 3300002481.a:JGI24020J35080_1000009 |
|  | JGI24020J35080_1000002579 | SSF21-22_1362A11_015 | 3300002481.a:JGI24020J35080_1000002 |
|  |  |  | 3300002481.a:JGI24020J35080_1000021 |
|  |  |  | 3300002481.a:JGI24020J35080_1000019 |
|  |  |  | 3300002481.a:JGI24020J35080_1000111 |
| (pylSn) unknown (Sandy's Spring West) |  |  | 3300009598.a:Ga0105154_1007862 |
|  |  |  | 3300009598.a:Ga0105154_1010143 |
|  |  |  | 3300009598.a:Ga0105154_1003385 |
|  |  |  | 3300009598.a:Ga0105154_1089079 |
|  |  |  | 3300009598.a:Ga0105154_1005677 |
|  |  |  | 3300009598.a:Ga0105154_1029239 |
|  |  |  | 3300009598.a:Ga0105154_1033458 |
|  |  |  | 3300009598.a:Ga0105154_1036596 |
|  |  |  | 3300009598.a:Ga0105154_1026421 |
|  |  |  | 3300009598.a:Ga0105154_1010292 |
| (pylSn) unknown archaeon  (Washburn Spring) | Ga0080004_11365474 (GroEL) | KON32232 | 3300005860.a:Ga0080004_1136547 |
|  |  |  | 3300005860.a:Ga0080004_1147017 |
| unknown archaeon  (Washburn Spring) | Ga0080004_119822121 (FepB) | ADM27435 | 3300005860.a:Ga0080004_1198221 |
| Archaeoglobus archaeon  (Washburn Spring) | Ga0080004_10963946 (Cdc6A) | WP_010877755 | 3300005860.a:Ga0080004_1096394 |
|  |  |  | 3300005860.a:Ga0080004_1099158 |
| bacteria (Soda Lake) |  |  | 3300000575.a:SL_4KL_010_BRINEDRAFT_10001702 |
|  |  |  | 3300000575.a:SL_4KL_010_BRINEDRAFT_10025033 |
|  |  |  | 3300000575.a:SL_4KL_010_BRINEDRAFT_10005171 |
|  |  |  | 3300000575.a:SL_4KL_010_BRINEDRAFT_10001060 |
|  |  |  | 3300000575.a:SL_4KL_010_BRINEDRAFT_10033048 |
|  |  |  | 3300000575.a:SL_4KL_010_BRINEDRAFT_10005171 |
|  |  |  | 3300000575.a:SL_4KL_010_BRINEDRAFT_10001060 |
