# Supplementary material for: RNA-Dependent Cysteine Biosynthesis in Bacteria and Archaea
Source: mBio. 2017 May 9;8(3):e00561-17. doi: 10.1128/mBio.00561-17 (PMC5424206; doi:10.1128/mBio.00561-17)
Supplement: TEXT S1 [file mbo002173292s1.docx]

**Supplemental text about tRNA-Thr-ED proteins.** Here, tRNA-Thr-ED is defined as a homolog corresponding to the editing domain of archaeal threonyl-tRNA synthetase (ThrRS-R) (1) and to the editing domain of archaeal trans-editing ThrRS-ed protein (2) (Fig. S4A). ThrRS-ed is composed of the N-terminal editing domain and the C-terminal anticodon binding domain of ThrRS-R and responsible for seryl-tRNA^Thr^ hydrolysis in some archaea, because these archaea lack ThrRS-R but have another threonyl-tRNA synthetase lacking the editing domain (ThrRS-cat) (2). Interestingly, tRNA-Thr-ED proteins lack the anticodon binding domain contrary to all previously reported Seryl-tRNA^Thr^ editing/hydrolysis proteins (2, 3). Phylogenetic analysis of tRNA-Thr-ED proteins and the editing domains of archaeal ThrRS-R/ThrRS-ed revealed a wide distribution of tRNA-Thr-ED homologs among prokaryotes (Fig. S4B). Furthermore, these homologs are more similar to the editing domain of archaeal ThrRS-R/ThrRS-ed than d-tyrosyl-tRNA^Tyr^ deacylase (DTD) which is known to be related with ThrRS-R/ThrRS-ed (4-6) (Fig. S4B). The residues recognizing the aminoacyl-adenosine moiety in ThrRS-R/ThrRS-ed are well conserved in tRNA-Thr-ED homologs except for the residue corresponding to the Glu135 of the crystal structure of *M. jannaschii* ThrRS editing domain (PDB id: 4RRF) (5, 7) (Fig. S4A, B). This residue is responsible for selecting the amino acid moiety (5, 7). Thus, it is likely that tRNA-Thr-ED group proteins may hydrolyze aminoacyl-tRNAs or hydroxyacyl-tRNAs. Among the tRNA-Thr-ED groups, SepCysS-associated ones of some Hadesarchaea/MSBL1 archaea and some methanogens (Figs. 1B, S1) are most similar to the editing domain of archaeal ThrRS-R/ThrRS-ed (Fig. S4A). Thus, it is suggested that the SepCysS-associated tRNA-Thr-ED genes are derived from archaeal ThrRS-R/ThrRS-ed genes or that they are sister group to archaeal ThrRS-R/ThrRS-ed genes. Furthermore, methanogens’ tRNA-Thr-ED proteins have a C-terminal cysteine-rich peptide (Fig. S4C), implying their involvement in sulfur metabolism. The finding of the two kinds of SepCysS-associating tRNA-Thr-ED proteins (with or without the C-terminal peptide) may validate the phylogenetic division of the Hadesarchaea/MSBL1 group and the methanogen group in the SepCysS clade VII (Figs. 1B, S1).

1. **Andam CP, Gogarten JP.** 2011. Biased gene transfer and its implications for the concept of lineage. Biol Direct **6:**47.

2. **Korenčić D, Ahel I, Schelert J, Sacher M, Ruan B, Stathopoulos C, Blum P, Ibba M, Söll D.** 2004. A freestanding proofreading domain is required for protein synthesis quality control in Archaea. Proc Natl Acad Sci U S A **101:**10260-10265.

3. **Liu Z, Vargas-Rodriguez O, Goto Y, Novoa EM, Ribas de Pouplana L, Suga H, Musier-Forsyth K.** 2015. Homologous trans-editing factors with broad tRNA specificity prevent mistranslation caused by serine/threonine misactivation. Proc Natl Acad Sci U S A **112:**6027-6032.

4. **Rigden DJ.** 2004. Archaea recruited D-Tyr-tRNA^Tyr^ deacylase for editing in Thr-tRNA synthetase. RNA **10:**1845-1851.

5. **Hussain T, Kruparani SP, Pal B, Dock-Bregeon AC, Dwivedi S, Shekar MR, Sureshbabu K, Sankaranarayanan R.** 2006. Post-transfer editing mechanism of a D-aminoacyl-tRNA deacylase-like domain in threonyl-tRNA synthetase from archaea. EMBO J **25:**4152-4162.

6. **Bhatt TK, Soni R, Sharma D.** 2016. Recent Updates on DTD (d-Tyr-tRNA^Tyr^ Deacylase): An Enzyme Essential for Fidelity and Quality of Protein Synthesis. Front Cell Dev Biol **4:**32.

7. **Ahmad S, Muthukumar S, Kuncha SK, Routh SB, Yerabham AS, Hussain T, Kamarthapu V, Kruparani SP, Sankaranarayanan R.** 2015. Specificity and catalysis hardwired at the RNA-protein interface in a translational proofreading enzyme. Nat Commun **6:**7552.
