# Supplementary material for: RNA-Dependent Cysteine Biosynthesis in Bacteria and Archaea
Source: mBio. 2017 May 9;8(3):e00561-17. doi: 10.1128/mBio.00561-17 (PMC5424206; doi:10.1128/mBio.00561-17)
Supplement: FIG S6 [file mbo002173292sf6.pdf]

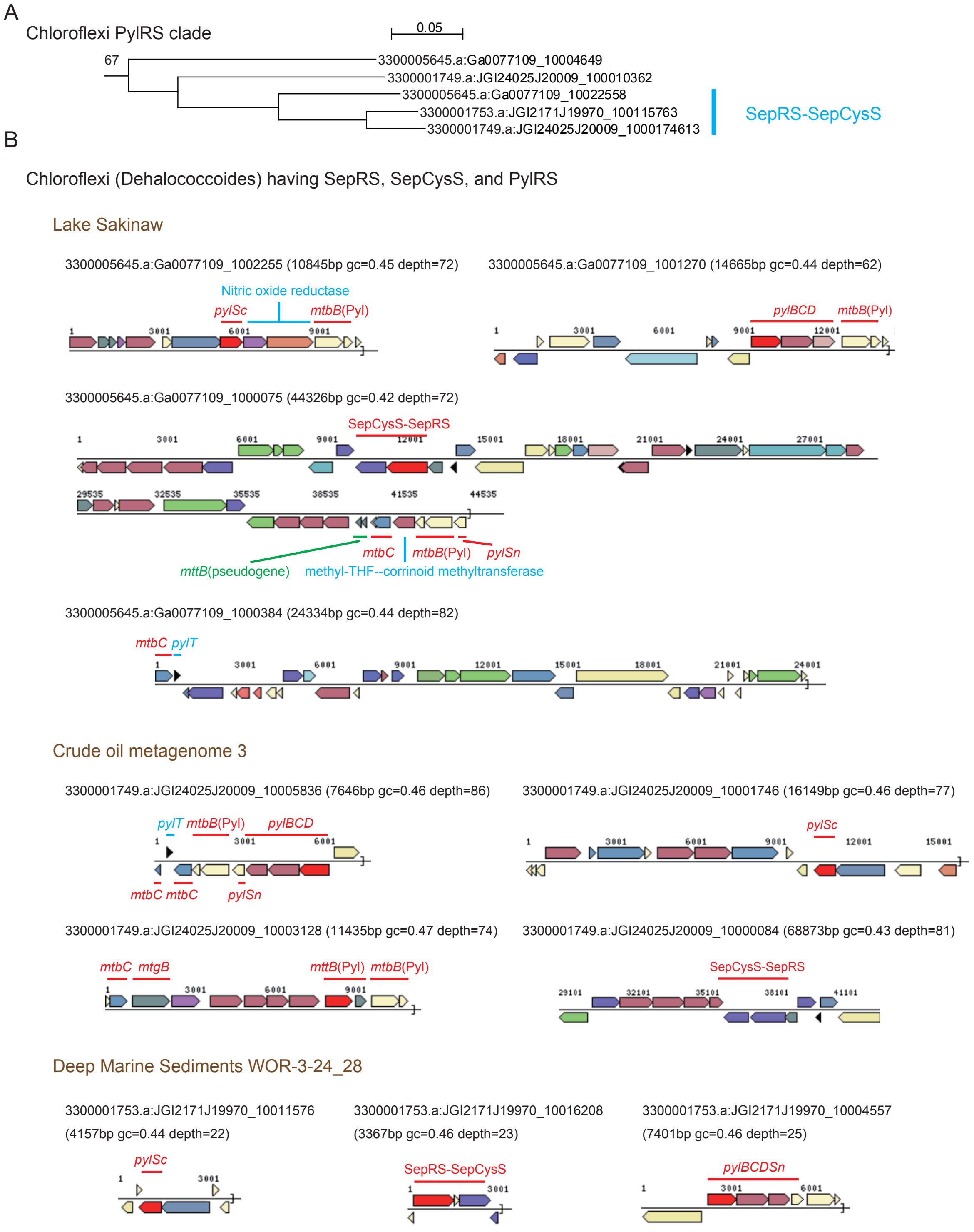
A
Chloroflexi PylRS clade
0.05
67
3300005645.a:Ga0077109_10004649
3300001749.a:JGI24025J20009_100010362
3300005645.a:Ga0077109_10022558
3300001753.a:JGI2171J19970_100115763
3300001749.a:JGI24025J20009_1000174613
SepRS-SepCysS
B
Chloroflexi (Dehalococcoides) having SepRS, SepCysS, and PylRS
Lake Sakinaw
3300005645.a:Ga0077109_1002255 (10845bp gc=0.45 depth=72)
Nitric oxide reductase
pylSc
mtbB(Pyl)
3300005645.a:Ga0077109_1001270 (14665bp gc=0.44 depth=62)
pylBCD
mtbB(Pyl)
3300005645.a:Ga0077109_1000075 (44326bp gc=0.42 depth=72)
SepCysS-SepRS
mtbC
mtbB(Pyl)
pylSn
mttB(pseudogene)
methyl-THF--corrinoid methyltransferase
3300005645.a:Ga0077109_1000384 (24334bp gc=0.44 depth=82)
mtbC
pylT
Crude oil metagenome 3
3300001749.a:JGI24025J20009_10005836 (7646bp gc=0.46 depth=86)
pylT
mtbB(Pyl)
pylBCD
mtbC
mtbC
pylSn
3300001749.a:JGI24025J20009_10001746 (16149bp gc=0.46 depth=77)
pylSc
3300001749.a:JGI24025J20009_10003128 (11435bp gc=0.47 depth=74)
mtbC
mtgB
mttB(Pyl)
mtbB(Pyl)
3300001749.a:JGI24025J20009_10000084 (68873bp gc=0.43 depth=81)
SepCysS-SepRS
Deep Marine Sediments WOR-3-24_28
3300001753.a:JGI2171J19970_10011576
(4157bp gc=0.44 depth=22)
pylSc
3300001753.a:JGI2171J19970_10016208
(3367bp gc=0.46 depth=23)
SepRS-SepCysS
3300001753.a:JGI2171J19970_10004557
(7401bp gc=0.46 depth=25)
pylBCDSn
