# Supplementary material for: RNA-Dependent Cysteine Biosynthesis in Bacteria and Archaea
Source: mBio. 2017 May 9;8(3):e00561-17. doi: 10.1128/mBio.00561-17 (PMC5424206; doi:10.1128/mBio.00561-17)
Supplement: DATA SET S1 [file mbo002173292sd1.rtf]

Sequence and alignment data

#Mega
!Title SepRS alignment;

#Lake Kivu metagenomic archaeon
----------------MFNTNEIKKLAK-QDYEKAWLETAKLLKTSG----KILEWKKQQ
--GSPNAVTELVLKFREIFLKYGFEEMINPTIVEETEVYKQYGSEAPVILDRCFYLAGLP
RPDIGISKERTQQIKKIG------------------------DVDIEKLKDIFKGYKKGE
IEGDNLVEELVNKLNIKTEQATAILS--IFPELKQIKP--VPTKLTLRSHMTGVWFSVIS
--SLIEKRSMPIKLFSIGPKFRREQKLDATHLYESLTASLVVVTRDMSLEDGQELAKIIL
NEFGFGKVDFQIKKA-TSKYYAPNTEFEIFIKHPKPEK----------WIEIGDGGFYSP
VSLANYNIK--HPVFNIGFGVERLAMLLTGVDDIRKLVYPQFYE--EIEFSDKEIAKSIE
LVEKPETKQGREIADLIYKIAKLKKDEVAPCKFVVYRGRFDK------------------
--KQIEVKIIEVEKGKHLVGPAGFNRIIVKDGNIIGTLKG-----------------DGV
EVSD-YMTGFANLAAKEIEAFVKTDKK---------------------------------
----------QTEIRIGMVKGLSDINFKVSEPVRRFIESNNKK-IDIRGPMFVSVVVKKV
---------------------------------

#3300002053.a:SMTZ23_1001501310
MVIKKERKKKELKEPVDARVIDVSYEEPTETPEDAQVELEEERDERI------SYL-TGK
--GTQHPVHELIQELRNILINSGFNELENSFFVAEKDIFKQYDVNQNLVFDKIYHLAEYP
RPAIELNQEQITQLSGIKS-----------------------DINIEVLKSIFNDYRENK
IENYQIFQRLMAELNLNCDQATNILK--IIPELNEKQP--KLTNVTLRSSITSSWFTTLA
--AIADKESLPIKIFSTGIWFKRGPKLDELRLYSHYGASCIITDENINISNGKVIAEEIL
NRLGFKEMEFKDNVE-NRNFNVTCDELGIYV------D----------DVEIATCGLFSP
EVLKKYGIE--LPTLYINFGLEHMVMVQKGFDDIRELMFPQFYK--AWKLNDEEIGKSLQ
FILKPRTELGKTIAKNLVKICEKNSHTISPCEFTVWEGPVKVKKN------KVQSIEPVE
LDKQLVVKVVKREKDAKLCGPAYLNEIVVKNGDIFSVQNN---------KEANHDLTEAY
HTNIRYLDAFSKLVGRRIEKKLTKGGFE--------------------------------
---------GGYDIKIGIIKEIDEINLQLNGGAVRYLLTNNKK-IDVRGPMFVNVECELK
DITEKKD--------------------------

#GB archaeon No. 5
----------------------MEKRTF-VERTSRGKDTGRALEDKV------DFL-TGQ
--GVPHPVSRAAEDMRKVMLSFGFDEVEISYLIPEEVLFRAYSDLFPVYQDITYYLGWLK
HRMMELEEERPEELTDE-------------------------EWE--CLKDIVKDVVDEN
LSLEEGVYRTMEELKVDFRGALRTLL--EIWEQVRGEV--VPERLTLRSSMPAAWLPTLR
--ALSSEDVFPLKLFTISKVFRREHLMEETHGRVFTLLSAVIEDPEIDLDAGLTVVRSVL
EKLGVRDVEFRYKHY-SPPYYQPAMEYELFLR----------------SYEVGTCGVFSE
EVLRSAGVP--NPVFHLEIGVERLLMAREGYPGVKELEFPQFYR--KWKLKDEEVAEGIK
PVVSPRSEWGRGVAQLLYEAFMKAREEGKVREKVTSERLTVRGEE----------IHPSE
EGRLKLTVFIGGLLKDEILGPAAGDRILVHEGNILGVPSR---------EVKSYLKRGAV
LTDYDYVRPLCLLAASRMESWLKEGREG--------------------------------
----------KGEVTVERVRSLQDVNLTLLPHVKEYVLSRSRK-IEVQGPLFFRVGYRGV
VNE------------------------------

#GB archaeon No. 6
------------------------------SQKKEGTLDQMKSEDIV------NYL-YGR
--GHPHPISEGVSEMRELLLDSGFDEIYTSFFVSRRDLRELTGELYPVLRDSVYHLSWVR
PAPLGPTPDVDRRLMARFP-----------------------DLDR-AELWNILDALDED
SSGEELLAQLMDELGLDVDDALTVMN--MIPELNRGDP--EVGDMTLRSFMPTSWLSTLE
--AIFDPENLPLRLFTVATAFRREAELDSSHIRTYNILSLAIGDDDLTLEKGMAVLRRIF
DNQGLSDISFLEKSY-QFPFFEKGTEIEIFGG----------------DLELGTCGMCSG
EILSSRGVT--TPVFIADIGVERVLMHRYGYPDIRQLLYPQFFA--AWNLTDEEIASSLR
YLRKPQTDYGREIARAIHRTYRECREKEEDEKRTAWKGILASSDYGKFLVTEERAKELGI
DGRPAEVVLRESRKGMGLCGPGAFNEIWVDQGNVVSVPPN---------MAKTLEERGAS
RTNKTFVKAFSRYAAWKIERSLERG-----------------------------------
----------HTSRKVEKIRDLEGINLKLTSKALYYILSHKKK-VDVQGPVYLSFSFRVK
DPR------------------------------

#GB archaeon No. 2
-------------MVLPIDEIRKKLREN-KSFEDLWLEYSDIFPDIT----RRIPLVKSM
--GKSHPVVDLIFTLREKLLSLGFTEVINPVFIPVEDVYKQYGPEAPVILDRIYFLAGLP
RPDIGLSTEYIDKIRKEVG-----------------------EIDFNILKNVLRKYRERE
IEADELIEVLKRELGISDKQCLRLIS--ILKDFFELKP--SPLAATLRSHMTAAWFITLS
--RVLRYLDPPLRLFSIDWVFRREQRVDKEHLRYYHSASIVVVDDSLNEEAAVELTKSIM
SHLGLSSVKIVKKKD-TSRYYAYGKEYEVYVEYR--GN----------LQEIGTFGLYSP
IALANYEIP--YPVYNFGIGVERLAQVLYDVDDIRVLVFPYLY----SVISDEDIAARIK
PILAPRTSYGEKLEKVILKNIEKYRNKTGPFKLEVYR----D------------------
--DKIKIYLYEPDP-KPYAGPAAFNRIYVHRGNISSL-----------------EEHEGV
YVGR-YIDFIVKKFARLVEDGE--------------------------------------
----------SGWLRVRWVEGPADANIRISSNVLKYIHEKQGV-VDIKGPVFVDIIVERN
---------------------------------

#Methanopyrus kandleri
------------MPFDRDKLEELRSLAQ-RDFDRAWKEGAKLVREPG-LRDRYPRLKVET
--GEPHPLFETIQQLREAYLRAGFREVVNPVIIPEEEVYKQFGPEAAAVLDRCFYLAGLP
RPDVGLGADKVEKLAEVLGRE-------------------PSEDEVERLRETLHAYKKGE
IDGDELTHEIAEALDTDDGTAVRILD-EVFPELKRLKPEPLEPPLTLRSHMTAGWFITLS
--EILKREDPPLKLFSIDRCFRREQREDESHLMTYHSASCVVVSDDVTVDTGKAVAEAIL
RQFGFEDFEFVPDEK-MSKYYVPGTQTEVYAYHPDLEDSIEDEELGPGWVEIATFGLYSP
VALAEYGID--YPVMNLGIGVERLCMVLHGIDDVRSLAY-VEYE--PWEPSDLELARMID
YERKPATSFGERLVREVVRGLHEHADEEGPVEVELFRGEFGD------------------
--REVVVHAVEEEKGEPLAGPAAFNRVYVLDGNLYAVPPE--------GDFGREIREEGV
YSGVSFEEGLAARLAYEVEELLATGGG---------------------------------
----------ETTVSVRKVSRPSQVNLSLPRKLLRYVTKKGGE-IEIKGPVFVTLRAEVR
---------------------------------

#Methanothermobacter thermautotrophicus
-----------------MKRKDIVKLSR-RDFERAWLESGKSLRKPH-HDMQYPRLRFET
--GKSHVLYDTIWMIREAYLRLGFSEMVNPLLIDEEHIYRQFGPEAPAVLDRCFYLGGLP
RPDIGLGTGRIQMIEDMGID--------------------VSDEKLENLKEVFRSYKKGD
LSGDDLVLEVSNALEVESHDGLRVLE-RVFPEIRDLKP--VSGRTTLRSHMTSGWFISLQ
--NIHDRYRMPLKLFSIDRCFRREQKEDSSHLMTYHSASCVVVDHEVPLDVGKAVAEGLL
EHLGFSRFRFRPDEK-KSKYYIPGTQTEVYAYHPLLKE----------WVEVATFGLYSP
IALSMYGID--QEVMNLGVGVERVAMILNQASDVREMVYPQIYG--EWRLSDRDIAEMLR
INLHPVTSDGRMLMEKIVKTWRAHADAPSPCSFEVYSGEFLG------------------
--RRIEVSALEVEENTRLLGPAVWNTVYIHDGNILGVPPGTE----LDSELITRARKEGL
NTGITYMEALAAEAAYRIEEMVVSGAE---------------------------------
----------EVEVRSTIARSLSDLNLTLEDTAMRYITGKNRE-IDLRGPLFSTIRCRLR
G--------------------------------

#Methanocaldococcus jannaschii
---------------MRFDIKKVLELAE-KDFETAWRETRALIKDKH-IDNKYPRLKPVY
--GKPHPVMETIERLRQAYLRMGFEEMINPVIVDEMEIYKQFGPEAMAVLDRCFYLAGLP
RPDVGLGNEKVEIIKNLGID--------------------IDEEKKERLREVLHLYKKGA
IDGDDLVFEIAKALNVSNEMGLKVLE-TAFPEFKDLKP--ESTTLTLRSHMTSGWFITLS
--SLIKKRKLPLKLFSIDRCFRREQREDRSHLMSYHSASCVVVGEDVSVDDGKVVAEGLL
AQFGFTKFKFKPDEK-KSKYYTPETQTEVYAYHPKLGE----------WIEVATFGVYSP
IALAKYNID--VPVMNLGLGVERLAMIIYGYEDVRAMVYPQFY---EYRLSDRDIAGMIR
VDKVPILDEFYNFANELIDICIANKDKESPCSVEVKREFNFNGER---------------
--RVIKVEIFENEPNKKLLGPSVLNEVYVYDGNIYGIPPTFEGVKEQYIPILKKAKEEGV
STNIRYIDGIIYKLVAKIEEALVSNVD---------------------------------
----------EFKFRVPIVRSLSDINLKIDELALKQIMGENKV-IDVRGPVFLNAKVEIK
---------------------------------

#Methanococcus maripaludis
----------------MFKREEIIEMAN-KDFEKAWIETKDLIKAKK-VNESYPRIKPVF
--GKTHPVNDTIENLRQAYLRMGFEEYINPVIVDERDIYKQFGPEAMAVLDRCFYLAGLP
RPDVGLSDEKISQIEKLGIK--------------------VS-EHKESLQKILHGYKKGT
LDGDDLVLEISNALEISSEMGLKILE-EVFPEFKDLTA--VSSKLTLRSHMTSGWFLTVS
--DLMNKKPLPFKLFSIDRCFRREQKEDKSHLMTYHSASCAIAGEGVDINDGKAIAEGLL
SQFGFTNFKFIPDEK-KSKYYTPETQTEVYAYHPKLKE----------WLEVATFGVYSP
VALSKYGID--VPVMNLGLGVERLAMISGNFADVREMVYPQFY---EHKLDDRAVASMVK
LDKVPVMDEIYDLTKELIDSCVKNKDLKSPCELTIEKTFSFGKTK---------------
--KNVKINIFEKEEGKNLLGPSILNEIYVYDGNVIGIPESFDGVKEEFKEFLEKGKSEGV
ATSIRYIDALCFKLTSKLEEAIVTNTS---------------------------------
----------EFKVKVPIVRSLSDINLKIDDIALKQIMSKNKV-IDVRGPVFLNVEVKIE
---------------------------------

#Methanoculleus marisnigri
---------------MRFDVEEFKKRAR-EDFEHAWHEGPSVLTPAG-VSGRYPRLRYTR
--ATPHPIFEIVQRLRETYLAMGFDEAMNPLIVEESDIYRQFGPEAMAVLDRVFYLGGLP
RPNVGIARKQLDEIEAILGRA-------------------VSPGTEEKLRETLHGYKKGT
IDGDELTHELAAVLEADDAAVVHILD-AVFPEFRELAP--ESSRNTLRSHMTSGWFLTLS
--SLWEKRHLPIRLFSVDRCFRREQEEGPTRLMAYHSASCVVAGEDVTLEEGKAISEALL
SAFGFTEFRFQPDEK-RSKYYMPETQTEVYARHP-VLG----------WVEVATFGIYSP
SALAEYGIG--VPVMNLGLGVERLAMIAYQSNDIRQLTHPQFFP---QEISDREVAGAVH
LREEPRTVAGKRMAEAIRATAAEHATAPGPCAFTAWKGEIAG------------------
--REVEVIVEEPESNTKLCGPACANEVFVHDGSVLGVPDI---------EKWATVRQEGV
STGITYLDAVSSLAAARIEEAARCGE----------------------------------
----------EAHVQVKMSKLPSDVNLRIEEYAMRHITDHNKK-VDLRGPVFLTVRSVIP
EQPTR----------------------------

#Methanoregula formicica
---------------MRFNPEDWKKKAH-ENFEGAWHEGPSVLTPAS-HAETYPCKGYKR
--AQAHPVFATINKLRETYLSMGFDEAEVPVIIDEKDIYRQFGPEAMAVLDRVFYLGGLP
RPNVGIARDRLDKINGILGKT-------------------MAPAIEEKLRETLHAYKKSE
IDGDELTFELSKVLETDDGVVVHILD-DVFPEFRELAP--ESSRSTLRSHMTSGWFLTLG
--SIWDKSPLPIRMFSVDRCFRREQAEGPTRLMTYHSASCIIAGDDVTIEDGKAVSEALL
SAFGYTDFRFQPDEK-RSKYYMPDTQTEVYARHP-VHG----------WVEVATFGMYSP
SALAEYGVG--VPVMNLGLGVERLAMIAYGANDVRQLVYPQFFP---RPLSDREIARAVH
LREEPSSPEGKLLATAIAKVAAANGSAQGPCSFDAWEGTLGG------------------
--TRVKVVVEETESNAKLCGPACANEIFVHDGSVLGVPDA---------EKWKQVRIEGV
PIGLSYLSAVSALAAARIEEAARTGK----------------------------------
----------ETTVQVKMAKLPSDINLKIDEYAMRFVTDSKKK-VDVRGPVFLSVRSAIL
P--------------------------------

#Methanocorpusculum labreanum
---------------MKFDIEEFKERRK-TDFEGAWHAGPSVITPPE-SSKIYPRYAYRR
--AKVHPIFDTIARLRAAYMSMGFDEAMVPVFIDEQDVYRQFGPEAAAVLDRVYYVGGLP
RPNVGISRERLDAIAEIIEKP-------------------LAEGTEEKLMKCLHAYKKGK
FDGDDLTHEMSVALGVDDGVVVHILD-TVFPEFKELAP--ESSRTTLRSHMTSGWFISLA
--QMWDKKPMPIRMFSVDRCFRREQEEDATHLRTYHSASCIVAGEDVTVEEGKAVAEGLL
SAFGFTEFRFQPDEK-RSKYYMPETQTEVYGKHP-VHG----------WVEVATFGIYSP
AALAEYGVG--VPVMNLGMGVERLAMVLTQAEDVRKLSFAQLYP---PVYSDTDLTKGIG
LREEPQTAEGRRAVRAVMETAAAHAAERSPCSFPAWKGELYG------------------
--HQVEIVVEEPEENTSLLGPAALNEVYVRKGAVLGVPDT---------EKFADVKAEGV
PVGISFLYSTANLALARIEEAARVGE----------------------------------
----------GTSIQVKMSKHPSDVNLKIEEYVMRYITDNKKK-LDLRGPVFMTITSKIL
---------------------------------

#Methanosaeta thermophila
---------------MKFDPAEIREAAE-KDFDGTWKRGVDYLERPS-LERIYPRRSYPH
--GRPHPVFETIQRLREAYLRMGFTEMMNPVIVDAQEVHRQFGSEALAVLDRCFYLAGLP
RPDIGISESRLSEISSIVGKE-------------------LTAEEIEALREILHSYKKGA
VEGDDLVPEISKAINASDSQVSMMLE-SVFPEFRELRP--EPTSRTLRSHMTSGWFISLS
--NLWYRMPLPVRLFSVDRCFRREQSEDAARLMSYHSASCVVMDEDMSVDEGKAISEGLL
SQFGFENFRFRPDEK-RSKYYIPGTQIEVYAYHPGLVG--SETKYGSGWVEIATFGVYSP
TALAQYDIP--YPVLNLGLGVERLAMILYGSKDLRALSYPQLQP--DWSLKPIEIARMIH
VDKSPMTVTGKEIARSVVETCVKYGNTPSPCEFDAWEGELFG------------------
--RRIKVSVVEPEENTKLCGPAYLNEIVVYRNEILGIPRT---------PKWEAAFEEGV
QTGIRFIDAFAELAAHEIEMATIEGR----------------------------------
----------GSETRVRIVRTAGEINVRIDPALERYITSYKKR-IDIRGPVFTTVRSELM
QGDKHGEKT------------------------

#Methanosarcina mazei
---------------MKFDPEKIKKDAK-ENFDHAWNEGKKMVKTPT-LNERYPRTTLRY
--GKAHPVYDTIQKLREAYMRMGFEEMMNPLIVDEKEVHKQFGSEALAVLDRCFYLAGLP
RPNVGISDERIAQITEILGD--------------------IGEEGIDKIRKELHAYKKGK
IEGDDLVPEISAALEVSDALVADMID-RVFPEFKELIP--QASTKTLRSHMTSGWFISLG
--ALLEREEPPFQFFSIDRCFRREQQEDASRLMTYYSASCVIMDEDVTVDHGKAVSEGLL
SQFGFEKFLFRPDEK-RSKYYVPDTQTEVFAFHPKLVG--SNSKYSDGWIEVATFGIYSP
TALAEYDIP--YPVMNLGLGVERLAMILHDAPDVRSLTYPQIPQYSEWEMSDSGLAKQVF
VDRMPETPEGQEIASAVVAQCELHGEEPSPCEFPAWEGEVCG------------------
--RKVKVSVIEPEENTKLCGPAAFNEVVAYQGDILGIPNN---------KWQKAFENHSA
RAGIRFIEAFAAQAAREIEEAAMSGAD---------------------------------
----------EHIVRIRIVKVPSEVNLKIGATAQRYITGKNKK-IDIRGPVFTSVKAEFE
---------------------------------

#Archaeoglobus fulgidus
---------------MKFDPQKYRELAE-KDFEAAWKAGKEILAERS-PNELYPRVGFSF
--GKEHPLFATIQRLREAYLSIGFSEVVNPLIVEDVHVKKQFGREALAVLDRCFYLATLP
KPNVGISAEKIRQIEAITKR----------------------EVDSKPLQEIFHRYKKGE
IDGDDLSYLIAEVLDVDDITAVKILD-EVFPEFKELKP--ISSTLTLRSHMTTGWFITLS
--HIADKLPLPIKLFSIDRCFRREQGEDATRLYTYFSASCVLVDEELSVDDGKAVAEALL
RQFGFENFRFRKDEK-RSKYYIPDTQTEVFAFHPKLVG--SSTKYSDGWIEIATFGIYSP
TALAEYDIP--YPVMNLGLGVERLAMILYGYDDVRKMVYPQIHG--EIKLSDLDIAREIK
VKEVPQTAVGLKIAQSIVETAEKHASEPSPCSFLAFEGEMMG------------------
--RNVRVYVVEEEENTKLCGPAYANEVVVYKGDIYGIPKT---------KKWRSFFEEGV
PTGIRYIDGFAYYAARKVEEAAMREQE---------------------------------
----------EVKVKARIVENLSDINLYIHENVRRYILWKKGK-IDVRGPLFVTVKAEIE
---------------------------------

#Archaeoglobus veneficus
---------------MKFDARRYRELVE-EDFEDAWKRSVEVLSPKN-PNAVYPRYGYAY
--GEEHPLFKTINDLRKAYLSLGFREVVNPLIVEDSHVRRQFGKEALAVLDRCFYLASLP
KPNVGMSAEKIAKIGEIIGRS-------------------VGSEEVGSLQQIFHAYKKGK
IDGDDLSFEVAKALKIDDITAVRILD-EVFPEFKELKP--EASSLTLRSHMTTGWFITLS
--KVADKLPLPIKLFSIDRCFRKEQGEDATRLYTYFSASCVVVDEDVSVDEGKAVAEALL
RQFGFEKFRFKPDEK-RSKYYIPGTQTEVFAFHPKLVG--GNTKYKDGWIEIATFGIYSP
TALSQYDIE--YPVMNLGLGVERLAMILYDYSDVRELVYPWLYG--RIELSDMDIARAIK
VAEVPNTTAGLRMANAIAETAEKYANEQSPCEFLAWQGELYG------------------
--RSVKVYVVEREPSTKLCGPAYANEVVVYQGSVYGVPRT---------EKWSEYFEKGI
STGMRYIDSFACLAARQIEEGAARGNA---------------------------------
----------EVETRVRVVEGLSDVNLSMQDNVRRYIASKGGK-IDVRGPMFITVRAEID
---------------------------------

#Geoglobus acetivorans
---------------MKFNAEEWLEKAR-KDFESAWLNSEVVLSERP-LGERYPLRGYEE
--GRAHPVFDTIQKLREAYLRIGFSEVINPLIVEEVHVRKQFSKEALAVLDRCYYLAGLP
RPNVGISEERIRKIEEIIEK----------------------NFDPDELRKILHIYKKGK
IDGDDLSAEIARVLGVEDQIALKVID-EVFPEFKTLMP--EPTTLTLRSHMTTGWFITLS
--KLSGKYPLPVKLFSIDRCFRREQSEDPTRLYTYFSASCVIMAEDVSVDDGKAVAEALL
RHFGFEKFRFRKDDK-RSKYYIPGTQTEVFAYHPHL-E--NNRKYADGWIEIATFGIYSP
TALAEYGIE--YPVMNLGLGVERLAMVLHDYSDVREMVYGFRH----IELSDLDIARKIK
LKDVPLTPKGIEIAGRVIQAFEENAAAQGPCEFTAYRGRLFE------------------
--KDVEIFVFERE-NAKLCGPAYANEIVVHDGNIYGVPKN---------EKFRELFDEGV
STGIRYLDSFAYCAVRRIEEMIAEGRE---------------------------------
----------DGEVRSRVVENLSDINLKLEEGLMNYLISRGKS-VDVRGPLFLSVGFRLL
---------------------------------

#Methanoflorens stordalenmirensis
--------------MKKIPTSEILRIKE-KSFEEAWKYGGELLDDGSTYDLRRIEQAPSR
--GAPHALNDLIEKMRQRLLELGFDEVINETIFPEADIMKQWGPTGFAILDRCYYLAALP
RPDVGLSSQKQEMIKSFGIH--------------------LPPKKVAALQLTLHKLKTGE
LESDELIREIAKVLDVPDDRAIQVFE-EVFFEFKDLKP--LASNLTLRSHMTAAWFDTLA
--TVQKYTAVPVTLFSIGPRYRREQREDATHLRVHDSASCVVMDEDVSIDVGKWLVEEFL
RPFGFDDFQFRQVATGTDTYYAPGKHYEAFAYSPDFND---ARSNDAGWLEVADFGLYSP
LTLANYGIE--YPVVNCGIGVERIAMIAHAFKDIRTLVYPQFYG--EFVLTDDELASLIA
LKHTPATLQGEAIQQAIVSVCKTHGADASPVEFVAWEGELLD------------------
--NFVKITVIEPEENTKLCGPAFLNEVFVKDGNVIGAVKL-----------------EGL
STGITYIKAFAAEAAYEIEKALQQGKT---------------------------------
----------KLEVRVRISRSPNDLNLRIDPIATRYITSRNKK-IDLRGPIFTTVRMQAV
---------------------------------

#2140918025.a:b3_nosca_v_00254410
--------------MKKIPTSEILRIKE-KSFEEAWKYGGELLNDGSIYDLRRIEPTPSR
--GAPHVLNDLIEKMRQRLLELGFDEVINETIFPEADVMKQWGPTGFAILDRCYYLAALP
RPDVGLSAQKQAMIKSFGIH--------------------LPPKKVAALQLTLHKLKTGE
LESDELIREIAKELNVPDDRAIQVFE-EVFFEFKDLKP--LASNLTLRSHMTAAWFDTLA
--TVQKYTAVPVTLFSIGPRYRREQREDATHLRVHDSASCVVMDEDVSVDVGKWLIEEFL
RPFGFEDFQFRQVATGTDTYYAPGKHYEAFAYSPNFED---AKNNEAGWLEVADFGLYSP
LTLANYGIE--YPVVNCGIGVERIAMIAHAFKDIRTLVFPQFYG--EFVLTDDELARLIT
LKNAPATLQGEAIQQAIVNVCKTHGADASPVEFVAWEGELLD------------------
--KFARITVIEPEENTKLCGPAFLNEVFVKDGNVIGATKL-----------------EGL
STGITYIKAFAAEAAYEIEQALQQGKT---------------------------------
----------NLEVRVRISRSPNDLNLRIDPVATRYITSRNKK-IDLRGPVFTTVRMQTV
---------------------------------

#2067725009.a:draft_perm_00034430
--------------MKKIPIDEILEIKQ-KSFEAAWKYGGELLNDRSIYDLHRIADVASP
--GQPHALNELIERMRQRLLQLGFDEIINETIFPQEDVLKQWGPTGYAILDRCYYLAALP
RPDVGLSSQKQAAIRSFNIA--------------------LPPEKVAALQLTLHKLKTGE
LESDELIREIGKELDIADDCAVEVFE-KVFDEFKDLKP--IPSNLTLRSHMTAAWFDTLA
--TIQKNTHVPVKLFSIGPRYRREQREDATHLRVHDSASCVVMDEDVSIDLGKWILQEFL
RPFGFEDFQFRQVATGTDTYYAPGKHFEAFAFHPDFEE---VTNEDAGWLEVADFGLYSP
LTLANYGIE--FPVVNCGIGVERIAMLMHGYKDIRTLVYPQFYE--EFVLTDDQLINLIT
LKKTPTTPQGKEIQRAIINICKTHGADKSPVEFLAWEGELLD------------------
--KFVRISVAEPEENTKLCGPAFLNEVFVKDGSIIGAIKP-----------------KGV
STGITYIEAFAAEAAYAIEQALKMGKN---------------------------------
----------KLEVRVRIARSPNDINLNLDPVATRYITSHNKR-IDLRGPVFTTVRMQAA
---------------------------------

#Sulfidic spring Ya4
-----------------------------------------------------LIIESRK
--FERHPLSEVIEKLRHAYLDLGFKEVYNPIFIEEKEIYKQWGSEAPTILDRCFYLATLP
RPDIGISQDKLAYLKKIGFN--------------------FEQEKKLALQSIFHNYKKGV
LNGEDLIPKIAEVLSIDEDKASTILS--AFPELKQKRA--NATSLTLRSHMTSGWFLTLK
--EFRN-EKLPVKLFSIGLCFRREQTLDATHLRSYHSASSVIMNKELSLEDAKSVAEGLL
KPFGIEEIKYVKKKR-SASYYEKDTETEVFVRAKSERE----------WVEIIDFGIYSD
NVLEKYRIF--HPVLNLGLGVERLAMTIYGFSDIRKLVYPQFYA--KLELSDLEIASSIT
IDKTPRTEAGKRIQAAIIEACEKYGDNPSPFECIVYDGEILG------------------
--KRVIVKLFQSEEGKKLLGRAAFNEIMVYDGNILGIPKE--DNDIPKIELIKEARERGV
PVGIRYIDSLAAFAANSIEEAVESGRK---------------------------------
----------DFIMKKAMVSTSGELNIKVGEDALRFITSRNKR-IDLRGPVFMMMKAQIE
---------------------------------

#Hot spring Ya3
-----------------------------------------------------LAIKQRR
--TEKHPLSEVMDKLRHAYLDLGFKEVYNPIFIEEKEIYKQWGNEAPTILDRCFYLATLP
RPDIGISQDKLDLLKKIGFT--------------------FDREKKIALQKIFHDYKKGI
FDGEGLIPRLADALSIDGDKASTILS--AFPELKQKRA--IATNLTLRSHMTSGWFLTLK
--EFQD-GELPIKLFSIDVCFRREQALDATHLRCYHSASSVIMNEELSLEDAKSVAEGLL
KPFGIEEIKCVKKKR-SASYYEKDTETEVFVRIKSQRD----------WVEVADFGIYDN
AVLEKYGIK--HPVLNVGLGVERLTMAIYGFSDIRKLIYPQFYA--ELELSDSEIASSIT
IDKIPRTEQGKKIQAAIIKACEIYGDAPSPFEYTAYDGRILG------------------
--RKVTVKLIQNEEGKKLLGRAAFNEIVVYNGDILGIPKE--DKELSKSELIKDAREHGV
PTGIRYVDSLAAFVANGIEEAIESGNR---------------------------------
----------EFFMKRTMISTSGELNIKVREDALRFITSRNKR-IDLRGPVFMIIKAQIE
---------------------------------

#Arc I group archaeon U1lsi0528_Bin089
-----------------------------------------------------------M
--GKSHILEDTISLLRTIYLDLGFDEVFNPLFITDEDMYKQWGSETPAILDRVYYLAGLP
RPNVGLSKEVKDKISQIVEL---------------------DERRVRNLERTLQDYKRGN
IEGDDFVESISKGLGIEFGDAERILD--LFKEFKDLIP--VPTNLTLRSHMTSGWFITLQ
--ALAGRSELPLKLFSIDRCFRREQREDQTHLRSHFSASCVVMDKEISPELGKEIVTNFT
EKLGFDKVKFKVKKR-SASYYEAGTEHEAFIKLG---D----------WIEIADFGLYSK
EVLKKYKIP--YDVLNIGQGAERISMIRSGVNDIRELIYPQFY---KVEFSDQDIAKSIE
FVEDIKTLDGEKLLKLLLETARQNKDVQSPCEFTSYKGDFLG------------------
--RQIEVKIVEPEENTKLIGPAGFNQIYVLEKAMVGILPD------SKDENSLKIIKNGV
DTKVSYIEAFFRKVVSKIETTKEQG-----------------------------------
----------EYEERIPIVRSISDINLSLPTYIHQYLRGKGK--IDIRGPVFTTVRWKVL
---------------------------------

#Arc I group archaeon U1lsi0528_Bin055
-----------------------------------------------------------M
--GKPHVLEETISILRNIYLELDFDEVFNPLFITDDDMYRQWGSETPAILDRVYYLAGLP
RPNVGLSKEVKDKISQFVEL---------------------DERRSRNLERTLQDYKRGT
IEGDDFVEAISKGLGIEFEAAERVLD--LFKEFKELIP--VPTNLTLRSHMTSGWFITLQ
--ALAGRSELPLKLFSIDRCFRREQREDQTHLRSHFSASCVVMDKEISPELGKEIVTNFT
EKLGFDKVKFKVKKR-SASYYEAGTEHEAFIKLG---D----------WIEIADFGLYSK
EVLNKYKIP--YDVLNIGQGAERMSMIRSGVNDIRELIYPQFY---KVEFSDQDIAKSIE
FVQDIKTPEGEKLLMALLETARQNKDTQSPCEFTSYKGDFLG------------------
--RKIEVKIVEPEDNTKLIGPAGFNQIYVLEKTMIGILPD------SKDENSLNIIKNGV
DTKVSYLEAFFRKVISKIESTKEAG-----------------------------------
----------EYEERIPIARSISDINVSLPTYIHQYLRGKGK--IDIRGPVFTTVKWKIL
---------------------------------

#Arc I group archaeon ADurb1013_Bin02101
-----------------------------------------------------------M
--GKPHALEETISMLRHIYLDLDFDEVFNPLFITDEDMFKQWGSETPAILDRVYYLAGLP
RPNVGLSKEVKDKISQFVEL---------------------DERRSRNLERVLQDYKRGN
IEGDDFVESISKGLGIGFDSSEKILD--LFKEFKELIP--VPTNLTLRSHMTSGWFITLQ
--ALAGRSELPLKLFSIDRCFRREQREDQTHLRSHFSASCIVMDKELTPELGKEIVTNFA
ERLGFDKVKFIVKKR-SASYYEAGTEHEAFIKLG---D----------WIEIADFGLYSK
DVLNKYKIP--YDVLNIGQGAERITMIRNKINDIRELIYPQFY---KVDFSDQDIAKSVE
FIQNINTPDGEKLLKALLETAKQNKDAQSPCEFISYKGEFLG------------------
--KKIXVKIVEPENNTKLIGPAGFNQIYVYEKAXVGLLPD------SKDENSLNIIKNGV
DTNISYLEAFFRKVISKIESTKEYG-----------------------------------
----------EYEERIPIVRSISDINITLPTYIHQYLRGKGK--IDVRGPVFTTVRWNII
---------------------------------

#3300001687.a:WOR8_100124077
---------------MQFDIEKIKRESK-KDYEKAWLETKDLLKLSG----NYFTL-EKK
--GKSHVINDFIFDARKKLISLGFEELILPMFIEEEEIYKQYGPEAALILDRLFYLAGLP
RPDIGISNEKIDKILEMLPG----------------------FDKFDELKKIFRDYKKAE
IEADDLIESFVLKLEIEESKASEIID-KIFPELKELKP--IPTKTTLRSHTTALWFKILP
--ELIAKRDLPIQLFSVAAKFRREQKLDSTHLYCSNTLSLIVVTEEISLDDCKRIASTVC
KELGYENNRTVIKKA-TSKYYAPQTEFEIFVEHPSTKE----------WIEIGDGGFYSP
VSLAQYNVP--HPVFNIGFGIERICMIKSGIEDIRKLVYPYFYE--DVSFTDEEMTNGIR
YKYVPLNENAIKIKNAIVETALAHKDDQSPVEITVWEGDLKG------------------
--RKVRVKLWEKDEGVKLLGPAALNRIWIKDGSILGLPQE-------------QELEKGI
KTDLTYLEGIAEKAAFDIEVGLDQGQT---------------------------------
----------SQEYRIKMCYRASEVNIEIDDRILEYIHSRQKK-IDIRGPVFVGLSYEVF
D--------------------------------

#3300002053.a:SMTZ23_1000157232
---------------MPFDIEKINRESK-ANYEIAWLKTANLLKLKG----AYFSL-QKN
--GSSHIVNDFIFEARKKMISLGFEELILPMFVDEQDVYKQYGPEAVLILDRLFYLAGLP
RPDIGVSNEKLEMIREILPD----------------------FNKLDILKQIFRDYKKGD
IEADDLIETFLDRLNINESEASAIID-NIFPELKQLKP--IPTKMTLRSHTTALWFKVLE
--VLIQKKKLPLQYFTIGQKFRREQKLDSTHLYVSNTLSIVVMAEEISLEDCQNISSLIC
NEIGFPTNRVETKKA-TSKYYAPQTEFEIFVQHPITKE----------WIEIGDGGFYSP
VSLAKYNIT--YPVFNIGFGIERVCMIKSQINDIRKLVYPYYYE--DISFSDEEISKSIR
YKNVPFTENGINIKNAIINAAIKNKDLDAPIEIKVWEGIIKG------------------
--HNIQVSIWERDENVKLLGPAALNEIWIKDGNIFGVPRD-------------KVPENAI
SADKSYLEGIASEMAYNVEILLDQDIN---------------------------------
----------SYDHRVKMCYRASEVNLELDDIVLEFIHSKQKK-IDIRGPVFLGLSFNMI
D--------------------------------

#3300001782.a:WOR52_1001152511
---------------MPFDIKKINRESK-INYEMTWLETANLLKLKG----TYFNL-QKK
--GSSHIVNNFIFEARNKMVSLGFEELILPMFVDEQDVYKQYGPEAVLILDRLFYLAGLP
RPDIGVSKEKLEKILEIVPD----------------------FNRIDALKQIFRDYKKGD
IEADDLIETFIDKLDINESEASSIID-NIFPELKQLKP--IPTKMTLRSHTTALWFKVLE
--ILMKKKQLPLQYFTIGQKFRREQKLDSTHLFVSNTLSIVIMAEEISLDDCQKISSLIC
SELGFPSNRVEIKKA-TSKYYAPQTEFEIFVQHPTTNE----------WIEIGDGGFYSP
VSLAKYNIP--YPVFNIGFGIERICMIISQIDDIRKLIYPYYYE--DILFSDEEIAKSIR
YKNVPFTEKGIEIKNAIIKTAIENKDLDSPIEIKVWEGIIKD------------------
--HNIEVKMWEKDDNVKLLGPAALNEIWIKDGNVLGLPMD-------------NVPENAF
SANKSYLEGIAFEMAYNIEILLEQNIF---------------------------------
----------NYDHRVKMCYRASEVNLELDDVILEFIHSKQKK-IDIRGPVFLGLSFNVI
D--------------------------------

#3300002052.a:SMTZ1_100046492
---------------MKFNIDDLNQASK-EDYEKAWLESKNLLKMKG----DLYAL-KSQ
--GKSHPTKDFIQDARKFMIELGFEELILPMFVDEMDVYKEYGPEAALILDRTYYLAETP
RPDIGISAEKIERIKELIPD----------------------FDQTKSLQNIFRKYKKGE
IEADDLFETFIKELSISESQASEMVD-KIFPEFKQMQP--ISTKKTLRSHTTALWFKVLA
--QFQKKKDLPQQYFIIGPKFRREQKMDATHLYVSNTLSCVIIAEEISLTDCQKIIAQIC
EKIGFKNSKTEIKEA-TSKYYAPQTEMEIFVEHPTTKE----------WIEIGDGGFYSP
VSLAKFGIE--YPVFNVGFGVERIVMIREKNEDIRKLTYPYFYE--DIHFTDEEIANGIN
YEYSPATKVGVDIKEAIIKAAIENKDKKSPLEIVVWEGTLNN------------------
--KKIQVSIWETDEGVSLLGPAALNKIWVHDSSILGLPPD-------------KEIENAI
DTGKTYIDAIGSYMASMTEKLAFQDTN---------------------------------
----------RFDHRVKISYRASEINLEIDEMIMEFIHSRQKK-VDIRGPVFIGLSYKVI
EGN------------------------------

#WOR (Asgard-like) SepRS
--------------MGKFDLQKLKTEME-RDYEKAWLMSKSLLKEKG----RLLRL-KPQ
--GNPHPIFTFIERTRKTLLRLGFKETVLPIIVDEGTIFREYGPEACLILDRVFYLAGLP
RPDIGISKEKLEKIKKIIPE----------------------FENVSELQKIFREYKVGN
IEADNMLEVIVSELEVKEDDASKMID-SVFSELKRLEP--IPTKLTLRSHTTALWFPALA
--KIQYLQPRPIQLFSIGPKFRREQKLDRTHLYSSYTISFVIMADMT-LEDGKIVVEDFL
KELGYKEVIFAQKRA-TSKYYAPQTESEIFIEHPKTGE----------VIEIGDLGFYSP
VALANFGIE--YPVFNAGFGVERFVMVETGETDIRRLVYPYFYS--KDDLKDEELLKGIR
LTESPETDLGKEIMKAIITIAEEHKDDSSPTKVLAWQKHFKS------------------
--QLTQVFVWEPDPDVKLLGPAALNEIWVSEGNIIGVVPT------------EKLKKEGQ
STGIRYLDAVAAKAAKDIEDLLKSDRN---------------------------------
----------EYMLRVRMVKNAADINLTIDEPTRRMFTSKGKK-IDIRGPVFIGILLERK
SL-------------------------------

#archaeon Odin LCB_4
--------------MVKFDVNKILKEAE-EDYEKAWIQTAELIPKKS----KGFRL-EKN
--GVSHPVMDFIQKFRASFIELGFEEVILPTILPEEEVYKEYGPEAPIILDRIFYLAGLP
RADIGLSERKKEEILRIYPD----------------------FSKFNELQEIFRKYKKSE
IEADNLLDIMVKELTLPEEVASKIID-KVFHEFKELKP--IPSNLTLRSHMTALWFPVLK
--ELSKKRLPPIQLFTVGEKFRREQSLDSTHLYSSFTASCVILNENLSLNDGVSVAKTIL
NHIGFKEVKFEFKQT-TSKYYAPKTEFEIFIYHPHRND----------WVEVGDGGFYSP
VSLAKYEVD--TPVFNIGFGVERFVMVLQQEDDIRSLVYPYFYK--EIAFTDQDIAKSIF
LIEQPKTDLGIEIMNKIVNVCVENKDRESPVTITVDERVVNG------------------
--KKLKVTVWETDPGVKLLGPAALNEIFVLNGNIIGSLPN------------EELLSKAV
RTNITYLKATAALAAAAVEKLIEKTSG---------------------------------
----------EAVIRVRMSKQPSDVNIGIPENIRKFITANRRR-IDVRGPVFIGITASLE
---------------------------------

#BOG (Asgard) archaeon
--------------MVKFDINSLLDAAK-RNYEKAWNDSAKLLPADG----NQFTLDPTR
--GASHPIYDFIQEARTALISLGFEEIVLPIFVSEDEIFKEYGPEAALITDRIFYTAELP
RPDIGISQKKMDMMVQIVPG----------------------FEKFDELQKMFKAYKKCE
IEADDFLEAMVDRLEITQSEASSIID-KVFPEFKQLKP--VPTKNTLRSHTTALWFPVLG
--ALMGKKPMPIQLFHVGLKFRREQKLDARHLYESNTMSLVICIDEITLEDCMNVAKTIC
TNIGFLDAKFEIKKT-AGKYYAPGTEFEIFVKHENYGD----------WLEIGDGGFYSP
VSCAKYGIS--YPVFNIGFGVERITMIKTGAADIRHLAYPYFYA--PTSFTDKEMLPFIR
IANKPSTEAGQELVTRIIDAIKEHGSDSSPCEISVGVFDMES------------------
--VPIDITLWQNEAGVKLAGKAIYNEIVIKDGEITCYESS-----------ASDPPDGAE
ITGIKFVDGIANEIAWQVEQLARSDDA---------------------------------
----------ERTCQYKLVKKASDINVIIDPAVMRFVTDKQRK-VKINGPAFVNVTAKKA
---------------------------------

#3300002053.a:SMTZ23_100372094
----------------MWKPEKIKKKAQ-QDYEKMWLETTNLLKRTG----RKILWKKSR
--GRTHPVNELIQKIRSVFLSYGFEEVVNPSIVSEDDVRRQYGPEASVILDRIFYLAGLP
RPDIGLSREKTLEIQRIVSG--------------------FFEDKIKKLQKIFREYKEGK
IEGDNLTEEMVDRLKIETEHATAILS--LFPELTKLIP--IPSKLTLRSHMTALWFPVLA
--ALQDKKTLPMKLFSIGKKYRREQKLDERHLYSSFVASMVVMAEDITLEDGVELTRTIL
SEIGFKNEKFEVKKA-TSKYYAPKTEIEVFIKSN--NQ----------WIEVGDIGLYSP
VSLSRYNIR--YPVFNAGFGVERIAMLLQDITDIRKLVFPQFYI--EWKLSDADIAKSIK
IGVEPKSTIGVEIQKAIKSTALENMDKLSPCEFLAYEGKMLE------------------
--KMIKVYVYETDLEVKLLGPAAHNRIYVYNGNVLGIPER----GMDQVSIVRKARQKGI
SVGFSYLDAVASLAAKKIEEAVQIGVK---------------------------------
----------DVNIRVKMAEHPSDLNVKISDVARRYITSTKKK-IEITGPVFIGVRAEIT
G--------------------------------

#3300010324.a:Ga0129297_1000129512
--------------MPKLDPKEILRRVEEKAFEEVWVETARLLRLEG----RGLEWRRDK
--GKAHPIMEAIWRIRRIMLSYGFEEIINPSIVEEAEVYRQYGPEAPIILDRIFYLAGLP
RPDVGLDKRRIIEIRKIDPK--------------------FSNGKIKELQRIFRQYKEGE
IEGDNLVEEIVDRLNIGPEHATAILG--FFPELTSLTP--TPSKLTLRSHMTALWFSVLA
--ALQDKKPLPVKLFSVGAKFRREQKLDEMHLYESYVASTVVMSEDVTLQDGVELTRTIL
SELGFKDARFEVKKA-TSKYYAPKTEMEVFVKAG--DI----------WVEVGDIGLYSP
VSLARYGIR--YPVFNAGIGIERIVMLLEGVGDIRKIAYPQFYL--EVEYSDEQLGKMIR
IREAPTTEDGRRLLDAIVQTAIKHAIESSPCEFLAYRGPFLN------------------
--RKLEVYVYEPDAGTKLIGPAAFNVIYVYDGNVLGVPER----GLDNAKIVREAREKGL
AVGIRYLDAVASLAAARIEKEVRLGKK---------------------------------
---------GEFSVRIRMAKQPSDVNIEVNDVAQRYVTSERKV-IDVRGPVFVGVKAKIS
S--------------------------------

#3300010328.a:Ga0129298_100023706
--------------MGKLNPNKILKWVEREGFEKVWAQSASLLPPKK----KGAKPAPAK
-AGKTHPIFDLVQKFRQAFIKLGFNEILNPLVVDETEVYKQYGPEAAIILDRCYYLASLP
RPEIGLGLDKTEELAKLSIA--------------------LTPEKTEKLQAVLRRYKKGE
IDSDDLVQSLAESLGVSDADAIAVIE-KVFPEFAALKP--QPSTLILRSHMTSAWFNTLK
--SLQYKTELPIKLFSVDYKFRREQQEDATHLRSSYVASCVVMDEEVDVKDGEEITKAIL
QPLGFKRFKFVKKKV-TSRYYAPGTEYEGFVYFPAMKQ----------WVEVVDYGIYNP
IALAKYGIE--YPVLNVGIGVERVAMLLQGETDIRRLAYPYEYA--EWVMSDTQIASMVQ
IAKQPKTPQGEKLQKAIIKTAIKYAKKPSPCEFTAFKGKVFG------------------
--RTLEVSVYEPDVGTKLLGPAALNSVYVYDGNILGIPAK----GLEDKPLVKEARKKGI
STKIRYLDAVAAQAAAEIEQAARVGRP---------------------------------
---------KIISVRTKMAKLPSDVNIQISDVAQRYVTGKNRI-ISVKGPVFVGVKAKIV
---------------------------------

#Ca. Bathyarchaeota archaeon B63
--------------MSPLKPDEILDRVEKEGFEKVWRESSSLIPKPS----GGLDILSGR
-HGHPHLIYDLIQKLRRRFLSLGFDEVFNPVIIEENEIYRQYGPEAPIILDRCYYLAALP
RPDIGLSRSKCEEIERLGVK--------------------LTSSKISALKRVLREYKRGA
IEPDDLIERISDALSIPDDLATRITS-QVFPEFSALKP--EPSNMTLRSHMTSAWFLTLQ
--ALQHRVELPLKLFSIDLRFRREQREDPTHLRVHHSASCVVMNDELSVEDGERITRALL
EPMGFRNLRFLKKKV-TSKYYAPGMEYEGFIYNPRSKE----------WVEIVDYGIYSP
IALARYGLE--HPVLNVGLGVERLAMILHGESDVRRLMYPQFYK--KPTFTDREIAGAIR
FGMEPRTKEGERIRERIISEAIKHRDALGPCRFLVYEGEVLS------------------
--KKVKVYIYENEAGATLLGAAAENMIYVYNGNIVGAPLR----GMEEAPLIREAREKGV
CTGFRYIDGVASLAAARIEEGLKMGLR---------------------------------
----------EVDVRVRMARRLSDINLEVSDAVRRFITDHKKR-ISITGPVFLGVRAEII
G--------------------------------

#Ca. Bathyarchaeota archaeon B26-2
----------MGEVRENLNPEEILKRVEVEGFEKVWKESGNLLPRPP---EGYSLPLRGR
--GTSHPLFDLIERMRKAFLDQGFTEVANPVIVEDTEVYRQYGPEAPVILDRCYYLAVLP
RPDIGLSKAKCREIEGLGVE--------------------MTEERTSNLKTVLRDYKRGE
IEADDLIERVSESLEVPDATATLIIS-KVFSEFTSLRP--EPTNLTLRSHMTTAWFLSLQ
--ALQHRVEMPIKLFSVGIRFRREQKEDPTHLRVHHAASCVVMDEAVDVREGEKITRNLL
EPLGFKEFRFVRKKV-TSKYYTPGMEYEGYIYHPKMRR----------WIEVANFGLYNP
IALARYGLE--HPVLNVGVGVERVALAFYGEEDVRRLVYPQFYG--EWRLTDVEIAGMIG
YEIEPKTREGHEIKRRIVEEALAHADDPSPCEVLAYEGRLLG------------------
--KVVRVYLYEKERGAKLLGAAARNVIHVYEGNVLGIPAK----GMDHIPIIREARERGV
STNLTYIEGIAALAAAKIEEAAEVGRN---------------------------------
----------QVDVRVRIVRRPRDVNIKISNVARRYITGRKRR-IDISGPVFVGIRAEIM
NQHV-----------------------------

#archaeon V1
---------------MRFNIKEVLEKAD-RDFEGAWAETGKLVGGRG-----AFKQ-GRK
--GSAHLLSETANRLREAYLELGFDEVVNPMIVDEADLYKQYGNEAPAILDRCYYLATLP
RPDIGIGAEKVEAMKRAGAP--------------------TDEAALGRIRDVFHDYKKGR
IDGDDLVERLTDGMGVPDTLALKVMN-EVFPELRRLEA--VPTKLLLRSHMTSAWFLTCL
--AVQHKLEMPVKLFCVGLRARREQQEDASHLRFHNAASSVIMDDEVSVDDGRAVAELVL
KRFGFQEVRTEQKKV-TAKYYAPGTEYEVFARSEK-AG----------WVELMDYGLYSP
VALARYGIE--RPVLNIGMGVERIAMLLNGYSDIREMAYPQFYG--EWIISDAALARQVS
FIEEPKTGEGREVERAIVSKVEERRDADSPCEFVAFEGMAGG------------------
--RRVRVKVFEPDPKVKLVGPAAFNELVVYDANILGIPEK----GMDGVELIRNAKTKGI
RTGIRYLDAVAKMAAARVERDG--------------------------------------
----------KMELRTRMAKLPSDINIRVSEVGVRYVSSRHGK-IDVRGPAFVGVYSEVD
E--------------------------------

#archaeon V3
---------------MRFNIKEVLEKAD-RDFEAAWAETGRLVGGRG-----AFQPNQGR
-RGAAHVLSETANRLREAYLELGFDEVVNPMTVDEADLYKQYGNEAPAILDRCYYLATRP
RPDIGIGAEKVEEMRRLGAP--------------------TDEEALARVRGVFHDYKKGK
VDGDDLVERLTEGMGVPDTLALKVMN-EVFPELRRVEA--QPTKMLLRSHITSAWFQTCL
--AVQHKLEMPVKLFCVGLRARREQQEDASHLRFHNAASSVIMDDEVSVDDGSAVAELVL
RRLGFQEVRTERKRV-TAKYYAPGTEHEVFARSEK-AG----------WVELMDFGLYSP
VALARYGIE--HPVLNIGMGVERIAMLLNGYSDIREMAYPQFYG--EWILSDAALARQIS
FIEEPGTAEGREVERAIVRKVEEMRGAGSPCEFVAFEGRVGG------------------
--RAVRVKVFEPDPNVKLVGPAAFNELVVYDGNILGIPEE----GMEGVELIRNARSKGI
RTGVRYLDAVAKMAAARVERDG--------------------------------------
----------RMEVRTRMAKLPSDINIRVSDVGVRYVSSRHGK-IDVRGPAFVGVYSEVV
KE-------------------------------

#Hot_spring_Ya1
---------------MKIRIKEVLEKAE-KDFEEAWRETGSLVGGKG-----AFAP-GKK
--GTKHVLIETANRLREIYLELGFDEAVNPMIVDEGDIYKQYGHEAPAILDRCYYLAALP
RPDIGIGSERIAKIREAGGP--------------------TDEASLSRLKELFHDYKKGK
VDGDDLVEKVAEGLGVPDTTALRILN-DVFPELRMLEP--VPTKLLLRSHMTSAWFLTCA
--AVQHKLEMPVMLFSVGLRARREQQEDATHLRFHHAASSIIMDEEVSVEDGKRVSEEVI
KRLGFGEVRVERKKV-TAKYYAPGTEHEVFAKVGG-TG----------WVELMDFGLYSP
VALAHYGIE--YPVLNIGIGVERVAMLLHGYKDVRELAYPQFYG--EWVLSDASLARQLS
FIEEPFTEEGKAVERAIVEAIEGHKDAESPCEFMAYEGKVSG------------------
--REVRVRVYEPDPGVRLVGPAAFNELVVYRANILGIPPQ----GMENVELVRAARSAGV
RTGIRYVDAVAKMAASRIERGG--------------------------------------
----------QADIRVRMVKLLSDINLKVSEVGLRYISSRQGK-IDVRGPAFIGVKSELV
IDQS-----------------------------

#Wastewater_b1
---------------MRLNTKEILEKAD-ADFEKAWRETGSLLTGKG-----ITEREGKK
--GTRHVLVETMNLLREIYLDLGFDEVVNPMIVEEADIYKQYGHEAPAILDRCYYLATIP
RPDIGISAEKIQKIKELGGP--------------------TDEEALGKLMEIFHDYKKGR
VEGDDLVEKISDSLGISDCLVLRILD-TVFPEFRKLEA--SPSKLLLRSHMTSAWFLTLE
--AVQHKLEKPVKLFSVGLRARREQQEDATHLRFHHAASSVIMDEDVSIEDGKRVAGAVA
KRLGFGEVRFERKKV-TAKYYAPETEYEVFAKTAN-GG----------WVEIMDFGMYSP
IALARYGIE--FPVLNIGMGVERIAALLHKFADIRELSYPQFYG--EWLLSDADLARQVG
FIEAPLTAEGKRVEEAIVRAIEEHRDAESPCEFIAYEGSVCG------------------
--KKVTVKVFEHDPKVRLVGPAAFNQIVVYDANIIGIPEK--AAGMEKSEIVREAREKGV
RTGIRYLDAVAKMAAARIERGG--------------------------------------
----------SAELRVRMAKLPSDINLRVSDVGMAFVTGRNGR-IDVRGPVFIGVSSEAE
AETAGIAMAEPAGTAEAAAHGGARDIRSEEG--

#Hot spring Ya2
---------------MRINIKEILERAE-RDFEGCWRETGSLVGGKG-----AYRA-GRK
--GSPHVLMETINRLREAYLDLGFDEVVNPMIVDEADIYKQYGSEAPAILDRCYYLATLP
RPDVGIGSKEIEAIRRAGGP--------------------VDQQSLERLRVTLHDYKKGR
IDGDDLVEKISEALNVEDTVALKVLN-EAFPQFSRLEP--IPTKQLLRSHMTSAWFLTCA
--AVQHKLERPIMLFSVGLRARREQQEDAKHLKFHHAASSVIMDEEVSVEDGKVVAGEIL
KRLGFKDVRFERKKI-TAKYYAPGTEYEVFARLGG-SD----------WFEVVDFGLYSP
IALARYGIE--FPVLNIGIGVERVAALLYGYNDVRELSYPQFYG--EWILSDAALARQIR
FLEEPVTEEGRALERAIVRAIEENRDAKSPCEFLAYEGIVGG------------------
--RRVVVKVFEPDPNVKLVGPAAFNEVVVYNANILGVPER----GMEKVSLVEEAREKGV
RTGIRYVDAIAKAAAARVEREG--------------------------------------
----------EVEMRVRMAKLLSDINLRLTDVGMRYITGKGGK-IDVRGPVFVGVYSRVV
P--------------------------------

#Sulfidic spring Ya2
---------------MRINIKEILERAE-RDFEGCWRETGSLVGGKG-----AYRA-GRK
--GSPHVLMETINRLREAYLDLGFDEVVNPMIVDEADIYKQYGSEAPAILDRCYYLATLP
RPDVGIGSKEIEAIRRAGGP--------------------VDQQSLERLRVTLHDYKKGR
IDGDDLVEKISEAL-VEDTVALKVLN-EAFPQFSRLEP--IPTKQLLRSHMTSAWFLTCA
--AVQHKLERPIMLFSVGLRARREQQEDAKHLKFHHAASSVIMDEEVSVEDGKVVAGEIL
KRLGFKDVRFERKKI-TAKYYAPGTEYEVFARLGG-SD----------WFEVVDFGLYSP
IALARYGIE--FPVLNIGIGVERVAALLYGYNDVRELSYPQFYG--EWILSDAALARQIR
FLEEPVTEEGRALERAIVRAIEENRDAKSPCEFLAYEGIVGG------------------
--RRVVVKVFEPDPNVKLVGPAAFNEVVVYNANILGVPER----GMEKVSLVEEAREKGV
RTGIRYVDAIAKAAAARVEREG--------------------------------------
----------EVEMRVRMAKLLSDINLRLTDVGMRYITSKGGK-IDVRGPVFVGVYSRVA
P--------------------------------

#Hot spring Na1
---------------MKLNIKEILKRAD-EDFEKAWKEL--IIEGKG-----VFKI-RRK
--GNPHIIMETINKLREIYLDLGFDEVINPMIVDEVDIYKQYGREAPAILDRCYYLATLP
RPDIGIGKNEIEKLKEIGVP--------------------VDEENIKKLKNILHDYKKGR
IDGDDLVEKISEELNIEDTLALKILN-DVFPQFKKLEP--IPSRQLLRSHMTSAWFLTCA
--AIQYKLEKPIMLFSIGLRARREQKEDEKHLRFHHVASSIIMDEEVSVEDGKIICNEIL
KRLGFNEIKFEKKKI-TAKYYAPGTEYEVFAKLGN-SD----------WFEVIDFGLYSP
IALARYEIE--YPVLNIGIGVERIAALLYGYKDLRELSYPQFYG--EWILSDNSLVKHIR
FIEEPKTKEGIIIEKAIIKTIEENRNANSPCEFIAYEGKILG------------------
--KNIIVKVFERDPNVKLVGPAAFNEVYIYNGNIIGIPEK----GLEDIEIIKEAREKGI
RTRIRYLDAIAKAAAARIEKYG--------------------------------------
----------EIEMRVRMVKQLSDINLSLSDIALRYITSKRGR-IDIRGPVFIGIYSKYV
---------------------------------

#Sulfidic spring Ya3
---------------MKINIKEILKKAD-EDFEKAWKEL--VIEGRG-----AFKI-RRK
--GNPHLIMETINKLREIYLDLGFDEVINPMIVDEVDIYKQYGREAPAILDRCYYLATLP
RPDIGIGKDEIEKLKEIGAP--------------------IDEESIKKLKNILHDYKKGK
IDGDDLVEKISEGLNIEDTLALKILN-DVFPQFKKLEP--IPSRQLLRSHMTSAWFLTCA
--AVQYKLEKPIMLFSIGLRARREQKEDEKHLRFHHVASSIIMDEEVSIEDGKIVCNEIL
KRLGFSEIKFERKKV-TAKYYAPGTEYEVFAKLGS-SD----------WFEVIDFGLYSP
IALARYEIE--YPVLNIGIGVERIAALLYGYNDVRELSYPQFYG--EWILSDDSLVKHIK
FIEEPKTKEGILIEKAIIKTIEENKDANSPCEFIAYEGKILG------------------
--KNIVVKVFEKDPNVKLVGPAAFNEVYVYNGNIVGVPEK----GLEEIEIIKEARKKGI
RTGIRYLDAIAKAAAARIEKYG--------------------------------------
----------EIEMRVKMVKQLSDINLSLSDVALRYITSKRGR-IDIRGPVFIGIYSKYV
---------------------------------

#Sulfidic spring Ya1
--------------MVRLPVKDILAKSK-ENFEEAWLAYGKLIPTKR---LKPKDI-LSC
GVGKPHPVYEVCQRLRQAFLSLGFEEVVNPLIVEEEDVKKQYGPEALAILDRCYYLAVLP
RPDVGLSKERVEALKKYVAD--------------------LTPDKVQRLQQVLHSYKKGH
IAGDDLIEEMSKALDVEDTMATRILW-DVFPEFRDLKP--EPTRLTLRSHMTTAWFLTVA
--ALQHKRPKPIKLFSVDVRVRREQYEDETHLRTHRAASCVVVDEDVVPDDGRDIAQAVL
EGLGFKEFKVVRKPV-TAKYYAPGTEYELYVKSYG--D----------WIEVANYGIYSP
VALANYEIE--YPVLNVGIGVERVAMILYSYKDVRELVYPQFYG--KWSLSDCEIAKQIS
LQHKPETQAGWRLVEAIVKAFEQYAEAPSPCEFKVYEGELLG------------------
--RRVEVYVYERDPGVKLVGPAAFNEIVVYEGNVIGIPPS--HVQSIVSPLIEEARSRGC
KTGIRYVDAFAALAAARIEAACMVGAD---------------------------------
----------EVDVRVRMVKLPSDINVEIGEVARRYINENRKV-IDVRGPAFLAVRARTS
EK-------------------------------

#3300009598.a:Ga0105154_10020794
--------------MVRLPVKDILAKSK-ENFEEAWIAYGKLIPTKR---LKPKDI-LGC
GIGKPHPVYEVCEKLRQAFLNLGFEEIVNPLIVEEEDVKKQYGPEALAILDRCYYLAVLP
RPDVGLSKERVEELKKYVAD--------------------LTLDKVQRLQQVLHGYKKGQ
IASDDLIEEMSKALGVEDTVATRILR-EVFPEFRDLRP--EPTNLTLRSHMTTSWFLTVA
--ALQHKRPKPIKLFSVDVRIRREQREDETHLRAHRAASCVVVDEDVVPDDGRDIAQAVL
ESLGFKEFKEVRKPV-TAKYYAPGTEYELYVRFNG--D----------WIEVANYGIYSP
VALANYEIE--YPVLNVGIGVERVAMALYGYKDVRELVYPQFYG--KWLLSDCEIAKQIS
LQHKPETQVGWQLVEAIVKAFEQYADAPSPCEFKVYDGELFG------------------
--RHVEVSVYERDPGVKLAGPAAFNEIVVYDGNVIGIPPS--HVQAITSPLIEEARLRGY
RTGIRYVDAFAALAAAKIEAACLAGAS---------------------------------
----------EVDIRVRMVKLPSDINIEVAEVARRYISENRKT-IDVRGPAFLAVKARMS
EK-------------------------------

#3300009598.a:Ga0105154_10022636
--------------MVRLPVKDILAKSK-ESFEEAWIAYGELIPTKR---LKPKDI-LSC
GVGKPHPVYEVCQRLRQAFLNLGFEEVVNPLIVEEEDVKKQYGPEALAILDRCYYLAVLP
RPDVGLSKEKIEKLKRYVAD--------------------LTSDKVQQLQQVLHGYKKGQ
IASDDLIEEMSKALGVEDTVATRILW-DVFPEFRDLRP--EPTRLTLRSHMTTAWFLTVA
--ALQHRRPKPIKLFSVDVRVRREQYEDETHLRTHRAASCVVVDEDVVPDDGRDIAQAVL
ESLGFKEFKVVKKPV-TAKYYAPGTEYELHVRSGG--D----------WVEVANYGIYSP
VALANYEIE--YPVLNVGIGVERVAMVLYGYKDVRELVYPQFYG--KWFLSDYEIAKQIS
LQHKPETQAGWQLVETLVRAFEQYAEAPSPCEFKVYEGDFLG------------------
--RYVEVYVYEKDPGVKLAGPAAFNEIVVYEGNVIGMPPS--QVQAITSPLLEEARLKGY
RTGIRYVDAFAALAAAKMEAACLVGAG---------------------------------
----------EVDVRVRIVKLPSDINIEIGEVAKRYISENRKV-IDVRGPAFLAVKAKIS
EK-------------------------------

#SSWTFF a1
--------------MAKLPVKEILEESK-KNFEEAWINYGKLIPVKR---LKPKDI-LGC
GIGKPHPVYEVCQRLRQAFLNLGFEEVVNPLIVEEEDVKKQYGPEALAILDRCYYLAVLP
RPDVGLSKEKVEMLKRYVAD--------------------LTPDKVQQLQQVLHGYKKGQ
IAGDDLIEEMSKALGVEDTVAMRILW-DIFPEFKDLRP--EPTRLTLRSHMTTAWFLTVA
--ALQHKRPKPIKLFSVDVRVRREQYEDETHLRTHRAASCVVVDEDVVPSDGEEIAQAVL
ESLGFKEFKAVKKPV-TAKYYAPGTEYELYVKFGG--D----------WIEVANYGIYSP
VALANYEIE--YPVLNVGIGVERVAMVIYGYKDVRELVYPQFYG--RWLLGDHEIAKQIS
LQHKPETQAGWQLAEAIAKAFERYAEAPSPCEFKVYEGSLLG------------------
--KHVEVYVYERDPGVKLAGPAAFNEIVVYEGNVIGIPPL--QVQSITSPLIEEARLKGF
KTGIRYVDAFAALAAARIEATCLAGAG---------------------------------
----------DVDVRVRIAKLPSDINIEIGEVARRYINENRKV-IDVRGPVFLAVRARVS
ER-------------------------------

#3300009598.a:Ga0105154_10008626
--------------MVKLPIKEILEESK-KNFEEAWINYGKLIPVKR---LKPKDI-LGC
GIGKPHPVYEVCQRLRQAFLNLGFEEVVNPLIVEEEDVKKQYGPEALAILDRCYYLAVLP
RPDVGLSKERIEVLKKYVAD--------------------LTSDKVQRLQQVLHGYKKGH
IASDDLIEEMSKALEVEDTVATRILW-EAFPEFKDLRP--EPTKLTLRSHMTTAWFLTVA
--ALQHKRSKPIKLFSVDVRVRREQYEDETHLRTHRAASCVVVDEDVVPDDGRDIAQAVL
ESLGFKEFKVVKKPV-TAKYYAPGTEYELYVKSDG--D----------WIEVANYGIYSP
VALANYEIE--YPVLNVGIGVERVAMVLYGYKDVRELVYPQFYG--KWFLSDYEIAKQIS
LQHKPETPAGWHLVETIVKAFEQYAEAPSPCEFKVYEGDLLG------------------
--RHVEVYVYERDPGVKLAGPAAFNEIVVYEGNVIGLPPL--QAQSITSPLIEEARLRGY
RTGIRYVDAFAALAAAKIEAACLAGAG---------------------------------
----------EVDVRVRIVKLPSDINVEIGEVARRYISENRKV-IDVRGPAFLAVRARMT
ER-------------------------------

#3300008019.a:Ga0105158_100012321
--------------MVRLPVKDILARSK-ENFEEAWITYGKLIPTKR---LKPKDI-LSC
GVGKPHPVYEVCQRLRQAFLNLGFEEIVNPLIVEEEDVKKQYGPEALAILDRCYYLAVLP
RPDVGLSKEKVEMLKRYVVD--------------------LTPEKVQQLQQVLHDYKKGH
IAGDDLIEEMSKALNVEDTVATKILW-EVFPEFRDLKP--EPTRLTLRSHMTTAWFLTVA
--ALQHKRPKPIKLFSVDVRVRREQYEDETHLRTHRAASCVVVDEDVVPDDGRDIARAVL
ESLGFKEFKAIKKPV-AAKYYAPGTEYELYVRSGG--D----------WIEVANYGIYSP
VALANYEIE--YPVLNVGIGVERVAMVLYGYKDVRELVYPQFYG--KWFLSDYEIAKQIS
LQRKPETQAGWQLVETIVKAFEQYAEAPSPCEFKVYDGEFLG------------------
--RHVELYVYERDPGVKLAGPAAFNEIVVYEGNVIGIPPS--RAQSITSPLIEEARLKGH
RTGIRYVDAFAALVAARVEAACLAGVS---------------------------------
----------EVDVRVRMVKLPSDINIEIGEVARRYINENRKV-IDVRGPAFLAVRVRVL
EG-------------------------------

#pJP 33 archaeon JGI MDM2 LHC4sed-1-M18
--------------MVKLPIKEILEESK-KNFEEAWINYGKLIPVKR---LKPKDI-LSC
GIGKPHPVYEVCQRLRQAFLNLGFEEVVNPLIVEEEDVKKQYGPEALAILDRCYYLAVLP
RPDVGLSKERIEALKKYVAD--------------------LTSDKVQRLQQVLHSYKKGH
IASDDLIEEMSKALEVEDTVATKILW-EVFPEFRDLRP--EPTKLTLRSHMTTAWFLTVA
--ALQHKRPKPIKLFSVDVRVRREQYEDETHLRTHRAASCVVVDEDVVPDDGKDIAQAVL
ESLGFKEFKVVKKPV-TAKYYAPGTEYELYVKSDG--D----------WIEVANYGIYSP
VALANYEIE--YPVLNVGIGVERVAMVLYGYKDVREMVYPQFYG--KWFLSDHEIAKQIS
LQHKPATPAGWYLVETIVKAFEQYAEVPSPCEFKVYEGDLLG------------------
--RHVEVYVYERDPGVKLAGPAAFNEIVVYEGNVIGLPPL--QAQSITSPLIEEARLRGY
RTGIRYVDAFATLAAARIEAACLAGVG---------------------------------
----------EVDVRVRIVKLPSDINVEIGEVARRYISENRKV-IDVRGPAFLAVRARMA
ER-------------------------------

#Hot spring Ja1
-------------------------------------------------------M-FKP
--GHPHPVFELTQKLRELFLRFGFDEVINTYIIEDTDMYKQYGAEAPAILDRCYYLAVIP
RPDIGISKEKIDKLKNIVPT--------------------VTTKEINGLKEVFHSYKKGK
ISSDDLVGEIASILNVNEAMALEVFD-NVFPEFKELKP--EPTKLILRSHMTSSWFHTIS
--ALQYKLPLPIKLFSVDIRFRREQREDEKHLSTHRVASCVIVDEALSIDEALSFTKAFL
KELGFIDVYFIKKSS-TPAYYAPNSNYEVFIKHKG--N----------DIEVAELGLYNP
ISLAKYDIV--YPVINIGFGVERIAMVIYNEQDIRRLVYPQFYG--IWVLTDEDIASTIS
LDKIPSTVDSWLIFDRLIETSKDNKDTQSPCTIRAYEGPFMK------------------
--KHIIIEFYENDPSVKLLGPAAFNRIYVYKGNILAIPEE----GFENIEYINEARTNGV
KTNIRFIDAAICLFLYQLEQAILIGAE---------------------------------
----------EVNLRIKMVKQPSDINIRIPQNIYHYITSRNLK-IDIKGPAFIGIYARIM
K--------------------------------

#Hot spring Ja2
-------------------------------------------------------M-FKP
--GHPHPVFELTQKLRELFLRFGFDEVINTYIIEDTDMYKQYGAEAPAILDRCYYLAVIP
RPDIGISKEKIDKLKNIVPT--------------------VTTKEINGLKEVFHSYKKGK
ISSDDLVGEIASILNVNEAMALEVFD-NVFPEFKELKP--EPTKLILRSHMTSSWFHTIS
--ALQYKLPLPIKLFSVDIRFRREQREDEKHLSTHRVASCVIVDEALSIDEALSFTKAFL
KELGFIDVYFIKKSS-TPAYYAPNSNYEVFIKHKG--N----------DIEVAELGLYNP
ISLAKYDIV--YPVINIGFGVERIAMVIYNEQDIRRLVYPQFYG--IWVLTDEDIASTIS
LDKIPSTVDSWLIFDRLIETSKDNKDTQSPCTIRAYEGPFMK------------------
--KHIIIEFYENDPSVKLLGPAAFNRIYVYKGNILAIPEE----GFENIEYINEARTNGV
KTNIRFIDAAICLFLYQLEQAILIGAE---------------------------------
----------EVNLRIKMVKQPSDINIRIPQNIYHYITSRNLK-IDIKGPAFIGIYARIM
K--------------------------------

#pSL50 archaeon JGI MDM2 LHC4sed-1-M8
------------------------------------------------------------
-----------------LFLRFGFNEVINPYIIEDTDMYKQYGAEAPAILDRCYYLAVLP
RPDVGMSKEKIDKLKSIVPL--------------------VAMKEIDNLKKVLHNYKKGK
ISSDDLVGEIAVALNVDEVTALKVFD-YVFPEFKELKP--APTNLILRSHMTSSWFHTIS
--ALQYKLPLPIKLFSVDIRFRREQREDEKHLSIHRVASCVMVDEVLSIEEALLFTKAFL
KELGFTDIYFIKKSQ-TSSYYAFNSNYEVFIKHQG--N----------NVEVAELGLYNP
VVLNKYGIA--YPVINIGFGVERIAMIMYNEQDIRNLVYPQFYG--IWMLTDEDIASAIS
LDKIPNTVDSWLIFEKLVETSKANKDVQSPCTIKAYEGSFMN------------------
--RHVTIEFYENDPNVKLLGPAVFNRIYVYKGNILAIPEE----GFEHVEYVTEARANGV
RTNIRLIDAAICLFLYQLEQAILIGAE---------------------------------
----------EVNLRIKMVKQPSDINIKIPQNIHHYITSKNLK-IDIRGPVFVGIHARIT
K--------------------------------

#Hadesarchaea archaeon DG-33
--------------MTKFNIRKLRASAK-KDYESAWLESAKLVEKRG----KLFSL-KDK
--TKTHPLFELIERARRVLLELGFAETVLPMIVDKKEVYAQYGPEAPVILDRVFFLAGLE
RPDIGISQKKIQEIRELVPD----------------------FKEIEKLQGIFRRYKRGE
IVADDLVEVMVKELDIREEQATGLLA--LFGELRELKP--VPMDLTLRSHTTAGWFLVLR
--ELQRRESLPLQLFSIGPKFRREQRLDPTHLYESWTASMVIAAEKISLEDGERITRKIL
DKLGFSEVKFSIKTA-TSKYYAPQMEFEIFVKHPKTGE----------FIEVGDAGFYSP
VALSHYDIA--FPVFNLGIGLERVLMIETGGTDIRVLMYPYLYK--AAEFSDRELADMIK
CEREPGTEIGRAIAAAIVKAAQQHADEPSPCDFKAFEGKLGG------------------
--KRVIVRVVEPERGTKLIGPAGFNEIYVYEGNVVGVPPK----GWEKDEFLNSVREKGV
STGISYIIAFAALAAQEIERAAKSGKK---------------------------------
----------QVKVRVRAAKLPSDINLVIDEAAQRYITANKKR-IDVRGPVFTTVVAEFG
---------------------------------

#Hadesarchaea archaeon _YNP_45
---------------MKFDVREIRARAE-KDYERAWLESAELIEKKG----RFFNL-QNK
--ARPHPLQELIAEVRRALLDLGFTEVVLPILVDRREVQAQYGPEAAVILDRVFFLAGLE
RPDIGISRKKFQEIKRIVPD----------------------FADLKKIQGIFRRYKKGE
ISADDLVEVMVQELGLKEEQATAILS--LFPEFKELKP--VPMDLTLRSHTTAAWFGALR
--ELQHREPLPLQLFSIGPKFRREQRLDESHLYESWTASLVIMAERMSLEDGDEVIRRVF
SKLGFDEVRIVRKKT-TSKYYAPQTESEIFVRHPATGE----------YLEVGDAGFYSP
ISLSNYDIA--YPVFNLGIGLERMLMIRTGEKDIRALVFPYLYR--PPEFSDEELAAAIK
MQREPRTEAGRMIAAAIVRTAQQHADEPSPCEFLAYEGEVAG------------------
--RRVIVKVVEPESNTKLIGPAGFNEIYVFEGNVIGVPPK----GWEQDEFLNNVRRRGR
ATGIRYLDAFAALAADEIERAAERGER---------------------------------
----------EVKIRVRAVKSLADINLRLDEAAQRYITSNKKK-IDVRGPVFTTVVAEFP
P--------------------------------

#Hadesarchaea archaeon _DG-33-1
--------------MAKLDIRKIRANAA-KDYERAWLETAELVEKRG----QLLNL-VDR
--RRPHALFELILKIRRALLELGFEEVVVPTIVERGEVFNQYGPESPVILDRIFFLAGLD
RPDIGLSKRRMQQIQKIIPK----------------------FKDFKHLQDIFRRYKRGK
ISSDDFVETMVRELMIGESQATALIS--LFKELKELKP--VPTSLTLRSHISAGWFSLLR
--EMVKREPLPIQMFSIGSKFRREQQLDATHLYDSWTASLVVVAERMSLEDGKRLVKQLL
SKLGFENVKLVVKKG-TSKYYAPRTEFEVFIRHLKMGE----------YVEVGDAGFYSP
VPLSKYDIP--YHVFNFGMGIERLLMVMTGETDIRALVYPYLYK--PTVLSDTKLAGMLK
FERAPESEVGKEIAQAIVRIAEQRADEPSPCEFKAFVGRVSG------------------
--RRVSVSVVEPESGTKLIGPAGFNTIYVHDGNFIGVPPN----GWEKDRFLNTVREWGV
STGIRYMDAFAALAAHEIEQAARRGRR---------------------------------
----------KVKVRVRAVKLLSDINLVLDKAAQRYITDKKKR-IDVRGPVFTTIIADIS
KTRATS---------------------------

#Hadesarchaea archaeon _YNP_N21
--------------MGKFDIRRLKEEAE-RDFEGAWLKSGELIKREG----KFFEL-ADK
--RAEHPLFGLIIKVREILLGLGFTEVVVPTIIEKSEVFKQFGPEAPVILDRVFFLAGLD
RPDIGIDAKRIEQIRRVVPN----------------------FAKVKELQSIFRRYKRAE
ISSDDLLETMVKELGVDEAQASSIIS--IFSELRDLRP--IPLDLTLRSHTTAGWFSVLA
--EMQRRQPLPLQLFSIGPKFRREQRLDPTHLYDSWTASIAIMAEEISLEDGKRIAGEIL
RRLGFDDFKMEIKTA-TSKYYAPQTEFEIFLRHPKTGK----------YLEVGDAGFYSP
VSLAKYDIA--NPVFNLGLGIERILMIKMGEEDIREVIYPYFYK--PPTFTDLEISKAIK
FERAPQTEVGKEIASAIVRVAERRADEPSPCDFEAFRGELLG------------------
--KHVKVSLVEPESGTKLVGPAGFNMVYVYDGNIIGVPPK----GWERDEFLQKVKASGK
STGIRYIDAFAALAAHEIERAVKENKR---------------------------------
----------RVKVRVPNIKLLSDINLKIDDAVHRYITDQKKR-IDVRGPFFTTVVAEIS
G--------------------------------

#MSBL1 archaeon SCGC-AAA259E19
---------------MKFDIDEIKKKAR-KNYERAWKESKDLIEKKS----RFSPP-SKK
--GSTHPLFDLVQEFRNLFVDLGFREVLVPVLVEKGEIRKQYGPQAPIILDRIFFLAGLD
RSDLGISQEDLEKIREEVPD----------------------FDKLEELEKIFRGYKEGE
ISADDLSEVLVKKLEIEEDQASHILS--LFEDFKELEP--VPSSMTLRSHTTAGWFSVLQ
--GMKDRESLPIQLFTVGPKYRREQELDETHLYRSWTASIVVMAEEIGQDDGEELAKKIM
EKLGFEKIDCKTKEA-TSKYYAPGSEFEIFVEHPGTGE----------MVEVGNGGFYSP
VSLANYDIQ--YPVFNLGVGLERVLMIRTGEKDIRELVYPYRYK--ELELSDEQIARMLK
IKKKPATEPGKKLAEKIEQVARKYKDKPSPCEFEVSEGEIHG------------------
--TEVLVKIVEPEEDTKLVGPAAFNFVHVVDGNIVGVPPE----GWEENDFLQKARKEGI
STGINYIKAFSNLAAREIEEAVDEGED---------------------------------
----------RVKIRVPIVESLTDINLNLERPARRYITNKNKR-IDVRGPVFVTILADIG
---------------------------------

#MSBL1 archaeon SCGC-AAA261F19
---------------MGFDIERVKRRAE-EDFEQAWLDSKDMIKKKG----KLLSL-RKK
--GSSHPLFDLIQEIRQLLIELGFREVVVPTLIEKSEIFKQYGPQAPVILDRIFFLAALE
RPDIGISNEKISKIKKIVKG----------------------FDSVDGLQSVFRRYKTGK
IEADDLSEVLMDELGVEEGQATAILS--LFEEFQELEP--VPTNMTLRSHTTAGWFFVLQ
--EVQQREDLPIQLFTVGSKYRREQKLDRTHLCKSWTASLVVMTEKMSLEDGKRLTEKIM
KELGFEEVDFNIKRA-TSKYYAPQTEFEVYVKHPETGE----------MIEVGDAGFYSP
VTLARYGIS--NQVFNLGIGLERILMIREGEVDIRALVYPYQYR--ELEFSDNEVARTLE
FEKEPKTNIGKKIAERIKKTAEDHKDEPSPCKFSAFKGKIND------------------
--SEVLVQIVEPEKDTKLIGPAGFNEIYIYDGNVIGVPPE----GWKEDEFLKKVREKGI
STGITYMDAFAAQVASEIEEAVQKGKG---------------------------------
----------EVKVRVPIARSLNDINLKLGKSARRYITNNKKR-IDVRGPVFATAIVKID
---------------------------------

#Ca. Bathyarchaeota archaeon BA2
---------------MRVDTKKLLKAAK-EDCEKTWAESAKLLKVKG----KYFTL-TDK
--RKAHPLFDLIIKTRNIFLEMGFEEVITPLITEECHVYLQYGPEAPLILDRIFYLAGLP
RADIGISKEKISEIKKIMPN----------------------LNEIKQLQHIFRRYKRGE
IESDDLTEVMVKELKVKEEQATQIIS--LFPEFKRLTP--VPTKLTLRSHTTSSWFPILK
--EMQFKEPLPLQLFSIGPKFRREQKLDVTHLYESWTASMAIMAEEMSLEDGQKIVEEIF
KKLDFGKVEFRIKRA-TSKYYAPKTEFEAFVRHPKTEE----------FIEVGDGGLYNP
LSLARYKIL--YPVFNFGAGLERIAMIKTGVEDIRKLVYPHLYV--KNEYTDKQLAEMVE
VDQTPSTREGEKLVKAIIQTAIKNADQASPCRFLVYKGEFLG------------------
--HNIEVYVYEPDVGAKLIGPAALNTIYVYDGNILGVPEE----EMLKTEIAREAREKGV
STGICYLDAIAAQAVATIEEKIKAGET---------------------------------
---------GEVGIRVKIAKLPSDVNIRVQDAGVSSITSKNKR-IIVKGPVFVGIKAKIM
K--------------------------------

#GB (pMC2A209) archaeon No. 9
---------------MAWDPRAIRRRAE-EDYEATWLETASLLKRRG----RFLSWAKGA
-RGKPHPICELIQRFRSILLSYGFEEVINPTIVPEGEVRRQYGPEAPIILDRVFYLAGLP
RPDLGIRAELAEKIRSEVVPG-------------------AGEDLIEGLKAIFRAYKEGR
IEADDLVEEMVKQLGIRPEQATAILA--LFPELRTLTP--VPTRLTLRSHMTALWFPVLA
--ALQDKKPLPIKLFSIGLKWRREQRLDETHLYDSHVASLVVMAKQISLEDGEELVRAIL
ADLGFKEARFVVKEA-TSKYYAPGMEVEVFIRHR--GN----------WVEVADIGLYSP
VSLSNYYIR--YPVFNAGFGIERIAMILYGYDDIRVLAYPQLYA--EVEFTDEQLAGMIS
IRELPTTPEGFEVARAIVRAAEQNANLPSPCEVLAYDGPLMG------------------
--RRVRVFLFEREPNARLLGPAAFNRIYVYDGSILGLPDV----GMEHVPSVREARERGV
KTDIRYIDGVAMLAARGIEEALRSGRE---------------------------------
----------GYELRIRMVKRPSDVNIAIDDVARRYITARRKK-IEVKGPAFIGIRAEVV
ANRH-----------------------------

#GB (pMC2A209) archaeon No. 10
---------------MAWNPREIKRKAE-EDYEATWLETAKLLKRRG----RFLRWPKGA
LRGKPHPICELIQRFRSILLSYGFEEVINPTIVPEGEVKRQYGPEAPIILDRVFYLAGLP
RPDIGIKAELARKIRSEIVPG-------------------AGEELVEGLKAVFRAYKEGR
VEADDLVEEMVRRLNIRPEQATAILA--LFPELTTLTP--VPTRLTLRSHMTALWFPVLA
--ALQDKKPLPIKLFSIGLKWRREQRLDETHLYDSHVASLVVMAEQISLEDGEELVKAIL
ADLGFKEARFVVKEA-TSKYYAPGMEVEVFIRHG--GS----------WVEVADIGLYSP
VSLSNYYIR--YPVFNAGFGVERIAMILYGYDDIRVLAYPQLYA--EVEFADEQLAEMVR
IGELPTTPEGFEIARAIVRAAELNADLPSPCEVLAYDGPLMG------------------
--RR------------------------------------------DHLPGVREAREKGV
RTDIRYIDGVAMLAAKGVEDAVRAGRE---------------------------------
----------SYELRVRMVKRPSDVNIAIDDVARRYITARRKK-IEVKGPAFIGIRAEVV
S--------------------------------

#GB (pMC2A209) archaeon No. 11
---------------MAWDPKAIREKAR-KDYERAWLETASLLKREG----RFLRWEKGQ
-PGKPHPVCELVQRFRSILLSYGFEEVINPTIVPDSEVRRQYGPEAPVILDRVFYLAGLP
RPDIGIKAEVAEKITREIVPG-------------------AGEGLIEGLKALFRAYKEGQ
VEADDLVEEMVKRLGIRPEQATAILA--LFPELATLTP--VPTRLTLRSHMTALWFPVLA
--ALQDKRPLPIKLFSVGLKWRREQRLDETHLYDSHVASLVVMAEQISLEDGVELVKAIL
ADLGFKEARFVVKEA-TSKYYAPGMEVEVFVRHPRTGE----------WIEVGDIGLYSP
VSLSNYYIR--YPVFNAGFGVERIAMILEGYEDIRVLAYPQLYA--EVEFTDEQLAEMVR
IGVRPLAPDGFRLAEAIVKAAEENKDLPSPCEVLAYEGPFLG------------------
--REVRVWLYEHEPNARLLGPAALNRIYIYQGNVLGLPDK----GLEHVPEVVEARARGV
RTGIRYIDGIAMLAARRLEEAVQAGQE---------------------------------
----------TLDLRVRMAKRPSDVNIAIDDVARRYITSRRKR-IEVKGPVFVGIRAEIG
TS-------------------------------

#CP (AK8) archaea
-----------------------MEKAE-QDYEKAWIETKNLLTIKG----RKFKL-EPK
--GAPNPIIEFNSLVRRVLIEMGFEEVVLPMIVKESDVYQEYGPEAPVILDRLFYLAGLP
RPDIGLGADSIKKIREIVPD----------------------FDKVKELQDILRRYKKNE
IESDDLVETMVTELGVREEQATSILD-NVFLEFKERKP--VPTDLTLRSHMSALWFPVLS
--AVMNKWKLPIQLFTIGRKFRREQRLDATHLYSSYTASFVIMAEEITLEDGMEIVRDFL
RRTGFQEVDFRFKKA-TSKYYAPQTEFEIFVRHPKSGD----------WLEVGDSGFYSP
VSLAKFGIE--YPVLNAGFGIERFVMIKTGETDIRRLSYPYFYK--DVGFTDEEIAEGIY
IEEKPQTETGRKIMQELIKTAEAHRDAEAPCEFVAWSGTIDG------------------
--KKVSVKILEKDKDTKLLGPAILNRIYVYDGNILGLPEE---------------KAVGT
PTNIRYLDALGAYAARRIEEMIEKNEK---------------------------------
----------NLELRIRIAKKASDVNIGVTTPVRHFITSKSKK-IDIRGPVFFTVSASVE
---------------------------------

#GB (AK8) archaeon No. 8
-----------------------MEKAE-KDYEQAWIETKNLLPLKG----RRFTL-KRK
--GAPNPIVEFINEVRNALVEMGFEEVILPVFVKESDVYREYGPESPVILDRLFYLAVLP
RPDIGLAEDSIKKIRKIVPG----------------------FSKTKELQSILRRYKRNE
IESDDLVETMVTKLGIREEQATSIVD-NVFSEFKNRKP--IPTDLTLRSHMTALWFPVLS
--AVQNKWELPVQLFTIGQKFRREQRLDATHLYASYTASFVIMAEEITLQDGMDIVREFF
QRIGFEEVDFQFKKA-TSKYYAPQTEFEIFVKHPETGE----------WLEVGDSGFYSP
VSLAKFDIE--YPVFNAGFGVERFVMIRTGETDIRRLSYPYFYR--EVEFSDEEIAKGVY
VVEQPKTETGKKIMEALIRTAETRKDEESPCEFVAWKGKIGG------------------
--KTVTVKVWERDKNTKLLGAAALNKIYVIDGNILGLPED---------------KAIGT
PTGIRYLDALAARVARKIEEMVEKNEK---------------------------------
----------ELN----------------KTPVRHYITSNSKK-IDIRGPIFLGVSVTVE
---------------------------------

#GB (W8A-19) archaeon No. 7
---------------MRFNIKALLEKAK-KDYESAWMESRRFLRIEG----RCFRL-KPL
--GKSNEIMDFIEEVRRTLVEMGFEEVILPVIVNESEVYKEYGPEAPIILDRVFYLAGLP
RPDIGLSKDKIRQIKEMTPS----------------------FNRFRELQRLFRRYKKGE
IEADDLVEAMVKELELTESDATKIID-GVFQEFKELKP--IPSSLTLRSHMTALWFPVLS
--EIYRWRDLPVQLFTVGQKFRREQRLDAVHLYSSFTASLVIMTDEITLDDGKEVVREFL
RKIGFSKAEFRIKDA-TSKYYAPRTEFEVFIQHPETKE----------WIEVGDCGFYSP
VSLAKYGIG--VPVFNAGFGLERFVMIRTGETDIRRLTYPYFYK--REFYLDEEIAESLS
YAEYPETGLGREIMNALINTAEANKDADAPCEFTAWKGNTSK------------------
--GELTVSVFETEKGVKLLGPAALNVICVENGNILGLPLE---------------KAKGV
RAKLRYLDGLAALFARKVEKEILNGEK---------------------------------
---------MELSMRVKMVKSAADINLLVRDNIRFYLTSKNKK-IDVRGPVFIGFSARLN
R--------------------------------

#Jinze (W8A-19) archaeon
---------------MRFNIKKLLERAE-EDYEAAWVESRELLKLEG----RYFRL-KPL
--GRSNEIMNFIEEVRKTLVEMGFEEVVLPVIVDESEVRKEYGPEATIILDRVFYLAGLP
RPDVGLSKEDAERIKELVPS----------------------FNEFEKLREILRKYKKRK
IEADDLVETMVNELSLQEEDATRIID-TVFHAFKNLTP--VPSPLTLRSHMTALWFPVLS
AIYKYKWKELPVQLFTVGQKFRREQKIDPTHLYSSYTASFVIMTEEISLEDGKSIVQEFL
GRLGFSKAEFRLKKA-TSKYYAPQTEFEVFIQHPETSE----------WIEVGDCGFYSP
VSLAKYDID--VPVFNAGFGIERFVMIRTGETDVRRLTYPYFYR--RDIYSDEEIAEGIC
YAEKPETELGEEIMNSIISVAEEHMHADAPCKFTAWKGKIDR------------------
--GELTVTVFEEEKGVKLLGPAALNVICIHEGNILGLPPS---------------EVRGT
VTKLRYLDGVAALFARKVEKEILEEGK---------------------------------
---------REVSMKVRMAKKPSDVNIYLSEQVRRYISSKNKR-IDIRGPVFISLAASLN
DS-------------------------------

#LHC4sed (W8A-19) archaeon
---------------MKFNIKKLLEKAE-EDYEAAWIESRELLKIDG----RYFRP-KPL
--GKSNEIMDFIEEVRKTLVEMGFEEVVLPVIVDEGEVRKEYGPEATVILDRVFYLAGLP
RPDVGLSKEDIEHVKKLVPS----------------------FNEFEKLREVLRRYKKGK
IEADDLVETMVKELGLREDDATRIID-AVFHEFKKLTP--ISSPLTLRSHMTALWFPVLS
AIYKYKWRELPVQLFTIGQKFRREQKIDPTHLYSSYTASFVIMTEEISLEDGKNIVQEFL
KKLGFPDASFKLKTA-TSKYYAPQTEFEVFIQHPETKE----------WIEVGDCGFYSP
ISLAKYDVD--VPVFNAGFGVERFVMIRTGETDVRRLTYPYFYR--RDIYSDEEIAEGIC
YAEKPETELGEEIMNAIIKVAEAHKDADAPCKFTAWKGKASG------------------
--GELTVTVFEEEEGVKLLGPAALNVICVHEGNILGLPPS---------------EVKGK
ITGLRYLDGVAALFARKVEKEILEEGK---------------------------------
---------REVNVKVRMAKKPSDVNLYLSEQVRHYISSKKKR-IDVRGPVFISLAASLE
ET-------------------------------

#Groundwater_a1
---------------MKLDVKKIREQSE-QDFEKAWIETADLLPKKG-----EIVLKKRR
--GEPSVMREMAQKIREALLSMGFDEMENRHVLDEADVYKQYGPESPVILDRCFYLAALP
RPELGIGKEKEAIIQKIDHS--------------------FSEKKKEKLAEILRRYKSGD
LEGDDFVDALVQGLGWKEEQATAVMD-KAFPELRKIRP--VPSNMVLRSHMTAVWFETLA
--GMQDKREHPIALFSIGPRFRNEQKEDASHLRVHHGASIVIMGENVTVEAGNKIASKLF
EKLGYSKPEFELKPA-TSKYYAPKSEYEVYVEWK--GK----------RLEVADSGMYSP
VALANYGIK--YPVYNIGFGLERMAMIQYSYGDIRELVYPQFFS--KLELSDEQIAAAIR
IAVQPKTEEGRNLADLLYHKTLKEKDKDTPYSFTAFKGRLNK------------------
--KSIVLDVFKDEEGKKLLGPAGLNKIYVYDGGVVAYVPE---------KLDAAVREKGI
DTGLDFLKAMCAYVAAKAEDAVESGEK---------------------------------
----------EFEYVFGMAKAPSDVNIAIPEHVHRFVTSKNKK-LDFRGPIFFGVRFKLA
---------------------------------

#Groundwater_a2
---------------MKLDVKKIREQSE-KDFEKAWIDTAELLPKKG-----EISLRKKR
--GEPSVMREMAQKIREALLSMGFDEMENRHVLDEADVYKQYGPESPVILDRCFYLAALP
RPELGISKEKEGIIHKIDAK--------------------FSAEKKEKLAEILRRYKSGD
LEGDDFVDALVQGLGWKEEQATAVMD-KAFPELRKIRP--VPNNMVLRSHMTAVWFETLA
--GLQDKREHPVALFSIGPRFRNEQKEDASHLRVHHGASIVIMGENVTAEAGNRIAAKVF
EKLGYSKPEFELKAA-TSKYYAPKSEYEVYVDWK--GK----------RLEVADSGMYSP
VALANYGIK--YPVYNIGFGLERMAMIQYAYGDIRELVYPQFFS--KLELTDAQIAASIR
IAAAPKTEEGKNLADLLYHKTLKEKDRDTPYTFTAFKGRLNK------------------
--KPIVLDVFKDEAGKKLLGPAGLNKIYAYDGGIVAYTPE---------KLDPAVREKGV
DSGLDFLKAMCAYVAARAEDAVANDEK---------------------------------
----------EFEHVFGMAKAPSDVNIAIPEHVNRFVTSKNRK-LDFRGPIFFGVRFRLT
---------------------------------

#MTBE-degrading_b1
--------------MAAFDTGEIKSKAK-SNFTDAWIATAKLIPSGT-----EISL-TRE
--GKPHLVRELIQKSRQILLNMGFDEVENLTLLPDSDVVKQYGPEARVILDRAFYLAELP
RPDIGLSARRIARLKKIAE-----------------------EIDIEKLQSILRNYKKGE
IEADNLIEELIAGLSITDYQATELMD-KVFPEIKELQP--VPSNKTLRSHMTATWFHTLA
--ALQDKASFPVALFSVGPRYRNEQREDARHLRVHHSASIVVMDPEMSLDAGRAVTRDIM
QQYGFSDIKFETKTA-TSKYYAPGQEQEVFVNHK--ST----------WLEIADIGMYSP
VALANFEIK--YPVFNAGFGIERLGMLIYEIDDVRKLAYPQFS---VTEYSDEEIAKSIT
YIASPETTRGQKIARAIEETARRHKDEIAPCEFSAWQ----D------------------
--KSIEVKVVEKEAGKRLIGPAGFNEICVVDGSIYSDVVP-----------------SGV
HTGINYMHTIAMGAAAAIESSND-------------------------------------
----------NLTYQVKGIRHLSDLNLQIPEAVREHIEGQQKK-IGIAGAVFVTIESRPV
GGESDKASGK-----------------------

#Lake_sediment_b1
------------------------------------------IPTGT-----KISL-PKK
--GKPHLVRELIQKSRQILLNLGFDEVENLTLLPDSDVVKQYGPEARVILDRAFYLAELP
RPDIGLSARRIAQLKKIAE-----------------------EIDIEKLQTILRNYKKGE
IEADNLIEELIAGLGITDYQATELLD-KVFPEIKDLQP--VPSNKTLRSHMTATWFHTLA
--ALQDKASFPVALFSVGPRYRNEQREDARHLRVHHSASIVVMDPEMSLDAGRAITRDIM
QQYGFSDIKFETKIA-TSKYYAPGQEQEVFVNYK--GT----------WLEIADIGMYSP
VALANFDIK--YPVFNAGFGIERLGMLIYEIDDVRKLAYPQFS---VTEYSDEEIAKSIT
YIASPQTAKGQKIAKAIEETARRHKDEIAPCEFLAWK----D------------------
--KSIEVKVVETETGKRLIGPAGFNKICVANGTIYSDVAP-----------------SGV
YTEINYMRAIAMGAAAAIESSND-------------------------------------
----------NLTYQVKGIRHLSDLNLQIPEAVRQHIEGQQKK-IGVGGAVFVAIEVKKL
---------------------------------

#Deep_marine_Sb1
--------------MAAFDTGEIKSKAK-SNFIDAWIATAKLIPTGT-----EISL-AKK
--GKPHLVRELIQKSRQILLDLGFDEVENLTLLPDSDVSKQYGPEARVILDRAFYLAELP
RPDIGLSARRMGEVRKIAQ-----------------------GMDVGKLQTILRNYKKGE
IEADNLVEELIIGLGITDYQATELLD-RVLPEIRELQP--LPSNKTLRSHMTATWFHTLA
--ALQYKAGFPVALFSVGPRYRNEQREDAHHLRVHHSASMVVMDPAMSLDAGRAIAENIM
QQYGFSDVKFETKTA-TSKYYAPGQEQEVFVKHK--DT----------WLEIADIGMYSP
VALANFDIK--YPVFNAGFGIERLGMLIHEIDDMRKLAYPQFA---IIEYSDEEIAKSIS
YMASPQTARGQVIARAIEETARKHKDELGPCEFLAWK----D------------------
--ESTEVKVLEKEAGKRLIGPAGFNEICVAEGVIYSDVAP-----------------SGV
YTGINYMRAISMGAAAAIENSSD-------------------------------------
----------NLTYQVKGIKHLSDLNLEIPEAVRQHIEGQRKK-IGVGGAVFVAIEARRF
---------------------------------

#Alkali_sediment_b2
--------------MAAFDTGEIKSKAK-GRFTEAWLDTAGLIPRGT-----SISL-TGK
--GKPHLVRDLIQRSRQILLDLGFDEAENLTILPEGDVVKQYGPEARVILDRSFYLAELP
RPDIGLSARRIARIKKIAE-----------------------TADIPRLQSILRKYKKGE
IEADNLTEEFISALGITDYQATEILE-KVFPEVKKLQP--VPTDRTLRSHMTATWFHTLA
--ALQHKVSFPAALFSVGQRYRNEQREDAHHLRVHHSASIVVMDDRMSLDAGRQITTAIM
QQYGFADVRFETKMA-TSKYYAPEHEQEVFVNHR--GT----------WLEVADIGMYSP
VALANFDIK--YPVFNAGFGVERLGMLLYGVDDIRRLVYPQFS---VAEYRDEDMAESIT
YIARPGTDRGQRIARAIEKTARRHKDAIAPCEFLAWG----D------------------
--KVCEVWLVEEEAGKKLIGPAGFNEICVADGTIYSDIVP-----------------SGT
PTGINYMRAIAAGAAAAIERSSD-------------------------------------
----------ALTYQVKGVKHLSDLNLEIPEAVRRHIEKQHKK-IGVGGAVFVTIEARPV
GGHSGRTSRE-----------------------

#Crude_oil_b1
--------------MANFDLNDIKERAK-KNFSNVWVSSTKLIPVDT-----KISL-KNK
--GKPHPVQELIQKSREILLSLGFDEVENFTILPNSDVVKQYGPEALVILDRIFYLAELP
RPDIGLSAKKIVQVKKIAG-----------------------EVELEKLQSILRSYKRGE
IEADDLVEELIARLNITDYQATELLD-KVFPELRKLRP--IPSDKTLRSHMTATWFHTLA
--ALQDKAAFPLALFSVGLRYRNEQREDAQHLRVHHSASIVVMDPEMSLDAGREITREII
RQYGFPDMKFETKIA-TSKYYASGQEQEVFVRHK--DN----------WLEIADIGMYSP
VALANFDIR--YPVFNAGLGVERLAMLLYEIDDVRKLVYPQFS---LSDYSDEEIAKSIT
YIASPRTPRGRKIAQAIEDSARKYKDEIAPCEFLAWQ----D------------------
--KSIKVKVIETEAGKKLIGPAGFNEICVDDGAIYSNVVP-----------------SGL
YTGINYMRAIAMGAAAAIENNPE-------------------------------------
----------DFTYQVKTIKHLSDLNLQTSEGVRQHIEGKQRK-IGVGGAVFVTIKAQPV
K--------------------------------

#Dehalococcoidia bacterium CG2_30_46_9
--------------MTIFDLNEIKTQAK-RNFADAWISTAKLIPQGT-----KIIL-KKT
--GKPHPVRELIQRSREILLKLGFDEVENFTILPDTDVVKQYGPEARVILDRVFYLAELP
RPDIGLSVKKMTQVKKIAG-----------------------EIEVEKLQSILRNYKRGE
IEADNLVEELITKLNISDYQATELLN-RVFPELKNLRP--LPSNKTLRSHMTATWFHTLA
--ALQDKATFPLALFSVGPRYRNEQREDANHLRVHHSASIVIMDPEMSLDAGREITREIM
KQYGFSDMKFETKTA-TSKYYAAGQEQEVFVSYR--GD----------WFEIADIGMYSP
VALANFDIK--YPVFNAGLGVERLAMILYELDDVRKLAYPQFS---VVPYTDEEIAKSIT
GIASPRTARGKKIAQAIEESARKYKDEIAPCEFLAWK----D------------------
--KSIEVKVLETEAGKKLIGPAGFNEICVADGTIYSATIP-----------------SGV
YTGINYMRAIAMGVAAAIENSHG-------------------------------------
----------ELTYQVKTIKHLSDLNLQIPEGVRQYIQGRQKR-IGIGGAAFVTIKAKPV
K--------------------------------

#Hot_spring_Yb1
--------------MATFDVNDIKTKAK-RNFTEAWIATAGLIPTNT-----EVSL-PRK
--GRPHPVRELIQRSREILLGLGFDEVENLTILPDTDVVKQYGPEARVILDRAFYLAELP
RPDIGLSSKKIEEIKKIAA-----------------------DISIDGLQAILRDYKKGE
IEADNLVEVLIHKLGINDYQATDLLD-KVFPEIKELHP--LPTNKTLRSHMTATWFHTLA
--ALQDRINFPVALFSVGPRYRNEQREDAQHLRVHHSASIVIMDPQMSLDAGRAVTRKIL
EQYGFFEIKFETKVA-TSKYYAPGQEQEVFVKHK--GS----------WIEIADIGMYSP
VALANFDIR--YPVFNAGFGIERLGMLLYDANDVRKLAYPQFS---LVEYSDEEIAQSIS
YINAPKTARGMEIAKAIEETARKYRGEIAPCEFVAWE----D------------------
--DSIEVKLVEKEPGKKLIGPAGFNEICVANGTIYSDLTP-----------------SGI
HTGINYMRAIAMGAAAAIEDGKE-------------------------------------
----------DLTYSVKMIRHLSDLNLQIPEAVRRYIEGQRKK-IGVGGPVFVTIHMRKK
AKQRS----------------------------

#Lake_Sakinaw_b1
--------------MGIFDLDEIKIQSK-RNFTETWMATAKLIPSGT-----NISL-EKK
--GKPHILCELIQKSREILLNLGFDEVENLTILPDSDVSKQYGPEARVILDRVFYLAELP
RPEIGLSDQKIAHVKKIAA-----------------------KASIEELKSILRSFKKGE
IEADDLVEGLVTGLNITDYQAIELLD-TVFPELKKLRP--VPSNKTLRSHMTATWFHTLA
--AMQHKVSLPLALFSVGTRYRNEQREDAHHLRVHHSASIVIMDPKMSLDAGEEIARKIL
QQYGFSDIKFETKVA-TSKYYAPGHEQEIFAKYK--GD----------WLEVADMGMYSP
ISLANFNIE--YPVFNIGLGVERLAMILNDVDDIRKLTYPQFS---VVEYSDDDIAKSIT
YIANPKTDVGRNIARAIEETARKYKDEIAPCEFLAWR----D------------------
--DSREVKIVESEVSKRLIGPAGFNEICVADGSIYSDVAH-----------------SGV
YTGINYMRAIAMGAAALIESTDE-------------------------------------
----------NLIYQVKMIKHLSDLNLQIPEAIRQHIEGQQKK-IGVGGAVFVTIKVQSS
K--------------------------------

#Crystal_Geyser_b4
--------------MATFDLNEIKAQAK-KNFTEAWVSTGKLIPKGT-----KISL-ERK
--GKPHLVRQLVQQSREILLNLGFDEVENLTILPDYDVSKQYGPEARVILDRVFYLAELP
RPEIGLSASKITQVKKIAA-----------------------EVDIEKLRSILRRYKKGE
IEADNLIEELIAGLDITDYQATELLN-RVFPELRKLRP--LPSNKTLRSHMTATWFHTLA
--ALQGKAEFPLALFSVGPRYRNEQREDAHHLRVHHSASVVVMAPEMSLDAGREITRDII
RQYGFSDIKFETKTA-TSKYYAPGQEQEAFINYK--GD----------WLEVADIGMYSP
ISLANFDIR--YPVFNAGLGIERLAMILYEVDDMRKLAYPQFS---VAEYSDEDIAGAIT
YITNPKTARGKKIAKAIEETAQKYKDEIAPCEFLAWV----D------------------
--DSIQVKVVETEAGKRLIGPAGFNEICVANGSIYSDVVP-----------------SGV
YTGINYMRAIAMGAAALIESGDE-------------------------------------
----------SLTYQVKTIKHLSDLNLRIPEAIRGCIEGQQKK-IGVGGAVFVTIKSQVL
KRKTS----------------------------

#Dehalococcoidia bacterium CG2_30_46_19
-------------MAEMFDLGRIRAQAK-GDFTEAWMSTAKLLPVDT-----KVSL-QGR
--GKPHLLRELIQKSREILLRLGFDEVENLTILPDSDVSKQYGPEARVILDRVYYLAELP
RPEIGLSNKKIIEAKKIVG-----------------------ELDVKALRTILRAYKKGE
IEADNLVEELINALDITDRQATELLS-RVFPELEKMRP--LPSNKTLRSHMTGTWFHTLA
--AIQDKAKFPVALFSVGPRYRNEQREDAHHLRIHHSASIVIMDANISLSAGREITEEIL
RQYGFSDIKIETKMA-TSKYYAPGQEQEVFINHK--SE----------WLEVADIGMYSP
VSLANFGIK--YPVFNAGLGIERLAMILYGIDDVREVAYPQFS---VAEFSDEEIARSIT
YLASPRTSRGRKIASALEQTARKHKDEIAPCEYVAWE----D------------------
--DLVVVKVVEVEQGKRLIGPAGFNEICVADGTIYSDIVP-----------------SGE
YSGINYMHAIAMAAAALIEDSHE-------------------------------------
----------DLPYQVKTVRHLSDLNLQIPEAILEYIERQQKK-IGVRGAVFVTIKSHFK
GRKDKVEG-------------------------

#Lake_sediment_b2
-------------MAEMFDLSKIRAEAR-RDFTEAWMSTAKLLPVDT-----KVSL-QGR
--GKPHLLRELIQKSRETLLRLGFDEVENLTILPDSDVSKQYGPEARVILDRVYYLAELP
RPEIGLSNKKIIEAKKIVG-----------------------ELDVKELRTILRAYKKGE
IEADNLVEELINALDITDRQATELLS-RVFPELEKMRP--LPSNKTLRSHMTGTWFHTLA
--AIQDKAKFPVALFSVGPRYRNEQREDAHHLRIHHSASIVIMDANISLSAGREITEEIL
RQYGFSDIKIETKMA-TSKYYAPGQEQEVFINHK--GE----------WLEVADIGMYSP
VSLANFGIK--YPVFNAGLGIERLAMILYGIDDVRKVAYPQFS---VAEFSDEEIARSIT
YLASPRTSRGRKIASALEQTARKHKDEIAPCEYVAWE----D------------------
--DLVVVKVVEVEQGKRLIGPAGFNEICVADGTIYSDIVP-----------------SGE
YSGINYMHAIAMAAAALIEDSHE-------------------------------------
----------DLTYQVKTVRHLSDLNLQIPEAILEYIERQQKK-IGVRGAVFVTIKSHFK
GKKSK----------------------------

#Deep_marine_Sb2
----------------MFDLNKIRTQAR-KDFTETWTSTSRLLPIDT-----EVRL-QGR
--GRAHILRDLIQKSREILLNLGFDEVENLTILPDSDVSKQYGPEARVILDRVYYLAELP
RPEIGLSNKKITEAKKIVQ-----------------------ELDVRKLRDILRAYKKGE
VEADNLVEELIGGLGISDHQATDLLS-RVFPELEKMRP--LSSNKTLRSHMTATWFHTLA
--AIQDKAKFPVALFSVGPRYRNEQREDAHHLRIHHSASIVIMDANISLSAGRKITEEIL
RQYGFSDIRVETKMA-TSKYYAPGQEQEVFVNYN--GE----------WIEVADIGMYSP
VSLANFNVK--YPVFNAGLGIERLAMILHEIDDIRKVAYPQFS---VVEFSDEEIVGSIS
YIASPGTSRGKKIASAIEQTARKHKDEIAPCEFLAWE----D------------------
--DLVEVKVVETEEDKRLIGPAGFNEICVADGTIYSDIVP-----------------SGE
YSGVNYMRAIAMGAAALIENSHE-------------------------------------
----------DLTYQVKAIKHLSDLNLQIPTPIREYIEKQQKK-IGVSGAVFITVESHFK
GGKSKVEG-------------------------

#Alkali_sediment_b1
--------------MAVFDVSSIRSNAK-TDFSGTWLSTASLLPRNT-----SVRL-PSR
--GRPHPVRDLMQRSREILLTRGFDEAENLTILSDSDVTKQYGPEALVILDRAFYLAQLP
RPEIGVSAQRVKEVERIVDR----------------------PIDVEELGAILRSYKRGD
IESDDLIEALMKRLDITDVQALDIIH-KVFPELYGLKP--VPTDKTLRSHMTATWFHTLA
--AMQDRAMYPVALFAVGPRYRNEQREDAHHLRVHHSASIVVMDPEMSLEAGRAITSEVL
REYGFEDTSFERKAS-TSKYYAPGLEEEVFVKYR--GQ----------WLEVADIGMYSP
VALSNFDIR--HPVFNAGMGIERLAMILHGSDDIRKLVYPQFS---VVEYSDKDIAESLS
FISAPRTDRGTKISQAIEGSARKHKDEIAPCEFVAFR----D------------------
--NRVEVKLVEREEGKKLVGPAGFNELCVADGTIYSDLQP-----------------SGT
YTGLNYMRAISMAAAALAEDATE-------------------------------------
----------PRELQVKMIRHLSDLNLELPDAVRQHIERQQKK-IRIGGGVFVTIEIHPV
QVEGGSSAATS----------------------

#Chloroflexi bacterium RBG_13_51_36
--------------MSTFDTGEIKSKAR-ENFTDAWMATAKLIPSGT-----EISL-PNK
--GKPHLVRELIQKSREILLNLGFDEVENLTVLPDSDVAKQYGPEARVILDRAFYLAELP
RPDIGLSEKRIAQVKEIAK-----------------------EIDIEKLQTIFRNYKKGE
IESDNLIEELIARLGITDYQATELMD-KVFPEVRELQP--VPSNKTLRSHMTATWFHSLA
--ALQNKASFPVALFSVGPRYRNEQREDARHLRVHHSASIVVMDPEASLDAGSAITRDIM
RQYGFTDIKFETKMA-TSKYYAPGQEQEVFIKHK--GS----------WLEIADIGMYSP
VALANFDIK--YPVFNAGFGIERLGMLIYEIDDLRRLAYPQFS---ITEYSDEEIAESIT
YIASPQTARGQEIAQAIEKTARQHKDEIAPCEFLAWK----D------------------
--EFIEVKIVETEAGKRLIGPAGFNEICVAKGAIYSDVVP-----------------SGT
HTGINYMRAVAMGAAAAIEDSTS-------------------------------------
----------NLTYRVKGIKHLSDLNLQIPETIRQHIEGRQKK-IGVSGPVFVTIEVNKR
L--------------------------------

#CG (Woesearchaeota) archaeon
--------------MVRFNTKRIQKDAQ-ADFERTWIETSEQLPRNT-----SVRI-RSQ
--GSTNPLRETVQKCREALIEIGFQEYENQTILPASDVYLQYGPEAPVILDRAFYLATLP
RPELGLSADKIGLTEGIIG-----------------------RFDQSTLQEILRSYKKGE
IEGDDFVETLVKRLSIDTGKATAIIE-RVFPELKEIAP--VPTEMTLRSHMTATWYHTLA
--AMQNKTHFPLAMFAVGPRYRNEQREDKGHLRVHNSASMVVMDPNMSLEAGKELTLEFL
VKMGFSDARFDVKAG-TSKYYAHQQEQEVFAKWH--GE----------WLEIGDIGMYSP
ISLANFGIT--SPVFNGGFGVERMAMIFGRYRDIREMVYPQFH---SSRYDDEDIAKSLS
VIKEPKSERGKDIARGIEDVARKNYDAMAPVRLIAYE----D------------------
--ECLRVEVLEEEEGQKLLGPAGLNNVYVQDGSILSTPN------------------SID
NAVTTVLQSSSLGIASDIEQDLETGKE---------------------------------
----------SGTYRVRMAKNLSDVNLRLPTAVHNYIVGEHKG-IRVKGPVFLTVEYTVK
NLTEVIEG-------------------------

#CG bacterium No. 3
--------------MVSLNTQDIIKQSE-MDFEKAWAETSKLIPRDT-----RIEI-KGR
--GKPHPVRNMIQKSRRILLYMGFDETENKTILPDTDVYKEYGPEAPVILDRAFYLAKIP
RSDIGLGDSEIAQIKGVIG-----------------------DFDVRALQGILRSYKKGE
IEGDDFVEEIVKQLDISQEKAMRILESKVFARFKGLVP--QPTNLTLRSHMTATWYHTLA
--ALQHESTFPLALFSVGPRYRNEQREDSAHLRVHHSVSLVVMDPNMSLEAGRRITEGIL
KKYGFDEVEFETKKA-TSKYYAKDQEQEVFAKHK--GE----------WLEIADIGMYSA
ISLANFGIE--YPVFNAGFGIERLVMVLKDYEDIRELVYPQFG---TQEFSDERIAESLS
HVNEPQTERGEKIAEAIERTARKHKDKIAPCEFVAYE----D------------------
--DQVLVKIVEREEGKRLIGPAAFNEIYVKDGNIYSDVQP-----------------QVG
HAKFNYMKGISREFAHNIEKDIAKNEA---------------------------------
----------TSTYNVKLVRSLPDINLKLPQGIEDYLTGNHKQ-FLIGGPIFITVEYLRK
---------------------------------

#Parcubacteria DG_74_2 bin
------MDNNKTKNKGAWDIKKLREKKE-KDFEKTWIETAKLLPRNT-----RLKL-KKK
--GMPNLVTEMIEKSRKILLNAGFKEVINKTILSEDDIYKQYGPETPLILDRTYYLATPS
KPDIGLSKKRIEEISKIIG-----------------------SFAPEKLQSILRDYKKGI
IEGDDFTEEIVNKLKIKPEQATVIIE-KVFPEFKKNKP--LCSNLTLRGHMTATWYHTLA
--ALQDKEEFPIALFSVGPRYRNEQREDFSHLCVHNSASIVIMDPEISIEAGKKITKEIL
KEYGFKNAKFEKKKA-TAKYYTPEQEEEVFVKKF--GK----------WVEIANIGMYSP
ISLVNFKIR--YPVFNAGFGIERLAMVLENIKDIRKLVYPQFY---EKEYNDKEIAESIS
YIKEPSILKGKKIAKLIEEIARKEKDKTSPCEIEAYR----D------------------
--NSIKVQIVEKEENKKLIGPAGLNYICVKDKNIYPDISP-----------------SGT
PTGKSYISGIANAIAYEIEKGNI-------------------------------------
----------PFTLKVKTIKGLSDVNMKIPKHIRDYLEGKGRK-VGLSGYVFIEIQVKKI
KN-------------------------------

#CG (Parcubacteria) bacterium
---------------MQFHAKTIKEQAE-KDFEKTWFETANLLTKNG----RKIYWEKSL
--GKKHPLHELNSIFRKIFLSYGFAETELLSIIPEQEIYKQYGPEAPLILDRVFYLAGLS
RKELGISEEKKIEIKKIIPD----------------------FDNFENIKKLFRDYKKGE
IEGDDFIEEMVKRLKIKESEATQIIQ-NVFPELKSLEK--IPTNLTLRSHMTASWFPLLS
--AIQRNVKLPIKLFSIGKRYRKEQKQDANHLYESTSASIVVMAEDITLEDGKDLTKKII
ADLGFKKVKIIQKKT-TSKYYAPGMDLEVFVDFQ--GK----------DLEIANLGFYSP
VSLAKYKIW--YPVLNLGFGVERIAMILKGVDDIRKLIYP--------------------
------------------------------------------------------------
------------------------------------------------------------
------------------------------------------------------------
------------------------------------------------------------
---------------------------------

#Altiarchaeales archaeon MSI_SM1
-------------------------------------------------------MQTTQ
--GKAHPIHTLIQKTREIFLGLGFDEIENPLFIQEEDVYKQYGKEAPVILDRVFYLGGLP
RPDIGLSDEEILKIKEISP-----------------------YISIDKFKKILRNYREGS
IEGDNMLEEMLKNLKINATQAMEILD--LFPAFKNIEP--VCGRTTLRSHMTAAWFLTLD
-AEVEKQSELPLKFFSIGLRFRREQKLDATHLRAHYGASCIIADKNLNIEDGMNVANKIL
NDMGFTDIKFTKKKS-TSNYYEQDKEYEIFS------G----------SIEIADCGMYSK
IALKNYNIPDALDVFNIGFGLERILMVRNNIGDVRKVLYPQFYE--DVHLSDEEIAKSIS
MLYLPETDEGRTLAGKIYENGKIHANEKSPCKFLCFEGNLMN------------------
--RKIKIFIFEDEENKNLLGPAALNEIYVFDGGIYGIPED----TEKFGEEGKKIKEKSI
KANLNFLYAVSNYFAKEIEDRVKKATE---------------------------------
------GQKEKFILEVKMAKSPSDINIIIKGRAKRFIGNENKR-IVLKGPVFMGIEVEIG
---------------------------------

#Altiarchaeales archaeon CG_SM1
-------------------------------------------------------MQHKQ
--GKPHIIHTLIQKTREIFLSAGFDEVENPLFIQEEEVYKQYGKEAPVILDRVFYLGGLP
RPDIGLSDKEIAKIKEISP-----------------------YISTDKFKKILRNYREGA
IEGDNMLEEMIKGMKINSTQAMEILD--LFPAFKNIEP--VCSRTTLRSHMTAAWFLTLE
-AEIEKQTELPLKFFSIGLRFRREQRQDATHLRTHYGASCVIADKNLNIEDGMKTAEKIL
NEMGFANIVFTKKKV-TSNYYKQDLEYEIFS------N----------NIEIADCGMYSG
MALKNYNIPGDIDVFNIGFGLERILMVRNNISDIRKVLYPQFYE--DLHLSDEEIAKSIC
ISSVPETDEGRNIAGKIYETAKIHACEKSPCKFLCFEGNLMN------------------
--RWLKISVFEDEENKNLLGPAALNEIYVFEGSVYGIPGD----IEKFGEEGKNIKGKGI
KTNLNFLYTVSNYFAKEIENRVSKEGKEGKEGKEGKVGKEGKEGKEGKEGKEGKEGKAGK
ESKVKEGEKEKFCLEIKMAKSPSDVNIMIRGRARRFISTENKR-IVLKGPVFMGLEVEIN
---------------------------------

#Altiarchaeales archaeon WOR_SM1_79 or WOR_SM1_SCG
---------------MPFNTKQIKQEAR-ENFEKAWLKTAELLPQES-----KKDYTTGA
--GTEHPIHALIRDVRKIFIKFGFSEVENQIFISEDDVYKQYGPEAPVILDRVYYLAGLP
RPDIGLSDDKILEVREIAP-----------------------YINIKNFKKILRGYREGA
VEGDNILEVMVNELKIKTEQASGIIN--LFPEFKNITP--VCSKQTLRSHMTAAWFPTL-
--EVMQDSELPLKLFSIGLRFRREQKLDATHLRAHYGASCVIMDDDISLASGXKLTEKIL
NELNFYDINFXKKKA-TSNYYAPGMEYEVFS------E----------SIEVADCGMYSP
VALANYDIK--IPVFNLGFGLERVLMIRDKIGDVRELLYPQFYK--NLDLSDEQIAEQVR
IAHEPETKEGRELAEKISKTAKSHADEKSPCSFPVYEGDFLD------------------
--KKISVSVVEKEDNTKLLGPAALNEIYVYCGNVYGIPKN----PEKLSSDLIEVKKEGI
KVKFNFLDAVSSGIAYGIENKIKTGSK---------------------------------
----------KGLLQVKMAKTPSDLNIEIAKAARRFITSKNKK-IILKGPVFMSVEIQ--
---------------------------------

#Altiarchaeales archaeon SCGC AAA252-I15
---------------MAFDTRKLREDAK-TDFRKAWLESTSLVPKEG-----EISYSLRE
--GKAHPVQDLIQKVRKVFLDMGFDEIENQMFISEEDVYKQYGAEAPVILDRVYYLAGLP
RPDIGLSDEKIAEVKSVA------------------------DVKVEDLKKILREYREGE
VEGDNLLEEMVTRLKITTDEAAEILS--IFPEFKNITP--VAGKTTLRSHMTGAWFPTIA
--ALYGKRDLPLLLFSVGLRFRREQKVDATHLRAHYGASMVIVDDNINVEAGRKLTEEIL
KRLDFTDIRFVQKKA-TSNYYAPDSEYEVFS------G----------DIEVADIGMYSP
VALAQYDIP--VNVFNLGFGLERFIMAKEKHSDVRELLYPQFYE--NLNLTDEEILDNID
MIERPKTEEGKELAIAIMKTANKHGNENSPCSFKAFEGDLGG------------------
--KKVTVSVVEKEDGTKLIGPAALNEVVVYDGALYGIPKD----TSKLKGNIDGILENGV
HSVWNFIDAISNYFAAEIERQVQAGET---------------------------------
----------QGFIQVKMAKTSADINIKVGEKARRFMEGKNKP-ISIKGPVFTAVEFTVE
---------------------------------

#GB(Altiarchaeales)archaeon No. 1
-----------------------------------------------------MKSKGKK
--GKTHPLQDIIQEVRKVFIDMGFDEVENQIFIPEGDVYKQYGSEAPVILDRCYYLAGLP
RPDIGLGKKQIAAIENIHP-----------------------GFDFDEFKKILRGYREGS
VEGDDLAEEMVNRLGIGMDEALRIID--LIPEFRSITP--IPSRITLRSHMTAAWFPTLQ
--AMQDTHELPLRLFSIGLRFRREQRVDSTHLRVHYGASCVVMDDRFDIDDGKKLSGKIL
GDLGFRNIRFERKKA-TSNYYEKDTEFEIYS------N----------DLEIADCGMYSR
VSLSNYDIG--HPVFNLGFGLERILMIRSRKEDIREILYPQFYK--PLELSDEEIARQVK
INIKPKTEEGKELVKLLVREAKRYSDERSPCRFRVYSGKLLG------------------
--RKVDIDLVEREKNTKLLGPAALNEVYVYDGNIYGVPSD----VGKLAKELTRVKERGI
KVDFSYIDAVMNLFVANVENLIMGGDK-------------------------AMGETSAA
VVSYAPDGKIRGYMQIKMAKTPADLNIKIGSIVRRFITSKNKK-IYIRGPVFTALEVI--
---------------------------------

#Altiarchaeales archaeon WOR_SM1_86-2 and WOR_SM1_79
---------------MVFDTKKIKSKAK-ENFEKSWIETANLLPKES-----KKDYLGGG
--GRGHIIQDAMQEVRKIFLKLGFDEVENPVFIPEQEIYFQYGPEAPVILDRCYYLAGLP
RPDIGLGDEKIKEIEEIAP-----------------------KFNFDEFRKILRQYRAGV
IESDDLVEAMVNRINLRTEIASKIID--LFSEFREIEP--VASRTTLRSHMTASWFSTIE
--AMQYKHELPMKLFSIGLRFRREQKVDETHLRAHYGASCIIMDDEISIDAGKKITSRVL
GELGFKDVSFVRKKA-TSNYYAPETEYEVFS------G----------KVEVADIGMYSP
VALANYDIP--YPVFNLGFGLERVLMIQKGLGDVRSVMYPQFYR--DLKLEDEDITEYIE
IDKTPVSDEGGKLAENIVEIAREHGDDPSPCKFLAYDGRLLG------------------
--KHIKAYVTEKEDNTKLLGPAALNEVYVYDHSIYGVPPG-IGEGMKNYNLLKEIKEKGT
PGGFSYLDACANLFAHEIENAVKRNEK---------------------------------
----------VGFWQIKMAKNPSDLNIVVGGIARRYISSKNKR-IDLRGPVFMSVELMVE
---------------------------------

#3300002231.a:KVRMV2_1001294692
---------------MIYDTKKIKESAK-KDFFATWKKTKGQYPKAS-ESFKDSLLKPGT
--PDPHPIPNTIQKIRSSFLRLGFKEVENQLFIEEQDVYKQYGPEAPAILDRCYYLAGIP
RPEIGLSDSKIKNIKGVVSG--------------------FGSKHTNALKEVLRDYKLGR
IEGDDLVETIEQKLDITTEESTKIIS--QFDELKNLKA--EPTSTTLRSHMTAAWFPTIE
--ALQDKIEHPIKLFSIGLRFRREQKVDATHLRAHYGASCAICDKNMEVESGKAITEQIL
KPLGFRDFNYIKKLA-TSTYYANDSEYEVFAKKG--EE----------NIEIADIGMYSP
IALANYDIK--YPVFNAGFGIERILMITHNLTDVREVMFPQFYQ--TIELSNEEIARAIS
VENQPQTSAGKKLSAAIEKTATKNADAASPCTFEAFSGEFLD------------------
--KNIMVNVVEKEENTKLLGPATLNEIFVYNGGIYGIPKE------GFKEKTKEIKEKGI
NTGITYLRAISDFFASEIETSIQDGK----------------------------------
----------PYSHQFKMAKSAPDLNIKIEDHALKYIQSKKKE-LMLKGPIFMAVEVTLQ
---------------------------------

#CG (Altiarchaeales) archaeon No. 3
---------------------------------------------------MVFKF-KQX
--AKPHPVCEMIEKIRQTFLKFGLSEVINPMIIEESEIYKQYGPEAAVILDRCFYLAELP
RPEIGLSNKKLDKIDKIAPMWREEICSEYSKSSCCCCSQNLDTNSETILKTLLREYKKGN
IEGDNMLEEMVKQLNISAQQASCILD-EVFTELKEIKP--QPTTKTLRSHMTSAWFSVLA
--KAHKKSEIPIGFFSIGPRFRREQKLDATHLYESTTASAVVMAQEFSETEIFSFVEKLC
NNLGFKKINIIRKKA-TAQYYENNKEWEVYALIN--GN----------DVEIADFGLYSK
RALKSYKIT--HSVFNIGFGVERLAMLSTKNNDIRNLVYWHMQE--GVSFSDKELIKQIE
IIEKPNTEQGLKLKELIAKTAKKYAFEKSPCRFNVFEDKFLG------------------
--KKIKVSVVEKEENTKLLGPXCLNNIYAFEGSIYGIPPK------NSFKGTNNIKNKGI
KANFNFLDAISSYFAAKIEERVLQELK--------------------------KTXLPKA
NDKYGKNNKKTNFWQLKMAKTPAEVNIKISEPANRFITSNNKK-IMLRGPIFMSIELKIE
---------------------------------

#CG (Altiarchaeales) archaeon No. 5
---------------------------------------------------MVFKF-KQQ
--AKPHPVCEMIEKIRQTFLKFGLSEVINPMIIEESEIYKQYGPEAAVILDRCFYLAELP
RPEIGLSNKKLDKIDKIAPMWREEICSEYSKSSCCYCSQNLDTNSETILKTLLREYKKGN
IEGDNMLEEMVKQLNISAQQASCILD-EVFTELKEIKP--QPTTKTLRSHMTSAWFSVLA
--KAHKKSEIPIGFFSIGPRFRREQKLDATHLYESTTASAVVMAQEFSETEIFSFVEKLC
NNLGFKKINIIRKKA-TAQYYENNKEWEVYALIN--GN----------DVEIADFGLYSK
RALKSYKIT--HSVFNIGFGVERLAMLSTKNNDIRNLVYWHMQE--GVSFSDKELIKQIE
IIEKPNTEQGLKLKELIAKTAKKYAFEKSPCRFNVFEDKFLG------------------
--KKIKVSVVEKEENTKLLGPACLNNIYAFEGSIYGIPPK------NSFKGTNNIKNKGI
KANFNFLDAISSYFAAKIEERVLQELK--------------------------KTNLPKA
NDKYGKNNKKTNFWQLKMAKTPAEVNIKISEPANRFITSNNKK-IMLGGPIFMSIELKIE
---------------------------------

#Z7ME43 archaea DG-70-1
--------------------MDSKSKRK-EDFESVWKKENKRVYGKK-----GFSLPDKK
--GKRHVLSRYLVKCEEILLDMGFDQVFLKPVWDETHVKMQYGPEAPAILDRLYYLASLP
RPDIAIQQDILDSILNRV------------------------DIDVEGLKQIFRDYKMGA
IDSGELIETFVHRLHISTEDALYILS--MFPELEEIKP--DPTSKTLISHFTTAWFPTLA
--SL--RREPPVLLYTSGWRFRREQKEDSSHLRAHYNLSFVIMGDLAIEDGMEIVKEFFG
RLD--MDVTFTLKEN-QPSYYAYDTNYEVFWE----------------GMEVADIGMFSP
VALAEYGIK--YPVFNAGPGLGRIAMLKEGLQDLRQVHFPELYG---RQYTDEEILESIY
VVKKA---ENQELVKKIVEAARTYKDRESPCTFNVYEDE---------------------
---KVKIELVEEEENTRLIGPAGFNEIYVYNGSIYGVPTA------SSFKGTNNIKNKGI
KANFNFLDAISSYFAAKIEERVLQ------------------------------------
--------ELKKTXRVGMVKSLSDVNLGVPDHVREFIRAQGR--IDVRGPMFTTVVVS--
---------------------------------

#Z7ME43 archaea DG-70
----------------MVDLEKIKEKAK-IDFENTWKGESSRFYGKR-----NFSLPERK
--GKRHVLSEYLLKAEQILLDMGFDQVFLKPIWDQTHVRMQYGPEAPAILDRLYYLASLP
RPDIAISQDIQDTIQKRA------------------------DISIDELKHIFRDYKMGT
IDSGELIETFVQRLQVNTEDALFFLS--LFPELEEIRP--DPSSKTLISHFTTAWFPTLA
--SI--TRELPVLLYTSGWRFRREQKEDRSHLRAHYNLSFVVMGDVGIEDGKEIVREFFK
RLD--MDITFQLKEN-QPSYYAYNTNYEVFWK----------------RMEVADMGMFSP
VALANYGIS--CPVFNAGPGLGRIAMLKEGLHDLRQVHFPELYG---KHYTDEEILESIY
LVKKA---ENQNLVKKIVETAEVYKDKESPCRVTVYKDQ---------------------
---KISVELVEEEDNTRLIGPAGFNQLYVCNGSIYGVPTV------LKNKKIAKILENGV
SNHMSYMEAFANAAVFSSEKGEH-------------------------------------
--------------QVGMVKRLSDINLDIPDHVREFIRAQGK--IDVRGPMFTTVVIS--
---------------------------------

#CG (SCA130) archaeon
----------------MYPVGILKKEAE-EDFYKAWNNSKKLIPLKG----LNYEFPKRR
--GESHILYDYEEKFRQILLNMGFDELILKPFWEDVHVKMQYGPEAPAILDRVYYTASIP
RPDIGISKDREKDIKKISK-----------------------KATIKVLKNIFRDYKMGK
IDPGDLMEELVVKLKINSSEAGKILS--LFPEMEALKP--VPSNNTLLSHFTTAWFPTLA
--SMVKNKNLPVQLFTTGWRFRREQKEDATHLKAHYNSSIVIMNKELSLEDAENITRKVM
SALGFPEVKIVYKEH-NAVYYAPGTDFEVFAETS-GGK----------MLEVADGGLYSP
ISLAKYGIP--YSVFNLGPSLHRTVMIKEGYNDVRNFTYPYEYE--NIELTDKELAGMIH
YDSEPSTEEARMLAEILEKKAIENRGKKGPVSVKVFDDEFMN------------------
--KKVVVEFFEPDKDETLLGSAALNKVIVYNGNVYGLPDK------GLNEEAVLIRKKGV
KTNITFLKGIINSFVKQLENTIINNG----------------------------------
---------KELELRVRMVKRASELNINIKRAAKYYITSKKAV-ISLKGPVFIGIKGIIK
D--------------------------------

#GB archaeon No. 3
---------------MLFDTKKIRDRSK-KNWELEWKKTAELVKGKG-----KFTEIKKR
--GSEHLLWKYIFRIREILLKMGFDEVILNPIQPYEEVKKQYGPEAGAILDRLYYLATIP
RPDIGLSNDKKDLIKKRLPN----------------------FKKFRELQEILKEYKRNE
IEGGEDFTESLVSRLKIKTSDALYLINTVFKELLELKP--EPTNFSLISHATTAWFPLLA
--ELQDKKEHPIMLFSLVWRFRREQKEDKKHLRAHLNFSMVVMDENFKIENGKELTEKFF
KKLGFEDVKFEVKPN-QPAYYAPGTNYEVFVKHK-KIG----------WIEVSEIGMYSP
VSLANYKIK--YPVFNSGPGLGRIVMALENINDIRELYYNKQI-----EFSDQEIAAGII
IDKKPETEEGKKIAEKILTGIIKNKDLIGKIRKKIYEGK---------------------
----VKVYVSEPEKGKKLLGPGGLNEIYVYKGCVLAVKPG--------DKKFEEILKKGV
KVC-SVLESISNYFAYQIEKG---------------------------------------
---------KRGRLTIKYADTLPSINLKMNKEISKYLADNKKE-IKINAPIFIDIEIE--
---------------------------------

#GB archaeon No. 4
---------------MIFDPEKIKNNAK-KDWEREWKKTAELVKGNG-----DFSLNQKK
--GSEHLLWKYALNIRKALLELGFDEVILNPIQSDEEIKKQYGPEARAIMDRVYYISTIP
RPDIGLSNEKKELILKKIPN----------------------FKKFAELQDILKRYKREE
IEGGEDFTETLVSELNISTSDSVYLIDEVFKELTELKP--EASNQTLISHATTAWFPVLA
--KLQDKKMHPIMLFSLVWRYRREQKEDSRHLKAHLNLSLVIMDEKFKIENGKKLTETFF
KKLGFKEVKFKVKPN-QPAYYAPGTNFEVFVKHP-KTG----------WIEVSEIGMYSP
VSLANYKIK--YPVFNSGPGLGRIVMALENIKDIRELYYDSGQ-----ELSDEQIAAGIE
IDKKSENKE---IAEAIKKGIIEHKDSLGIVRKKVYEGI---------------------
----SKVYVLEPEEGKKLLGPGGMNEIYVYHGNILGVKPG--------DEKFKEIRENGV
KVC-SFLDAISNYFAYNVEKG---------------------------------------
---------QKGILTVKYADTLPSINLKMDKSISRYMTDNKKE-IKIAAPIFVDIEIC--
---------------------------------


#Mega
!Title SepCysS alignment;

#Lake Kivu metagenomic archaeon
------------------------------------------------------------
---------MIKMTDEIEPRFVEESFINLNPIQRGGIAS--LAARKAVISYV-DGYSICD
YCL---GCVNKIKKPPVEEFL-TQVSEFLGMDYTILTNGCREAVYAVFHSLLKPDD----
-----TIVVDANRHYTTIVAAENIGLRIFEI-ENSGYPEFKINPDNY-QRVFDEIKNKTG
-KYPKLALLTHIDGSYGNLVDAKKVSKICSDYKIPFLLNAAYSSGRMAVNGKNLGADFIA
TSCHKGWAIGGGNAGFLSITEKWRDKILRVS--------KEYEAKQLEILGCS-TRGSVV
PALLASFPYVTER--IKHWDEEVEKTRFFIKGAEKL-GLKQLGEKPHNHDLVLLETKL-F
HDISKKHKKGGYFLYHELRKRNITGIKPGLTKQFKASIYGLNKEQISYVLDAFKDIIEGR
-------------------

#WOR (Asgard-like) SepCysS
------------------------------------------------------------
------MSIEAKIAGDIEVRTREELFINIQPIQVAGRLT--PEAMKALIAYG-DGYSVCD
WCRK--PFRLDCIKKPPIDKFHKEIAEFLGMDVARAVPGARRGFQAVANTLVEKGD----
-----TVVMSSLAHYTEFLAVEAAGGIVKEI-PPN--EKNIVTGEAT-AEKIEKVKAETG
-KLPKLIMIDHFDYSLGNEHDVYGIGKVAKEYDIPFLYNGAYTVGVMPVDGKKIGADFIV
GSGHKSFASPA-PSGILATTKEWAERVFRKSSMKGDLTGRVFEAKEVEMLGCT-LMGANM
IAMMASFPAIKER--VKHWDEEIKKINYFLEEFLRIEGNKVVSEMPRKHTLTKVDTIESF
HKIAKTHKRKGWFLSDELKKRKIVGMFFGATKTWKLNTYGLTWNQIEYLTKAFIEIAEKY
NIPHTLAKKVEKERKL---

#WOR (Asgard) SepCysS
------------------------------------------------------------
------MDSTELQKYCGISRNIEESFINIHPIQRGGVLT--AEARKVLLDFG-DGYSMCD
FCFE--ARIVKVKKPPVVDFI-QDLATFLDIDEVRPTAGARHAKRAIMEAITEPGD----
-----TIVLDSLAHYTTYITAEAAKLNVKEV-PHSGHPYFLLNVEDY-ATKIEEVEDSTG
-KLPALVVLTHVDYNYGNLSDVKRVAEISHKYGVPFLLNGAYTVGIMPVSGRDLGVDFLV
ASGHKSMSASA-PIGLLGATSEWAEKVFERSKIKGDWSRRTFGAKEIHILGCPPVFGPPL
ATLMASFPKVVER--IKHWDEELEKARYFADNLQKIEGINQLGKKPKEHTLIHFESPS-F
HEVAQNHKRKGFFLYDALKKKKIMGIHPGLSKSFKVNTFGLNFRQVEYIVNSFQEIAVEN
GITIH--------------

#archaeon Odin LCB_4
------------------------------------------------------------
--------MPWDICMLDNIRVVEEDYINLNPIQRAGVLT--SAARKALISYG-DGYSTCD
LCLK-PFRLDKISKPPIGEFY-QELAEWLNMDVVRVMPGARRGFQAVVLAFLKPGD----
----PVAVGSVLSHYTVFLAVEEVGASLAEV-NVD--ENYVIKPEFL-ASKIEEIRRLKG
RDQPKLIILSHVDYLFGNVHPIRELVKVAKEYDVPFLYNGAYTVGVMPVDGKSIGADFIV
GSGHKSMASPA-PTGILATSSEWASKIFSTSSIKGDVSSRSFGIKETHLLGCT-VMGAPL
LAMMASFPYVKER--VKNWDEEVRKAQFFVNEFEKIEGNKVLGQRPRLHTLMKLDTSESF
DKIAQAHKKKGYFLYHELENRKITGIFPGATRNFKINVYGLTWDQIRYVAGAFKDIAVKY
NLRVSE-------------

#BOG (Asgard) archaeon
------------------------------------------------------------
---MDIIKDELRKFQGLLRENSQDQYINLQPIQRGGILP--PESKKTLLSFG-DGYSMCD
FCLK--GRLDMIEKPAISQFL-QLFARWVDMDVSMPTGASRQAKKIILQQLASKVESGVA
-----VLVVDALAHYSTYVAAESAGVEIVEV-PHEGYPDFKIDPDKY-AETIDQARDDRN
-KQVIAALITHADYLYGNITSPEAVGKICKDKGVPFIVNGAYTVGIMPFSGRQCGADFVT
ASGHKSMASSG-PIGMLSCSAEYQDLLFPRSSIKGNWSGRTFKRKITTLIGCPSVYGAPL
ATLMSSFAHVVERTRPENWEREKENARLLADALLRIDGVKMLGMQPHEHTLMQFETKP-F
MDVANNAPKKGFFLYNELKTRGIVGIFPGMVKSMKLNTYGLTTAQVEHVAKSFIEIAEKY
GCTVN--------------

#Archaeoglobus fulgidus 2nd
------------------------------------------------------------
----------MLSKPMELLRPSK-GMINVHPIQRGGILT--EEARKVLLEWG-DGYSMCD
ICLE--GRVDLLDKPPVKRFK-EEVAEFLGMDEVRFTAGARHAKFVAMSGFKG-------
-----ALVVDSLAHYTTYIAAELNGLRVYEV-PNTGYPEFRIEAESY-TDVFEKVKEETG
-DYPAVALLTHADYKYGNLADAKKVAEICRDYGIPLILNTAYTSGIMEVSGRETGCDFIV
ASGHKSWAATA-PIGILATTFEFAERVFRVSEVRGNWSGRAFTKKEVALFGCSPVYGLPL
VTLMASFPVVKER--VKRWKEEVEKARWFVEEMEKIEGVMLLGERPKNHTLMNFETPS-F
NLIAKKHRRKGYFLYHELKERGIFGVQPGMTRNVKLNVYGLSWEEVERVAEAFKEIAEKY
GLEVED-------------

#Geoglobus acetivorans
------------------------------------------------------------
-------MELSRYHPNNLKRISE-GMINIHPIQRGGILT--EEAKKILLSWS-DGYSVCD
VCLE--GRVDLIKNPPINQLK-QDVAEFLGMDTARFTAGARHAKFVIMSAFKGG------
-----TLVLDSLAHYTSYIAAELNDMKIYEV-PNSGYPDFKINPEGY-AQTFERVKEETG
-SYPDIALLTHVDYRYGNLVDAEKVGKICEEYEIPLVLNTAYTSGLMEINGKKLKASFIV
GSGHKSWAATA-PIGILATNYELADRAFQTSKVRGEWSGRAFTKKELSMFGCSPVYGLPV
ITLMASFPKVVER--VKRWDEEVEKARWFVKEMEKIEGLQLIGERPKNHTLMHFESPA-F
HSIAKSHKKKGYFLYHELKKRGVFGVQAGMTKNFKVNTYGLSWEELERVANAFKDIAEKY
GVEVED-------------

#Hadesarchaea archaeon YNP_N21
------------------------------------------------------------
------MVEINVDKYKNLRREIGEDWINVHPIQRGGILP--KETRETLLSFA-DGYSVCD
YCIE--GRVDLIKKPPIADFA-GDVAKFLNMDEVRFTSGARHAQWMVLKSITKPGD----
-----TIIIDSLAHYTTYIAAELNDLNIVEI-PHSGYPEFRINVEDY-ATKIEEVKSQTG
-KLPAAIFLTHVDYRYGNLADAETVGKIAEKYDVPFILNAAYSAGVMPVDGKKLRADFLT
ASGHKSWAASG-PIGILATTYEFSEKTFRTSTIRGPWSGRAFSKKETCSFGCPPVFGAPI
MTLMASFPHVVER--VKHWDEEVEKARWLVGELERIKDFRQLGERPRRHTLMSLETLS-F
HRVAKNLPGKGFFLHRELKKRKITGIQPGLTKIIKLNTYGLSKDQIKRVAEAFHEIAMEH
NIEVE--------------

#MSBL1 archaeon SCGC-AAA261F19 1st
------------------------------------------------------------
------MVEIDLDKYRNLERRVGEDWINIHPIQRGGILP--SESRKALLEFA-NGYSTCD
YCLE--GRVDLVKKPPILDFA-EDVAKFLNMDEARFTPGARGAQRTVFEALTEPGD----
-----TVVLDSLAHYTSYLAAEANDLNVKEV-PHSGYPEFKIRLDEY-ADKIEEAKSETG
-ELPALILLTHVDYRYGNLTDSKKVSKIAEEYGVPFVLNAAYTSGVMPVDGKELGVDFLI
GSGHKSWAASG-PVGILATTYEHADETFRTSMIQGSWSGRSFTKKEVYNYGCPPVFGPPI
ATLMASFPHVVER--VERWDEEVEKVRWLVKELEKIEDFHQLGERPRRHTLFSLETPS-F
HKVSKKRKRRGFFLHEELKERKISGVHPGMTKQIKLNTYGLTKEEIEEVANAFHDIARKY
DVKVEE-------------

#GB (pMC2A209) archaeon No. 9
----------------------------------------------------------MG
RSALGTSVKSARRYSSLSREARKPGIINLNPIQRGGILP--DVARKALDEFG-DGYSLCD
FCTK--GRIDKIRKPPVEDFL-SDLASFLGMDVVRLTNRSREAMFVILMALKRSRGG---
----SAIVVDSNAHYSTYLAAEAAGLSVFEV-PNSGYPEYKVDLGAY-GRVADEVEEETG
-EPPVAIILTHADSHYGNLNDPRPVAALCRKLGVPFLLNAAYTAGVMPINGGKLGADALI
SSGHKSWASSG-PIGIMALSSELAEEVLVPS--------SRFPNKELFLLGCP-VLGAPL
ATLMASFAYVVER--VERWPDEVRKARWLVAELERIEGVRQLGVRPKEHHLICLETPS-F
HEVARRHKKRGFFLYHELKARGIVGIEPGLTRRIKLSTYGLSEDELVKVAEAFQDIARSY
NLSVS--------------

#GB (pMC2A209) archaeon No. 10
------------------------------------------------------------
-------MGKARRYSSLSREAGKQGLINLNPIQRGGVLT--EEARRALSEFG-DGYSLCD
FCLE--GRIDRIRKPPVSDFL-ADLASFLGMDVVRLTNRSREAMFVILMALRKSRGG---
----SAVVLDANAHYSTYLAAEAAGLKVFEV-PNSGYPEFKVELSAY-KDAVDEAEEETG
-EPPVAAILTHVDPHYGNLNDPRPVSRLCRRKGVSFVLNAAYTAGVMPIDGKDLGADAIV
SSGHKSWACSG-PVGIMALTSELADEVLVPS--------SRFPKKELFLLGCP-VLGAPL
ATLMASFAHVVER--VRNWPGEVRKARWLVEELERIEGTRQLGVKPKEHHLICLETLG-F
HEVARRHKKKGFFLYRELKARGIVGIRPGMTRRIKLSTYGLSEEELGVVARAFQEIARKH
GLSVS--------------

#GB archaeon No. 2
------------------------------------------------------------
--------------------MLQKKVLLVDPIRSGGVVP--DGIKRELLEYL-DGYSTCL
YCT---GDLHELRKPNIKKLV-ESLEEFIDCDYVRLVHGAREGMFTVMFAMFKEYVLKGA
R--APVVVADGNAHYTSILAAERSMLDVVFT-ATTEHPEYCVVAEDY-EKKISEVEREHG
-KPPLLALVTYPDGYYGNLPELKRIVDICKSHGVPVLINAAYAIGRMKFSLRDTGADFVV
GSGHKSMACTG-PIGVLGIVGREAEIVLRKS--------KFKEEKEVELLGCT-VRGAPA
ICLLKVLPYLKKR--VVKWDAKVNIARYVADRLEKELEFRLLGERPRRHDLLHFETPR-L
YEISVKIRDR-FFLYKELKKRKIIGIKPGITKRMKISTYLLEKEDANYFVDSLVEIVERF
EKYGFTNR-----------

#Methanocaldococcus jannaschii
------------------------------------------------------------
-----------MDKYKNLTRSLTREFINLNPIQRGGILP--KEAKKAVYEYW-DGYSVCD
YCH---GRLDEVTCPPIKDFL-EDIAKFLNMDCARPTHGAREGKFIVMHAICKEGD----
-----YVVLDKNAHYTSYVAAERAKLNVAEVGYEEEYPTYKINLEGY-KEVIDNLEDK-G
-KNVGLILLTHVDGEYGNLNDAKKVGKIAKEKGIPFLLNCAYTVGRMPVNGKEVKADFIV
ASGHKSMAASA-PCGILAFSEEFSDKITKTS--------EKFPVKEIEMLGCT-SRGLPI
VTLMASFPHVVER--VKKWDEELKKTRYVVDELEKIG-FKQLGIKPKEHDLIKFETPV-L
DEIAKKDKRRGFFFYDELKKRGIGGIRAGVTKEIKMSVYGLEWEQVEYVVNAIKEIVESC
K------------------

#Methanococcus maripaludis
------------------------------------------------------------
-------MDINTDKYKNITRNLEREMINLNPIQRGGIIP--TEAKKIIYEYW-DGYSVCD
YCS---GRLDQIETPPINEFL-EDMSKFLGMDITRPTHGARESKYAVMNSICKEGD----
-----YVVLDGNAHYTSYVALERAKLNYEKT-EIEEYPTFRVIPESY-AEKIDLLEDS-K
-KNIGLILLTHVDGNYGNVADVEKVGKIAKSKGYPFLLNCAYSAGRMPIDGKKLNVDFIA
ASGHKSMAASG-PCGLLSINKKYEDEVLETS--------KVNVVKELQMLGCT-SRGIPI
LSLMASFEHLIER--VKKWDLEVEKTRKVVNELEPLG-FNQIGEKPRNHDIIRFETPI-L
DKIAEKDKRRGFFFYEELKKRGIGGIRRGVTKEFKMSVYGLTNVQVDYVINSMKSIINEL
R------------------

#Methanothermobacter thermautotrophicus
------------------------------------------------------------
---------MECADYG-LTRKLERDNLNLNPLQRGGVLP--AAARKALHEFG-DGYSVCD
YCD---GRLDQVTRPAINCFL-DDLADFTGSDAVRTVHGAREGKFAVMHALCERGD----
-----TVVVDGNAHYTTHLAAERNGLEIVEV-PSTGHPSYEVTPEAY-REVLEETID--R
-VEVKLAVLTHVDGNYGNLTDARGVADVCRKLGVPLLLNCAYSMGRLPVNLRELGVDFVV
GSGHKSMAASG-PIGVLGMKSEWEDTVLRRS--------GRHEKKELELLGCT-SRGAPL
ATLMASLPYVRER--VSRWDGEVKKTRYLVSELEDIGGIEQLGVRPKEHDLVRFETPV-F
HEIAASHPRKGFFLYEELKKRGIVGIRRGQTKWFKCSIYGMTEEQVQYVVDSFRDIVEEN
R------------------

#Methanopyrus kandleri
------------------------------------------------------------
---------MNLDRYRNIVRETERKYINVNPIQRGGVLT--PEARKALLEFG-DGYSVCD
FCE---GLLHEIEKPPIRQFH-EDLAEFLGMDVVRITAGARYAKEAVMSALCEEGD----
-----VVVADSLAHYTTFVAAEKAGATVREV-PNTGHPEYKVKVDEY-ARVIDEVEDERG
-DPPALALLTHVDSEYGNLADAEKFVKICRKKGVPALLNCAYTMGRMDLSNLSPKPDFMV
GSGHKGMAACA-PCGVLAMREEWEEEVLRGSSLRGDVSGREWPHKEVEMLGCT-VMGAPI
VTMMASFPHVVER--VKRWKEEVRKTRWFVKEMERIEGVRQLGERPKRHDLVKFETPG-F
HEVAEDHPRRGYFLYEELKKRGVIGIQPGQTETIKASVYGLTDEQVEHVVRAFHEIAEEY
GLEVS--------------

#Archaeoglobus fulgidus 1st
------------------------------------------------------------
-----------------MFKRETKDFINIDPLQTGGKLT--EEARQALLEWG-DGYSVCD
FCTT--GRLDEIKTPPIHDFIHNQLPKFLGCDVARVTNGAREAKFAVMHSLAKKDA----
-----WVVMDENCHYSSYVAAERAGLNIALV-PKTDYPDYAITPENF-AQTIEETKKR-G
-E-VVLALITYPDGNYGNLPDVKKIAKVCSEYDVPLLVNGAYAIGRMPVSLKEIGADFIV
GSGHKSMAASG-PIGVMGMKEEWAEIVLRRS--------EKYKNKEVELLGCT-ARGATI
ITLMASFPHVRER--IKRWDEEVEKARRFAAEMEKL-GIKQLGDNPHNHDLMFFHAEV-L
YEISKKAKGGRFFLYRELKSRKIHGIKPGLTRYFKLSTYGLSDEEVDYVLNAFKEIIEKY
S------------------

#Archaeoglobus veneficus
------------------------------------------------------------
------------MPKFGFIERQTKDFINIDPLQTGGKLS--EEAKKALVEWG-DGYSVCD
FCT---GRLEEIKTPPIYDFVHETLPEFLGCEVARITNGAREAKFAVMHALARPDA----
-----WVVLDGNAHYTSYVAAERAGLNVAEV-PNTGHPGFRIEPDRY-AEVIEETKKK-G
-D-VVLAVLTYPDGNYGNLPDAKRIAKICHEYDVPLLLNCAYAVGRMPVKMKEIGADFIV
GSGHKSMASAG-PIGVLGMREDYSSALLRKS--------AKYKKKEIEFLGCT-ARGVTI
MTLIASFPHVVER--VKQWDKEVEKARWFSAKMEEL-EIVQLGDKPHNHDLMFFESQK-L
YEISKKAKKGRYFLYKELKKRKIHGIKPGLTKHFKLSTYMVPKEQLEIVIRAFEEIIDRY
S------------------

#Methanosarcina mazei 1st
------------------------------------------------------------
-----MTLDDSSLQKFGFIKRETLGSINIDPLQTGGLLT--GAAKQALVEWG-DGYSVCD
FCG---GVLDLVKKPPIHDFVHKALPEFLGCDEARVTNGARESKFAVMHSMGKPGD----
-----WVVLDGLAHYSSYVAAERAGLNIEVV-PHAGSPEYHLDPGRY-GKAIEEVTKENG
-KPPVLALVTYPDGSYGNIPDAAKIASVCHEYDVPLLLNGAYSVGRMPVSAKEIGADFIV
GSGHKSMAASG-PVGVLGVSEEYAPVVFRKS--------VHNKVKEIELLGCT-ARGATV
MTLMASFPEVVKR--TRNWDQEVENARWFSSRLEGM-GFIQRGQKPHSHDLMFFEAPG-F
YEISQKVKNGRYFLYRELKERNIHGIKSGLTKYFKLSTFGLGKEKLGTVADAFEDILKKY
ENI----------------

#Altiarchaeales archaeon WOR_SM1_79
------------------------------------------------------------
-----MENLIQQSQKFKDLHREALKYINLNPLQRGGILS--DAARKIVNEWA-DGYSVCD
YCG---GCLDLIKNPPIEEFVHDKLPKFLGTYAARLTNGAREGKFAVMHSLCEKGD----
-----TVLFDSNAHYSSFVAAERVGAEVVTV-PNSEYPEFKIDENLY-AEKIEEIKK-AG
-KEPKLALLTYPDGNYGNLADAKKVGKICREYGVPFLLNGAYSVGRMPVNAKKLNADFVV
GSGHKSMAASG-PIGVLGTTSEYEDVLFRKS--------PKYKIKEIELLGCT-SRGLPV
ITLMASFPHVLER--IRGWDEEVSNARWFLDKISGIPGIKPLGIQPTEHDLNFIETPV-F
YEISKKHKKRGYFLQKELKARGIVGIKPGLTNFFKLSTYLLTTDEIKTVADAFLEIYEKY
EDLLE--------------

#Altiarchaeales archaeon WOR_SM1_86-2
------------------------------------------------------------
-----MEELILQSQKFKDLHRETAKYINLNPLQRGGILS--DAARKIVNEWA-DGYSVCD
YCG---GCLDLIKKPPVEEFVHDKLPKFLGTDTARVTNGAREGKFAVLHTLCEKGD----
-----TVLFDSNAHYSSFVAAERVGAEVVTV-PNSGHPKFRIDEDSY-ADKIEEIKKDTG
-KYPKLALLTYPDGNYGNFSDAKKAGKICHEYDVPFLLNGAYSVGRMHINGKKLNADFVV
GSGHKSMAASG-PIGVLGTTSEYEDILFRKS--------AKYKIKELELLGCT-SRGLPI
ITLMASFPHVLER--IKRWDTEVANARWFLNKISEIPGIKPLGVQPTEHDLNFIETPV-F
YEISKRHKKRGYFLQKELKSRGIMGIKPGLTKFFKLSTYLLTTDEIRLVADAFFEIYEKY
EGLLK--------------

#Altiarchaeales archaeon CG_SM1
------------------------------------------------------------
-----MKIQNSQLEKFKNLHRKSFKFINLDPLQRGGILS--KEARKILDEWA-DGYSVCD
YCG---GCLDKIKNPPIEEFVHEILPEFLGTDIARITNGAREGKFLVMHAIAKEGD----
-----YVVMDQNAHYSSYIAAERSRLKVKFV-PNSGYPEFKINVDDY-AKVIDEVKNKTG
-KFPALVLLTYPDGSYGNLPDAKAAGKICKDYNVPFLLNGAYSVGRMEINASDFNADFIV
GSGHKSMAASG-PIGVLGAKKIYEEILFRKS--------ERYKIKEIEQLGCT-ARGLTV
ITLMASFPHVYER--VKHYDEEIRKARYFSEKIGNL-GINQLGEKPHNHDLMFFESQK-L
FEISQKHKQGRFFLYSELKKQGIIGIKPGLTKNFKLSTYALSDEEIKKVIDAFGEIVKLD
QLCAK--------------

#Wilbur Geyser a1
------------------------------------------------------------
--------MESNFQKFKKLHREAINFINLNPLQRGGILS--EEARKILNEWA-DGYSICD
YCD---GRLDHIKNPPIEEFVHKILPDFLGTDTARITNGAREGKFLIMHAIAKEGD----
-----YIVMDQNAHYTSYLAAERLGLKIKLV-KNSGYPEFKIKADDY-ADAIEEVKKESG
-KPPSIALITYPDGNYGNLPDAKKIGKICHEYDVPLILNGAYSVGRMPVNAKNLNADFIV
GSGHKSMASSG-PIGVIGTSKEYEKILFKKS--------EKYKIKEIELLGCT-SRGLPI
ITLMASFPYVAER--IKHYDKEVETARYLSKNFENL-GINLLGEKIHNHDLMFFESKK-L
FEISNRHKKGRFFLYYELKKRGIV------------------------------------
-------------------

#CG (Altiarchaeales) archaeon No. 3
---------------------------------------------------MTFQKTNLD
HKNPSIINHNQENHNKINLNRENLGLINLNPLQRGGIIP--LETRAILNQWL-DGYSVCD
FCR---GCLDKIQRPPIETFVHKTLPKFLGCDEVRITNGAREGKFMIMHTICEKGD----
-----WILIDRNAHYSTFVAAERAGLNIEKT-PKTSAPEHKINETDF-ASKIKEMET-AG
-RKPKLALLTYPDGNYGNLPDAKKVAEICRNAEVPLIINGAYAVGRMPINLKEIGADFIV
GSGHKSMAASG-PIGVVGATKKWAEQVFAKS--------KNYPLKGVEELGCT-SRGHSI
ISLMASFPYVIKR--VANWDKEVENARYFSDEITKIRGIKAIGEQPHNHDLMAFETLV-F
YEISTTHKRRGFFLYDELKARGITGIKPGLTKNFKLSTFGLTRKEIDKIIGAFKEIAKIK
-------------------

#CG (Altiarchaeales) archaeon No. 5
---------------------------------------------------MTFQKTNLD
HKNPSIINHNQENHNKINLNRENLGLINLNPLQRGGIIP--LETRAILNQWL-DGYSVCD
FCR---GCLDKIQRPPIETFVHKTLPKFLGCDEVRITNGAREGKFMIMHTICEKGD----
-----WILIDVNAHYSTFVAAERAGLNIEKT-PKTSAPEHKINETDF-ASKIKEMET-AG
-RKPKLALLTYPDGNYGNLPDAKKVAEICRNAEVPLIINGAYAVGRMPINLKEIGADFIV
GSGHKSMAASG-PIGVVGATKKWAEQVFAKS--------KNYPLKGVEELGCT-SRGHSI
ISLMASFPYVIKR--VANWDKEVENARYFSDEITKIRGIKAIGEQPHNHDLMAFETLV-F
YEISTTHKRRGFFLYDELKARGITGIKPGLTKNFKLSTFGLTRKEIDKIIGAFKEIAKIK
-------------------

#Altiarchaeales archaeon MSI_SM1
------------------------------------------------------------
-----MEIQNSQLEKFKELHREALKFTNLDPLQKGGILS--KEARQILDEWA-DGYSVCD
YCG---GSLDRIKNPPIEEFVHEILPKFLGTDIVRLTNGAREGKFLIMHAIAKEGD----
-----YIVMDQNAHYSSYVAAERARLKVKFV-PNLGYPEYKINADDY-AKVIEEVKNETG
-KFPALVLLTYPDGSYGNVPNAKAVGKICNEYKIPFLLNGAYSVGRMEINTSDFNADFIV
GSGHKSMAASG-PIGVLGAKKEYEEILFRKS--------EKYKIKEVEQLGCT-ARGLPV
ITLMASFPYVYER--VKHYDEEVRKARYFSEKVGNL-GINQLGEKPHKHDLMFFESQT-L
FEISQKHKRGRFFLYDELKKRRIIGIKPGLTKNFKLSTYLLSDEEIKKIVEAFEEIVNLN
R------------------

#Methanosaeta thermophila 1st
------------------------------------------------------------
-----MPSSITSKLDKFRCMKRSISEINLDPLQRGGILT--PEARAALVEWG-DGYSVCD
FCE---GMLDRVRNPPVEEFVHEILPRFLDVDDVRITHGAREGMYAAMHSLCDAGD----
-----YIVADGNAHYTTVLAAERAGLEISYV-ENSGEPEYTINPEGY-REAIRDAADQ-G
-YRVSMAVLTYPDGNYGNLVDARRVAEICHEEGVPLLLNGAYSIGRMPVSARELDADIVV
GSGHKSMASSG-PIGVLGAKQEYADIIFRRS--------KSKKNKEVELLGCT-VRGAGL
LTMMASFPHVLER--VGRWQEEVDKARWFSSELEEL-GIEQQGEKPHNHDLMFFKSER-L
YEISKSARKGRYFLYHELKDRGICGIKPGLTRQFKLSTYLIPREDLERVVSAFHEIIEMG
SRVEAE-------------

#Lake Sakinaw b1
------------------------------------------------------------
--------MGKLLQKLTKLHKSHKGLINLMPLQTGGSLS--DAAREALIEFG-DGYSVCD
FCL---GSLCNIANPPVREFVHELLPEFLGCEVATLTYGARDSMFMVMHSLTQPGD----
-----SIVVDGNRHYTTIVAAERVGLNVFEV-PHSGYPEFRIDVNDY-VPFIEKYN----
---PKLILLTYPDGNYGNLADAAKLGEIAQEYDSPYVLNGAYSVGRMPIKMNGIGADFLV
ASGHKSMASAG-PCGILGMKRKWEETVLRKS--------KTYNNKEIELLGCT-VRGVPL
ITLMASFPCVKER--INYWDKQVAKAQWFSEELEKL-GFKQLGEKPHRHDLLHFETPM-F
YEISKHVRERGYFLYEELKGRGIWGPQPGMTKAFKISTFAADEGQLGFVVDSFKAILSKY
S------------------

#Crude oil b1
------------------------------------------------------------
----MGQARSKSLEKLSKLHRPHKSLINIMPLQTGGLLT--QAAREALIEFG-DGYSVCD
FCL---GNLSNITNPPIRDFTHEFLPPFLGSEVATITHGAREGIFMVIHSLTQPSD----
-----SIIVDGNRHYTTIVAAERVGLNVIGV-PNSGYPEFKINVDDY-VPLIEKHK----
---PKLILLTYPDGSYGNLPDAKKLGQIVQKYDIPYILNGAYSVGRMPVNMADIGADFIV
GSGHKSMASAG-PCGVLGMKKKWEDIVLRKS--------QAYRNKEIELLGCT-LRGVPL
ITLMASFPYVQER--VLHWDEQVPKAQWFSAELEKL-GFKQLGEKPHRHDLLYFETPI-L
YDISRHVRERGYFLYKELKERGIWGPQPGLTKAFKVSTFAADRKQLGFVIDSFKTILSKY
S------------------

#Deep marine Sb2
------------------------------------------------------------
-------MKDKTLQKLGELHRKHKDIITLMPLQTGGLLT--EAAKKALIEFG-DGYSVCD
FCT---GNLSNIANPPIREFVHELLPEFLGCEVAAVTHGAREAKFMIMHSLARLGD----
-----SIVVDGNRHYTTIVAAQRAGLNVVEV-PSSGYPEFTIDVNEY-EPFIEKHH----
---PRMILLTYPDGNYGNIPDAKRLGRIARNHDVPFILNGAYAVGRMPVNMNELGADFVI
GSGHKSMASAG-PCGVLGMKKRWEEVVLRKS--------ETYTNKELELLGCT-LRGVPV
ITLMASFPYVLKR--VDDWDEQVLKAQWFSSEVEKL-GFKQLGEKPHRHDLLHFETPV-L
YEISKRVRERGYFLYKELKDRGIWGPQPGLTKAFELSTFAASREQLSLVVDSFKSILSKY
S------------------

#Dehalococcoidia bacterium CG2_30_46_19
------------------------------------------------------------
-------MKDKRLQKLGELHRKHKDIITLMPLQTGGLLT--EAARQALIEFG-DGYSVCD
FCS---GSLCDITNPPVQEFVHELLPEFLGCEVATITHGAREAKFAIMHSLAKPGD----
-----SIIVDSNRHYTTIVAAERVGLNVIEV-PSSGYPEFTIDVNEY-EPLIEKYH----
---PRMILLTYPDGNYGNIPDARRLGDIAQEHDVPFILNGAYAVGRMPVNMNELGVDFVV
GSGHKSMASAG-PCGVLGMKKRWEEVVLRKA--------EAYSNKEVELLGCT-LRGVPL
ITLMASFPYVVER--VNHWDEQVLKAQWFSNELEKL-GFKQLGEKPHKHDLLHFEAPM-L
YEISQRVRERGYFLYKELDDRGIWGPQPGLARSFKLSTFAASKEQLSFVVDSFKSILSKY
S------------------

#MTBE-degrading b1
------------------------------------------------------------
---MTKQAENKSLQKLANLQRQHKGLINLMPLQTGGILT--DAAREALIEFG-DGYSVCD
FCE---GILCSVTNPPIRDFVQELLPQFLGCEVATITHGAREAKFMVMHSLAQPGD----
-----SILVDANRHYTTVVAAERVGLNIIEL-PNSGHPEFRVDVDEY-IQFIKKHA----
---PKLILLTYPDGNYGNLPDAGRLGEIAQEYNIPYILNGAYAVGRMPISMDEIGADFII
GSGHKSMASAG-PAGVLGMKKKWEASVLRKS--------GKYSKKEIELLGCT-IRGVPL
ITLMASFPYVKER--VNHWDEQASKAQWFSAELEKL-GFTQLGERPHRHDLLHFDAPA-L
YEISKRVRERGYFLYKELKERGIWGPQPGMARSFKLSTFAADRQQLGFVIDSFKAILNKY
S------------------

#Chloroflexi bacterium RBG_13_51_36
------------------------------------------------------------
----MEQESSKSLQKLRGLRREHKELINLMPLQTGGILT--NAAREALIEFA-DGYSVCD
FCQ---GSLCNVSNPPIRDFVQELLPQFLGCEVATVTHGAREAKFMIMHSLARPGD----
-----SILVDANKHYTTVVAAERVGLNVIEV-PNSGHPEFKVDVNEY-APLIKEHA----
---PKLILLTYPDGNYGNLPDAKRLGEISQEYDIPYVLNGAYAVGRMPLSMEQIGVDFIV
GSGHKSMASAG-PAGVTGMKKKWEEVVLRKS--------GKYRNKDIELLGCS-IRGVPL
ITLMASFPSVKER--VQHWDEQVSKAQWFSAELEKL-GFRQLGEKPHRHDLLHFDAPA-L
YEISKRVREKGYFVYKELKERGIWGPQPGLARSFKVSTFAVDRQQLGYVIDSFKAILSKY
A------------------

#Deep marine Sb1
------------------------------------------------------------
----MEQKDSKLLQKLSGLRREHKELINLMPLQTGGILS--DAAREALTEFA-DGYSVCD
FCQ---GVLCNISNPPIRDFVQGLLPPFLGCEVATITHGAREAKFMVMHSLARPGD----
-----AILVDGNRHYTTFVAAERVGLDVIEV-PSSGHPEFRVDVNEY-ASLIKQHA----
---PKLILLTYPDGNYGNLPDARRLGEIAQEYNIPYVLNGAYAVGRMPVSMEEMGADFII
GSGHKSMASAG-PVGVLGMKKKWEETVMRKS--------GRHSNKDIELLGCT-VRGVPL
ITLMASFPHVKER--VNHWDEQVSKAQWFSAELEKL-GFIQLGEKPHRHDLLHFDTPA-L
YKISKRVRERGYFLYKELKERGIWGPQPGMASSIKLSIFAADKEQLGFVIDSFRAILNKY
S------------------

#Hot spring Yb1
------------------------------------------------------------
----MEQKPSRSLQKLSNLQREHKGLINLMPLQTGGILT--ESAKQVLVEFG-DGYSVCD
FCQ---GNLCSVTNPPIRDFVEELLPQFLGCEVATITHGAREAKFIVLHSLTQPGD----
-----SIIVDANRHYTTIVAAERAGVNVIQV-PNSGYPEFRVDVEEY-IPLIRKHA----
---PKLILLTYPDGNYGNLPDAKRLGEIAREYDIPFVLNAAYAVGRMPINMKEIGADFVI
GSGHKSMASAG-PCGVLGMRKKWQDKVLRKS--------STYSNKEIELLGCT-LRGVPL
MTLMASFPHVTER--VKGWQDQVVKAQWFSAELEKL-GFKQLGEKPHRHDLLHFDAPA-L
YEISKQVRERGYFLYKELKEKGIWGPQPGLAKSFKLSTFAADRQQLQFVINAFRAILDKY
SSR----------------

#Alkali sediment b2
------------------------------------------------------------
---MAEQAASRPSQQLSNLRRQHKGLINLMPLQTGGLLT--TTARQALVEFG-DGYSVCD
FCQ---GSLCNITNPPIREFVQELLPSFLGCEAATITHGAREAKFMIMHSLARPGG----
-----SIIVDANRHYTTVVAAERAGLKVIEV-PHSGYPEFTVDVKQY-APLIEEHS----
---PDMILLTYPDGNYGNLADAGGLGQIAREYGIPYILNGAYAVGRMPVSMEEVGADFVV
GSGHKSMAAAG-PAGVLGMKRKWEEVVLRRS--------GTYGNKEVELLGCS-IRGVPL
MTLMASFPSVAER--VRDWNEQVSRAQWFSAALEDL-GFKQLGERPHRHDLLHFETPA-L
YEISRRVRERGYFLYKELKERGIWGPQPGLARSIKLSTFAADRQQLQLVIESFKAILDKY
G------------------

#Crystal Geyser b4 1st
------------------------------------------------------------
----MKQPTNKALQKLGALHRLHKGLINLMPLQTGGILT--DAAREALVEFG-DGYSVCD
FCL---GNLCNITNPPIKKFVHELLPQFLGCEVATITHGAREAKFMVMHSLAKPGD----
-----SIIVDGNRHYTTIVAAERAGLNVIEV-PHSGYPEFRIDVNDY-IPVIKKHN----
---PKLILLTYPDGNYGNLPDARKLGEIAQEYDIPYILNGAYAVGRMPVKLSDVGADFII
GSGHKSMASAG-PCGVLGMKKRWEEIALRKS--------KAYSNKEVELLGCT-VRGAPL
VTLMASFPYVKER--VNHWDEQVLKAQWFSAELEKL-GFKQLGEKPHGHDLLHFEAPM-L
YDISKRVRERGYFLYKELKERGIWGLQPGLTKAFKLSSFAADKEQLALVIDSFKAILDKY
S------------------

#Lake sediment b3
------------------------------------------------------------
----MEQKSSKSLQKLSELRRQHKSLINLMPLQTGGMLT--DAAREALIEFG-DGYSVCD
FCQ---GSLCNITNPPIQEFVQELLPHFLGCEVATITHGAREAKFMVMHSLARPGD----
-----SILVDANRHYTTFVAAERVGLNVIEV-PNSGYPEFKIDVNEY-TPLIKKHV----
---PKLILLTYPDGNYGNLPDARRLGEIAQEYDIPYVLNGAYAVGRIPISMEEMRADFII
GSGHKSMASAG-PAGVLGMKKKWEKTVLRKS--------GTYSNKEIELLGCS-IRGVPL
ITLMASFPHVKER--VQHWDEQVAKSQWFSAALEEV-GFKQLGEKPHRHDLLYFDAPM-L
YDISKRVRERGYFLYKELKDRGIWGPQPGLARSFKLSTFAADRRQLGFVIDSFKTILNKY
S------------------

#Crystal Geyser b4 2nd
------------------------------------------------------------
----------MATQEFSSFKREQKDLINLMPLQTGGILT--DAAREALVEFG-DGFSTCD
FCL---GNLCNITNPPIKKFAHEPLPRFLGCDVATITHGAREAKFMVMHCLTNPGD----
-----TIIVDGNRHYTTVVAAERAELNIIEV-PSSGYPEFKVNVADY-IPLIEKHH----
---PKLILLTYPDGNYGNLPDAKRLGGVAAKYNVPYILNGAYAVGRMPTSMMEIGADFII
GSAHKSMASAG-PCGVLGMKKKWENILLRKS--------AVYKKKEVELLGCT-VRGVPL
ITFMASFPYVKER--VNHWDEQVAKAQWFSQELENL-GLKQLGGKPHRHDLLHFDAPA-F
YEISQHVPERGYFLYKELKKRGIWGPQPGLAKSFKVSTFAASKEQLGFVIDSFKDILQKH
HSKAV--------------

#Alkali sediment b1
------------------------------------------------------------
----MPQP--ADLERLTTLARPHRDMINIMPLQTGSILT--DAARRALVEFG-DGYSVCD
FCQ---GRLTDIANPPVRQFVNEMLPEFLGSERATLTYGARDGVFMVLHSLTKPGD----
-----VVVVDGNRHYSTAVAAERAEVELVTV-PNSGYPEYTVDAEGFDVALSKQHK----
---VALVILTYPDGRFGNLADAPRVGEIAESLGIPYLVNGAYCVGRMPVRMSELHADFIV
GSGHKSMASAG-PSGVLGMKARWEEVVLRRS--------SLHKTKEVECLGCT-VRGVPL
VTLMASFPSVVER--VQHWDEQVAKAQWFSAAMEEI-GFRQLGEKPHRHDLMVFETTA-L
YDISQRVRERGYYLYKEMEARGIWGVKPGQTKALELSTFAATREQLKQVVEAFQDILEKY
R------------------

#CG (Woesearchaeota) archaeon
------------------------------------------------------------
----MNSTIQELQQRYANLTRTYNQGIIVNPLQTGGRLT--DAAKAAMQQFG-DGYSVCD
HCK---GRLEGISNPPIDTFVEKDLPQFIGADAVKLTHGAREGKYLVFHAITKPGD----
-----VVVVDRNRHYSSDAAIQRAGLNVVKV-DNDGTLERKMNVEDY-IPLIKQHR----
---PKLLFITYPDGSVGNLPDVKRLGDIAQEYEIPLVVNAAYAIGRMPISLQDIGADFVI
GSGHKSMASMG-PVGVLGMKEKWRDILTRPV--------EGHKGKEIECLGCT-VRGTPL
ITLMASFPEVVER--TGRWDEEVAKARWFSGELESL-GLEQMGEKPHAHDLMMFQTDV-F
YQISGKHPQKRAFLYEALKENGIFGVKHGLTKSMKISTYGTSREDLEKVVATFKDLVNRY
G------------------

#CG bacterium No. 3
------------------------------------------------------------
------MKVEDLKRKYGNLRRPYRDFIDLNPLQTAGRLT--DAARSSLLEFG-DGYSVCD
QCK---GTLEKIASPPILGFVHDDLPEFIGADIVKITHGAREGKFMVMHVVTSLGD----
-----VILVDENRHYSTDVAAQRAGLNIVKV-SNSGDPERLIDVEDY-VPLIKQHR----
---PKLILLTYPDGNVGNLPDAKRLAEISKEYEVPYLLNAAYAIGRMPISHHEIGADFIV
GSGQKSMASAG-PIGILGFDKKWAEIVAAES--------PTHKGKDIECLGCS-VRGIPL
ITLMASFPYVVER--IQHWDEEVQKARWFSEQLEVL-GLVQLGEKPHKHDLMMFETDI-F
YEISKVHPKKRALLYEALRDNGIHGLKHGNTKSMKISTYGTPKEDLKKVIDVFEEIVDTY
-------------------

#CG (Parcubacteria) bacterium
------------------------------------------------------------
-------------KKFINLKRETRGMINIDPLQAGGILT--DEARQALVEWG-DGFSVCD
FCQ---GQLSEIKNPPIYDFVHQILPEFINADAICLTNGAREGKFMVMNAIAKPGD----
-----VIIIDKNAHYTTYVAAERAGLKIKEV-ENSGYPEFKIDVNKY-EEAIKEIQKEKG
-N-IVLLLLTYPDGNYGNLPDAKKLAEISKKYNIPFLLNTAYAIGRMPINMNEIGVDFVI
ASGHKSMASSG-PIGFIGMKKQWEDILMKKS--------QYFPKKEIENLGCT-ARGTAI
MTLMASFPKVVER--IKQWQEQVEKARWFSEEMEKL-GLKQLGEKPHNHDLMFFESKK-L
FEISKKHKKGRYFLYQELKKNNIWGIKPGLTKNFKLSTFSASKEELKKILKTFEELRDEK
L------------------

#GB archaeon No. 3
------------------------------------------------------------
----------------MQLKRSKF-EINLNPIQRGGILT--KEARDALIEFA-DGYSVCD
FCP---GRLDLIEKPNIKKFVHEELPEFLDCDLARTTNGAREAMFAVMNSLKVAEKNK--
---SKIVLADGNAHYSSYIAAERAGLDIDIV-ESSGYPEYKIDVEKF-EEKIDLNK----
---PVLVLLTYPDGNYGNFPDVKKLSKICKNKGIPLLLNCAYSIGRLEIKLNKLGVDFLI
GSGHKSMAASG-PIGILGFNKEWEKIILRKS--------RYFPNKEIEMLGCS-SRGPSI
ITLIASFPTIRER--IKYWDEEVEKARYFSKEMEKL-GLKQLGEKPHNHDLMFFEAKP-I
YEISKKHKKRGYFLYHELKKRGIGGIKPGLTKNFKVSTYQLTKEEIDRVVKAFEEIVK--
-------------------

#GB archaeon No. 4
------------------------------------------------------------
----------------MLLKRQKS-EINLNPIQRGGILT--KEGREALIEFG-DGYATYD
ISP----GLEIEEMPIIKEFLKKELPEFLGADISKFTLGAREGIFNVMHCITKPGD----
-----TIIVDGNRHYTTITAAERCKLKIIEV-KNSGKPDYKINVEDY-EDLIKKHK----
---PSMILLTYPDGNYGNLPDAKKLGKVAEKYKVPYLINGAYSVGRMPVSMKEIGADFII
GSCHKSMASSG-PLGILGAKKKWEKILFKKS---------ELVNKEIEFIGSE-SRGTAV
ATLIASFPKVKER--IKNWDKEVKKARYFSSEMEKL-GINQLGEKPHNHDLLFFEAPT-L
YKISQKHKKRGYFLYHELKKRRITGIKPGLTKNFKISTYLLTKEEIDKVISAFREIIEKN
L------------------

#Parcubacteria DG_74_2 bin
------------------------------------------------------------
----------------MIYKRQNKNKININPIQAGGILT--KDARKTLIEWG-DGYSVCD
IWYS--GKIDKIENPQIRKFINEDLPKFLGSDIARIIGGAREGICAIMHAVAKPGD----
-----IILVDENKHYTTILAAEKNGLKVVEV-PNSGHPEYKIDVRDY-EKLIKKHK----
---PALILLTYPDGNYGNMPDAKKLGEIVIKYNIPYLLNAAYSAGRLPVDLIAINGDFIV
ASGHKSMAASE-PIGVLGFRKKWKDTLFKKS--------FFYPDKEIEFLGHY-QKGAPM
MTLMASFPYVKKR--VEEWEKQIEKARWFSAEMEKL-GFKQLGEKPHNHDLLFFESPQ-L
YKISQKHKEGRFFLYKELKKKGIYGIKPGLTKHFKLSTFAASKEELKKLLEVFKEILIK-
-------------------

#Arc I group archaeon ADurb1013_Bin02101
------------------------------------------------------------
-----------MNIGKLELRDREESFINLNPLQTGGRTT--SDARKAIISYI-DGYSICD
WCK---GALHITTEPDIAGFLGEVS-DFLGMDYALPTNGCREAKYGVMHAISTGG-----
-----SILCDGNMHYSSEVSAERAGLKIYKV-PNKGEPEYKINPEDY-SEGFELIKKETG
-KYPELALLTHVDGEYGNVVDAKEVGKICNDYSVPFLLNTAYSSGRMEIDGKKIGADFIA
CSGHKSWAAGGGNIGILAIKEGWQDKVFKPG--------ANWKSKPLEILGCS-SRGASL
LALMASFPHVKER--VKKWDEEVKKARYLVNELEKI-DIKQIGEKPKNHDLIKLDTPV-Y
DNIAKTHKKKGYFLYYELKDRGIIGMKAGRTRKFKISTYGLSWEQVSYVAESFLEIGGG-
-------------------

#Arc I group archaeon U1lsi0528_Bin055
------------------------------------------------------------
-----------MNIGKLELRDREESFINLNPLQTGGKTT--SDARKAIISYI-DGYSICD
WCK---GALHITTEPDIAGFLGEVS-DFLGMDYSLPTNGCREAKYGVMHAISSGG-----
-----SILCDGNMHYSSEVAAERAGLKIYKV-PNGGEPEYKLIPSGY-AEGFETIKKETG
-KYPELALLTHVDGEYGNVVDAKEVGKICNDYSIPFLLNTAYSSGRMEIDGKKIGADFIA
CSGHKSWAAGGGNIGILAIKEGWQDKVFKPG--------TNWKSKPLEILGCS-SRGASL
MALMASFPHVKER--VKNWDEEVKKARYLVNELEKI-DIKQLGEKPKYHDLIKLDTPV-Y
DNIAKTHKKKGYFLYYELKEKGIIGMKPGRTRKFKISTYGLSWEQVSYVAESFLEIGGG-
-------------------

#Arc I group archaeon U1lsi0528_Bin089
------------------------------------------------------------
-----------MKIGKLELRDREESFINLNPLQTGGRTT--ADARKAIISYV-DGYSICD
WCK---GALHITTEPDIAGFLGEVS-DFLGMDHALPTNGCREAKFGVMHAISTGG-----
-----SILCDGNMHYSSEISAERAGLKIFKV-PNNGEPEYKINPSDY-AEGFENIKKETG
-KYPELALLTHVDGEYGNVVDAKEVGKICNDYSVPFLLNTAYSSGRMEIDGKKIGADFIA
CSGHKSWAAGGGNIGILAIKDGWQEKVFKPG--------TNWKSKPIEILGCS-SRGSSL
LALMASFPHVRER--VKKWDEEVKKSRYLVKELEQL-DIKQLGEKPKNHDLIKLDTPV-F
DEIAKTHKKKGYFLYYELKDMGILGMKPGRTRKFKISTYGLSWDQVKYVSECFLKIGGG-
-------------------

#Ca. Bathyarchaeota archaeon BA1
------------------------------------------------------------
----------MSKGILSEWRAREELFINLEPLQRGGVVP--AEARKVALSYV-NGYSTCD
HCL---GTLHILKKPPICDFLIQVA-EFLGMDRAIITHGCREAKFAVMHAITKPGQ----
-----AIVMDENKHYTSYVAAERAGLKIYEV-PSTGHPKFRVEPEEY-ISAVEKVRKETG
-ELPMLVLLTHVDGTYGNLVDAEKVGKICQEYNVPFLLNTAYSSGRMPINGKKLLADFVT
CSGHKSWAAGAGVVGILAIRDEWKDKVFKPS--------QRYKVKPIEILGCS-ARGSST
LVLMASFPYVMER--VKHWDVEVEKARWFSNQMESLGDIRQLGDKPHNHDLIRFETPV-F
DRIAQKHKRRGYFLYEELMKRKIVGIKAGKTKSFDLSTYGLNKEQLSYVIDSFKDIINKL
S------------------

#CG (SCA130) archaeon
------------------------------------------------------------
---------------MTELRVVEEDFINLNPLQTGGRTT--PDVRKAVNSFI-DGYSVCD
WCL---GDVNTIKNPDIQGLRDDIG-KFLGADEVILTNGCREAKYAVMQAVCKKGG----
-----KVLADVNRHYSTDVAAELAGLKMEFV-QNSGSPEFKIRAEDY-AKRIDEVS----
---PDLVLLTHVDGNYGNVADAAKVGAICRKKGVPFILNAAYSAGRMLVDNKKIRADFIA
CSGHKSWAVGGGNIGLLGVNNDFKDKVLKRS--------SEYKNKILGILGCS-ARGSSA
VALLNSFPSVKKR--VKDWGKEVKNAQWFANKMLSINGVELIGENPHQHDLMQFSTPV-F
YEISQKHKKKGYFLYKFLEEKGVRGLKAGRTKNFKVSTYGLGMEKLKYVFEVFREAAKL-
-------------------

#Hadesarchaea archaeon DG-33
------------------------------------------------------------
--MPRIPKERLDRYLGLKREFKEERYINLNPIQRGGVLT--SEARKALLEFG-DGYSTCD
WCPPKAARLDAIERPPIAQFM-RDLAEFLNMDVARVVTRCREAKFITFTTLGEPGD----
-----YVVVDSLAHYSTYIAAELARLKIKEV-PHGGPPEFKLDLDAY-ADRIEEVKRETG
-KLPAVVLLTHVDYLYGNLNDAAAVGKICKKYGVPFLLNAAYTAGVMPIDGKKLGADIIV
SSGHKSWAASA-PTGILALKEELVDKMLARSKVVGDWSERKFGLKEYALLGCT-VMGAPL
ISLMASFPHVVER--VERWPQEVEQAGYLVEQLERIEGTRQLGVKPKQHTLIHMESDG-F
YKASQTHKRRGFFLYDELKRRGIVGLQPGLTKHFKLNPYGLSREKVEQVTKSFLEIANEQ
KLNVN--------------

#Hadesarchaea archaeon YNP_45
------------------------------------------------------------
--MPKISKERLEIYRELRRDFKEDEFINLHPIQRGGVLS--PEARKAIVEFA-DGYSTCD
WCPPKTARLDMIERPPIAQFM-QDLAEFLGMDVARVVTRCREAQFISFTMLGNPGD----
-----YVVLDSLAHYSTYTAAELARLRVKEV-PHSGYPEFRLNLEAY-AEKIEEVKRETG
-KPPAAVLLTHVDYLYGNLNDAAVVGKICKEYGVPFILNAAYTGGIMDIDGKKLGADIIT
SSGHKSWAASA-PTGILAATSEMGEKLFARSKIVGDWSKRKFGVKEYALLGCT-VMGAPL
MTLIASFPHVVER--VERWPQEVEKARYLVEQLERIEGTRQIGVRPKNHTLIHMESDG-F
FRASQAHKRRGYFLYEELRRRGIVGVQPGLTKHFKLNTYGLSQEKVERVAQAFLEIAREQ
NLSVS--------------

#Sulfidic spring Ya3
------------------------------------------------------------
---MKIPKERLEKYRNLKREFSEEEYINIHPIQRGGVLT--EEAMKALIEFG-DGYSVCD
FCPPKEARLDMITRPPIRDFY-KDLAKFLGMDYVRVVTRCREAIFIAFKMLSEPND----
-----YVIIDSNAHYSTYLAAELSGLRIKEV-PNSGYPEFKVNLEAY-IDKIEEVKKEKG
-KLPSAILLTHVDYLYGNLNDAKIVGKIAKDYNIPFILNCAYSAGVMPINGKELGADIIT
GSGHKSWAASA-PTGIIAINESLKEKILARSKISGDITKREFKAKEVALLGCT-VMGAPL
ITLMASFPKIVER--VEKWEEEINKIRYLVSELERIEGFKQLGIRPKLHTLTHMESES-F
YKVSINHKRKGFFLYEELKERKIVGIQPGLTKHFKFNTYGLTWEQIKYIRDAFFDIARKY
NVNIK--------------

#Hot spring Ja3
------------------------------------------------------------
---MKIPKYRLEKYKNLKRLFSEDEYLNIHPIQRGGVLT--EEAMKAIIEFG-DGYSVCD
LCPPKSARLDMITSPPIKDFY-EDLAEFLGMDYARVVTRCREAIFIAFRMLGERGD----
-----YVIIDSNAHYSTYLAAEFAELKIKEV-PNSGYPEFKINLESY-EEKINEVKKETG
-KFPIAIFLTHVDYLYGNLNDAKIVGKIAKDYGIPFILNCAYSAGIMPINGKELYADVIT
SSGHKSWAASA-PIGIIAFNKSLYDKITAKPKIKGDLTNRSFEIKELALLGCT-VMGAPL
MTLMASFPHIVER--VEKWDEEVNKARYIVNELEKIEGFKQLGVKPKMHTLIHMESES-F
YKVSLNHKRKGYFLYEELKERKIVGIQPGLTKHFKFKIYGLSWNQIKYLRDAFFEIAEKY
GIK----------------

#archaeon V1
------------------------------------------------------------
---MKISSERLKKYEGMKRDFSEEEYINLQPIQRGGVLT--EEARRALIEFG-DGYSVCD
WCPPKTARLDMIDDPPIHQFY-ADLAEFLGMDLARIVTRCREAKFIAFKMLGKAGD----
-----YAIIDSTAHYSSYISAELADLRVKEV-PNTGAPEYRIDPELY-ASKIEEVKKETG
-RLPAALLLTHVDYLYGNLNDAKAVGKIAEEYKVPFILNCAYSAGIMPIDGKEVGADVIT
SSGHKSWAACA-PTGILALRNSLGEKVLAKPKIEGDLTKRKFVSKELGLLGCT-VAGAPL
VTLMASFPEVVRR--VERWGEELEKIRFLVKEMERIEGVRQIGMRPKMHTLTHLETDP-F
FKASETHKRKGYFLYDELRARKIVGIQPGLTKHFKLNTYGLTWDQVKYVKDAFLEIASKY
GMKVN--------------

#Sulfidic spring Ya2
------------------------------------------------------------
---MKIPKERLEKYKGLRRDFAEEEYINLHPIQRGGILT--EEAMRAILEFG-DGYSTCD
WCPPKTARLDMITNPPIQDFY-KDLAEFLGMDVARVVTRCREAKFIAFKMLGRPGD----
-----YVVLDSTAHYSSYIAAELADMRVKEV-PNSGPPEFRVDLNEY-ATKIEEVKEECG
-RLPAVILLTHVDYLYGNLNDAKIVGKIARDYGVPFILNCAYTAGVMPVDGKELGADIIT
SSGHKSWAASA-PTGILAFNRSLEGKLATRSKIEGDLTKRKFGAKELAMLGCT-VMGAPL
ITLMASFPEVVSR--VERWGEEIGKVRYLVSELERIEGVRQLGIRPKLHTLTHMETEG-F
YKISLAHKRKGYFLYDELRERKIVGIQPGLTKHFKFNTYGLTWDQIRYVRDAFFDIAKKY
GLSVS--------------

#Hot spring Ja1
------------------------------------------------------------
-----MSDTNMDKYKYLRRELNKSELINLNPIQRGGILT--KEAREALLEFG-DGYSTCD
WCPPKFARLDRIEKPPIKDFY-EDLAEFLGMDVARVVTRCREAMFIVFKMLSLPGD----
-----YIVVDSLAHYSTYLAIEFAGLKVKEV-PHTSYPNFQLLLEMY-RDKIEEVKKETG
-KLPAAVLLTHVDHLYGNFNDTLIVSKIAHEYNIPVILNAAYTAGIMSVNGKKLGVDVIV
SSGHKSWAASA-PTGILALTEDMAKKILPTATITGDWSKRSFKTKEMPLLGCT-VMGAPL
ITLMASFPHVVKR--VEKWNEELEKVQYFVKEIEKIEETRVLGVKPKKHTLIHVESIG-L
HKVSEKHHRRGFYLYDELKKRKIVGIQPGLSKHFKLNIYGLSWDEVKYVVNAFHDIAATY
GLKI---------------

#Hot spring Ja2
------------------------------------------------------------
-----MSDTNMDKYKYLRRELNKSELINLNPIQRGGILT--KEAREALLEFG-DGYSTCD
WCPPKFARLDRIEKPPIKDFY-EDLAEFLGMDVARVVTRCREAMFIVFKMLSLPGD----
-----YIVVDSLAHYSTYLAIEFAGLKVKEV-PHTSYPNFQLLLEMY-RDKIEEVKKETG
-KLPAAVLLTHVDHLYGNFNDTLIVSKIAHEYNIPVILNAAYTAGIMSVNGKKLGVDVIV
SSGHKSWAASA-PTGILALTEDMAKKILPTATITGDWSKRSFKTKEMPLLGCT-VMGAPL
ITLMASFPHVVKR--VEKWNEELEKVQYFVKEIEKIEETRVLGVKPKKHTLIHVESIG-L
HKVSEKHHRRGFYLYDELKKRKIVGIQPGLSKHFKLNIYGLSWDEVKYVVNAFHDIAATY
GLKI---------------

#pSL50 archaeon JGI MDM2 LHC4sed-1-M8 1st
------------------------------------------------------------
---------MLDKYKCLRREFNKPGLINLNPIQRGGILS--KEAREALLEFG-DGYSTCD
WCPPKFARLDKIEKPPIREFY-EDLAEFLGMDTARVVTRCREAMFIAFKMLSSPGD----
-----YVVVDSLAHYSTYLAAEFAGLRVKEV-PHSGYPSFEVLMEMY-RDKIEEVKKETG
-KLPAAVLITHVDYLYGNFNDATVVSKIAHEYGVPVILNAAYTAGVMPVNGGKLGVDVIV
SSGHKSWAASA-PTGIIALTEDMAGRILKTSSITGDWSRRSFKVKELPLLGCT-VMGAPL
ITLIASFPHVVER--VKRWSEELEKVQYFVKEIERIEGTRILGVKPKKHTLIHVESIG-L
HKVSEKHHRRGFYLYDELKKRGIVGIQPGLSKHFKLNIYGLSWDEVKHVVNAFHEIASTY
GLKIQ--------------

#Sulfidic spring Ya1
------------------------------------------------------------
---MTLTKDRLNKYLNIRRELSESKYINVNPIQRGGVLS--KEARMALIEWG-DGYSVCD
FCPPQTPRLDKITNPPISDFY-KDLAEFLGMDEARVVTRCREAKLIAFMMLARPGD----
-----YIVLDSLAHYTSYLAAEVVGLRIKEV-PNSGYPEFRVSPEGY-AAKIEEVVKETG
-RPPALALVTHVDYLYGNMVNAAKIAEVCREYGVPVVLNAAYTAGVSPVDGRKLGVDVIV
SSGHKSWAACA-PTGILAMTEEAAKKILRTSSVEGEWSKRRFAMKELALLGCT-VMGAPL
ITLIASFPHVVER--VGRWEEEVEKARFVVRELERIDGFKHLGTRPKMHTLIHMESQS-F
YEVSKKHKRRGYFLYDELKARGIVGIQPGLTKHFKFNVYGLTWYQVKHFVESFHEIARKY
GVEVR--------------

#3300009598.a:Ga0105154_10051793
------------------------------------------------------------
---MSLTRDRLSKYLNIRREFSESKYINVNPIQRGGVLS--KEARMALIEWG-DGYSVCD
FCPPRTSRLDKITNPPISDFY-EDLADFLGMDEARVVTRCREAKLIAFMMLARPGD----
-----YIVLDSLAHYTSYLAAEVAGLKIKEV-PNSGYPEFRVSPEGY-AAKIEEVVKETG
-KPPALALVTHADYLYGNVVDAAKIANVCREYGVPVVLNAAYTAGVSPVNGKKLGVDVIV
SSGHKSWAACA-PTGILAMTEEAAKKILRTSSVEGDWSKRRFAMKELALLGCT-VMGAPL
ITLMASFPHVVER--VNRWEEEVEKARFVVRELERIDGFEQLGIRPKMHTLIHMESQS-F
HEVSKRHKRRGYFLYDELKARGIVGIQPGLTKHFKFNVYGLTWGQVKYFVESFHEIAKKY
GVEVR--------------

#3300009598.a:Ga0105154_10004335
------------------------------------------------------------
---MTLTRDKLNKYLNIRREFSESRYINVNPIQRGGVLS--KEARMALIEWG-DGYSVCD
FCPPQAPRLDKITNPPISDFY-KDLAEFLGMDEARVVTRCREAKLITFMMLARPGD----
-----YIVLDSLAHYTSYLAAEVAGLRIKEV-PNSGYPEFRVSPEGY-ATKIEEVVRETG
-RPPALALVTHVDYLYGNMVDAAKIAEVCHGYGVPVVLNAAYTAGVSPVDGKKLGADVIV
SSGHKSWAACA-PTGILAMTEEMAKRVLRTSGVEGEWSKRRFAMKELALLGCT-VMGAPL
VTLMASFPHVVER--VSRWEEEVEKARYVVRELERIDGFKQLGTRPKMHTLIHVESQS-F
YDVSKKHKRRGYFLYDELKAKGIVGIQPGLTKHFKFNVYGLTWDQVKHFVESFHEIARKY
GVEVR--------------

#SSWTFF a1
------------------------------------------------------------
---MTLTRDRLNKYLNIRREFSESKYINVNPIQRGGVLS--KEARMALMEWG-DGYSVCD
FCPPQAPRLDKITNPPISDFY-RDLAEFLGMDEARVVTRCREAKLITFMMLAKPGD----
-----YVVLDSLAHYTSYLAAEVVGLKIKEV-PNSGYPEFKVSPEGY-ATKIEEVVKETG
-RPPALALVTHVDYLYGNMVDAAKIAEVCREYGVPVVLNAAYTAGVSPVSGKELGVDVIV
SSGHKSWAACA-PTGILAMTEEAAKKILRTSSAEGEWSKRRFAMKELALLGCT-VMGAPL
ITLMASFPHVVER--VSRWEEEVEKARFVVRELERIEGFKQLGTRPKMHTLIHVESQS-F
YEVSKKHKRRGYFLYDELKSKGIVGIQPGLTKHFKFNVYGLTWSQVKYFVESFHEIARKY
GVEVR--------------

#3300009598.a:Ga0105154_10031313
------------------------------------------------------------
--------------LDIRRELSESKYINVNPIQRGGVLS--KEARMALMEWG-DGYSVCD
FCPPQAPRLDKITNPPIGDFY-EDLAEFLGMDEARVVTRCREAKLITFMKLAKPGD----
-----YIVLDSLAHYTSYLAAEVVGLRIKEV-PNSGYPEFRVSPEGY-ASKIEEVIKETG
-RPPALALVTHVDYLYGNMVDAAKVAEVCREYGVPVVLNAAYTAGVSPVNGRKLGVDVIV
SSGHKSWAACA-PTGILAMTEETAKRILRTSSVEGEWSKRRFAMKELALLGCT-VMGAPL
ITLMASFPHVVER--VGRWEEEVEKARFVVGELERIDGFKQLGTRPKAHTLIHMESQS-F
YEVSKKHKRKGYFLYDELKARGIVGIQPGLTKHFKFNVYGLTWSQVKYFVESFHEIARKY
GVEVR--------------

#3300009598.a:Ga0105154_10038695
------------------------------------------------------------
---MSLTRDRLSKYLNIRREFSESMYINVNPIQRGGVLS--KEARMALIEWG-DGYSVCD
FCPPQAPRLDKITKPPIGDFY-KDLAEFLGMDEARVVTRCREAKLIAFMMLAKPGD----
-----YIVLDSLAHYTSYLAAEVVGLRIKEV-PNSGYPEFSVSPEGY-AAKIEEVVKETG
-RPPALALVTHVDYLYGNMVDAAKIAEVCREYGVPVVLNAAYTAGVSPVNGRKLDVDVIV
SSGHKSWAACA-PTGILAMTEEAAKRILRTSSVEGEWSKRRFAMKELALLGCT-VMGAPL
ITLMASFPHVVER--VSRWEEEVQKARFVVRELERIDGFKQLGTRPKVHTLIHMESQS-F
YEVSKKHKRKGYFLYDELKARGIVGIQPGLTKHFKFNVYGLTWGQVKYFVESFHEIARKY
GVEVR--------------

#pJP 33 archaeon JGI MDM2 SSWTFF-2-K12
------------------------------------------------------------
---MSLTRDRLSKYLNIRREFSESKYINVNPIQRGGVLS--KEARMALIEWG-DGYSVCD
FCPPRTSRLDKITNPPISDFY-EDLADFLGMDEARVVTRCREAKLIAFMMLARPGD----
-----YIVLDSLAHYTSYLAAEVAGLKIKEV-PNSGYPEFRVSPEGY-AAKIEEVVKETG
-KPPALALVTHADYLYGNVVDAAKIANVCREYGVPVVLNAAYTAGVSPVNGRKLGVDVIV
SSGHKSWAACA-PTGILAMTEEAAKKVLKTSSIEGDWSKRRFAMKELALLGCT-VMGAPL
ITLMASFPHVVER--VNRWEEEVEKARFVVRELERIDGFKQLGIRPKMHTLIHMESQS-F
YEVSKRHKRKGYFLYDELKAGGIVGIQPGLTKHFKFNVYGLTWGQVKYFVESFHEIAKKY
GVEVR--------------

#Jinze (W8A-19) archaeon
------------------------------------------------------------
---MPLRPERLSKYRNLTR-STEIEAINLNPIQSGGRAGNYPEVVKSVLNFI-DGYSTCD
YCIK--GRLEEIETPPLCDFH-ADLAEFIEMDEVRLLPGCRQAQFAAFHSLASPGD----
-----YVVIDSLAHYSTYLAAERAGLKIIEV-PNSGFPYFEVSPELY-AEKIEEAKRVTK
-KTPALVFLTHVDYSYGNLADAEAIAKVSHEYDVPFMLNCAYTIGRMPVSGRKLKADVVV
ASLHKSFASPA-PSGILAANGEYAEKIFRKSTVKGEWSGRTFPNKEVELLGCT-LPGCVV
VGVMAAFPYVAER--VERWSEEVEKARYFSSQLTKIEGVIQLGQTPHNHDLLHFESSS-F
FEVARAHPRKGFFLYDELKARGVVGVQPGLSRSWKLSTYGQSWENVKKAAQAFLEIAEKY
NIPVS--------------

#LHC4sed (W8A-19) archaeon
------------------------------------------------------------
---MPLEPERLLKYRNLTR-STEVEMINLHPIQSGGRAGNYPEVVKAVLNFI-DGYSTCD
YCIK--GRLDEIETPPLCDFH-ADLAEFIEMDEVRLLPGCRQAQFAAFHSLASPGD----
-----YVVIDSLAHYSTYLAAERAGLKIVEV-PNTGFPYFEMPPELY-AEKIEEVKRATK
-KPPALVFLTHVDYSYGNLADAEVIAKISHEYDVPFMLNCAYTLGRMPVSGRKLGADVMV
ASLHKSFASPA-PSGVLAANEEYADKIFRKSMVKGDWSGRAFPNKEVELLGCT-LPGCVA
VGVMAAFPYVVER--VERWNEEVEKARYFSSQLTRIEGVIQLGQSPHNHDLLHFESSS-F
FEVARAHSRKGFFLYDELKSRGITGVQPGLSRSWKLSTYGQCWENVRKAAQAFLEIAEKY
NIPIS--------------

#GB (W8A-19) archaeon No. 7
------------------------------------------------------------
--MLPFSPYATSRYLNLTR-TTEMNMINLNPIQSGGRAGDYPGVVKAVLEFI-DGYSTCD
YCIK--GRLDEIETPPLSDFH-ADLASFIDMDEVRLLPGCRQAQFAAFHSLAEPGD----
-----YVVVDSLSHYSTYLAAERARLNIVEV-PHSGHPHFEINPELY-AEKIEEVKRSTG
-KLPAVVFLTHVDYSYGNLNDAKEVGKIAREYGVPFMLNCAYTIGRMPVSGKSLHADIVT
ASLHKSFASPA-PSGILAANGEYAERIFVKS-----------------------------
------------------------------------------------------------
------------------------------------------------------------
-------------------

#CP (AK8) archaea
------------------------------------------------------------
----MFDEKVLEKYKNLVR-GVDENMINLHPIQSGGRAGDYPGVVQAVLRFV-DGYSVCD
FCMK--GRLDLIESPPLCDFH-RDIAAFLDMDVARLFPGCRQAQYAAFHALVKPED----
-----TIIIDSLAHYSTYLSAERVGARIVEV-PHSGYPDFDLLLDSY-ADKIEEVKRDSG
-KWPALAFLTHVDYNYGNVFDAKSVGKILHDYGIPFVLNGAYTVGRMPISGKDLGADILT
ASLHKSFASPA-PSGLLLANVDYADVIFKNSSIRGPWSGRSFENKEVETLGCT-LPGCVA
IGVMAAFPYVAER--VKHWDEEVKKARYFIEQLERIAGIKQLGQRPHNHDLVHFESES-F
FEIAQKHPRKGFFLYEELRERGVTGIQPGLSKFFKVSTYGKTWNQIKLAAEAFLDIAQKY
NIPIH--------------

#GB (AK8) archaeon No. 8
------------------------------------------------------------
----MFDEGVLERYRNLTR-DVEERMINLHPI------------------------SVCD
FCLK--GRLDLIESPPLCDFH-RDIAAFLEMDEARLFPGCRQAQYAAFHALVKPDD----
-----TIIVDSLAHYSTYLSAERVGARIVEV-PHSGYPEYDLFIDAY-AERIEEVKKETG
-KWPALAFLTHVDYNYGNVFDAKSVGEVIHDYGVPFVLNGAYTIGRMPVSGRELGADVMT
ASLHKSFASPA-PSGLLLANEGYSDVIFRRSSIRGPWSGRNFENKEIETLGCT-LPGCVA
IGVMAAFPYVVER--VKHWDEEVKKARYFIEQLERIDGIKQLGQRPHRHDLIHFESKS-F
FEVAQKHPRKGFFLYDELKARGVIGVQPGLSKSFKVSTYGKTWEQIKIAAEAFIEVAQKY
NIPTH--------------

#Methanocorpusculum labreanum 1st
------------------------------------------------------------
----------MKCCDNIDARMVDETSINLDPIQVGGRLT--PEAMKAMISWG-DGYSVCD
NCRK-PFRLDYIEKPPLKDFH-VDVAEWLGMAQARTVPGARRGFQQVAGTYVEKGD----
-----PVLIGALAHYTSYLSVELQKGIVREI-PKT--ADNHITAEDT-ANRIEDVVREFG
-IAPKLLYIDHVDYQFGNMHDVKGIAKVAHQYDIPVLYNGVYTVGIMPVNGKDLGVDFII
GSGHKSMAAPA-PSGILAATEERANEVFRTTQMEGDLTGRKFGIKEVGILGCS-LMGAPI
VGLLASFPTVKAR--VEHFDEELANSKIVVEALRFIEGTKILSEYPRKHTLTRVDTTGSF
DQVAQTHKKRGFFLSSALGKKGITGIIPGATKVWKFNTYGMTKKQAEYVADTYLEIAETN
GLTIN--------------

#Methanocorpusculum labreanum 2nd
------------------------------------------------------------
----------MKCAGQIEARAVEESSINLDPIQAAGRLT--PEAMKAVIAWG-DGYSVCD
NCHK-PFRLDYVTKPPIADFH-EEAAKWLGMDKIQLVPGARRAFQEVTGALVGKGE----
-----PVIMTGMGHYTAYLSVEVVNGVVREI-RPT--ADHHITADAA-AESIENAVREFG
-YAPKLVFVDAVDFMYGNMHEVEKIAKVAHQYDIPVLYNGVYTVGVLPVDGKKLGVDFVV
GSGHKSMAAPA-PSGILATTDEYAEIVLRTTKIQGDITGRKFGIKQVGILGCS-LMGDPA
VGLIASFPRVKER--VNHFDEELKNHKIVTDALLSIEGTKVASEYPRKHTLTRMDTAGSF
DVIARTHKKRGFFLSSALNKRGITGIMPGVTKQWKFNTYGLTKTQAEYLADSFIEIAEEN
SMVVG--------------

#Methanoculleus marisnigri
------------------------------------------------------------
----------MKCAVDIESRDVEEMYINIDPIQAGGRLT--VDAMKAAISFA-DGYSVCD
NCRS-PFRLDYIQKPPIAQFH-ADLAAWLNMDTARVVPGARRGFQAVASTYVEKGD----
-----PVIVTSLAHYTEFVAVEEAGGIPLEV-PKD--EKNHITPDAA-AEKIEAVILEFS
-KTPPLLFIDHVDYQFGNVHDVAAITKVAHQYDIPVLVNGAYSVGIMPVDGKALGADFVV
GSGHKSMAAPA-PSGVLATTNEHAERVFRTTQAKGDVTGRTFGIKEVEMMGCT-LMGVTV
VGMMASFPHVKER--VKHWDTEVAHSQAVVDALLSIEGTKVLSDYPRQHTLTRIDTRESF
DKVAQQHKKRGFFLSSDLKKRGITGVIPGSTRVWKFNTFGLTEKQIRHVGESFVEIAREN
GLNIV--------------

#Methanoregula formicica 2nd
------------------------------------------------------------
----------MRCGQGIEARQVDELFINIDPIQAGGRLT--ADAMKAVVAYG-DGYSVCD
NCRK-PNRLDYIAKPPIAEFH-KDVAKWLNMDAVRTVPGARRGFQAVAHTYVNKGD----
-----PVLLTSLSHYTEFLAVEESGGVAREI-PAN--DQKLVTPDAT-AEKIEAVKREFG
-RAPVLAFIDHVDYQYGNMHDVAGIAKVCRQYDVPIVYNGAYTVGILPVDGKAMGVDFVV
GSGHKSMAAPA-PSGVLAATAEREKEVFRTTAIIGDVTNRKFGIKEPEMMGCT-LMGVTL
VGMMASFPHVKER--TKHFDKELANHKIVMDALLSIEGTKCLSEFPRKHTMTRIDTIASF
DKVAESHKKRGYYLQSALEEKGITGVIPGATKVWKFNTYGTTRKQAEHLGRAFVEIAREN
GLAVKG-------------

#Hadesarchaea archaeon DG-33-1
------------------------------------------------------------
---------MRNIVEDIEIRAREELFINIHPIQSGGRLT--PEAMKAVIAYG-DGYSTCD
WCRK-PFRLDKIKRPPIEEFH-VELAEFLGMDQARVVPGARRGFQAVVSSLVEKGD----
-----SVIVSSLAHYSEFLAVEDAGGIVKEV-PLN--EKNIVTAEAT-ARKIEEVKKETG
-KLPKLIMIDHFDYMLGNEHDIRGIAKVAKEHDVPFLCNGAYAIGVLPVNGKEIGADFLV
GSGHKSFASPA-PSGIVATTNEWVEKVFRTTQMVGDVTKRKFGIKEVEMLGCT-LMGANL
IAMMASFPAVKER--VKHWDDEVKKINYLMDEFLKIEGNKVPSEMPRKHTLTKVDTTGSF
DKIAQTHKRRGFFFSDELSDRKIKGEFAGATRAWKLNSYGLTWDQIRYLAQAFQDIAKKY
GIKVLQ-------------

#Ca. Bathyarchaeota archaeon BA2
------------------------------------------------------------
---------MKQMIEEIEVRAREELFINIQPIQAAGRLT--PEAMKAIIAYG-DGYSTCD
WCMA-PFRLDKIRMPPINEFH-VKLAKFVGMDQARVVPGARRGFQAVVSSLVEKGD----
-----SVIVSALAHYTEFLAVENVGGIVKEV-PLN--EENIVTGENT-AQKIEKVKKETG
-KLPKLIMMDHFDYMFGNEHDIHGIAKVAKEYDVPFLANCAYSLGVMPVNGKEMGADFLV
GSGHKSFASPA-PSGILATTNEWAEKVFRTTQMVGDLTGRKFGIKEVEMMGCT-LMGANL
IAMMASFPAVHER--VKHWDDEIKKANYFIDEFLKIEGNKVLSEMPRKHTLTKVDTTGSF
DKIARTHKRRGFFFSDELKKRRIIGEFAGATRQWKLNTYGLTWDQIKYLAEAFQDIAKKY
GMNVA--------------

#pSL50 archaeon JGI MDM2 LHC4sed-1-M8 2nd
-MSKKAVEALFDAMFALTDLRQIFRETKPVFNFSLEQRKVIKSILGGVREFLDVIEREID
NPTLKYSFPRKYIADGVEVRAREELYVNIHPIQPGGRLT--SEAMKALIAYG-DGYSTCD
WCRK-PFRLDKIHRPPIDEFH-IELAKFINMDEARVVPGARRGFQAVISSLIDKGD----
-----YVVVSSLAHYTEFLAVEDAGGVVAEV-PLN--ENNIVSGEAV-AGKIEEVKRRTG
-KYPKLIMIDHFDYMYGNEHDIYGVSKVAKEYNIPFLCNCAYSLGVMPIDGKKIGADFIV
GSGHKSFASPA-PSGIVAATGEWAPVIFRTTQMVGDVTKRKFGIKEVEMLGCT-LMGGTL
LAMMASFPRVKER--VKKWGEEVEKSNYFAKEFLRIKGSKILSEMPRKHTLTKVDTTESF
DKIARKHKRRGFFLSDELEERGIIGEFAGATRSWKLNTYGLTWDQVKYLIEVLHEIAIKH
GLEVT--------------

#pSL50 archaeon JGI MDM2 LHC4sed-1-N8
---------MMMWKSALTDLRQIFRETKPVFNFSLEQRKVIKSILGGVREFLDVIEREID
NPTLKYSFPRKYIADGVEVRAREELYVNIHPIQPGGRLT--SEAMKALIAYG-DGYSTCD
WCRKPF-RLDKIHRPPIDEFH-IELAKFINMDEARVVPGARRGFQAVISSLIDKGD----
-----YVVVSSLAHYTEFLAVEDAGGVVAEV-PLN--ENNIVSGEAV-AGKIEEVKRRTG
-KYPKLIMIDHFDYMYGNEHDIYGVSKVAKEYNIPFLCNCAYSLGVMPIDGKKIGADFIV
GSGHKSFASPA-PSGIVATTGEWAPVIFRTTKMVGDVTKRRFGIKEVEMLGCT-LMGGTL
LAMMASFPRVKER--VKKWGEEVEKSNYFAKEFLRIKGSKILSEMPRKHTLTKVDTTESF
DKIARKHKRRGFFLSDELEERGIIGEFAGATRSWKLNTYGLTWDQVKYLIEVLHEIAIKH
GLEVT--------------

#MSBL1 archaeon SCGC-AAA259E19
------------------------------------------------------------
----------MNIGDDIETRAREELFINLNPIQAAGRLT--PEAMKAMISYG-DGYSTCD
WCMK-PWRLDKIERPPIQEFH-SNLAEFLGMDEARVVPGARRGFQAVVSTLVEKDD----
-----TVVVSALAHYTEFLSVEQAGGKIKEI-PLN--ENNIATGEAA-AQKIEKVKEETG
-ELPKLIILDHFDYMFGNEQDVEGIAKVSDDYDIPFLLNGAYSVGVMPVDGKELGADFLV
GSGHKSFASPA-PSGILATTDEWTDEVFRTTEMVGDVSGRKFGIKEVEMLGCT-LMGSNL
IAMMATFPEIKRR--VKDWDEEVEKANYFLDQFLKIKGNRVLSEMPRKHTLVKVDTRESF
DKIAKNHDRRGYFLYDELKERGIIGVFAGATRTWKINLYGLTWDQIEHLAKSYKEIARKY
ELPVEE-------------

#MSBL1 archaeon SCGC-AAA261F19 2nd
------------------------------------------------------------
-----------MIGEEIETRAREELFINVNPIQAGGRLT--TEAMKALISHG-DGYSTCD
WCKK-PFRLDKIKKPPIDEFH-SDLADFLGMEEARVVPGARRGFQAVVSTLVDKGD----
-----FVIVSALSHYTEFLSVEQAGGTIREV-PLN--KENIHTAKAT-ANKIEEIKKETG
-DVPKLIIADHFDYMLGNEHEVEEIAKVAHEYDVPFLYNGAYSIGVKPVNGKKIGADFMV
GSGHKSFASPA-PSGILATTDKWADEVFRTTEMEGDVTGRNFGLKEVELLGCT-LMGSNL
IAMMASFSRIKER--VKNWDEEIKKVNYLMDEFQKIEGNEILSEMPRKHTLVKVDTRKSF
DKIAKDHSRRGYFFYDELKERGIIGIFPGATREWKMNTYGLTWKQIRYLAKSFKEIARNY
GLNVQE-------------

#Methanoflorens stordalenmirensis 1st
------------------------------------------------------------
----------MKIN-DIPVREVEENYVNIHPIQAAGRLT--AEAMKALIAYG-DGYSTCD
QCRK-PFRLDKITKPPIAEFH-SDLAKFLNMDEARVTPGARRAFQAVCLSLAEKGD----
-----IVLVSALAHYTEFLAVENARAAVKEV-PLN--KDNVVTGDAV-AQKIEEVTREEG
-KMPALVMLDHFDYSFANEHDIAGAAKAAHQYDVPILYNGAYTVGIKPVDGKALGVDFVV
GSGHKSMASPA-PSGVLATTSEYADQVLRTTQMIGDVTGRKFGIKEVEMLGCT-LMGANL
LAMIASFPAIQRR--IKDWQEYVAKSNIVIDAILDIKGNKCLSEHPRKHTLTKVDTTGSF
DKIATTHKRRGFFFSDELKQRGVVGEFAGATRTWKLNTYGLTWDQTSYVADVFKEIAGKY
DLL----------------

#Methanoflorens stordalenmirensis 2nd
------------------------------------------------------------
----------MKIAGNIPVREVEENFINIQPIQAAGRLT--AEAMKALIAYG-DGYSTCD
QCRK-PFRLDKITKPPIAEFH-SDLSRFLNMDEARVTPGARRAFQAVCLALVEKGD----
-----TVLVSALGHYTEFLAVENAKAIVREV-PTN--DLNIVTGDAV-AQKIEEVTREQG
-KSPALVMLDHFDYSLANEHDVAGAAKAAHQYDIPILYNGAYTVGIKPVDGKALGVDFVV
GSGHKSMASPA-PSGVLATTSGYADTVFSTTQMIGDVTGRQFGVKEVQMLGCT-LMGGNI
LAMMASFPTVRRR--VEGWGEYVTKSNYVVDALLSIKGNMCVSEYPRKHTLSKVDTTGSF
DTVARIHKRRGFFFSDELKQRGIVGAFSGATRAWKLNTYGLSWDQIRYVADAFKEIAGKY
GLL----------------

#Methanosarcina mazei 2nd
--MDIRTQKTFEALFALEDIRETLRQTLP-NITTSEEEHSIKEGVNKVRKILDDIENSTG
TP------EIRKIADNIEIRSREETYINIQPIQAAGRLT--PEARKALIAYG-DGYSTCD
ACRK-PFRLDKITSPPIAPFH-EQLASFVNMDVARVVPGARRGFQAVASTLVNKGD----
-----SVLVSALAHYTEFLAVEEAGGIVREV-PLN--EKKIVTADAT-AEKIEAVKQETG
-KLPVLVMIDHFDYQYANEHDVKGIAKVAHQYDIPLLYNGAYTVGVRPVDGKDIGADFVV
GSGHKSMASPA-PSGVLATTEEWAPKVFRTTQMTGDLTKRKFGVKEVEMLGCT-LMGSTL
LGMMASFPAVRER--VKNWEDELKKSNYLIDGLLAIEGSKVVSEYPRKHTLSKVDTTGNF
DVIAQNHKRRGFFLSDELSGKGIVGEFAGATRVWKLNTYGLSWEKVRYVVDTFQDIANKY
ELQVKK-------------

#Methanosaeta thermophila 2nd
--MDIRTQKTFEALFALEDIREILRQRLP-DTITPEEEHSIRKGVDRVRKILDDIENSTG
VP------EIRKIANNIDIRSREENYINIQPIQAAGRLT--PEARKALIAYG-DGYSTCD
ACRK-PFRLDKISSPPIAPFH-EQLASFVNMDVARVVPGARRGFQAVASTLVDKGD----
-----SVIVSALAHYTEFLAVEEAGGIVREV-PLN--EHNIVTADAT-AERIEAVKQETG
-KLPVLVMIDHFDYQYANEHDVKGIAKVAHQYDIPLLYNGAYTVGVMPVDGKDIGADFVV
GSGHKSMASPA-PSGVLATTEEWAPKIFRTTQMTGDLTGRKFGVKEVEMLGCT-LMGSTL
LGMMASFPTVKER--VKNWEDELRKSNYLIDGLLAIKGSKVVSEYPRKHTLSKIDTTESF
DVIAQKHKRRGFFLTDELSSKGIVGEFAGATRVWKLNTYGLSWEKVRYVIDTFQDIADKY
NLQVKREDNSNDNREL---

#Methanoregula formicica 1st
--MSIRIQKTFEALFALEEVRGLFRESLP-TGFDPAEEAAFRQKIAEVKAILADLEAGTG
TP------KVTKIAGSIDLRTREEEAINIQPIQAAGRLT--TEARKALISYG-DGYSTCD
ACRK-PFRLDKISKPGIAEFH-ADLAKFVGMDHARVVPGARRGFQAVTGTLVNKGD----
-----TVIVSALAHYTEFLAVENAGGVVREV-PLN--DKNIVTGEAT-AQKIEEVKAETG
-KFPVLVMIDHFDYQFANEHEIKEIGKVAHQYDIPFLYNGAYTVGVMPVNGKAIGADFVV
GSGHKSMASVA-PSGVLAMTEEWLPKALRTTSMVGDLTKRKFGIKEVEMLGCT-LMGGTL
LSMMASFPTVKAR--TLKWEEEVQRSNYFIGRLLKIAGSRVLSEYPRKHTLTKVDTTGSF
DTVAQTHKRRGFYLSDELSSRGIVGEFAGATRTWKLNTYGLSDKKVRYLADAFCEVAEKH
GLAVEK-------------

#Groundwater_metagenome_a1
------------------------------------------------------------
------------MCDNLETRSRDENYINIQPIQAAGKLT--ADAMKAMISYG-DGYSVCD
FCLK-PFRLDKIKKPGLEGFH-ADLAKFVGMDVARTVPGARRGFQAVVSSLVSKGD----
-----SVVLQAVGHYTEFLAVEQSGGVAYEA-AIN--DRGIVTGDAV-AEAIQRAKKKEG
-KLPKLVIFDHFDYTLANEHDIKGAVKAAKEFDIPVLYNGAYTVGIKPVDGKALGVDFVV
GSGHKSMASPA-PSGVLATTEEGAKKVFRTTQITGDVSGRKFGVKEVEMLGCT-LMGANI
VGMMASLPHLKER--VKHWDAEVKNSNYFVEQFLRVDGNKIVSECPRKHCLTKVDCTESY
DKVAQKHKDHGYFLTHELDSAGITGIFPGATRSFKMSTYGLTTEQVKYLAESFVDIAKKH
GLEVH--------------

#Groundwater_metagenome_a2
------------------------------------------------------------
------------MCDGLETRARDELYINIQPIQAAGHLT--ADAMKAMISYG-DGYSVCD
FCLK-PFRLDKIKKPSLEEFH-VDLAKFVGMDVARTVPGARRGFQAVVSSLVGKGD----
-----SVVLQAVGHYTEFLAIEQVGGIAHEA-AFN--DRGIVTGEAV-AQAIENAKKKDG
-KLPKLVIFDHYDYTLANEHDILGAVKAAKEYDIPVLYNGAYTVGVKPVDGKALGVDFVV
GSGHKSMASPA-PSGVLATTNEGAEKVFRTTKISGDVSGRKFGVKEVEMLGCT-LMGANI
VAMMASLPHLKER--VKCWDAEVKNSNYFVKEFLRVDGNKIVSECPRKHCLTKVDCTESY
DKIAQKHKDHGYFFTHELDSRGITGIFPGSSRSFKMSTYGLTDSQVRYLAESFLDIAKKH
GLEVH--------------

#Z7ME43 archaeon DG-70-1
MDKASQIYALYDAMFALEDMREIYRRSLP-TELTENEKSELKEKIQHTRSLLEKIESGVK
SH------QKKYIVDKLEPRIREEAFINIQPIQAAGRLT--PEARKALIAYA-DGYSVCD
QCLS-PFRLDKIRNPPIDQFH-QELADFLGMDEARVVPGARRGFQVIMNSLVGKGD----
-----TVVLSSLGHYTEFLAVEQAGGTAREV--FS--DNNVVTGEAY-AEKIEKVKKEMK
-TLPKLVMVDHFDYMYGNLHDIKGIAKVAKEYDIPFLLNGAYSVGVLPVNGKDIGADFVV
GSGHKSMASAA-PSGVVATTEEWADTIFRTTKISGDITGRTFGVKEVEMLGCT-LMGATV
ITLMASFPAVKKR--VKHWDEEVEKSNYFSNQFKRVSGSEVLSEEPRKHTLTRVDTTGSF
DKIAKTHKKKGYYFSNELKNRGITGPFAGATRKWKLNTYGLSWEQIKYLSGAFVEIAEKY
ELPVSD-------------

#3300001854.a:JGI24422J19971_1000009527
--MDSRIQKTYEALFALEDLREILRETSPEHKLNKEQEEKFKKILSELKKSLEGIENNKG
DA------KIRYVTDRIELRTKEEEFININPIQAGGRLT--KEARKALIAYG-DGYSICD
YCFK-PFRLDFIKRPPVHDFY-QELAEFVGMDQARVVRGARRGFQIVASSLLNSGD----
-----IALISSLGHYSLALAVESVGAIWKEV-PLN--EKNIITAENT-QKKIESVKKETG
-KFPRLIAISHIDYMFGNEHDVSGIAKIAKEYEIPFLYNGAYTVGIMPVNGKKIGADFIV
GSGHKSMASPA-PTGVLAVTDEFSKKVFKTTESSGDVTGRKFGVKEVYLLGCT-VMGAPL
IAMMASFPRVKER--VKHWDEEIKKSNYFIREFLKIKGNKAVSEMPRKHTLSKVDSTGSF
DKIAKKHKRRGYFLCDELTKRKITGIFPGATRQFKLNTYGLTWDQIKYLGNVFREIAEEY
RI-----------------

#GB (Altiarchaeales) archaeon No. 1
MKVNPRVQKTYEALFLLEDLREILRETSPEHKLNREQEERFKKILSELKKNLEEIENNEG
DA------RIRYITDRIELRSKEEEFININPIQAGGRLT--REGRKALIAYG-DGYSICD
YCFK-PFRLDFIKRPPVHDFY-QELAEFVGMDQARVVRGARRGFQIVASSLLKKGD----
-----IVLISSLGHYSLALAIESVGAIWKEI-PLN--EKNIITAENT-QKKIESVKEETG
-KFPSLIAISHIDYMFGNEHDVSGIAKIAHEYEIPFLYNGAYTVGVMPVNGKKIGADFVI
GSGHKSMASPA-PTGVLAVTDEFSKKVFKTTELSGDVTGREFGIKEVYLLGCT-VMGAPL
IAMMASFPMVKER--VRHWDEELRKSNYFIREFLKIRGNRVVSEMPRKHTLSRVDSTASF
DKIAKKHKRRGYFLYDELAKRKITGIFPGATKQFKLNTYGLTWDQIKYLGNAFREIAEEY
GI-----------------

#Altiarchaeales archaeon SCGC AAA252-I15
------------------------------------------------------------
---------MEPIIPGIQVRSREEDYINIQPIQAAGRLT--LEARKALISYG-DGYSVCD
FCLR-PFRLDKIHKPAINQFT-LDLAEFLNMDEARVVRGARRAFQIVVNSLVEKGE----
-----TVLVSSIGHYSLALAVESRGALWREV-PLN--EENIVTSEDV-ASKIDSIKKESG
-SLPKLVAIHHVDYQFGNLHPVAEIGKVCRDYGIPFLLNGAYTVGTMPVDGKKLNADFVV
GSGHKSMAAPA-PTGVLAATEEYAAKVFRTTKAEGDVSGRKFGIKETELLGCT-VMGAPL
VALMASFPKVRER--VKNWDDEVEKVNYFTQEFLKIPGNKVLSELPRKHTLTKVDTTETF
GKVSETHKKKGYFFTNELSKRGIVGPFPGATHAYKINTYGLSWSQIKHLSESYLDIAREY
DLI----------------

#Ca. Bathyarchaeota archaeon B63
------------------------------------------------------------
------MIGASSLQKYEAIKNRYRDAVIIHPIMTGGLVPEEAQRRLIEEGWIGIGYSACF
DCIE--GRSSLISKPPIRDFL-ADVAGFLGGDVAEHTFGCRAAQFAVMKAISDYIRDGGG
RDYARVVLADSLCHYTTVIAAEAAGLRLEEV-PHGGYPEYRVEAEGF-RRKFEEIREETG
-KLPGLIALTHVEPYYGNLNPAEEVGRIAEEHEIPYMVNAAYTAGVMPVDMREMHADFLT
VSAHKSMASLG-PLGFLVTNYEWAKRVFEASKASTRWSGRTFASKVPNIFGCS-IGGIPL
ISSMYSLRHVVER--VRRWEEELEKIRWFIREMENLEGFMLLGERPHRHHLLHFESPI-F
WEISKRHRRRGFFLAEEMIKRGIVGLHRGLSKHVKLSVYGLEWSSVRRVRDAFFEIAEKY
IKEFKIAWSSPRQDVSHKG

#Ca. Bathyarchaeota archaeon B26-2
------------------------------------------------------------
------MVKLGSLQRYSQIRNQYKGTTIVHPIMTGGLVPEDVQRRLYEEGWTKIGYSVCF
DCLE--GRSSLISKPPITKFL-GDIAEFFGGDRAEHTFGCRAAQFAVMKTISSFLKSEGS
KRYTKVVLADPLCHYTTAIAAEATGLRIVEP-PHSGYPEYKVEAESF-RKKIEEIKRETG
-RLPGLIVVTQVDPYYGNLNPAEEVGEVAEEYDIPYMVNAAYTAGVMPVSMRELHADFLT
VSAHKSMASLG-PLGFLVTNYEWHKRVFQRSKIVTEWSGRAFGKKIPNIFGCS-IGGIPL
ISAMYSFPHVVER--VGRWEEELKKTRWFIREMEKIDDVMLIGERPHRHHLLHFETPI-F
WEISKRHRRRGFFLAEEMIKRGIVGLHRGLSKHIKLSTYGLSWEELEKVRDAFHEVAEKY
AGESVGSI-----------

#Sulfidic spring Ya4
------------------------------------------------------------
----MSYKFDEKITKFRSIDNPYKHDMIIHPIMRGGIIPDEVQKRIIDEGWMKVGYAVCY
DCIE--GRSGLVSKPPIDEFL-STIAKFFDGDVAEHTFGSRAAQFSVMKTIADNIS----
EDFAKVIITDPLCHYTTAIAAEMNGFRLVEA-QHSGYPYYKVRAEDF-VNKIEIIKKETG
-KLPALIVLTHVEPYYGNLNPAKEVGKIAKEFNIPYMVNGAYTAGVLPVSMRELQADFLT
VSAHKSMASIG-PLGFLITNQEWSDKVFKKLTIKPDWTGRTFGKKIVNIFGCS-VGGLPL
ISALYSFPYVVKR--VEKWEEELGKVRWFISEMEKIDGIRLVGEKPHNHHLMHFETPI-F
WEISKRHKRKGFFLAEEMMKRGIVGLHRGLSKHIKISSYGLSWEELKKVRDAFYDIAKKY
VDEFKLDFDLT--------

#Hot spring Ya3
------------------------------------------------------------
----MGYKFDEKIAKFRSINNPYKHDVIIHPIMRGGIIPDEVQKMIINEGWMKVGYAVCY
DCIE--GRSGLVSKPPINEFL-STVAEFFDGDMAEHTFGSRAAQFSVMKTIADNIS----
EDFARIVLTDPLCHYTTAVAAEMNNLRLLEA-QHSNYPDYKVNAEDF-VNKIESIKKETG
-KLPGLIAVTHVEPYYGNLNPVKEIGKIAKDYGIPYMVNAAYTAGIFPVSMKELKADFLT
VSAHKSMASIG-PLGFLVTNYEWSDKAFKKSTIKPDWTGRTFGKKIVNIFGCS-IGGLPL
ISAMYSFPHVAER--VKKWEEELDKARWFISEMEKMDGIKLVGERPHNHHLMHFETPI-F
WEISKHHKRKGFFLAEEMIKRGIVGLHRGLSKHMKISLYGLSWEEVKRVKDAFYDLTQKY
VDEFKLNYELPKA------

#3300002231.a:KVRMV2_1004402032
------------------------------------------------------------
-----------------MKPYRYKDKMIVNPLMRGGLVPEEVQKALYEDGWTEVGYNVCF
DCIE--GRSGLITKPGIKTHL-GDVARFFGGDVAEHTFGCRGAQFSVMNTIRERVR---E
EKTSEFVIADPNSHYSTNIAAEMCSLKTVEP-PHLGFPEYKVSADSF-TEKIDEVRREHG
-E-PALIAVTHADPYYGNIAPVEEIGKIAEDNEIPYMVNAAYTGGVMPLNMKAMKADFLT
LSAHKSMASVG-PLGYLVTNFHWAKKAFITSREKPDWSGRAFGKKIPNIFGCS-VGGLPL
ISSMMTFPMVKER--VRHWDEELKKTTAFIDEMEEIGDTMLLGERPHRHHLLHFETPD-L
WEISK-------------------------------------------------------
-------------------

#3300002231.a:RMV2_1000579014
------------------------------------------------------------
------------------------------------------------------------
------------------------------------------------------------
----------------------------------------EFDPQAF-GDKIDKIKRDEG
-KKPSAVVMTHADPYYGNIEPVEEVGKICEEKEVPFMLNAAYTAGVMPIDMKKIKCDFLT
ASAHKSMASLA-PLGYVVANHTWGKKVFATSSERPDWSGRLFGKKIPNLFGCS-VGGIPL
ISSMMAFDSVVER--VKYWDEELKNIRWFVEEIENFGDIMLIGQNPHNHHLLHLETPK-F
WEISKTHKRKGFFLAEELEKRGIVGVHKGMTKHIKVSVYGLSKEELKTVRDAFEEIANL-
-------------------

#3300001782.a:WOR52_100302482
------------------------------------------------------------
-MDKLVYNNDLVGKYSHFYRSINEEFINIHPIQRGGILT--PEAYKALISFG-DGYSICD
NCLK--GRIDQIENPPVFEFL-KDMARFLAIDNVILTAAARDSKRMVMQALAKKNP----
--NNKVVIVDSLAHYTTYLAIETNNLKVREV-PNSGDPEFGIKSEDY-EKTIKQVIKEQG
-KTPLLVVLTHVDYKYGNLNNPGPIGEICHNYDIPFLLNAAYSAGILPINCKENKIDFIA
CSGHKSMAASG-PIGLLGFEEKYYEDIMVNSQIQGNLTNKSFPNKICTLMGCPPVYGAPL
ITLMASFPAIVKRSQKEYIEEEAKKANYVIQNIKNVKGMTILGKLPKFHPLTNLRTDG-F
AEVAKSHPRKGFFLREEFKERGVIGLLPGISKEMKFNTYGLTWEQVKHFSSAFLDIAKKY
NLT----------------

#3300002053.a:SMTZ23_100002708
------------------------------------------------------------
-MDRLEYNDELLGKYSHFYRSINEEFINIHPIQRGGILT--PEALKALISFG-DGYSICD
NCLK--GRIDQIENPPVLEFL-KDTARFFNIDNIIPTAAARDSKRIIMQALAKKYP----
--NKKTVVIDSLAHYTTYLAIEANNLKVREV-PHSGEPDFIIKSEDY-EKTIKQVKKDEG
-QTPLLVLLTHVDYKYGNLNDPSPIGEICQNHDIPFVLNAAYSGGILPINCKEDKVDFIA
CSGHKSMSASG-PIGLLGFKENYYEDIMVNSKIQGNLTSKSFPNKICTLMGCPPVYGAPL
ITLMASFPTIVKRTQKEYVDEESNKANFVIKNIENVNGITILGKLPKVHPLTNVKTDC-F
AEIAQSHPRKGFFLREEFKERGIIGLLPGISKEMKFNTYGLTWGQVKHFSSAFLDIAKKY
NII----------------

#3300001687.a:WOR8_1001119110
------------------------------------------------------------
-MKGLEFSSALEQKYATLTRRVNEEFINIHPIQRGGLLT--PEAYKALISYG-DGYSLCD
NCLK--GRLDEIDNPPVRDFL-SDMASFLKINHVMPTAAARDSKRIIMEVLAKKYP----
--DRITVVVDSLAHYTTYLAVEQNDLKVREV-PNSGEPEYSIDPQGY-QVVINEVLEEDG
-CPPLLAVLTHVDYKYGNYNDPKPVGEYCRDFQVPFLLNAAYSGGVLPIDCESNNIDFIS
CSGHKSMAASG-PIGLIGFSDQFHDDIMKNSRIQGNISSKSFPNKICTLMGCPPVYGAPL
ITLMASFPAIVRRTQSEYVEEESKKANYVVDKINPIEGIEVQGKLPKIHPLTNVKTPA-F
VDVAEKHPRRGFFLRDEFKEKGVIGIAPGISKELKFNTYGLTWNQVKYFTESFLEIAEKY
HLI----------------

#3300002052.a:SMTZ1_100279371
------------------------------------------------------------
-MVGLEYSDKLYKKYATLSRQVNENFINIHPIQRGGVLT--PEAQKVLLEYG-DGYSLCD
NCLK--GRIDLIKNPPIDEFL-IDFAKFIDMDNAMPTGAARDAKRMAITALKERFP----
--ERKTVIIDSLAHYSTYLAIELNKLKIKEV-PNSGDTYFEINPEDY-RTKIEEVKKEEG
-VTPLLVLLTHVDYQYGNYNDPKPIGDISKEYDVPFLLNAAYSAGILPVFGRELGVDLLS
CSGHKSMAASG-PIGMLGFSDEFVDNILVPSKIHGDLTDRSFSGKFCNFLGCPPVYGAPM
MTLMASFPSVVKRTQKNVVEDEQKKINFVVEEISKIKGIEVLGKLPKIHPLTHIKTES-F
NEVAKSHKRRGFFVRDEFKKRGIL------------------------------------
-------------------

#BL (MCG) SepCysS
------------------------------------------------------------
--------------------------------MTGGILPKEVVEKLSSEKWMKAGYANCF
DCLA--GRSGLISKPPIGDFL-ADIAQFYGGDVAEHTFGSRAAQYTVMKTISEFAGSEGA
DEYAKTALVDPLCHYTTFMAAEANAIELVEA-PHSGYPDYRVKSEDF-AEKIGEIRAKYG
-KLPALIALTHAEPYYGNLNPVKEIGEIAEEYGIPYMVNCAYTAGVMPINMKEFKADFIT
VSAHKSMASLG-PLGFLVTSFEWSKKAFKTSTIRTDWSGRSFGKKIPNIFGCS-IGGIPL
VSAMYSFPHVVER--TKNWEKELEKTRWFIDEMEKMDGMMMIGERPHRHHLTHFETPA-F
WEISEHHRRKGFFLAEEMVSRGIVGLHRGLSKHVKLSVYGLSWDQVINVRNAFQEILDSH
AKTSKSR------------

#BL (MCG) bin
------------------------------------------------------------
---------MVRVLDDLEIRAKEELFINLEPLQRGGITP--ATARKAALSYI-DGYSTCD
FCL---GTLHLIKKPPIADFL-VQAAAFLGMDTAMLTHGCREAKFAVMHSITRPGQ----
-----AIVVDANRHYTSYVAAERAGLKVYEV-PSTGYPDFKINPDEY-SNVIDKVKADVG
-ALPALVLLTHVDWIYGNLVDAKKVGKICKEYKVPFLLNTAYSSGRIPINGKELMADFVT
CSGHKSWAAGAGTVGLLATSKDWITKIFRTS--------EAYKVKPLEILGCS-TRGAST
IALIAAFPFVKER--VKHWNEEVEKARWFSTQMEALGDIKQLGDKPHNHDLIRFETPA-F
DKIAQKHKRRGYFLCDELEKRKITGIKAGKTRAFDLSTYMLTREQLGYVLDSFKDILKKL
S------------------


#Mega
!Title PylRS alignment (N-terminally trimmed);

#Syntrophaceticus schinkii
VTWTATQKQRLSELDAD----EALHDQVFADTAEREEH-FKQLERKLAAKNKEELLALKN
DSLRPLLCQLESRLVDRLTKEGFVQVVTPILLSRGMLNKMTITPEHPLVKQVFWVDK-DK
CLRPMLAPNLYHLLRDLRRLWGK-PVRIFEVGPCFRKESQGAYHLNEFTMLNLVELGDLE
G-QQEERLRELANIAMDAAGIKN-YRLEKDECHVYGNTIDVISGD-LELGSGAYGPHKLD
PPWGIVDPWVGIGFGLERLVVTVKGHKNIRRAGRSLSYLDGARLNI-----

#Desulfotomaculum gibsoniae
INWSISQKQKLIELNGK----SELLDLRFQDKQKRDQT-FQKIEKELVKKNKDHLLELKE
VIHRPLLSSLEIQLSNLLCKTGFVQVNTPIILPKAMLHKMTITPEHPLYKQVFWVDN-NK
CLRPMLAPNLYHYLKILDRLWSK-PVRIFEIGPCFRKESQGAQHLNEFTMLNLVELGVDK
G-KQTERLKELGSLVMEEIGVKN-YEFVETESEIYGITVDVVFDD-LELGSGAFGPLKMD
EQWGIFEPWVGIGFGLERLAMTLQGHRNIRRVGRGLTYLDGSLLNI-----

#Desulfitobacterium hafniense
SFWTKVQYQRLKELNAS----GEQLEMGFSDALSRDRA-FQGIEHQLMSQGKRHLEQLRT
VKHRPALLELEEKLAKALHQQGFVQVVTPTIITKSALAKMTIGEDHPLFSQVFWLDG-KK
CLRPMLAPNLYTLWRELERLWDK-PIRIFEIGTCYRKESQGAQHLNEFTMLNLTELGTPL
E-ERHQRLEDMARWVLEAAGIRE-FELVTESSVVYGDTVDVMKGD-LELASGAMGPHFLD
EKWEIFDPWVGLGFGLERLLMIREGTQHVQSMARSLSYLDGVRLNIN----

#Bilophila sp. 4_1_30
MIFSEEQQRRLGELGAT----SEDLQAGFTDSAERNRA-FQRLESRLVTEQHERLDALCE
GPRRPFILELEERLSAVLRTAGFLQVHTPIILSRARLEKMGVFDGSIMEKQVFWIDS-KR
CLRPMLAPHLYEYMREVGRLRPR-PVRLFEVGPCFRRETQGQRHANEFTMLNLVEMGLPE
GTDLNARLCELGAMVLDAAGIEG-WRMTGEDSAVYGETSDFVDKNGMELASSALGPHPLD
AAWGIMENWVGIGFGLERLTMAATGESTMAKTGRSLSYLHGIRLRI-----

#Firmicutes bacterium CAG:238
EKFTITQRERLTELDAA----SEVLEREFDTKEERNAE-FKKIETQMAREGRQHLKELVE
ERHITFTEEVSRRLQDWLMKDGYTKVSTPTIITRQMLEKMTIDEFHHLSEQVFWLDR-KR
CLRPMLAPNLYVMMRELKRITNQ-PVKIFEIGSCFRKESQGAKHMNEFTMLNCVQLAVVQ
DGRQVEALKEMAHSAMQALGIED-YELVVEESTVYGSTVDIEIDG-IEVASGSYGPHFLD
KQWGVFDTWVGIGFGVERLAMAMSKSKTIKRFGRSIAFIDGEPLNI-----

#candidate divison MSBL1 archaeon SCGC-AAA382A20
MNLTSSQKQRLREL-GW-----DGSIPDFDNKKERDQF-FNKTATKLKNRNKERFLKLLE
N-KVPSWRRVERKLRNIFYELGFVEVQTPSIISPSLLEKMDIGEESKLYNQIYQIKGEKK
SLRPMLAPNLYRELRYFSRISDEEVIRLFELGSCFRKENGGERHLNEFKMLNAVEMGNIK
D--TKKRLDELISNVFSP-FA-N-YKVEKEKSTVYEETVDVNIKN-TEVASCVIGPHFLD
SNWHIDEPWVGLGIGVERLTRVIEGEPSVKPFGKSYVYQDGIRLDIE----

#Candidatus Methanomethylophilus alvus
VKYTDAQIQRLREY-GN----GTYEQKVFEDLASRDAA-FSKEMSVASTDNEKKIKGMIA
NPSRHGLTQLMNDIADALVAEGFIEVRTPIFISKDALARMTITEDKPLFKQVFWIDE-KR
ALRPMLAPNLYSVMRDLRDHTDG-PVKIFEMGSCFRKESHSGMHLEEFTMLNLVDMGPRG
D--ATEVLKNYISVVMKAAGLPD-YDLVQEESDVYKETIDVEING-QEVCSAAVGPHYLD
AAHDVHEPWSGAGFGLERLLTIREKYSTVKKGGASISYLNGAKIN------

#Thermoplasmatales archaeon BRNA1
MKYTDAQIQKLREY-GN----GDYSGAEFDDASARDKA-FSRDMSAATKDNEAKIQAMFS
KPDRPALTRLMADIAAALTAEGFIEVRTPIMITKDALTRMTITPGRPLYKQVFWIDD-NR
ALRPMLAPSLYSVMRSLRDHTDG-PVKIFEMGPCFRRESHSGMHLEEFTMLNLVDMGPNE
D--AIETLKKYIDVVMKVVGLEN-YDLVQEESDVYKETIDVEING-QEVCSAAVGPHYLD
AAHDVHEPWSGAGFGLERLLALREKYSTVRKAGASVSYLNGAKIN------

#methanogenic archaeon ISO4-H5
CKLTDPQIQRLREY-GH----EPKNESEFETEEERDKA-FTKMMSKLQRENEKGIRDMIA
NPRHHRLMELELQLSEALIKEGFIEVKTPILISKAELAKMTIDENHPLYQQVFWVDD-KR
CLRPMHAINLYNIMRELRGHTDG-PVKFFEIGSCFRAESHSNDHLEEFTMLNLVDMGPQG
D--TTEKIKHYIDIVMKTIGL-D-YELVHEESDVYKETIDVEVDG-EEVCSAAVGPHYLD
KAHNINEPWCGAGFGLERLIMMRDGDGSVKKTGKSVNYLNGYKIN------

#methanogenic archaeon mixed culture ISO4-G1
VKFTDSQIQHLMEY-GD----NDWSEAEFEDAAARDKE-FSSQFSKLKSANDKGLKDVIA
NP-RNDLTDLENKIREKLAARGFIEVHTPIFVSKSALAKMTITEDHPLFKQVFWIDD-KR
ALRPMHAMNLYKVMRELRDHTKG-PVKIFEIGSCFRKESKSSTHLEEFTMLNLVEMGPDG
D--PMEHLKMYIGDIMDAVGV-E-YTTSREESDVYVETLDVEING-TEVASGAVGPHKLD
PAHDVHEPWAGIGFGLERLLMLKNGKSNARKTGKSITYLNGYKLD------

#Methanomassiliicoccus luminyensis 1st
TRLTPAQAQRIREM-GG----TVDPSLAFSSEAERESA-FQRISADLQGANLAKIRRCAE
APERHPIGSLENTLACALAAKGFIEVKTPMMIPADGLVKMGIDESHPLWNQVFWVGP-KK
ALRPMLAPNLYFLMRHLRRSVPA-PLLLFEIGPCFRKESRGSNHLEEFTMLNLVELAPQA
D--ATERLKEHIATVMNAVGL-P-YELVVEGSEVYGTTIDVEVDG-VELASGAVGPLPMD
KPHGITEPWAGVGFGLERIALMRTKEQNIKKVGRSLVYVNGARIDI-----

#Methanomassiliicoccus luminyensis 2nd
FDMTPSQKQRLREL-GR----VPDEGAAFSTAEDRDAA-FIKEVAYYQSYNRNVVRDALD
APKRHPLSHMEEVLAQALVDEGFLDVKTPTIISGDSIRKMGISCAHPLNKQIFWVDG-TR
CLRPMLAPNLYFLMRHLKRNAQL-PLRLFEIGPCYRIETHGSDHLEEFTMLNLVELAPQG
D--PLAQLHHHIATVMGAVGL-D-YQLCECDSEVYSRTIDVEVDG-SEVASAALGPHALD
RAHGIEDPWVGVGFGLERLLMSKSAESNIRKVGRSLIYLQGARIDV-----

#Methermicoccus shengliensis
IGFTDTQVQRLKELGGD----QKIVGCRFSSVADRDEV-FETTVKNLVEENREKLRRMAH
SPSRCSLFELEDRLASTLVGMGFMEVATPMLLSASNLKKMGIDESHPLWEQVFWVDK-KR
CMRPMLAPNLYFLLKHLKRNIKK-PVRIFEIGPCFRKESQGSRHLEEFTMLNLVELAPDC
E--PTERLTELIEKVMGQIGL-E-YRLKNESSEVYGNTVDVEVDG-VEVASGAVGPHFLD
GAYGITDAWVGVGFGLERLLMVMEGHSNIRKVGRSLVYLNGARIDI-----

#Acetohalobium arabaticum
MKFTQAQQQRIIELDAEGKYKGYIKDKEFDSSKEREDD-FQKIEDDLVNKNKKRLEELQG
RHQQPVLRRMEFRLVELLKESGFMEVVTPIKLAKGHLEKMGIDEEHSLWKQVYWLEEDNY
CLRPMLAPNLYYLLGYFENSLSK-PIKIFEVGPCFRRESRGSKHLSEFTMMNLVELGPKG
D--PTERLKSLIAELMDYLEL-E-YELEEEESVVYGKTIDVVVNG-IEVGSGAVGPHLLD
EAWNISDSWVGVGFGLERLIICKEEFESIKRVGRSLIYQDGVRLNI-----

#3300000575.a:SL_4KL_010_BRINEDRAFT_100017023
MKLTSGQRQRLKELDIFGEH-SEIFEKEFNSREEREQA-FKSLENELAGKNQDRIARLTE
KKFRPDISRLENELQEILAEINFTEVKTPIRLARGKLERMGITAEHPLWQQVFWLEGKDY
CLRPMHAPNLYEILSRLEKTLVS-PVSIYEVGTCFRKESSGSQHLSEFTMLNLVELGPEK
E--PADRLKEIVEEIMSGLNI-D-YRIAVEDSSVYGETIDVMVNE-LEVGSGAVGPHKLD
QAWDISRSWAGIGLGLERLLMAAEGYASISRIGRSLIYQDGARLNV-----

#3300002481.a:JGI24020J35080_100005770
LTFTDAQKQRLKELMLE----EEELILKFKSSEEREEV-FKKISYRLTTKNKERLELLRS
YKKRPDLRKIESKLVSLLTSIGFTEVVTPIILSRGLLERMNIHEGDHLWNQIFWIDR-DH
CLRPMLAPNLYFLLGHLGRLWPR-PIRIFEIGPCFRKESKGARHLPEFTMLNLVELSPEK
D--PEERLKELITKIMDALEM-E-YELSYEDAECYGKTLDVHSRG-IEIGSGVAGPHELD
KNWGIYDPWAGIGFGIERIILAKEGFANIKKIGRSLIYLDCVRLDII----

#3300002481.a:JGI24020J35080_100014672
MEFTHTQKQRLVELGAD-----DFISMRFKSKDERNKK-FEEIVKKVVADTKRNLEIIRR
ISKRPYLKIIEDEISKKLRKNGFIEVSTPILIRKEALQKMGIDDEHPLMNQIFWVDG-NT
CLRPMLAPNLYTLWSRLRNIWKP-PISIFEVGPCFRRESKGTHHTQEFTMLNIAELSPEK
S--AEERLVEIAKLVMDVVGV-E-YELVWEESEVYGESLDVVVNG-IEVASGAFGPHKLD
ENWNITEPWAGIGFGLERLVLACEGGANITRFSRSLMYLDGWRLDIK----

#3300005860.a:Ga0080004_119822112
VRFTLGQIHRLIELGAE----PAEIDRDFESEAKRNEE-FNRIAGDLARKNLKDIKDFLE
RKKKPLVRLVEEKLRETAIRLGFSEVVTPIIIPKSFIKRMGINEEDPLWQQVMFIDD-RR
ALRPMLAPNLYVVMAKLSNIVK--PVKIFEIGPCFRRETDGRYHLEEFTMFNMVELAPEG
D--PKTRLLDYIDAIMKEVGL-N-YVLSVEPSKVYGETIDVVVDG-IEVASAAIGPKPMD
ANWGVHEPWIGVGFGVERLAMLIGGYRSIARCARSLTYLNGSTLSVIKLKR

#3300009598.a:Ga0105154_10078622
IRFTRGQIHRLIELGAE----PTELERDFETEAERDKE-FNKIAENLARKNLKNIKDFLE
QRRKPLVRVIEEKLRTTALRLGFSEVVTPIIIPRLFIKRMGIDEGDPLWKQVMLIDD-KR
ALRPMLAPNLYVLMAKLSNIVR--PVKIFEIGPCFRRETGGRYHLEEFTMFNMVELAPEG
D--PKERLLDYIDTIMRDIGL-N-YTISVEPSNVYGETLDVVVNG-IEVASAAIGPKPID
ANWGVREPWIGVGFGVERLAMLVGGYNSIARIAKSLSYLDGSTLSVIKLRW

#3300002481.a:JGI24020J35080_1000009179
ITFTPSQKQRLIELGAE----LSKLEASFTSPREREKV-FNSLAEKLTRKNRETIKKLKD
SGKRPPLKTVESKLVNVLTSLGFVEVVTPTIIPKTFISRMGIEKESNLWQQILWLDG-RR
CLRPMLAPNLYFVMGKLRRFWK--PVRIFEVGSCFRKDTRGSFHTEEFTMLNFVELAPDA
N--PRERLAELIAKTMGGLGIGS-YEVRSESSEVYGETLDVLVKD-VEIGSAAIGPKPMD
VNWGIVDPWVGLGLGIERVAMVTGNHHPIARVSRSLIYLNGARLDIK----

#3300005860.a:Ga0080004_11365477
ISFSITQKRRLTELGAS----SELLEKSFKNVEERDEF-FDNCVSELVSSNKNKLKNLVH
KGFCPSLRTIERKIQEASILQGFTEVSTPILMPSSYIRRMGIDESNKLWKQVIWITN-NL
CLRPMLAPNLYYVMRRLRRFLR--PVRIFEIGPCFRKEEEGLLHSTEFTMANIVELAPDR
E--PLVALRDMIDKIMTSIELIN-YDIKESPCAVYGKTFDVLVND-IEVASGVVGPIPID
INWGITEPWAGVGFGLERLAMLKKGLNRIKPVVRSLTYLDGILLIM-----

#3300005860.a:Ga0080004_109639435
WEYTEQQKNKLKSIGVK----KELLGRKFESLMERE-----LTYSEILKNYSDQIKIKLN
NKKFNSLRRVEDKIRKKLLELGFIEVITPTLLSRDSIIKMGISESDPIWDQIYWLENHSK
CLRPMLAPNLYVLMNRLRKFIS--NVKIFEIGSCFRKE-RGRRHLTEFTMCNAVIFPPSK
Q--PQDELNELIQELMKVLNINE-YKIVDESSSIYGKTIDIVSEG-IEIASCVIGPVDID
VNWKIDNHWVGIGLGLERLLMVINKSEKISEYSKSLDYLDGIYIGGV----

#Methanohalobium evestigatum
ETFTSAQKERINEL-LL----PGEKIPF-----SNEPSKFKEIESELVNKRRNDFKQMYE
NDREEQIAKLERTISQFFVDKGFIEIKAPIIIDIDSVKKMGIDTDHKLSKQIFYLDN-KH
CLRPMLAPGLYQWLKNFDKILPD-PIKIFEIGPCYRKESEGSQHLEEFTMFNFCQMGSGA
---NRENLLNHIDDLLKHLNI-D-YKIIDDNCHVYGETIDIVHGD-LELSSAVVGPVPID
MNWGIDKTWIGAGLGLERLLKVKHGYKNIKRASKSHSYYNGISTNL-----

#Methanohalophilus mahii
PDYTPAQKKRITTL-LS----PADDLSS-----IKELPTFKELETELVKKRKQDLRQMYE
DDRRHQLAQLERDISLFLIEKGFMEVRTSVLIPAKFIERMGITEEDPLYKQIFRVDE-NT
CLRPMLAPGLYNYLHNFDNIMPD-PLKIFEIGTCYRKESDGKEHLEEFTMVNFCQMGSGC
---TKENLLNIIDDLLKYLNI-D-YEVISDNCMVYGDTIDIMHGD-MEISSAVVGPIPQD
LDWGVTKPWMGAGMGIERLLKVKHKYTNIKRSSRSISYYNGITTNLR----

#Methanosarcina mazei
PALTKSQTDRLEVL-LN----PKDEISL-----NSGKP-FRELESELLSRRKKDLQQIYA
EERENYLGKLEREITRFFVDRGFLEIKSPILIPLEYIERMGIDNDTELSKQIFRVDK-NF
CLRPMLAPNLYNYLRKLDRALPD-PIKIFEIGPCYRKESDGKEHLEEFTMLNFCQMGSGC
---TRENLESIITDFLNHLGI-D-FKIVGDSCMVYGDTLDVMHGD-LELSSAVVGPIPLD
REWGIDKPWIGAGFGLERLLKVKHDFKNIKRAARSESYYNGISTNL-----

#Methanosalsum zhilinae
PAFTPSQKKRLEAL-LM----PEEVIPD-----PSENLNFQELESSLVNRRKKDIVKIYE
DDRENQLGKIERIITKFFVDRGFLEIKSPILIPIEYIERMGITEDKELFEQVFKVDK-NM
CLRPMLAPGLYNHLRKFDKVLPD-PIRIFEIGPCYRKESDGSQHLEEFTMLNFCQMGSMC
---TRKTLENLIDELLEFMDI-E-YEIVSDNCHVYGATIDVLHKD-MELASAVVGPIPKD
ADWGITKPWIGAGFGLERLLKVMHNYKNIRRASRSESYYNGITTNL-----

#Archaeon V1
TGFTEGQRQKLLELGTD----ASEMERLFEDASERDEA-FKTASKKMSRKNRESLEDFMN
RRRKPLVRELEERIRGALISRGFSEVVTPILIGGKEIEKMGISRGDHLWRQIVWVGE-DQ
CLRPMLAPNLYVIMEKLSAIGK--PVRIFEVGQCFRRDTRGQLHLEEFTMMNMVELAPPD
D--PKGRLLDYIETVMKAAGL-E-YVIEPESSEVYGETCDVRVKG-VEVASAAIGPKQMD
CNWGISDVWLGVGFGLERLAMFAGGHNSVARVARSLFYLDGSKLNVK----

#3300005645.a:Ga0077109_10022558
ISFSKVQKQRLRELDAP----VDFQEKSFSSVQDRDKA-FRNIEQLLVRQGKQHLLEVRN
VRHRPALCELETKLTDVLIKNGFFQVITPIILAKGLLAKMSITPEQPLFKQVFWVSD-DK
CLRPMLAPNLYYLLAEFARLYDK-PIRAFEVGPCFRKDSKGGHHLNEFTMLNLVELGSPE
E-GGRQRLEELAAIVMDAAEIKD-YQLTSTCSEVYGETIDVVAG--LEVGSGVIGPNALD
SQWGWVDPWVGIGFGLERLVMVNKGYQNIQRVGRALMYFDGVRLNI-----

#3300001749.a:JGI24025J20009_1000174613
INFSRVQKQRLRELDAP----LNLQEKSFPSAQERDTE-FRNTEQLLIRQSKQHLVETWN
IHRRPALCALEAKLTNVLIKNEFVQVVTPIILAKGLLAKMSITPEQPLFKQVFWISE-DK
CLRPMLAPNLYYLIGKFARLFGK-PIRVFEIGPCFRKDSKGGHHLNEFTMLNLVELGSPE
E-KRKQKLEELAAIVMDAAEIQD-YQLIPARSVVYGETIDVVAG--LEVGSGVIGPNALD
TQWGWIDPWIGIGFGLERLIMVSKGYQNIQRIGRALIYFDSVRLNI-----

#3300001753.a:JGI2171J19970_100115763
INFSRVQKQRLRELDAP----LNLQEKSFSSAQERDAA-FRNTEQLLIRQSKEHLLETWN
IHRRPALCALDTKLTNVLIKNGFVQVVTPIILAKGLLAKMSITPEQPLFKQVFWISE-DK
CLRPMLAPNLYFLIGKFVRLFEK-PIRVFEIGPCFRKDSKGGHHLNEFTMLNLVELGSPE
E-QGKQKLEELAALVMDAAEIQD-YRLTPARSDVYGETIDVVAG--LEVGSGVIGPNALD
TQWGWIDPWIGIGFGLERLIMVAKGYQNIQRVGRALMYFNSVRLNI-----


SepCysE protein sequences
>gi|11387334|sp|Q58876.1|Y1481_METJA RecName: Full=Uncharacterized protein MJ1481
MRVEYSKDLIRKGISTISQLKKAKIRVEKDDKKISYKDAKPGKIDVNEFKKAIYLLIEADDFLYKKAPKHELNEEEAKEFCKLIIKCQEHLNKILANFGFEFEEKEIDEGALYIVSNKKLFKKLKNKNPNLKVVCTEGMLDIEDMRAIGVPEKALEGLKKKVEIARKNVERFIEKYKPEKIFVVVEDDKDELLYLRAKNLYNAEKLDADEILD
>gi|499179279|ref|WP_010876819.1| hypothetical protein [Methanothermobacter thermautotrophicus]
MDRIRFEQAEKLIRKSCINLKREQGFRDASDGVIDTGKLQEAMMELIDAEEYLYTSLPTHELGGEDASEFCRKLIAAREAIDHILADFGVLERQDPSERIREAARGKLIIVNNSSVKKLLVKAGVEAQNILVAGAPLSVDDMREINPKIPESALRGIEKKIEHLRNDIERKLDALEDVLVVGEPDKSTVLLAARAEELYGADSRLMENIKDLNSEDILELLS
>gi|503632181|ref|WP_013866257.1| hypothetical protein [Methanothermococcus okinawensis]
MNRLNDSKILIKKAVNTIDKLDYELKLKNNTKTKTISESKSISYKDAKSGTINVDEFKKAVYAIIEADDYLYKKAPLHNLNDDEAKMFCKLVFQTQKHLNNVLKAFGFEFEEGAQLDESALYIVSNKKLLKNLKNKMPNLNIISTEGILEAEDMKVINPNMPEGALKGIEKKCNITKEQITKLINKLNPSKVIVIVDDSNKADELIYIRAKELYGAEKISIDDIDL
>gi|499484521|ref|WP_011171161.1| hypothetical protein [Methanococcus maripaludis]
MSETQYSKELIKKAVETISKAKTVSATQNFEKNENKKTFSDAKSGKIDTIEFKKAVHSLFEADEYLYKYAPNHDLDEEKARAFSKLLFDAQKHINNVLGGFGFDIETVALDGQALYIVSNKKVLKSLKDINPDLNIISTEGVLEIEDMKVVNPKIPEKALLGIEKKCKITKEQISKVISNISPSKVVVLVKNGDVADELIYKRAKELYNAEKLNADEIL
>gi|757148822|ref|WP_042703019.1| hypothetical protein [Methanobrevibacter arboriphilus]
MKDFRLKQAQHLLKKSAVNSEGDFKLKAPNSANINVKIFEHIFKDLIAAEDFIYSSLPKHQLSEEEAGEFTKFLISARNNIDSILTDFNVIEKTEEKIDMSKLTENILFITSKNNFKKLLKKLGVDVQRIIVASVPLDVMDIKEINPKIPESALKGIETRVKHIHNDINRKKNSLNPEKIIVLAENDLNGQLLGKRAEEYYDAIIYLNDNLKDLNDNELIEIIENN
>649737992 YP_004003962 hypothetical protein [Methanothermus fervidus DSM 2088 chromosome: NC_014658]
MDRIRIKQCERLIKKASKSKPKIKSKDKITINKVKEGQIDIETLNDAINKLLESETFIYKTLPSRKIKGKDAQEFCNNLMFVKKKIEKILSDFDVIKTVEEEIPKEILIVTQNTRIKKRLQDIGVDPRQIIVAGVPLALEDMKEINPNIPEHALKNIEKRIKVIKNEISRKIKHFEKILVVVQKDKSGKLLAKRAKELYNAEVMFCEDPKDIDLHKLSKFWGG
>2625509754 hypothetical protein [Methanopyrus kandleri AV19 (re-annotation) : Ga0076456_11]
MTRVERLEELLSHAVSQRSGRIRVVTDVDELRLDEEVVRRIVHRAAELDHALVESAPEHVLRGERAQEVARALDDILRSVLELVGVEEETERDLDDLFKDSVLVVVRGRERKALRELVDAPIVQTGGPLVPEDYRKVNPNLPEKLPEGLVKSVERARRELEEYIRKSGAKRIVLVREEDDRVGEVLEEELPEVAEELGVDHEVIVVPDFTELSPSDLLSGRTK
>GB (W8A-19) archaeon No. 7
MPFLIPPDVVDKITSAVDDLITLWSIIRRSSPSHVLNGDEEKSFIERLKRASLRLSEALERLDVKESGLEGLESSLSTLSPHTTLILVASPSLRKKLLGMGIPRSRVLAIGGPLTVDDMKKLNPDISDQAVKGLEARIERFWRDLERRAKEIKDVILLLGEGKRADDMIARRSSLISERTGVNVRVIRLKRFDDPSLKVLLRFFGG
>GB (AK8) archaeon No. 8
MNKWESDVVNFGYEDIEGIRKAVDALITILCEIRSFAPGYDLDSAAEERIRSLLRAVREALGGTMEKFGLQRLEEGGKSEVGRVDLSGILFVVVSSSARKRLLDLGVEPRFVVVTGGPLNAVDMKALNPGISDEALEMVGRKVESVWREIESRAAEAERIVVLVEEGSEGGMMVSERADEIQRRTGVQTSVKTFPSVRGLSLEFFEEFLGEEDV
>CP (AK8) archaea
MESGLLNFGSDDIVRIRGALDALISILCVVRAFAPNYNFDSAAEENIRGLLKNVRDILDLTMDKFGLGKLGEAKETEVKAIDFSGVLFITVSSSTKRRLLDLGVDPRFVVVTGGPLDVGDVKILNPSISDEALETVRRKVEGVWRDIERKASEAKKILILAEAGNRGDLMVSERAGEIERRTGLKTLVKTFSSLKDLSGEFFEDFLGDANV
>Jinze hot spring (W8A-19) archaeon
MPLSLSLDAARKLSSTIEALLTAWSLVRASAPRLILKEKEERDFVEKLLLAHRALSELLSLLGLDKQENELISALSELNPNETLLLVVPPSLMRRLVGVGIPHERVISIGGPLSAEDAKALNPHLPEEAMKGVEARLKNFWRELERKIKGARTVLFILEKAGKVDELIAKRASMLSEKFGVDVKVVYLTNLDSCVEILPRFFRRE
>LHC4sed hot spring (W8A-19) archaeon
MLFPFSIDAVRKLSSVIDALLTAWSLVRMSALHSILNEKAEVEFAEKLLSAHRVLSELLSLLGLSQRENELGNVVSELDPNETLVLVVSSSLMRRLVGNGVPREKVISIGGPLSVEDARALNPNISEESMRSIESRLKTFWRELERKIKGVRTVILILERGGKVDELIAKRAGMISERFGVDVKVVYLTNLDSCVEVLPSFFRGS
>BOG (Asgard) archaeon
MDIQQLQAELSGLINGLIDAKAKLDGEIPTIQATFSERDQVTKELLRVHGKFTKILETLDLIVEXAIPAKTSDTSVQSNIDPERYLAISCRNAIKEKLDSFQTLTLDQVTTGGPLEARHYKSLNPSIPDAALANIEKKIEKVFKEIQKKSANKEIILLIGVKDENTDQTLFESKELIESRVNKPVFIIEVESLQKMTEIDLRKLIVSSEEAR

CP (AK8) archaea selenoprotein sequences
3300010308.a:Ga0136652_1000089
>HdrA
MSNNEKTASEPAIGVYVCEUGVNIGGVVNCKEVAEFASKLPNVKTSKTYSFFCSDPGQKIIKEDIEKLGINRVVVASCTPKTHEPTYRTLLQSAGISPYYFQMVNLREHCSWVHYKNPKEATEKAKHLVAAGVARARLLEDVPQRTVKVEPAVMVVGAGIAGISAAITVANHGLKVYLVEKKPTIGGLMAQLDRTFATDDCAIUILAPKMVEAAKHPNIKLLTYTEVKELKGSPGEFIVTLLKKPRYVNESKCTGCGACSEKCPVEVPDEFNNGLGTRKAIYIPFSQAVPKIALISMEDCIQCKSCERQCLAGAIDYDQKPEEFEVKVGAIIVATGADIYDAAERKDYGYGIYENVITQMELERMLSPTGPTFGHIYRISDRKVPKRIAMIQCVGSRDIKTNPYCSEVCCMVAMKNAKLIKQHDPNTEVTIYYMDMRTVDEGHEEYYMGARGHGVKFVRGNVARVKEDPKTKNLLLRVEDTLTGEIKREEVDLLVLSTAVIPSKSGTELGAILGIDMSASGFLRPVHSGLSPQETKTRGIYICGIAQGPKDISYAVSSAKAAASAATAWCLAGEATLELITPIVEEKLCIGCRRCERNCPFGAIKVIEGVAKVDEASCKGCGICVASCPAHALDLRYYRDKQIREETAALVET
>VhuD
MTYQVVVYACWKUGYGVADLTGVSRVAYDPALRIVRLRCTGRLDSSLLLEPICNGADGVMVVGUHIGECDFEKGNIMAEKHVNFVKRILERRGIGGDRVNMYHCSAAEVGRFKESVEDMMGKIRKL 
3300010308.a:Ga0136652_1000328
>VhuU
MPEIDPKILNLLEMVMRSYDCULSCSAHLVILDSEGKEIASKEIVIGSG
3300010308.a:Ga0136652_1000397
>SPS_N
MEDYRLTKAVTLYGUSCKLPQTKLLNLLEKVGVKKDPSGRVVGGIGEDAAVIKIQKDLVLIETVDFITPNVDDPYIQGKIAAVNVTNDVYNVNHNDLHA
>SPS_C
MVSFLAILGIPENMPLKMAEEMLRGINDLISELGTTTITGGHTIINPWPLVGGVASGIAHPDQIVTHKDAQPGDILILTKPLGTQPAMAVYRGLRDPAVAEQILKLVSRKEAEEMVEKGIKLMTTSNKPVAEVMRVVKPHASTDVTGFGLLGHTQGIAESSKVDITIHTLPIIKGTLQISDLFGYGLQSGESAETAGGMILFVPSDLKENLEHELDRRSVTHYEIGKTSKGSGKVDFSKAEILEV

LHC4sed (W8A-19) archaeon selenoprotein sequences
3300008019.a:Ga0105158_1000576
>HdrA
MVEKEKKAEEPVIGVYVCKCGVNIGGVIDTEAVAEYASKLPNVKVAKTYSFFCSDPGQKMIKEDIETQGINRVVVAACTPKIHEPTYRALLQSAGLSPYFLEMVNLREHCSWVHMTDPQAATEKAKKLVAAGVARARVLEDVPQKRLPVKPAVMVIGGGIAGISAALSVANHGIKVYLVEKEATIGGLAVRLDRTFPTDDCAIUILAPKMVEAAKHPNIEILSYTEVLDVKGIPGSYTVKLLKKPRYVDEKKCTGCGSCTEVCPVAVPDEFNYGLGYRKAIHIPFPQAVPKVALISMDDCIQCKSCQKECRAGAIDYEQKPEEIELTVGSIIVATGFEIYDISKYGDYGYGRFENVITQIELERMLSPTGPTGGKVVRISDGKHPRRIAMIQCVGSRDVKRNPYCSEVCCMVAIKNAKIIKQESPDTEVIIFYMDIRGIDEGHEEYYLATREYGVVFVRGRVAEVIEDPETKSLKLLVENTLTGELLETEVDLLVLSAAVVPSEGTKKIAQILGLNRMPSGFLAPQHVALNPQETKSPGIYICGAALGPKNIPYAVSSARAAAAAATAWTLSGEVAVEMMIPEVNKELCVGCRRCERTCPFGAIKVVDGKAVVSEVQCKGCGSCVASCPAHALDMRYYRDKQIIEEIKAAIKVH
>VhuD
MPTPSFNIISFCCWKUGYGASDLTGVSRVQYDPSVIVVRVKCTGRVDVKHILEAIRQGADGVMVVGCHPGECDFGDGNMKARQRVEFAKKVLDKVGLGGDRVNMYNVSAAEIGRFRDAIMDMIEKLKKIGPNPLRG
3300008019.a:Ga0105158_1000238
>VhuU
MVEVDPKLLNLIEMIMRAYDCULGCGAHLVVLNHKDEKIAEKDIKLSES
3300008019.a:Ga0105158_1000017
>SPS_N
MLEYRMTKVVSLHGUACKLPQADLRRLLEMAGITVPRGVLSGPEEDAAVIRVRDDMVLVKTIDFITPNVDEGYVQGKIAAANATSDVYSAATVIGVARTSEGGNGDSRLPSCPRNTH
>SPS_C
MPVEIAVDILRGIKDYLDEVGGAFVGGHTIFNPWPLSGGEVTAIAHPNQIVYQRGAEVGDVLVLTKPLGTQPAMAVYRTMKDPETCEIVLSVLSRKEAEELVKKAIRFMTKPNKSVAEAMQEVKPNAATDVTGFGLVGHAENIAKESGVDIEVQAIPLMKNAIRVSELFGYGLERGEAAETSGGILAALPRERVDEFMDALRRRGVTAYVVGQAKQGGGKVTVKPDVEIIEV

BOG (Asgard) archaeon selenoprotein sequences
3300003218.a:JGI26339J46600_10001041
>HdrA1
MSENSDNIIKEPVRIGVYVCQUGVNIGQFIDTEQVRTFAESLPSVLVARTFTFFCSDPGQEMIKRDIQELGVNRVVVAACTPKIHEPTYRDLLVSAGLSPYYFEMVNLREHCSFVHSKDKNAATEKAKSLVAGGVYRAAVLEDVPRKEVQVTRSVLVVGAGIAGMNAAINLANHGISVHLVEKEPTIGGRMAQLDRVFSTDDCAIUIVAPIMVKASKHPNIKLHTYAEVVEVDGITGNYSVKIKQKPRHVNPKLCTGCGSCIAKCPVEVPDEFNRGIGMRKAIYIPFAQAVPKIAVIDKDACMNCGLCQKTCQRGAIDHDEQESFDTVTVGAIIVATGWDEYPVQKYGTFGYGRYQNVITQMELERMLSPVGSTHGHVLRISDKKTPSRIAMIQCVGSRSMHENANAYCSSVCCNLALKNAQLIKQEIDGADVMIFYMDMRCWDKQNEEYYRRIREKGVIFIRGIPGDVKEDPMTGNILLTYENTLEGTVQEVAVDILVLSAGMEPSAGTREIASVLGMDKSPGGFLKEIHQCLSPQETKSAGIFIAGCAAGPKNIPYSVSSALAAAADASAIVSSDVFAKELITAIVDTERCVACHRCEKSCNYDAIIVNPETNVAEVNDLNCKGCGICVSSCPAKAIVLRYYRDDQLGGKVYAILKDSSKAPQVVQENIDASTSMQPD
3300003218.a:JGI26339J46600_10000997
>HdrA2 (partial)
MNKGIKMSKKEEIVPQRTGTSQNLKVAFYSCQUGINIKGKVDCDELVNFAKSIDGVAISKTYSFLCTESGQDLIMNDIRELDIDRVVVAAUTPRTHEPIYRSSIAKAGLSPYYFQMVNVREQCSWCTESKEDATEKAKILVKSGIERSKHLEIVPIKTVEIKKSTMVVGGGITGMNAALDLANQGIKVHLVESSTTIGGKMAQLDRTFPTDDCAIUILGPKMLDVSRNQNIEILSYSDVKKVEGTVGNYHVTVETKPRYVNTDKCTGCGACNEACPVKKIPNEFNEGLDNRKAIYIAFQQAVPKKATIDADKCLYMKTGACQFCVKACPAGAIDFEQEPKVVEFDVGSIIVATGLGLYKPTEYGYGTYANVINQLQLERILAPNGPFGGHVRRVSDENELKEIVFIQCVGSREPERPFCSGVCCMIALKNAKLLKEELPDANITMCYIDVRTNEKGFEEYYQRAKDTGIRMIRGKVGELVEDPETKNLKIRVYSSLTDEIININADLVVLSTAILPSKNTEQIAKVLGLEVDKNGFLTESHFGLMPQETKMKGVYVAGFAQGPKNISYSVSQARAAASKVAELTSAGKIDLELITAEVNKNACISCKRCEKVCNAGAIKVVDNSGAVVDDTSCEGCGVCASCCP
3300003218.a:JGI26339J46600_10001173
>HdrA3
METEKHEIENIGIFVCRUGINIAGIVDVPFLVQELGKIDGVKAFEYLSFCTESGATVIADAIKKHNLKRILVAACTPRTHEPVFHAVLRDAGLPPRMLEFVNIREHCSFVHMKAKDRATQKALKLINASLERARLLETVPTKTVEVTPRALVVGGGIGGLSAAVELGNQGYDVILVEKKTSIGGRMAQMDRTYPTDDCSIUILGPKMLEANRNENVTIMTNCQIQAVNGYIGNFEVDIKHNPRLVIEDRCNGCGQCADVCPIYVSNYFDEGLSARKCIDKAFAQAVPSVYDIVLDKCVHCYACVKSCELDAIDFSQQPEITREKVGAIVVATGWDLYNPPVPNMYGYGIYDNVVTQIELERLLAPNGPNLGHLTRVSDHNTPKRVAFINCVGSRGKVYPHCSNVCCMLSVKNAQLIKAEYPDAEIIVNYIDMRCAGRDYEEYYERARKAGIIFIKGLPSSLQEDPLTKNLTLTFESEDVGEIMTFEADMVVLSSASLPSKGTEEISQVLKLERTSVGFLKETHARLNPIETKTPGVFIAGSCQGQKPIDATVNQGKGAAAAAASLLSKGTYEIELIRAYPDGVRCSECHECVEACPYHAISIQHGAGITVDPIMCRGCGLCESACKSQAIKLRYFREEQYNALIDTLLTPEG
>VhuD
MTFEPKIIVFVCWKUGYGSADMAGTIRGQYPPSIFPILVPCTARVSADMVVRAFTDGADGVLVVGUYPGECGYETGNYHARRLIEYLQVVLNAAGIDGERLHLEFCSAAEGRKFQQTAIDMNEKILKMGPNPGTKAQT
3300003218.a:JGI26339J46600_10002996
>VhuU
MVTGEEEKDSHPELNVLEMVIRIYDFULSCAAHSIVIEKGGKEIYRRPLNMGPVNL
3300003218.a:JGI26339J46600_10012099
>SPS (partial)
MEENTKKTFDLTSYSKVYGUSCKLPESDLKEMLGMTSIYNNPSTYGEDASVIDIGXSRYLVQNIDVFTPIVDDPETQGRIAACNITNDLFACGVLNVTTMLSFLATPVDVPKWALAGILNGMQAFANDLGTTITKGQTIQNPWILVGGAASGFVEKENLITHAGVKPGDSIILTKPLGIQAIMALSRLIKNPEVADSLLEALSSDEFEPTIQRAIDLMTTS

tRNA-Thr-ED protein sequences
>2684030661 threonyl-tRNA synthetase [candidate divison MSBL1 archaeon SCGC-AAA259A05 : Ga0126884_1001]
VKVLFIHADYLSYRVKEKTPSAEDIEGSRKKGSMDEPLITLISIESSDERSEAEKLAEATLKTISDVSSKIGVKNIALFPFAHLSEDLASPDFAVSVLKETESKLKEEGFNTLRVPFGWYKEFELKSKGHPLSVLSRILAP
>2678493176 threonyl-tRNA synthetase [candidate divison MSBL1 archaeon SCGC-AAA259D18 : Ga0126887_103]
VKVLFIHADYLSYRVKEKTPSAEDIEGSRKNGSMDEPLITLISIESSDERSEAEKLAEATLKTISDVSSKIGVKNIALFPFAHLSEDLASPDFAVSVLKETESKLKEEGFNTLRVPFGWYKEFELKSKGHPLSVLSRILAP
>2684034245 threonyl-tRNA synthetase [candidate divison MSBL1 archaeon SCGC-AAA259E17 : Ga0126888_1001]
MKALFIHADYLSYKVKEKTPSAEEIEGSRKNGSMDEPLIALISIESGDERSEAEKPAETTVETISDVSSKIGVKNIALFPFAHLSEDLASPDFAVSVLKETESKLKEEGFNTLRVPFAWYKEFELKSKGHPLSVLSRTLAP
>2684035454 threonyl-tRNA synthetase [candidate divison MSBL1 archaeon SCGC-AAA259E22 : Ga0126890_1002]
MKLLFIHVDYFNYKVKKETELAEKIDSSQRSGETENSLLVLLSVEKEDEESKTGSDKLVSRALNEIEEIVTQIKVENITLFPFAHLSESLSSPDYAISVLKKLEKKAKKSGLNTLRVPFGWYKNFELKSKGHPLSVLSRTIHL
>2684044923 threonyl-tRNA synthetase [candidate divison MSBL1 archaeon SCGC-AAA259E19 : Ga0126889_1116]
MKVLFIHADYLSYKVKEKTPSAEEIEGSRKNGSMDEPLIALISIESGDERSEAEKPAETTVETISDVSSKIGVKNIALFPFAHLSEDLASPDFAVSVLKETESKLKEEGFNTLRVPFGWYKEFELKSKGHPLSVLSRTLAP
>2684043507 threonyl-tRNA synthetase [candidate divison MSBL1 archaeon SCGC-AAA259E19 : Ga0126889_1001]
MRILLIHADYVNYEVETRGKFAEEITPDRKEGSLEDPLIAFVSIEENDENSGTESGDLVEKAFREIRKVASKIKVRNVALFPFAHLSESLSSPDFAVSVLKDLEDRFKKSKFNSFRAPFGWYKEFEFRSKGHPLSILSRTVRLN
>2684050137 threonyl-tRNA synthetase [candidate divison MSBL1 archaeon SCGC-AAA261G05 : Ga0126900_119]
MKLLFNHVSNFEYRATEKTKIGTELEEGEKTGETGESILVKVCSESGDDDSTVERTLDEIKDISNQVGVKSLVLFPWAHLSQDLASPDAAEEMMEEMKKRLEGEGFEVLKAPFGWYKEWKLESKGHPVSVLSRAV
>2684048943 threonyl-tRNA synthetase [candidate divison MSBL1 archaeon SCGC-AAA261O19 : Ga0126901_123]
MKLLFNHVSNFEYRATEKTKIGTELEEGEKTGETGESILVKVCSESGDDDSTVERTLDEIKDISNQVGVKSLVLFPWAHLSQDLASPDAAEEMMEEMKKRLEGEGFEVLKAPFGWYKEWKLESKGHPVSVLSRAV
>SCGC-AAA261O19_fused_2684049153_and2684048917
MRILFIHADYVLYRVKGKTKAAEELEPGEKQGGMSEPLIAFVSVEDEDENKGRAEELVEKAFEEIQDVAAMVKSKNIAIFPFAHLSGKLASPDFAISILGELESRVRKADYEVIRAPFGWYKEFEFRSKGHPLSALSRSVSL
>2684042338 threonyl-tRNA synthetase [candidate divison MSBL1 archaeon SCGC-AAA259O05 : Ga0126895_1009]
MRILLIHADYVNYEVETRGKFAEEITPDRKEGSLEDPLIAFVSIEENDENSGTESGDLVEKAFREIRKVASKIKVRNVALFPFAHLSESLSSPDFAVSVLKDLEDRFKKSKFNSFRAPFGWYKEFEFRSKGHPLSILSRTVRLN
>2684048119 threonyl-tRNA synthetase [candidate divison MSBL1 archaeon SCGC-AAA261F19 : Ga0126899_124]
MKFLFVHADYVSYEVKQKSKVAEEIDDAHRKGRMENPLIVFLSVEERDENSEKNAKPVDRALGEIKDIASKIKVKNVALFPFAHLSESLASPDYAISVLKELETELEKSEFNLLRVPFGWYKEFEFRSKGHPLSVLSRAVRS
>2684027657 threonyl-tRNA synthetase [candidate divison MSBL1 archaeon SCGC-AAA382A20 : Ga0126904_1105]
MKLLFNHVSKFKYRATEKTKIGKGIGEDEKSGETEEALLIKICSESGDDDSTIERALEEIEDVSDQIGVEIFVLFPWAHLSQDLASPETAEEMMEELKNRLESEGFEVLKAPFGWYKEWELESKGHPISVLSRAV
>gi|282155391|dbj|BAI60479.1| truncated threonyl-tRNA synthetase [Methanocella paludicola SANAE]
MRILAIHASHISWRATKKAKFAEEITKKDGSMDGCVVLFSCVEKQDEVEPARVVEGATREIRKRLGMLKVNKVVVFPFAHLTSALGRPEIALQVLKDLEKSLAGHGYEVERAPFGWYKEYDLKSTGHPLSELSMSICPYEGKSCDASCPYCSHPINISELSKVAPTEDI
>gi|757145807|ref|WP_042700015.1| hypothetical protein [Methanocorpusculum bavaricum]
MKILGIHTDRVWYKVTKKTKIAEPDPVREDEMENCVLLFASVEKSDELSPELTVTSTVESIKLRLSRLGATRVMLFPYAHLACDLGCPGVSQWILKTIQSRLIEEGIETKRAAFGWYKEFEIKSKGHPMADFSMTICPYAGGECESSSKCCQSEVKND
>gi|500158251|ref|WP_011832921.1| hypothetical protein [Methanocorpusculum labreanum]
MRILGIHADRVWYKVTKKTKMAEPEPVREDEMDNCVLLFATVEKSDEVTPELTVSSTVESVKLRLSRLGATRVMLFPYAHLASDLGCPGVSQWILKSIQSRLIEEGIETKRAAFGWYKEFEIKSKGHPMADFSLTVCPFAGGECDKSSKCCQNESKNC
>gi|255513450|gb|EET89716.1| threonyl-tRNA synthetase [Candidatus Micrarchaeum acidiphilum ARMAN-2]
MRFLCWHVDYFRAKPAGRGRSTVVEEGEAVDIEKSAMLLFISFEKSDESSAEDVLDRAVSEIGSIAAQLNEHTIILNPFAHLFGELSSPQFGAEMIEKLAERLRDRKFEVYRLSFGIFYEIELRAKGHKLARMSRKIG
>gi|998177161|gb|KXH76595.1| hypothetical protein AM326_02380 [Candidatus Thorarchaeota archaeon SMTZ-45]
MKLLMFLITNFWFRPEERGNAEGTSFDEALLAWIQSEMHDEADRTGIIRKLVKNIRWLAKKHETSRIILHSFAHLSDSKSTPEFADDIIEETAERFRDREYEVHIVPFGAFHEFKMHVKGPSLAKVFKSF
>gi|735020988|gb|KHO54818.1| Threonyl-tRNA synthetase editing protein [archaeon GW2011_AR21]
MKLLMFHAKEFWYKPHAGLGDAAESDKITDAIVAFIHVEESDKDRVEEIVGKSAGNIQWLAKKNNTDKVVLHSFAHLSSSKAEPETADRIIQKIGEKLKKNLTAQTVPFGRFYEFSIHVFGQSLAKVFKDI
>gi|501690478|ref|WP_012617811.1| hypothetical protein [Methanosphaerula palustris]
MKLLMFNTHEFWFRTFKNSLNEDNDEDIEKTVENSLVIFIHIEENDIERKERLVKKATDNITWLAKKTGRKRVVLHSFAHLSDSKSESTGAQDIFLALQEKLISRGLDTTMTPFGYLNEFRIQVLGDSLAKVWKSL
>gi|503663579|ref|WP_013897655.1| hypothetical protein [Methanosalsum zhilinae]
MKMLMFDTEHFRFETHSKTLENAEDTRSQANIENAAVIFMHVEAVDEERQNKVIKKAVTNLRWYLNKLDKNRIVLHSFAHLSSSKSSPECAVEIISKIKEKLEQRDIEVHTTPFGYFSQFSIHVKGESLAKVFKEI
>gi|501056134|ref|WP_012107602.1| hypothetical protein [Methanoregula boonei]
MKLLMFDTNEFWYKPFQKTLETAQTAVDEAAFADSLVIFVNVEEEDELQKDKSIRKAAENILWLAKKTGRTKVVLHSFAHLSESKSGIGFAEAALQGIRDRLAAKGLTVAITPFGYFLEFKIHVKGESLAKVWKSV
>gi|504866259|ref|WP_015053361.1| hypothetical protein [Methanolobus psychrophilus]
MKLLLFDVEYFWFDTHCKTIESVEDVEIEERIENTAVVFIHAESEDEERKSKIVKSAVGNIKWYLNKVNKDKIVLHSFAHLSSSKSSPEFAMEVIVSIEEKLRNKGINVSTVPFGYLYQFSIHVKGESLAKVFVEI
>gi|851383732|ref|WP_048204916.1| hypothetical protein [Methanococcoides methylutens]
MKMLMFDTESFWFETFSKTLDHVDDIEREESFTDSAVVFIHVEAEDEPRKNKVVKSAVANLKWYLNKVNKKRIVLHSFAHLSSSKSSPEFAVEVILAIRDKLESKGIEAHTTAFGYFSEFSIHVRGESLAKVFKEI
>gi|502801646|ref|WP_013036622.1| hypothetical protein [Methanohalophilus mahii]
MKMLLFDTEYFWFDTFSKTLENVEDIKKEEKIENTAVVFIHVELEDEERKNKVVKNAVKTFKWYLNKVDKEKIVLHSFAHLSSSTSSPEFAAEIISEIKEKLINKGIEVHTTPFGYFSEFSIHVRGESLAKIFKEI
>gi|851355508|ref|WP_048193892.1| hypothetical protein [Methanococcoides methylutens]
MKMLMFDTEYFWFETFSKTLDHVDDTEREEKIEDTAVVFIHVEAEDELRKSKVVKKAVANQKWYLNKVNKERMVLHSFAHLSSSKSSPEFAVEIILAIKEKLDNKGIDVHTTAFGYFSEFSIHVRGESLAKVFKEI
>gi|780807238|gb|KJS12371.1| hypothetical protein VR67_09260 [Peptococcaceae bacterium BRH_c8a]
MRFLIIHVNSFKSIITEKGRSKFAEPPKPAETAVEEALVVLTSVEKADENDPETVAYKAAEEIAANAENLKVRTVVIHPFAHLFADLGKPSVAIRVMQNVQERLAQKGYEVIRTPFGWFNTLEINAKGHPYSRVARIITADS
>gi|818495021|gb|KKR49220.1| Threonine-tRNA ligase [Candidatus Magasanikbacteria bacterium GW2011_GWC2_40_17]
MRSLILHADKFATKVVDKSKWPKGVCPEEKKSDSEEMAKCLVVFFCVEEGDGKKQVDAIYAEILKTADEVKTKNLMISPFVHLSSNIAKPDVAKKLYEQLAGKFIGSDFVVKTSHFGYHKSLLLEIKGHPGSFRYREFY
>gi|647588080|ref|WP_025865235.1| hypothetical protein [Prolixibacter bellariivorans]
MKVLVIYADRFAYEPAIKNLDEVETIEKGAAHENCIVAFIQMEEDDEEKSIASREKKLVNHLKWTARKNDTQYIVLHSFAHLSESKASPGFTKAIFDAAEKRLQNAEYQTAQTPFGYFLDLDIKAPGHSLARIWASL
>gi|818282759|gb|KKP68189.1| Threonyl-tRNA synthetase editing domain protein [Candidatus Roizmanbacteria bacterium GW2011_GWC2_35_12]
MKLLMFRVNEFWYKTFSKTLDNVEKVEKEEKIGKSLIVFIQVEKEDEERKDKIKKKAFENIKWLVKKVNVGEIILHSFGHLSESKSSPEFAQEMINEIKKSLDERNFRVKTTPFGYFLEFKIHVLGESLAKVFKSL
>gi|527028376|ref|WP_020878968.1| hypothetical protein [Desulfovibrio sp. X2]
MKLLMFYGPEFWSKPFRKTLPEAGEAPGELSVSAAAVVFYQCEEHDAGRKAAVLQKTLKNIKWLAGKFSTRRVVLHSFGHLSASKADPGFARGLMDEVRARLESVDYEVHETPFGWLNEWRMHVSGESLAKVFKDI
>gi|672404793|dbj|GAK55471.1| ThrS1 protein [bacterium UASB270]
MKLLMIYAERFAYTTALKALESVPDIQKTHALERALVGFIHVESQDEERLSEVETKLIKNLKWAARKNETTRIVLHSFAHLSESKAAPEATRIVLANAERRLFNADYEVHQTPFGYFLDLDIQAPGTPIARIFKEF
>gi|501881609|ref|WP_012663148.1| hypothetical protein [Desulfobacterium autotrophicum]
MRVLFWYCDNFDWNPAIKTIEEVPDAQPFGCTNAVVAFIHVEPKDLDPASSAETKLVKNSKWLARKWSVNQIVLHSFTHLGEEKADPDMAQAVLNNAQVRLETSGYDTIQTPYGYFLDLSIKAKGHPLARIYKEF
>gi|1027166986|gb|OAG27255.1| hypothetical protein TH606_07855 [Thermodesulfatator autotrophicus]
MKIIMFYALEFSWKPYQKVLASGEEAPEAKALSKAVVIFYQVEAEDPPREKKVVEKLVKNIKWLARKFETKTIVLHSFNHLSTSKASPEESYAIISKAKEKLKRADFELYETPFGWLNEWKMYVAGESLAKVFKEI
>gi|818342085|gb|KKQ24083.1| Threonyl-tRNA synthetase editing domain protein [Candidatus Roizmanbacteria bacterium GW2011_GWC2_37_13]
MKLLMFHVSEFWYKTYSKTLENVDTQEKEEKINEGIVVFIHIEKEDEERENKLRGKAIDNIRWLLKKTNTNTVVLHSFAHLSESKSSPEFAQKLIEGLKNSLEERHIITHVTPYGYFLEFKLHVLGESLAKVFKSF
>gi|406900694|gb|EKD43573.1| hypothetical protein ACD_72C00217G0002 [uncultured bacterium]
MRALILHTNRFETKVIMGSNWPKGIISETVSSTEKIELENCLTIFFCVEESDTEKQLDELYTEIIKTSDEIQTNNLLISPFVHLSNNIAKPALAKSLYERLVKKIDNTKYNVKSSPFGYHKSLMLDIKGHPGSFRYREFY
>gi|493987620|ref|WP_006930409.1| hypothetical protein [Caldithrix abyssi]
MRLLMIYCTKFAYKTSVKGLEQVDELHESAQFENALVGFIHVEPDDEQHLSTVQTKLVKNLKWAARKNETNRIVLHSFAHLSEEKGDPELTKKIFDNAEERLKSAGYETAQTPFGYFLDIDVQAPGHSFARLFKEF
>gi|921240230|ref|WP_053184666.1| hypothetical protein [Sunxiuqinia dokdonensis]
MKVLVMYVNEFSYTPAEKNLEEAETVLNGKTIADAILAFIQVEEGDELKDVKSREKKLVNHLKWTARKNNCKRIILHSFAHLSESKASVEFTKSLFDEAEKRLQNADFETFQTPFGYFLDLKIDAPGYSLARIWATL
>gi|931442889|gb|KPK27437.1| hypothetical protein AMJ61_05690 [Desulfobacterales bacterium SG8_35_2]
MKLLLFYAHSFSYETAAKSLPGIADVYKKESVADAAVIFFHVEAEDAEKRSKVVQKFVKNVKWLCGKFATRNVVLHSFNHLSGSKADPVFSKEILEEVTERLARTAFQVMVTPFGYFNEFTIHVAGDSLAKVFKEF
>gi|498213209|ref|WP_010527365.1| hypothetical protein [Thermophagus xiamenensis]
MKVLVFYTKKFAYKTAEQNLSNEELNIFYPDIKEINHQGEYSNCITAFIQVEKEDEEKGVKSREKKLANHLKWVARKNNTHSILLHSFAHLSSSKASLKFTADLLKAACTRLQNGDYEASITPFGFFLDINMEAPGFSMARVWGDL
>gi|498107944|ref|WP_010422100.1| hypothetical protein [Anaerophaga thermohalophila]
MKVLVFYTRRFAYKTAEQNLSDEEIALFYPGSKEIVHEAEHTNCITAFIQIEAEDEEKGLKSREKKLANHLKWVARKNNTSSILLHSFAHLSASKASLKFTAELFHAVEARLKNGNYEASQTPFGFFLDLNMEAPGYSMARVWGDL
>gi|938913275|ref|WP_054696123.1| hypothetical protein [Desulfosarcina cetonica]
MRVLFWYCDRFAWQPTLKTLDDAPEATPMACDQAVVAFIHVEPADVVDGSSAETKLVKNAKWLARKWETREVVLHSFTHLGEAKAEPAAAGTLIDRVQQRLDAAGYLAVKTPYGYFNDLALQAPGHPLARIYKEF
>gi|515147175|ref|WP_016775851.1| hypothetical protein [Anaerophaga thermohalophila]
MKVLVFYTKKFAYKTAEQNLSDEEIALFYPGDKEIVHEGEHTNCITAFIQAEAEDEEKGLKSREKKLANHLKWVARKNNTSSILLHSFAHLSASKASLKFTAELFNAVEERLKNGNYDPSQTPFGFFLDLNMEAPGFSMARVWGDL
>gi|769891745|ref|WP_045033176.1| hypothetical protein [Draconibacterium sediminis]
MKVLVMYVDEFSYQPAEKNLEDVEDITEGAQFSDSILAFIQVEESDEEKDVKSREKKLVNHLKWTARKNDCKSVILHSFAHLSESKASVEFTKELFYLAEKRLQNADFTTAQTPFGYFLDLNIKAPGFSLARIWATL
>gi|740777457|ref|WP_038562741.1| hypothetical protein [Draconibacterium orientale]
MKVLVMYVEEFSYQPAQKNLEEVEEITAGAQFSDSILAFIQVEESDEEKDVKSREKKLVNHLKWTARKNNCKSVILHSFAHLSESKASVEFTKELFDLAEKRLQNADFTTAQTPFGYFLDLNIKAPGFSLARIWATL
>gi|952995948|ref|WP_057954645.1| hypothetical protein [bacterium L21-Spi-D4]
MKLLLIYANNFGYTPTTKTLEDAEAHTESHDFEKVQTAFIQAEAEDADREADVTKKLVKNLKWIMKKNEAETLILHSFAHLSESKADPDLTKSIFDKAEEKMKNAGYTVHQTPFGYFLDLRLDAPGLSLARVFKDL
>gi|504026813|ref|WP_014260807.1| hypothetical protein [Desulfovibrio africanus]
MKLLMFYAPEFWHKPFQKTLPEAPEAEPTGAMARDAVVVFYHGEAEDHDRRSSVLSKMLKNIKWLGGKFNTKQVVLHSFGHLSESKADPEFVRELVAEVRQRLENTGYSVGETPFGYLNEWKLHVAGDSLAKVFKSI
>gi|931450213|gb|KPK34090.1| Archaea-specific editing domain of threonyl-tRNA synthetase [Nitrospira bacterium SG8_35_1]
MKILMFYSPSFWFRTFEKVLDDVADQEADKTEVNAIVIFYHIEAEDVERKSKILTRLIKNIKWIAGKFKTSRLVLHSFNHLSTSKAPPDFSAALMSEARERLVRSGFEITETPFGYLNEWKMHVAGDSLAKVFKDL
>gi|931386354|gb|KPJ76195.1| hypothetical protein AMJ54_12595 [Deltaproteobacteria bacterium SG8_13]
MRVLFWYCERFGWDPTIKTLDDAPDAVPDTRDKAVVAFIHVEPDDAGGESSAETKLVKNAKWLARKWETKRVVLHSFTHLGEQKAEPETARALIERARQRLDKAGYRAVETPYGYFNDLTLQAPGHPLARIYKEF
>gi|492830085|ref|WP_005984039.1| hypothetical protein [Desulfovibrio africanus]
MKLLMFYAPEFWHKPFQKTLPEAPEAEPTGAMARDAVVVFYHGEAEDHDRRSSVLTKMLKNIKWLGGKFNTKQVVLHSFGHLSESKADPELVRELVAEVRQRLENTGYSVSETPFGYLNEWKIHVAGDSLAKVFKSI
>gi|1011161568|ref|WP_062083045.1| hypothetical protein [Bacteroidales bacterium 6E]
MKVLTMFVTQFGYEPAEKNLESAELVTQGALFENAILAFIQVEEADESYDVGSREKKLVNHLKWVCRKNNCNKVILHSFAHLSASKAKAGFTKELFDLAEARLKNASFETAQTPFGYFLNLDIKAPGFSLARIWTEL
>gi|1011203906|ref|WP_062124034.1| hypothetical protein [Geofilum rubicundum]
MKVLCFYTSKFSYTIGDKNHEEADEPALEGRFSSCIVAYIQAEETDETQDILSREKKLTNHLKWVARKNNTESIVLHSFAHLSASKASLSFTRELFKATKARLENGHYQTAITPFGYFLNLSMEAPGFSMARIWADL
>gi|498350280|ref|WP_010664436.1| hypothetical protein [Marinilabilia salmonicolor]
MKVLVFYTKRFAYKTAEQNLPDEDLKVFYPDTAVIKHQEEHKDCITAFIQVEAEDEERDLKSREKKLANHLKWVARKNNCSDILLHSFAHLSASKASLKFTAQLFDATRKRLNNGDYTASQTPFGFFLDLDMEAPGFSMARVWGDL
>gi|651361124|ref|WP_026473474.1| hypothetical protein [Alkaliflexus imshenetskii]
MKVLCFYTTSFSYTIGEKNHEEAIDAPCPASFAECIVAYIQVEEADELQDVKSREKKLSNHLKWVARKNGTEKIVLHSFAHLSSSKASMQFTRSLFDAALERLQNGGYQVSVTPFGYFLNLKMEAPGFSMARIWAEL
>gi|931409955|gb|KPJ97537.1| Archaea-specific editing domain of threonyl-tRNA synthetase [Nitrospira bacterium SG8_35_4]
MKILMFYSPEFWFKTYEKVLPDVPVQDADTSVKNAVVVFYHAEAGDPEREGSVLKKFIKNIKWLAGKFGTTNVVLHSFNHLSTSKAPPDFTCSLIDNAKERLAGSGFIITETPFGYLNAWKMHVAGESLAKVFKDI
>gi|818686197|gb|KKT31428.1| Threonyl-tRNA synthetase editing domain protein [Microgenomates group bacterium GW2011_GWF1_44_10]
MKLLMFHTKKFLYKPFISDSAGSEKTTLENSLVAFIHVEENDKEKSDIINKAVGNIKWLANKNNTNTVVLHSFAHLSNNKSDPETANDLIQKISEKLKKSLTTHIVPFGQFYEFSMHVMGPSLAKVFKDL
>gi|818713923|gb|KKT58094.1| Threonyl-tRNA synthetase editing domain protein [Candidatus Amesbacteria bacterium GW2011_GWA1_44_24]
MKLLIFHAKEFWYKPYSSASATEKTNFENCLVIFIHVEEVDKDKGDMIGKAVGNIKWLADKNKTSSVVLHSFAHLSNSKSDPETANEMIQKISERLRKNLSTHIVPFGQFYEFSVHVLGPSLAKVFKDL
>gi|818602806|gb|KKS51929.1| Threonyl-tRNA synthetase editing domain protein [Candidatus Gottesmanbacteria bacterium GW2011_GWA1_42_26]
MKLLMFHAESFWYKPYNSASTETEKTSFENCLVIFIHVEEIDKDKGDVIGKAVGNIKWLADKNKTNSVVLHSFAHLSNSKSDPETANEMIKKISEKLKKNLSAHIVPFGQFYEFSVHVLGPSLAKVFKDL
>gi|818475169|gb|KKR30575.1| hypothetical protein UT61_C0004G0002 [Candidatus Woesebacteria bacterium GW2011_GWA1_39_8]
MKLLMFHAKSFWYKPYNSDSSETEKTIFQNCLVIFIHVEEADKGKSDVVDKAVGNIKWLANKNKTTVVVLHSFAHLSTSKSDPDTANDIIHKMNEKLEKSLSTHIVPFGQFYEFSIRVLGPSLAKVFKDL
>gi|818523015|gb|KKR75771.1| Threonyl-tRNA synthetase editing domain protein [Candidatus Levybacteria bacterium GW2011_GWA2_40_8]
MKLLMFHTESFWYKPYKSNSSETEKRIFQNCLVIFIHVEEADKGKSDVVDKAVGNIKWLANKNKATIVVLHSFAHLSTSKSDPDTANDLIHKINEKLEKSLSTHIVPFGQFYEFSVHVLGQSLAKVFKDL
>gi|818344476|gb|KKQ26419.1| Threonyl-tRNA synthetase editing domain protein [Microgenomates group bacterium GW2011_GWC1_37_12b]
MKLLMLHTESFWYKPYNSDSSKTEKTIFQNCLVIFIHVEEADKGRNDVIDKAVGNIKWLANKNKVTIVVLHSFAHLSTSKSDPDTANDIIHKINEKLEKRLSTHTVPFGQFYEFSVHVLSQSLAKVFKDL
>gi|503673960|ref|WP_013908036.1| hypothetical protein [Thermodesulfatator indicus]
MKIIMFYALEFSWKPYQKVLDSGEDTPKETSLSKTVVVFYQVEAEDPAREKKVVEKLIKNIKWLARKFDTKAVVLHSFNHLSSSKASPEESYAIIEKAKEKLKRTGFNIYETPFGWLNEWKMHVAGESLAKVFKEI
>gi|551230526|ref|WP_022854300.1| hypothetical protein [Thermodesulfatator atlanticus]
MKILMFYAPEFWWKPYQKVLPQAEEGFSETTVKNAVVVFYQVEEEDPAREKKVVEKLVKNIKWLARKFDTKNIVLHSFNHLSVSKASPEEGLALINKAKEKLLRAGFNLYETPFGWLNEWKMHVAGESLAKVFKDL
>gi|503023238|ref|WP_013258214.1| hypothetical protein [Desulfarculus baarsii]
MKLLMFYAPSFWFKTHEKALAEAPEADIEQTVEHAVVVFYQVEAHDVDDQTATINKTIKNIKWLSRKFQAGAVVLHSFNHLSASKAPPQAAQALMEAAAQRLAAVGLTVQQTPFGYLNEWRLHAAGPALAKVFKEF
>gi|931449200|gb|KPK33154.1| hypothetical protein AMK70_09670 [Nitrospira bacterium SG8_35_1]
MKLLLFYAHSFAYETAAKSLPTVPEVKKKERVTDAAVIFFHVEAEDREKRSKVIQKFVKNVKWLCGKFGAGNVVLHSFNHLSSSKADPEFSQAVLEEVAERLTKKGFAVMLTPFGYFNEFAIHVAGDSLAKVFKEF
>gi|500016339|ref|WP_011697057.1| hypothetical protein [Syntrophobacter fumaroxidans]
MKLLMFHAPEFWFKTFDKAIPDAPDCKEEKSFRQAVVIFYQVEGEDVGREDGIVSKWVKNAKWIAGKFATRTVILHSFNHLSLSKAPPETAHRLAAQVRGRLERAGFSVFETPFGYLNEWKLHVSGESLAKVFKEI
>gi|1044670335|gb|OCC14217.1| hypothetical protein DBT_2359 [Dissulfuribacter thermophilus]
MKLLLFFAKSFEYRPFKKVLDHVEDVSTQGAKWSNTVVVFFQFEEEDLEKGPELVRKLVKNIKWISGKFSTKTVVFHSFNHLSSSKAPSWFAQPKIDQAKKRLENSGYTVGETPYGYLNEWSIHVSGESLAKVFKEL
>gi|522078660|ref|WP_020589869.1| hypothetical protein [Desulfobacter curvatus]
MRILFWYCEKFSWKPTIKTLEDYPNADADERQNVVVAFIHVEPKDTQDGAGAETKLVKNAKWLARKWDVKTILLHSFTHLAEEKAEPDDAKGVLDRVQGRLETAGYEASQTAYGYFNDLLIEAKGHPLARIFKEF
>gi|916541391|ref|WP_051148482.1| hypothetical protein [Desulfospira joergensenii]
MKVLFWYCDRFAWTPTLKTLDQVPDSQPDEFTDTVVAFVHVEPKDLETGSSAETKLLKNAKWLARKWDVEKILLHSFTHLAREKADPGRAKALLDRAEARLSKAGYQASQTAYGYFNDLSMEARGHPLARIFKEF
>ThrRS_gi|499569338|ref|WP_011250121.1| threonine--tRNA ligase [Thermococcus kodakarensis]
MRMLLIHSDYLEYEVKDKALKNPEPISDEQKTGRLDEVLAVFISVEKVDETNPDEVVEKAVKEIEDVASQIKAERIFVYPFAHLSSELAKPDVALEVLRKIEEKLREKGYEVKRAPFGYYKAFKLSCKGHPLAELSRTIV
>ThrRS_gi|887492400|pdb|4RRF|A Chain A, Editing Domain Of Threonyl-trna Synthetase From Methanococcus Jannaschii With L-ser3aa
MKMLLIHSDYLEFEAKEKTKIAEETENLKGKLDECLACFIAVEREDENNPEGTAIGAVEEIEKVANQLKVNNIVVYPYAHLSSDLSSPETAVKVLKDIESILKERGYNVLRAPFGWYKAFKISCKGHPLSELSRKIVAKEE
>ThrRS_gi|887492416|pdb|4RRQ|A Chain A, K121m Mutant Of N-terminal Editing Domain Of Threonyl-trna Synthetase From Pyrococcus Abyssi With L-ser3aa
MRVLLIHSDYIEYEVKDKALKNPEPISEDMKRGRMEEVLVAFISVEKVDEKNPEEVSLKAIEEISKVAEQVKAENVFVYPFAHLSSELAKPSVAMDILNRVYQGLKERGFNVGKAPFGYYMAFKISCKGHPLAELSRTIVPEEARVE
>ThrRS_gi|974124415|gb|KUO39163.1| hypothetical protein AVW05_01905 [Hadesarchaea archaeon DG-33]
MRILLVHSDFLEFEAKQRTPLAEDVSSEQRSGRLEEVLVVFTAAEEEDGSNIEGVSKNAAREIAEVARKVEAKRVAIYPYAHLSSSLASPKAAIKLLDATAGALREFGLEVHRLPFGWYKAFKLSCKGHPLSELSRTITA
>ThrRS_gi|985672555|gb|KXB03237.1| hypothetical protein AKJ48_04080 [candidate divison MSBL1 archaeon SCGC-AAA261O19]
MRILLIHADFLEFEAKEEAPVAEEVPEGMKSGRAEETLVVFMAVEDSDEVNPDAVVKNALSEIKEVQEEVGAQSIVLYPYAHLSDSLASPEVAIQVLDGLRNGLEEAGVDVLRLPFGWYKAFQLSCKGHPLSELSRTITP
>ThrRS_gi|851303025|ref|WP_048166335.1| threonine--tRNA ligase [Methanosarcina thermophila]
MQLLLIHSDYIEYETKKQTPVAEKIEESLKSGRLEEALTAFMAVESVDEANPEETIEKTVSEIEKVAAQVKTNRIMLYPYAHLSSDLSSPKTAVQVLKGVEAALSGKYEVKRAPFGWYKAFSISCKGHPLSELSRSIRP
>ThrRS_gi|73919808|sp|Q74MP3.1|SYT_NANEQ RecName: Full=Threonine--tRNA ligase; AltName: Full=Threonyl-tRNA synthetase; Short=ThrRS
MRALFLHSNRIKVIARQKALKEADQLEKPEFDVNKEHLAVFVAVEHGDNLSVAEQLVEEIKKVLEKIGIKEKVVVLYPYVHLTNNPSSPKLAKEVLDKAYDLLKSEGFEVYKAPFGWYKEFEIHVKGHPLAELSRTIKP
>ThrRS_gi|166225513|sp|A1RY76.1|SYT_THEPD RecName: Full=Threonine--tRNA ligase; AltName: Full=Threonyl-tRNA synthetase; Short=ThrRS
MKTLLIHAKHFEYEAREKALDAAESIDGNRSGSFENALVVFVTVEKGDGSSQDVVEEAAADVLDVFRRVGASRVVVYPYAHLSDDLADPEEAKRVLSQLAERISSAGVPVSRAPFGWYKRFSVECYGHPLSELSRTIKP
>ThrRS_gi|850981479|ref|WP_048058916.1| threonine--tRNA ligase [Desulfurococcus kamchatkensis]
MRILLIHARRFSYSVVKPAVEEPEPLEEGGKTLMLENVLVVFTSIERGDDESIISSAVNEVINVFNQVKASSILLYPYAHLSPDLAPPFEAVKILNAFYTALKNTGLQVYKAPFGWYKSFTLECYGHPLSELSKTIKK
>ThrRS_gi|19886944|gb|AAM01759.1| Threonyl-tRNA synthetase [Methanopyrus kandleri AV19]
MRLLFIHADEMSFEARQKTKIAEEEPPIKEAEVEDCLVVFAAVQEADEENPKAIAEAAVEEIEDVAGELKADRIVLYPYAHLADDLASPDVAVEVLKRMEGLLKERGYEVVRAPFGWYKAFRLACKGHPLSELSRTVTP
>ThrRS_gi|499726405|ref|WP_011407139.1| threonine--tRNA ligase [Methanosphaera stadtmanae]
MRTLMIHSDYLRYKTRSKTKIAEDIDDEKRVSGVDEALVAFIAVEKEDEENPELIINKAVKEILNVQNKVNAENIVIYPYAHLSSSLSNPDIAQKILKGIEAELLDNNEAVLRVPFGWYKSFELSCKGHPLSELSRTIT
>ThrRS_gi|166225487|sp|A3MTU4.1|SYT_PYRCJ RecName: Full=Threonine--tRNA ligase; AltName: Full=Threonyl-tRNA synthetase; Short=ThrRS
MRVLYIHAERFSWESREPALDIRDEPGSGAAANALVVFVSVERGDSSDEEFLRRVARDVVETAEKVKATAVVIYPYAHLSNDLARPYVAKEVVNKLYEVVKSEFKGEVYKAPFGYYKAFEVKCLGHPLAELSRSFKP
>ThrRS_gi|304378426|gb|ADM28265.1| threonyl-tRNA synthetase [Ignisphaera aggregans DSM 17230]
MKLLTIHAKEFMFKPTERALDIFDEPITSERYENVLVAFITVEEDDEKDIDGVVTQAVNEIIDIYKKVGARGIVIYPYAHLSSSLAAPSVAKEIVSKVYEVLKNEGFNVVKAPFGWYKEFELHCYGHPLSELSRTIRP
>ThrRS-ed_gi|496365544|ref|WP_009074534.1| Ser-tRNA(Thr) hydrolase [Metallosphaera yellowstonensis]
MILLLIHASQFSYQVKEKAIEKAEEPEVRELSLENVLVVFTSVEKGDDDSTVEKAKESIMDVVKRVNATSVVIYPYAHLSQNLAEPSIALVMLRKLHERLREIGLNVSRAPFGWYKSFSLTCYGHPLSELSKRITNETEY
>ThrRS-ed_gi|497674632|ref|WP_009988816.1| Ser-tRNA(Thr) hydrolase [Sulfolobus solfataricus]
MIILFIHASDFSFNVKERAIKEPEEAKLKSIELKNTLVCFTTVEKGDDEEILSKAIDDILDVYSKVKADSVVIYPYAHLSSNLANPDTAIKILESLENLLKDKVKVYRAPFGWYKAFSISCYGHPLSELSRRIRKTEELE
>ThrRS-ed_gi|383795667|gb|AFH42750.1| Threonyl-tRNA synthetase-like protein (thrS-like) [Fervidicoccus fontis Kam940]
MRILQIHAKKFSFRTVSEALQKKDETPIHELSKENVLVAFISVERGDDEKVIENATESIAEHAKKVNANSIIIYPYAHLSSNLEKPDQSIKILDELSERLKDKKLEVARAPFGWYKEFELSSFGHPLAELSREFSSAVSLQIK
>DTD_gi|30749356|pdb|1J7G|A Chain A, Structure Of Yihz From Haemophilus Influenzae (Hi0670), A D-Tyr- Trna(Tyr) Deacylase
MIALIQRVSQAKVDVKGETIGKIGKGLLVLLGVEKEDNREKADKLAEKVLNYRIFSDENDKMNLNVQQAQGELLIVSQFTLAADTQKGLRPSFSKGASPALANELYEYFIQKCAEKLPVSTGQFAADMQVSLTNDGPVTFWLNV
>DTD_gi|68566302|sp|P0A6M4.1|DTD_ECOLI RecName: Full=D-aminoacyl-tRNA deacylase; AltName: Full=D-tyrosyl-tRNA(Tyr) deacylase
MIALIQRVTRASVTVEGEVTGEIGAGLLVLLGVEKDDDEQKANRLCERVLGYRIFSDAEGKMNLNVQQAGGSVLVVSQFTLAADTERGMRPSFSKGASPDRAEALYDYFVERCRQQEMNTQTGRFAADMQVSLVNDGPVTFWLQV
>DTD_gi|59797485|sp|O14274.1|DTD_SCHPO RecName: Full=D-tyrosyl-tRNA(Tyr) deacylase
MKAVIQRVLNASVSVDDKIVSAIQQGYCILLGVGSDDTPEDVTKLSNKILKLKLFDNAEQPWKSTIADIQGEILCVSQFTLHARVNKGAKPDFHRSMKGPEAIELYEQVVKTLGESLGSDKIKKGVFGAMMNVQLVNNGPVTILYDTKE
>DTD_gi|968121891|ref|NP_001304972.1| D-tyrosyl-tRNA(Tyr) deacylase 1 isoform 1 [Homo sapiens]
MKAVVQRVTRASVTVGGEQISAIGRGICVLLGISLEDTQKELEHMVRKILNLRVFEDESGKHWSKSVMDKQYEILCVSQFTLQCVLKGNKPDFHLAMPTEQAEGFYNSFLEQLRKTYRPELIKDGKFGAYMQVHIQNDGPVTIELESPA
>DTD_gi|499725640|ref|WP_011406374.1| D-tyrosyl-tRNA(Tyr) deacylase [Methanosphaera stadtmanae]
MKLVVQRVTSAKVEVNNNIVGKIGKGYLVLLGIKKTDTKKEADYMINKLMKLRVFEDEENKMNLSIQDIDGEILLIPQFTLYGDVTHNNRPSFSNAMKPTDAKKLFEYCCNECEKKVHTQKGEFGAFMDVNLVNNGPVTIIIEKEYNS
>DTD_gi|757138071|ref|WP_042692319.1| D-tyrosyl-tRNA(Tyr) deacylase [Methanobrevibacter oralis]
MKLVIQRVTNAKVEVDNKITGEIKEGLMVLVGFGLNDTTREVDYLASKLVKLRIFEDENEKMNLSIRDIGGKLLLVPQFTLYGRTKKNRPSFHKALNPTKASELFDYFVGICSKDVPVETGVFGAFMNVSLLNNGPVTILLEKEFD

tRNAPyl sequences
Hot, Sandy's Spring West         GGGGGCTCGGCCGAGGCGGCCACAGGGGCTCTATACCC-CTGCAGCCGGGTTCAACTCCCGGAGCCCCCG
unknown archaeon                  AGGGGCTTGGCCGAGGCGGCCACAGGGGCTCTATACCC-CTGCAGCCGGGTTCGACTCCCGGAGCCCCCG
Methermicoccus shengliensis     GGAGGGTTGGTC-CGG-GACCGCCAGGCCTCTACAGCCACGGTAGCTGGGTTCGACTCCCAGGCCCTTCG
MBG-E archaeon                    GGAGGGCAGGTCGGGGAGACCGCATGGACTCTAACTCC-ATGCAGCCGGGTTCGACTCTCGGGCCCTCCG
Methermicoccaceae archaeon      GGAGGGTCGGTCGGGAAGACCACACGGACTCTAACTCC-GTGCAGCCGGGTTTGACTCCCGGACCCTTCG
Bathyarchaeota archaeon          GGGGGATCGGCC-GGGGGGCCACAAGAGTTCTAACCTC-TTGGAGCCGGGTTCGACTCCCGGATCCCTCG
Archaeoglobus archaeon           GGGGGCCTAGTCGAGGTGACTACACAGATTCTAAATCT-GTGGAGCCGGGTTCAACTCCCGGGGCCCTCG
other sequences
>Chloroflexi bacterium RBG_13_51_36 tRNACys
GGCGGTGTAGCCAAGAGGTTAAGGCAGGGGTCTGCAAAACCCCGATGCGGCGGTTCGAATCCGCCCGCCGCCT
>LHC4sed (W8A-19) archaeon tRNASec
GGCGGGCTGCAGCGGGGGTGGAACTAAAGCTGCCCAGTCCCCTGCCTCGGACTTCAAGCTTCATGGGGATATCCGAGGAACCCGGTGAGGGTTCGGGGTTCGATGCCCTTGCAGGCGAAAGTCCTCCAGCCCGCCGCCA
>CP (AK8) archaea tRNASec
GGCGGACTGAAGCAGGGGTGGAGGAGCCTATCTAAGGGGCTCCGCCCAATCCCTTGCCCCGGACTTCAAATCCGTGGAACCCGGCAAGGGTTCGGGGTTCGATACACGTACTCTTATTAGGGTATGTTTGCGAAGATCCCCCAGTCCGCCGCCA
>BOG (Asgard) archaeon tRNASec
GGCCTTGCACGCTCCTGGTGGTCCGGGAGGGCGGACTTCAAATCCGTTTGGCTCGACAAGGGCCAGGGGTTCGATTCCCCTGCGAGGCCG
