# Supplementary material for: RNA-Dependent Cysteine Biosynthesis in Bacteria and Archaea
Source: mBio. 2017 May 9;8(3):e00561-17. doi: 10.1128/mBio.00561-17 (PMC5424206; doi:10.1128/mBio.00561-17)
Supplement: TEXT S2 [file mbo002173292s2.docx]

**SupplementaL Materials and Methods**

**Plasmids.** The open reading frames (ORFs) of the SepRS genes of Parcubacteria (KPJ56532) and Chloroflexi (3300002966.a:JGI24721J44947_1000093732) were codon-optimized and chemically synthesized. PCR products were seamlessly inserted into pET22 vector (Novagen) using Gibson Assembly (NEB). The ORFs of the SepCysS genes of Parcubacteria (KPJ56533) and Chloroflexi (JGI24721J44947_1000093733) were codon-optimized and cloned into pET15 and pET22 vectors for expression in *E. coli*. The *M. jannaschii* *O*-phosphoseryl-tRNA^Sec^ kinase (PSTK) and SepCysS genes were previously cloned into pET vectors (1, 2). The *M. maripaludis* SepRS was previously cloned into a pET vector (3). tRNA^Cys^ genes from the Parcubacteria DG_74_2 bin (Ga0111216_10418 and Ga0111216_103201 encoding Parcubacteria type A37 and archaeal type G37 tRNA^Cys^, respectively) were chemically synthesized and cloned using Gibson Assembly (NEB) into a modified pUC18 vector (T7 promotor adjacent to the SbfI restriction site). The *M. jannaschii* PSTK gene and the *E. coli* tRNA^Sec^ gene were cloned into the two cloning sites of pACYCDuet-1 (Novagen) to make pACYC-MjPSTK-EcselC (4). The *araC* and arabinose promoter cassette of pBAD-RSF (5) was transferred to pCDFDuet-1 (Novagen) to make pBAD-CDF. The ORF of the *E. coli fdhF* gene was cloned under the arabinose promoter to make pBAD-CDF-fdhF.

**The oligonucleotides used in this study are shown below.**

| Primer | Sequence | Application |
| --- | --- | --- |
| J22Hot.f | CTTTAAGAAGGAGATATACATATGGCCACCTTTGATGTGAACGAC | Chloroflexi (3300002966.a:JGI24721J44947_1000093732) SepRS gene amplification |
| J22Hot.r | GTGCTCGAGTGCGGCCGCGCTACGCTGTTTCGCTTTTTTAC |  |
| 22bJRS.f | GAAGGAGATATACATATGGACAACAACAAAACCAAAAACAAAG | Parcubacteria (KPJ56532) SepRS gene amplification |
| 22bJRS.r | GTGCTCGAGTGCGGCCGCATTTTTGATTTTTTTCACCTGGATTTC |  |
| 22bJCS.f | GAAGGAGATATACATATGATCTACAAACGCCAGAACAAAAAC | Parcubacteria (KPJ56533) SepCysS gene amplification for pET22 insertion |
| 22bJCS.r | GTGCTCGAGTGCGGCCGCTTTAATCAGGATCTCTTTGAACACTTC |  |
| 15bJCS.f | GCCGCGCGGCAGCCATATGATCTACAAACGCCAGAACAAAAAC | Parcubacteria (KPJ56533) SepCysS gene amplification for pET15 insertion |
| 15bJCS.r | CTTTGTTAGCAGCCGGATTATTTAATCAGGATCTCTTTGAACAC |  |
| 22JChot.f | CTTTAAGAAGGAGATATACATATGGAACAGAAACCGAGCCGTAGC | Chloroflexi (3300002966.a:JGI24721J44947_1000093733) SepCysS gene amplification |
| 22JChot.r | GTGCTCGAGTGCGGCCGCACGGCTGCTATATTTGTCCAGGATG |  |
| p22.Cter | GCGGCCGCACTCGAGCAC | pET22 backbone amplification |
| p22.Nter | CATATGTATATCTCCTTCTTAAAGTTAAAC |  |
| p15.Nter | CATATGGCTGCCGCGCGGCACCAGGCCGC | pET15 backbone amplification |
| p15.Cter | TAATCCGGCTGCTAACAAAGCCCG |  |
| Parcu_a.f | CGACTCACTATAGACGGGGTGGCGGAATCTG | archaeal G37 tRNA^Cys^ amplification |
| Parcu_a.r | GTACCCGGGGATCCCCTGGAGACGGGGGTAGGATTTG |  |
| Par_b.f | ACGACTCACTATAGGCGCGGTAGCCAAGTAGCTAAGG | bacterial A37 tRNA^Cys^ amplification |
| Par.b.r | GTACCCGGGGATCCCCTGGAGGCGCGGATGGGAATTGC |  |
| PucPar_a.r | CCCGTCTATAGTGAGTCGTATTAACCTG | pUC18 backbone amplification for archaeal G37 tRNA^Cys^ insertion |
| pUC.b.r | GCGCCTATAGTGAGTCGTATTAACCTG | pUC18 backbone amplification for bacterial A37 tRNA^Cys^ insertion |
| Puc3.f | CCAGGGGATCCCCGGGTACCGAGCTCG | pUC18 backbone amplification for archaeal and bacterial tRNA^Cys^ insertion |
| Parc37.f | GAACGCAGGAGACTGCAAATCTCCATTATGTAGG | mutagenesis of archaeal G37 tRNA^Cys^ |
| Parc37.r | CCTACATAATGGAGATTTGCAGTCTCCTGCGTTC |  |

**Overexpression and purification of recombinant Parcubacteria and Chloroflexi SepRS.** Recombinant pET22-sepS plasmids were transformed into *E. coli* BL21(DE3) for protein production. Cells were grown to OD_600_ 0.6 at 37 °C, cooled and supplemented with 1 mM isopropyl β-d-1-thiogalactopyranoside (IPTG). After induction cells were grown at 18 °C for 11 hours. Cells containing the C-terminally his-tagged proteins were resuspended in buffer A (0.1 M sodium phosphate pH 7.4, 0.15 M NaCl, 10% glycerol, 10 mM β-mercaptoethanol) and broken by ultrasonic treatment. After purification using Ni-NTA chromatography eluted proteins were concentrated and stored at -20 °C in buffer S (0.1 M sodium phosphate pH 7.4, 0.15 M NaCl, 50% glycerol, 10 mM β-mercaptoethanol). Prior to the aminoacylation assay, proteins were transferred to buffer A and their concentration was measured according to the theoretical extinction coefficients (225080 M^-1^ cm^-1^ for Parcubacteria SepRS and 201240 M^-1^ cm^-1^ for Chloroflexi SepRS) (6).

Genes encoding Parcubacteria and Chloroflexi SepCysS proteins were introduced into pET15b and pET22 plasmids, respectively. Protein production was executed in *E. coli* BL21(DE3) under the same conditions as for SepRS proteins. The collected cells were transferred to an anaerobic chamber (Coy Laboratories) for anaerobic protein purification. The cells were resuspended in buffer Ax (50 mM Tris-Cl pH 7.5, 0.3 M NaCl, 10 % glycerol, 10 mM imidazole) and lysed for 15 minutes with 1× BugBuster (Novagen); 15 U Benzonase (Sigma) and one cOmplete EDTA-free Protease Inhibitor Mixture tablet (Roche) were added to the sample. After purification using Ni-NTA chromatography eluted proteins were concentrated and stored at -80 °C in buffer Sx (50 mM Tris-Cl pH 7.5, 0.15 M NaCl, 50% glycerol). Prior to testing, proteins were transferred to buffer Sa (50 mM Tris-Cl pH 7.5, 0.15 M NaCl) and their concentration was measured according to the theoretical extinction coefficients (105660 M^-1^ cm^-1^ for Parcubacteria SepCysS and 82740 M^-1^ cm^-1^ for Chloroflexi SepCysS).

**Transcript preparation, purification and labeling.** After BstNI digestion to generate 3’-CCA end, tRNA^Cys^ isoacceptors were produced by *in vitro* transcription essentially as described before (3). The reaction was then loaded on a HiTrap DEAE FF column (GE Healthcare) and purified as described (7). Fractions containing the tRNA were concentrated, ethanol precipitated and resuspended in redistilled water. Prior to use tRNAs were heated to 78 °C for 3 minutes and slowly cooled to ambient temperature in the presence of 10 mM MgCl_2_ to facilitate renaturation. tRNA labeling on the 3’-terminal adenosine using [α-^32^P]-ATP (Perkin Elmer) was executed as described before, without variation (8).

**SepRS assay *in vitro*.** Enzyme’s activity was assayed in a reaction mixture containing 3 µM tRNA^Cys^, 6 mM ATP, 50 mM Tris pH 7.5 at 25 °C, 50 mM NaCl, 10 mM MgCl_2_, 5 mM dithiothreitol (DTT), 2 mM phosphoserine and trace amounts of appropriate ^32^P-labeled tRNA. The enzyme concentrations were 1.7 µM and 5.2 µM for Parcubacteria and Chloroflexi SepRS, respectively. Reaction was conducted at 37 °C in case of Chloroflexi and at 25 °C in case of Parcubacteria SepRS. From a 20 µl reaction aliquots (2.5 µl) were taken at given times and mixed with 2 volumes of stop solution (0.4 M NaOAc pH 5.2, 0.1 % SDS). Quenched reaction aliquots were then mixed with 2 volumes of P1 nuclease (0.1 mg/ml in 0.3 M NaOAc pH 5.2) and incubated at room temperature for an hour. After digestion 1.5 µl of each aliquot was spotted on a PEI-cellulose thin-layer chromatography (TLC) plate and developed in a buffer containing 10 % ammonium acetate and 5 % acetic acid. Upon completion TLC-plates were dried and separated radioactive spots of AMP and Sep-AMP (originating from the aminoacylated and free 3’-terminal adenosines of tRNA) were detected by imaging plates (Fuji Films, exposition time 24-48 h). Imaging plates were scanned on a Molecular Dynamics Storm 860 Phosphoimager, and radioactive spots corresponding to AMP and Sep-AMP were quantified using ImageJ (9). Percent of aminoacylated tRNA was then calculated from the ratio of signal intensity obtained for Sep-AMP radioactive spots divided by the total intensity corresponding to both AMP and Sep-AMP spots.

**SepCysS assay *in vitro*.** Activities of SepCysS enzymes were assayed in a reaction mixture containing 50 mM Tris pH 7.5, 50 mM NaCl, 10 mM MgCl_2_, 5 mM DTT, 8 mM ATP, 1 mM phosphoserine, 40 mM sodium sulfide, 0.1 mM pyridoxal phosphate, 5 µM Chloroflexi SepRS, and 20 µM Parcubacteria or 40 µM Chloroflexi SepCysS. This mixture was reconstituted anaerobically. Reactions were conducted at 37 °C and started by the addition of 5 - 20 µM appropriate tRNA^Cys^ variant combined with trace amounts of the same ^32^P-labeled tRNA. From a 20 µl reaction aliquots (2.5 µl) were taken after 20 minutes, quenched and digested as before. 1.5 µl of each digested sample was then spotted on a PEI-cellulose TLC plate and developed in 1 M acetic acid, titrated to pH 3.5 with ammonium acetate.

**SepCysS assay using *E. coli*.** The Δ*selA* phenotype of the *E. coli* JS1 strain (2) transformed with pACYC-MjPSTK-EcselC and pBAD-CDF-fdhF was complemented by introducing plasmids expressing one of the three SepCysS proteins of *M. jannaschii*, Parcubacteria, and Chloroflexi. Overnight cultures of transformed cells were spotted on LB agar plates supplemented with 1 μM Na_2_MoO_4_, 50 mM sodium formate, 0.1% l-arabinose, 10 μM IPTG, 100 µg/mL carbenicillin, 34 µg/mL chloramphenicol, 100 µg/mL spectinomycin, and with or without 1 μM Na_2_SeO_3_ and incubated for one day at the room temperature in an anaerobic chamber. These agar plates were overlaid with a 0.75% top agar containing 250 mM sodium formate, 1 mg/mL benzyl viologen, and 25 mM KH_2_PO_4_ (pH 7.0) and incubated for a few minutes in the chamber.

1. **Sherrer RL, O'Donoghue P, Söll D.** 2008. Characterization and evolutionary history of an archaeal kinase involved in selenocysteinyl-tRNA formation. Nucleic Acids Res **36:**1247-1259.

2. **Yuan J, Hohn MJ, Sherrer RL, Palioura S, Su D, Söll D.** 2010. A tRNA-dependent cysteine biosynthesis enzyme recognizes the selenocysteine-specific tRNA in *Escherichia coli*. FEBS Lett **584:**2857-2861.

3. **Hohn MJ, Park HS, O'Donoghue P, Schnitzbauer M, Söll D.** 2006. Emergence of the universal genetic code imprinted in an RNA record. Proc Natl Acad Sci U S A **103:**18095-18100.

4. **Yuan J, Palioura S, Salazar JC, Su D, O'Donoghue P, Hohn MJ, Cardoso AM, Whitman WB, Söll D.** 2006. RNA-dependent conversion of phosphoserine forms selenocysteine in eukaryotes and archaea. Proc Natl Acad Sci U S A **103:**18923-18927.

5. **Haruna K, Alkazemi MH, Liu Y, Söll D, Englert M.** 2014. Engineering the elongation factor Tu for efficient selenoprotein synthesis. Nucleic Acids Res **42:**9976-9983.

6. **Wilkins MR, Gasteiger E, Bairoch A, Sanchez JC, Williams KL, Appel RD, Hochstrasser DF.** 1999. Protein identification and analysis tools in the ExPASy server. Methods Mol Biol **112:**531-552.

7. **Easton LE, Shibata Y, Lukavsky PJ.** 2010. Rapid, nondenaturing RNA purification using weak anion-exchange fast performance liquid chromatography. RNA **16:**647-653.

8. **Ledoux S, Uhlenbeck OC.** 2008. [3'-^32^P]-labeling tRNA with nucleotidyltransferase for assaying aminoacylation and peptide bond formation. Methods **44:**74-80.

9. **Schneider CA, Rasband WS, Eliceiri KW.** 2012. NIH Image to ImageJ: 25 years of image analysis. Nat Methods **9:**671-675.
